# Diurnal migration patterns in willow warblers differ between the western and eastern flyways

**DOI:** 10.1186/s40462-023-00425-x

**Published:** 2023-09-21

**Authors:** Kristaps Sokolovskis, Violeta Caballero-Lopez, Susanne Åkesson, Max Lundberg, Mikkel Willemoes, Tianhao Zhao, Staffan Bensch

**Affiliations:** 1 Department of Biology, Lund University, Sölvegatan 37, 223 62 Lund, Sweden; 2 Department of Biology, University of Turku, Vesilinnantie 5, 20500 Turku, Finland; 3 GELIFES, University of Groningen, Nijenborgh 7, 5172.0664, 9747 AG Groningen, Netherlands

**Keywords:** Barrier crossing, Geolocation, Migration, *Phylloscopus trochilus*, Plasticity, Passerine

## Abstract

It is a long-standing view that the main mechanism maintaining narrow migratory divides in passerines is the selection against intermediate and suboptimal migratory direction, but empirical proof of this is still lacking. We present novel results from a willow warbler migratory divide in central Sweden from where birds take the typical SW and SE as well as intermediate routes to winter quarters in Africa. We hypothesized that individuals that take the intermediate route are forced to migrate in daytime more often when crossing wide ecological barriers than birds that follow the typical western or eastern flyways. Analyses of geolocator tracks of willow warblers breeding across the entire Sweden, including the migratory divide, provided no support for our hypothesis. Instead, birds that migrated along the western flyway were the most likely to undertake full day flights. The probability of migrating for a full day when crossing major barriers declined linearly from west to east. We speculate that this difference is possibly caused by more challenging conditions in the western part of the Sahara Desert, such as the lack of suitable day-time roost sites. However, it may equally likely be that willow warblers benefit from migrating in daytime if favorable tailwinds offer assistance.

**Supplementary Information:**

The online version contains supplementary material available at 10.1186/s40462-023-00425-x.

## Background

Stable migratory divides are not uncommon in nature [1–3]. A fundamental hypothesis explaining the maintenance of migratory divides is that hybrid offspring inherit intermediate migratory direction and suffer higher mortality as a result [4, 5]. Under this assumption, maladaptive migratory routes can be characterized by more direct barrier crossings compared to more optimal routes that involve detours [6]. A recent stream of papers using geolocator data has demonstrated that nocturnally migrating passerines often extend their night flight well into day-time when crossing considerable ecological barriers [7–12].

The willow warbler *Phylloscopus trochilus* is a small, mainly nocturnally migrating old world passerine that breeds across the entire Eurasia and winters in tropical Africa [13]. In Europe, the southwestern subspecies *P. t. trochilus* migrates to western Africa through the Iberian Peninsula and the western part of Sahara Desert (western route), whereas the northeastern subspecies *P. t. acredula* migrates through the Balkan Peninsula to eastern and southern Africa (eastern route). In central Sweden, SE and SW migratory populations meet and interbreed [14]. The location of the migratory divide in central Sweden seems stable over time and is rather narrow, only ~ 250 km wide, suggesting that selection against intermediate migratory phenotypes keeps it stable [15]. We analyzed a data set of 72 willow warbler geolocator tracks from breeding sites across Sweden. The birds we tracked took a diverse range of routes that allowed us to compare flyway-specific behaviors. In addition to describing the patterns of full day time flights in willow warblers we wanted to answer the following questions. (1) Is full daytime migration more common in birds that take the intermediate route? (2) Does full day migration primarily take place over open water or desert crossing? (3) Are birds more likely to undertake a full day flight if they had begun the migration late in the season?

## Methods

### Fieldwork

We deployed 466 geolocators on adult male willow warblers: 60 in southern Sweden (Lat 55–58°N), 50 in northern Sweden (Lat 65–66°N) and 356 in the migratory divide in central Sweden (Lat 61–64°N). In 2018 we tagged 349 individuals across all the sites and retrieved data from 56 in 2019. An additional 117 loggers were fitted on birds in central Sweden in 2020 of which we retrieved 16 in 2021. The loggers were produced by Migrate Technology Ltd (model: Intigeo-W30Z11-DIP 12 × 5x4mm, 0.32 g). We used a nylon string to attach them to birds with the “leg-loop” harness [16].

### Geolocator data

The total data set included 72 tracks for autumn migration and 62 tracks for spring migration, because 10 loggers stopped recording soon after the onset of the spring migration. To extract location data from geolocators we used the R package *GeoLight* 2.0 [17]. Twilight events were obtained with a light threshold of 3 lux. The most extreme outliers were trimmed with the “loessFilter” function and using a K value of 3. For this study we carried out a “Hill-Ekström” calibration for the longest resided winter site. The equinox periods were visually identified by inspecting standard deviations in latitude. Latitudes from equinox periods were excluded (mean number of days removed due to equinox was 45, range 25 – 68). The timing of autumn departure from the breeding site was estimated by manual inspection of longitudes and latitudes plotted in time series. To estimate the barrier crossing longitude, we extracted the longitude when birds crossed latitude 35 N° in both, spring and autumn. For 29 birds in autumn, it was possible to directly extract the longitude at crossing latitude 35°N. For the remaining cases, the exact crossing of latitude 35°N was obscured by the equinox, therefore we calculated the mean longitude of the ten days following the onset of autumn equinox as an estimate of the barrier crossing longitude. For the spring migration, 48 birds crossed latitude 35°N after the equinox. The measure of barrier crossing was therefore indirectly estimated for 14 birds.

**Fig. 1:**
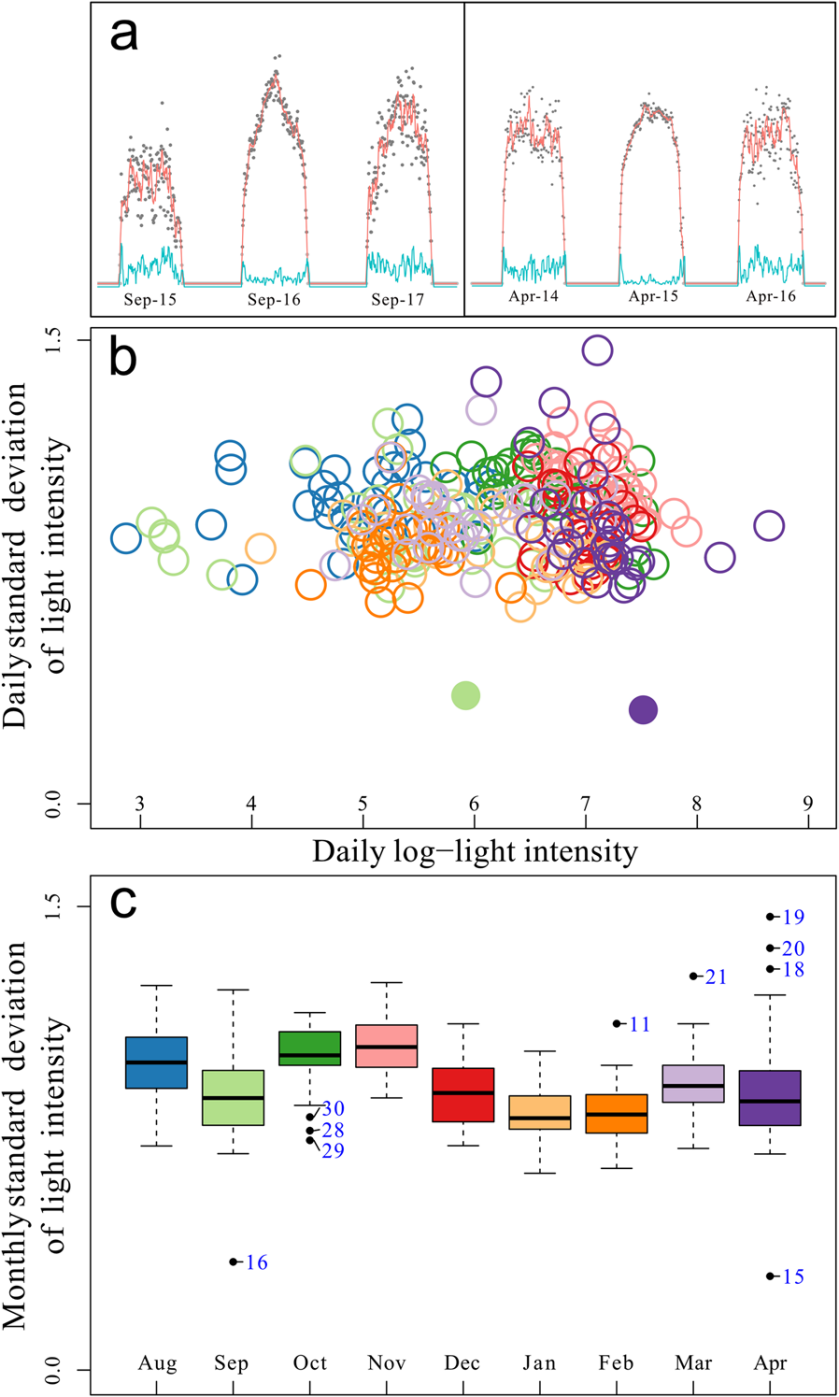
An example of the FDF identification workflow. **a** Two cases of three subsequent days for which the middle day shows high light intensity (red line) and low standard deviation (blue line); on one day in autumn (to the left) and one day in spring (to the right). **b** A scatter plot of daily mean light log intensity against daily mean standard deviation in light intensity. The two filled circles represent the two days from the annual cycle identified as FDFs by inspecting the plots shown in panel a. Colour of circles refers to months according to colour code in panel c. **c** Boxplot summarizing standard deviation in light intensity per month. Numeric labels near the outliers denote the dates of the corresponding month. In this case, two days (16th Sep and 15th Apr) clearly stand out. The examples are from the bird tagged with logger ID BM199

To identify when birds were flying during daytime, we visually inspected the year-round light patterns of light intensity (maximum lux value recorded every five minutes) plotted on log scale and identified days with unambiguous smooth curves that also showed decreased standard deviation in lux values (Fig. 1a, Additional file 1). This is not a fully objective approach as it relies on case-by-case human decisions. We only analyzed Full Day Flights (FDF) and excluded patterns that spanned less than a full day. With Full Day Flights, we only refer to migratory flights during the light part of the day. To strengthen our confidence in correctly assigning FDFs, we also created scatterplots with mean daily standard deviation of light intensity against mean daily log-transformed light intensity, expecting that both measures will clearly identify the days of non-stop flight (Fig. 1b, Additional file 2). Standard deviation proved to be a more robust measure of FDF than light intensity. The light intensity was in many cases rather low despite very clear FDF patterns, possibly due to the birds flying under an overcast sky or partial shading of the sensor on the logger with feathers. We further created boxplots of the standard deviations of the lux values for each month of each individual and inspected the days that showed outlier values (data points outside 1.5 times the interquartile range below the lower quartile). Among the outliers from the boxplots, we found 90% of the dates manually identified as FDFs. This gave us confidence that our manually identified FDFs are reliable (Fig. 1c, Additional file 2). The loggers we used did not record activity data. Therefore, we do not know whether the birds were in active flight during the night before or after the FDF and thus, we present and discuss our data without this assumption.

### Statistical tests

We used logistic regression models to understand whether barrier crossing longitude and departure timing affected the probability of the birds to carry out FDFs. The response variable in all models was a factor with two levels (whether the bird committed an FDF or not). We used “glm” function from base R and set family = 'binomial'. For linear effects of barrier crossing longitude and departure timing on FDF, we ran simple binomial regressions. For testing whether intermediate barrier crossing longitudes are associated with more frequent FDFs, we added a quadratic effect of crossing longitude (Table 1). To identify over which of the ecological barriers, the Mediterranean Sea, Sahara Desert or the Arabian Peninsula, the birds committed FDFs, we estimated the nearest available positions before and after each FDF (for the full set of maps illustrating the location estimates of FDFs including instances where ecological barrier crossing was impossible to identify, see Additional file 4). Normality of all data sets and subsets was evaluated with the Shapiro normality tests. All work was carried out in R version 4.1.1 (R core team 2021). Additional file 3 contains all data we used in the analyses.

**Table 1:** Results of logistic regression models. The longitude at which barrier crossing took place in each season has been abbreviated as “long”

Models and variables	β	Std. Error	Z	Pr(> Chi)
Autumn FDF ~ long AIC: 78.33				
Intercept	0.12	0.32	0.38	
Long	− 0.11	0.03	− 3.70	0.00002
Spring FDF ~ long AIC: 74.96				
Intercept	1.34	0.43	3.12	
Long	− 0.06	0.02	-3.43	0.0002
Autumn FDF ~ long + I(long^2) AIC: 80.21				
Intercept	0.17	0.34	0.49	
Long	− 0.09	0.05	− 1.78	0.00002
I(long^2)	− 0.001	0.003	− 0.35	0.72
Spring FDF ~ long + I(long^2) AIC: 76.78				
Intercept	1.45	0.50	2.84	
Long	− 0.08	0.06	− 1.28	0.0002
I(long^2)	0.0005	0.001	0.42	0.67
Autumn FDF ~ breeding site departure AIC: 96.71				
Intercept	3.92	8.720	0.45	
Breeding site departure	− 0.02	0.04	− 0.52	0.60
Spring FDF ~ winter site departure AIC: 88.91				
Intercept	0.22	1.40	0.15	
Winter site departure	0.0006	0.02	0.03	0.98

## Results

For none of the birds we recorded more than one instance of FDF per season, 17 individuals carried out FDFs in both spring and autumn, and 25 did not do FDFs in either season. More than half (56%) of the tracked birds carried out FDFs in spring and about one third (35%) in autumn.

**Fig. 2:**
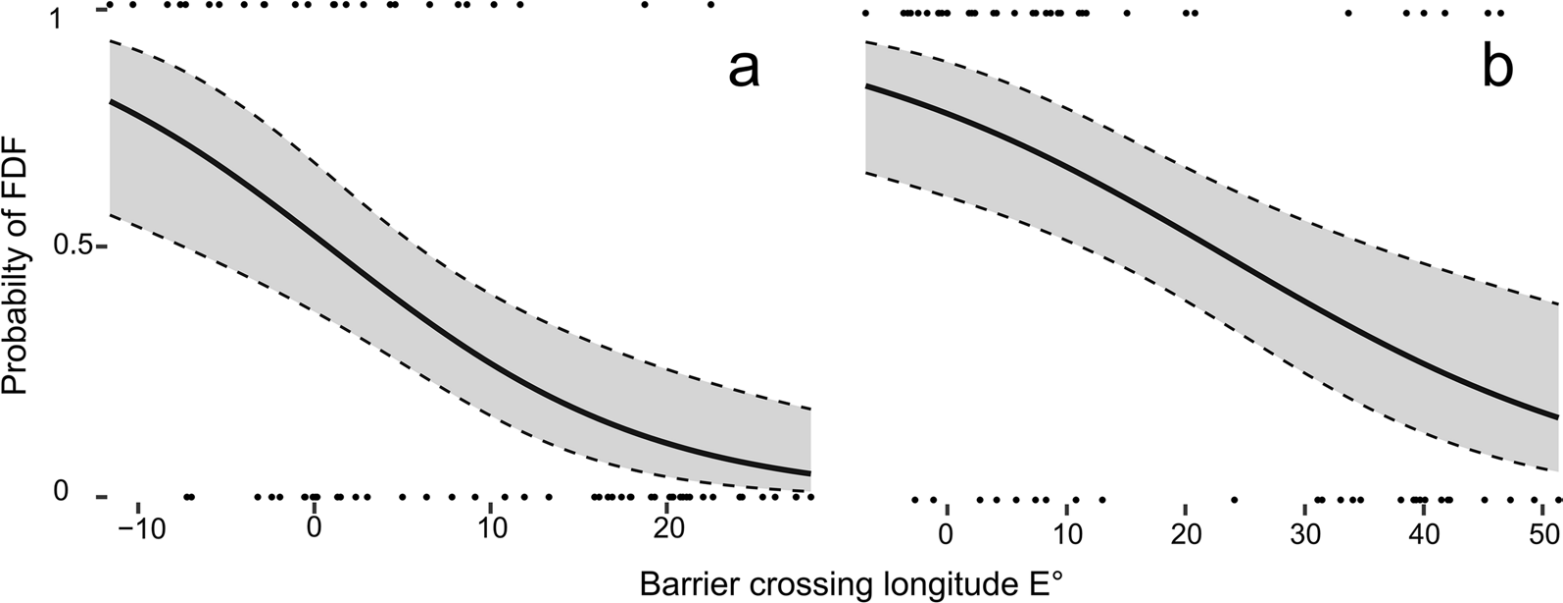
The probability of willow warblers undertaking a FDF in autumn (**a**) and spring (**b**) migrations relative to the barrier crossing longitudes. Grey shading shows 95% confidence interval

**Fig. 3:**
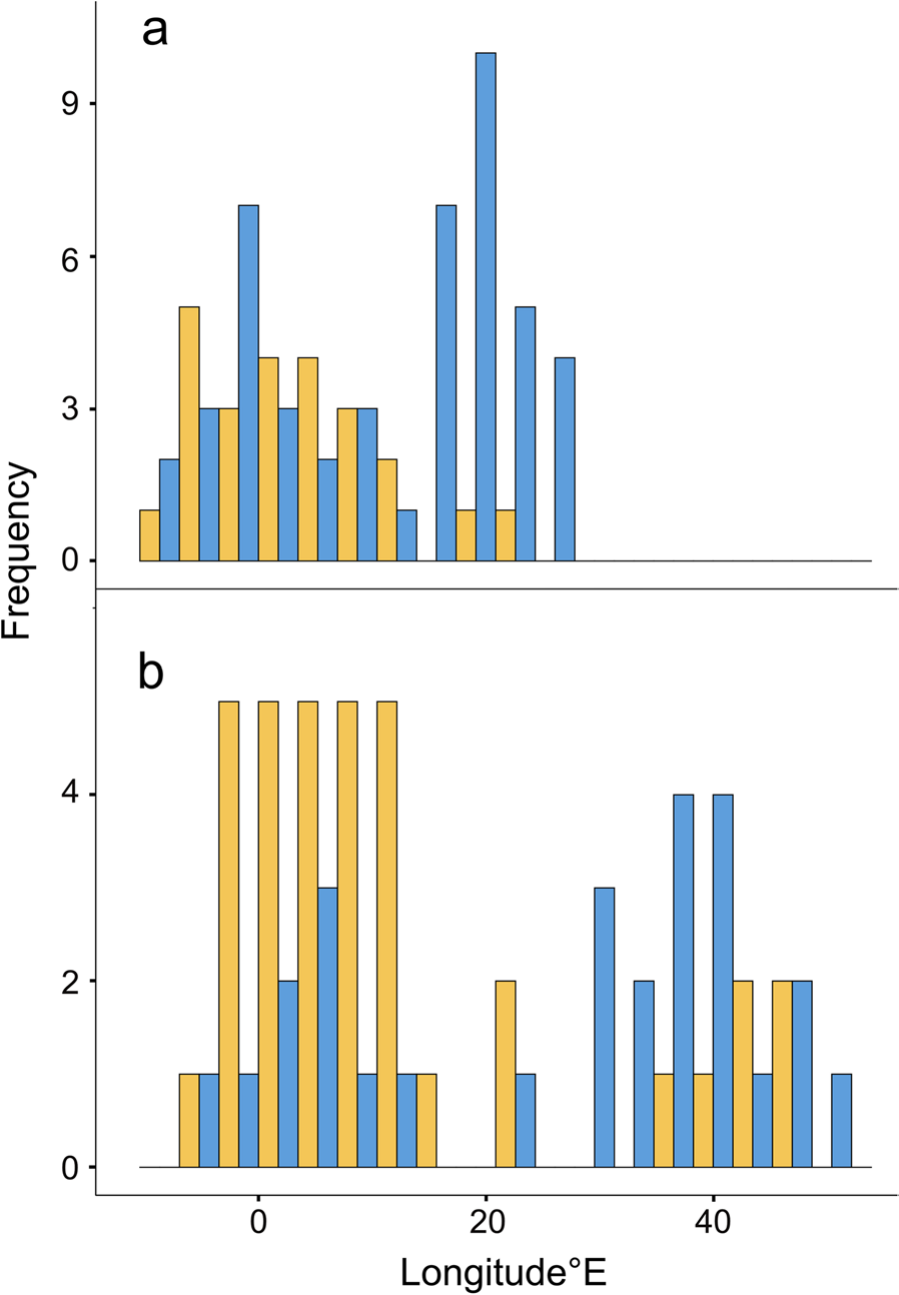
Barrier crossing longitudes for autumn (**a**) and spring (**b**). The X-axis shows the longitude and the Y-axis the frequency of birds undertaking FDFs (orange bars) and birds that did not (blue bars)

The probability of birds undertaking a FDF decreased significantly the more easterly longitude they used for barrier crossing (Figs. 2, 3), both in autumn (*P* = 0.00002) and in spring (*P* = 0.0002). The inclusion of a quadratic term of barrier crossing longitude in the model revealed no increased probability of undertaking FDF at the intermediate barrier crossing longitudes (autumn: *P* = 0.7, spring: *P* = 0.7, Table 1).

**Fig. 4:**
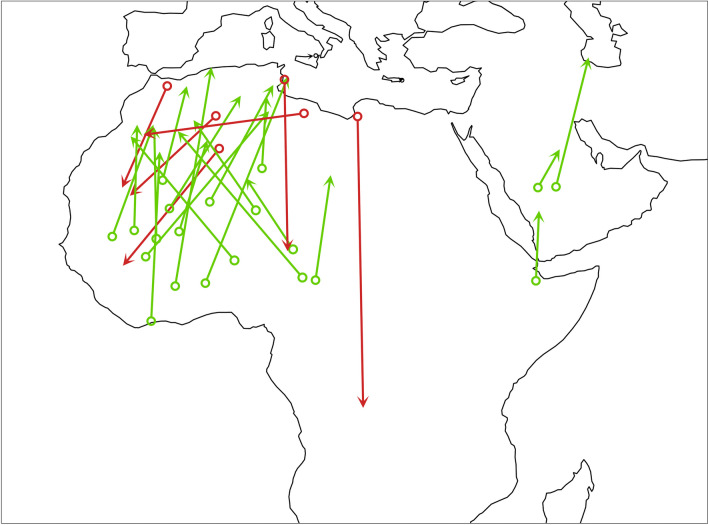
Estimated locations for 27 recorded FDFs. Red denotes autumn, green spring. The open circles show the last known position before a FDF and the arrowhead the first reliable position following the FDF. All the birds did only one FDF between the two points. Note that lines do not represent the routes between the two locations and the duration of travel between the two positions is always more than one day

About half of the FDFs occurred during the equinoxes. Nevertheless, for 27 (out of 60) instances we were able to identify in which of the major ecological barriers it took place. In all determined cases, the FDFs took place either over the Sahara Desert or the Arabian Peninsula (Fig. 4).

The timing of migration had no effect on the probability of undertaking a FDF in autumn (*P* = 0.6) or in spring (*P* = 0.6, Table 1). All the FDF events were recorded within the expected migration periods in autumn (median: Sep 20, range: Aug 27–Oct 13) and in spring (median: Apr 18, range: Mar 27–May 2).

## Discussion

We recorded FDFs in most of the tracked willow warblers. However, none of the birds undertook a FDF more than once per migration season. Nocturnally migrating songbirds generally have the capacity to fly in the daytime when crossing large ecological barriers [8, 11]. Some of the previous studies of this phenomenon have used the term “strategy”, implying that FDF is a genetically hardwired behavior. Instead, we argue that occasional day-time migration in nocturnally migrating birds is better viewed as phenotypic plasticity within the reaction norm [18] and hence should be referred to as a “tactic” [19]. Malmiga et al. (2021) used repeated tracks to show that individual great reed warblers *Acrocephalus arundinaceus* can alternate and carry out FDFs in one year but not the other, showcasing that it is indeed a tactic.

Willow warblers from northern and southern Scandinavia use the typical western and eastern flyways respectively. Our main hypothesis was that the willow warblers from a migratory divide taking the presumably inferior intermediate route [5, 20] will have the highest probability to undertake FDFs. Surprisingly, we found that the highest probability for FDFs was for birds that followed the western flyway. The probability of undertaking a FDF decreased linearly with more easterly barrier crossings. The intermediate route of willow warblers might still be less optimal in other ways, such as compromised foraging opportunities at stopovers or higher predation pressure. Songbirds migrating from Europe to Africa commonly deploy detours that prolong the total distance of the journey [6]. The precise reasons for why birds often do not take direct routes are yet to be understood.

Locations of FDFs showed that for the birds following the western flyway, most FDFs took place in the western Sahara as in several other species studied so far [8]. We were not able to identify locations of FDF in more than half of the cases, therefore it is possible some did take place over the Mediterranean Sea. European ortolan buntings *Emberiza hortulana* that migrate along the western flyway did not undertake FDFs in autumn but 20% did in spring. Russian and Belorussian ortolan buntings that follow the eastern flyway did not undertake FDFs in autumn or in spring. [8]. This is consistent with the general pattern we found in willow warblers. Different species have different life histories that often preclude meaningful comparisons of their behaviors in isolation. Except for ortolan buntings and our study, we are not aware of any other published data set that includes FDF patterns between flyways within the same passerine species. In a close relative to the willow warbler, the wood warbler *Phylloscopus sibilatrix*, all three tracked British birds undertook two consecutive FDFs in autumn [8], however wood warblers from more eastern populations have not been tracked yet. Out of two spring tracks, one bird carried out two consecutive FDFs and the other bird carried out one FDF. Wood warblers differ from willow warblers in that they take the intermediate route in autumn whereas the spring route follows the western edge of the Sahara [21, 22]. Wood warblers have pointier and longer wings than willow warblers suggesting better adaptation for long flights [23].

We expected birds which start the migration later in the season to compensate for the delay and have a higher probability to undertake FDFs to reach winter grounds faster but found no evidence of that. Previous studies of several songbirds have shown that daytime migrations are more common in spring than in autumn [8, 9]. This holds true in our data as well since many more willow warblers carried out FDFs in spring (56%) than in autumn (35%). A possible explanation is that in spring, males are under greater selection to reach breeding sites early to secure high quality breeding territories leading them to attempt barrier crossings without waiting for optimal weather conditions [7, 9]. According to this hypothesis, males should do more FDFs in spring than females. We cannot test this in the current dataset because we only tracked males. In great reed warblers and pied flycatchers *Ficedula hypoleuca,* however, there are no differences in FDFs between the sexes [7, 9].

One interpretation of the FDF pattern in willow warblers is that the conditions for migration along the western route are more challenging than along the eastern route. This is in accord with a recorded decline of *P. t. trochilus* but not *P. t. acredula* in Sweden. Between 1980 and 1999 there has been a significant decrease of willow warblers ringed at Falsterbo (ringing station in southern Sweden where primarily SW migrating *P.t. trochilus* are passing through) but no change in the number of willow warblers ringed at Ottenby (a ringing site on an eastern coastal island of Sweden where mostly SE migratory *P. t. acredula* pass through) [24]. Through satellite tracking, Hewson et al. (2016) established that among British common cuckoos *Cuculus canorus*, the southern populations that take the westerly route are declining more than the northern populations that migrate along a more easterly route [25]. It is important to note that we only obtained tracks from the birds that did survive the migration and successfully returned to the breeding sites, and therefore, we can only speculate whether FDFs indicate harsher conditions along the flyway.

A second interpretation of our result is that birds following the western flyway more often have the opportunity to take advantage of tail winds and then prolong the nocturnal flight in the daytime. Wind plays a crucial role in migratory behaviours of birds [26, 27]. It has been shown that flight departure is timed relative to favorable wind conditions [28] and that winds may influence route selection [29]. In a radar study across the western part of the Sahara Desert in Mauritania, Schmaljohann et al. (2007) further showed that diurnal flights involving predominantly nocturnal passerine migrants were associated with favorable tailwind assistance, suggesting the birds were opportunistically using the winds to extend their flight ranges [30]. Such diurnal flights were more numerous in spring as compared to in autumn [30]. We, therefore, suggest that this may be part of the explanation as to why our willow warblers crossing the western part of the Sahara Desert were more frequently involved in FDFs. Arboreal birds like willow warbler, which forage by gleaning and do not accumulate large fat reserves for migration, are thought to have fewer opportunities to find suitable stopping sites in desert areas [8]. Even though we think FDFs in willow warblers reflect challenges along the flyway, it is equally likely that FDFs result from birds benefiting from favorable tailwinds.

We could not show that the intermediate migratory route over the central Mediterranean and the Sahara Desert entails more frequent use of FDFs than the eastern or western flyway. Research programs on avian migratory divides [2, 15, 20] assume that the main mechanism maintaining narrow contact zones is selection against hybrids with intermediate migratory routes. However, this assumption remains to be demonstrated.

### Supplementary Information

**Additional file1**: Images (.png) with light intensity data used to identify FDFs. Red lines show log-transformed light intensity (lux) as a 25 min rolling mean. Blue lines depict rolling standard deviation in light intensity and grey dots show data points. The X-axis marks each calendar date

**Additional file2**: Plots (.pdf) that we used to verify FDF as outliers from normal light pattern during migration. Boxplots depict daily standard deviations in light intensity per month; outliers are labelled with the date of corresponding month. Scatter plots show daily values of light intensity (log transformed) against standard deviation in lux values. The filled circles denote FDF days we identified with manual inspection

**Additional file3**: Data table (.xlsx) used to perform the analyses and create all figures for the manuscript

**Additional file4**: Maps (.pdf) depicting locations where all the recorded FDFs took place. Black dots show closest reliable locations before and after the FDF event. The width of the red polygon shows the limits of standard deviation in longitude during the exact day when FDF took place

## Data Availability

All the data is attached as supplementary files. Raw geolocator data is available on dryad https://doi.org/10.5061/dryad.stqjq2c6t.

## Abbreviation

FDF: Full Day Flight
