# Supplementary material for: Diurnal migration patterns in willow warblers differ between the western and eastern flyways
Source: Mov Ecol. 2023 Sep 21;11:58. doi: 10.1186/s40462-023-00425-x (PMC10512566; doi:10.1186/s40462-023-00425-x)
Supplement: Supplementary file 2 — Additional file2: Plots (.pdf) that we used to verify FDF as outliers from normal light pattern during migration. Boxplots depict daily standard deviations in light intensity per month; outliers are labelled with the date of corresponding month. Scatter plots show daily values of light intensity (log transformed) against standard deviation in lux values. The filled circles denote FDF days we identified with manual inspection [file 40462_2023_425_MOESM2_ESM.pdf]

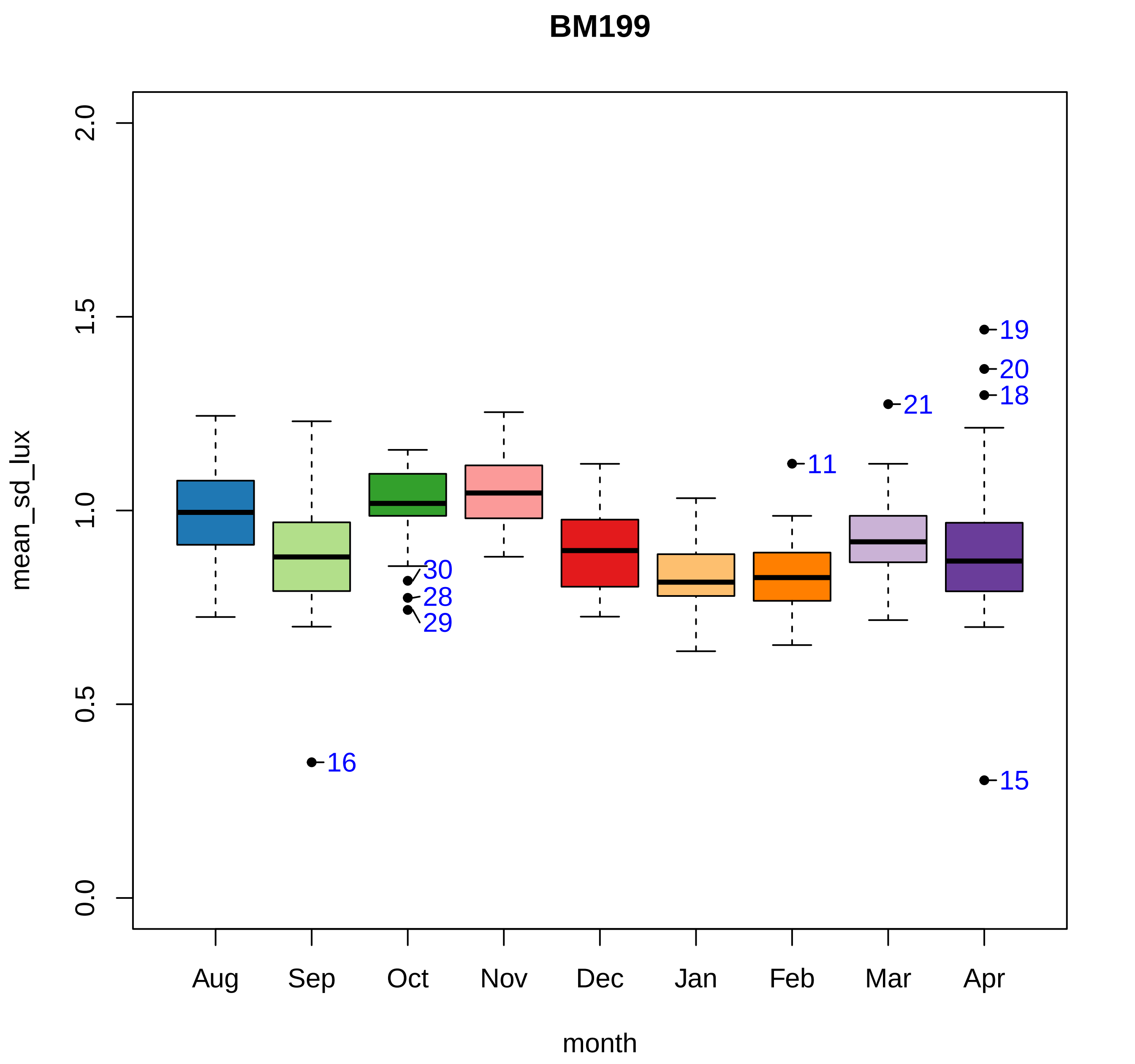
BM199
mean_sd_lux
0.0
0.5
1.0
1.5
2.0
Aug
Sep
Oct
Nov
Dec
Jan
Feb
Mar
Apr
month
16
30
28
29
11
21
19
20
18
15

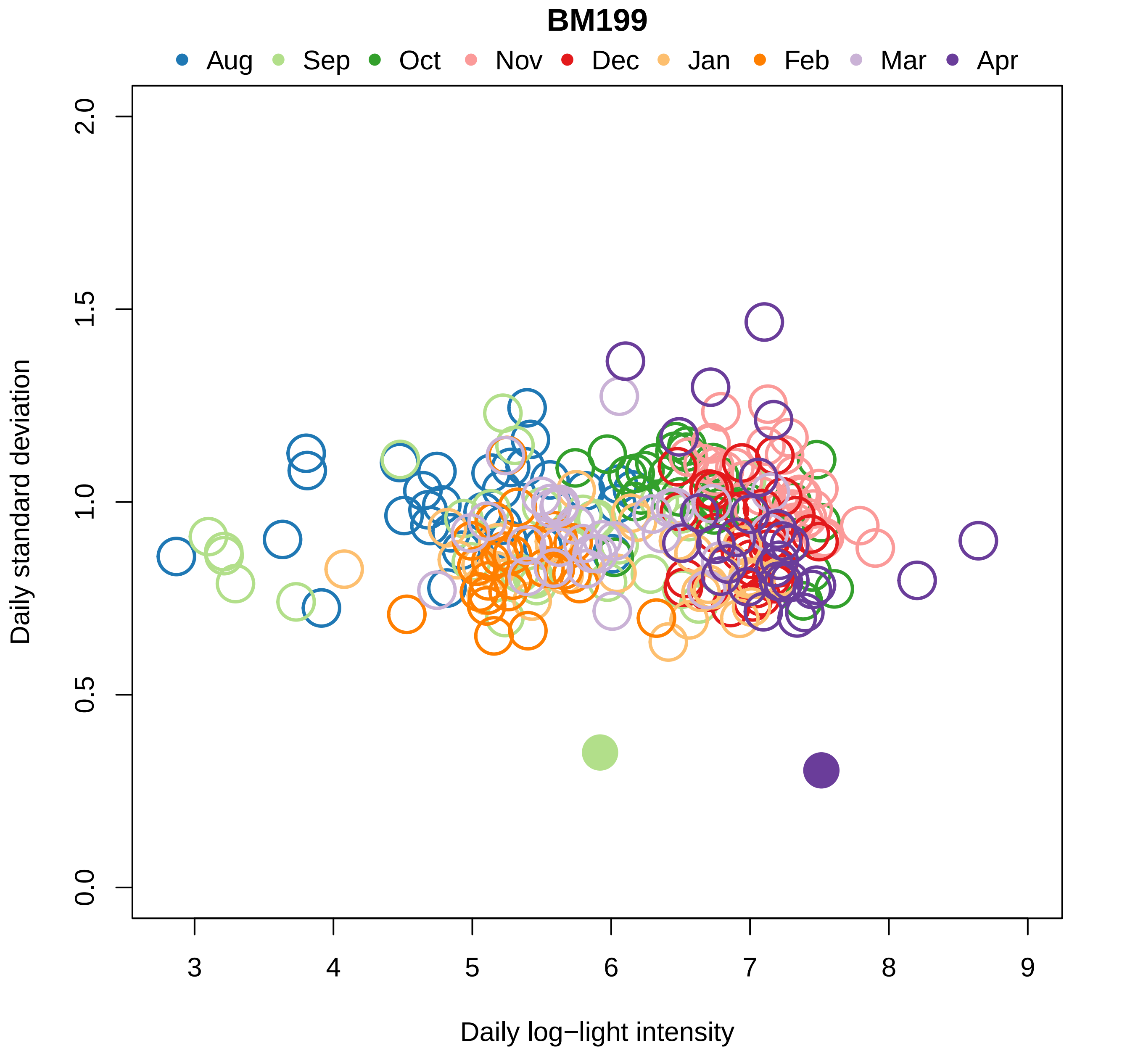
BM199
Aug
Sep
Oct
Nov
Dec
Jan
Feb
Mar
Apr
Daily standard deviation
0.0
0.5
1.0
1.5
2.0
3
4
5
6
7
8
9
Daily log−light intensity

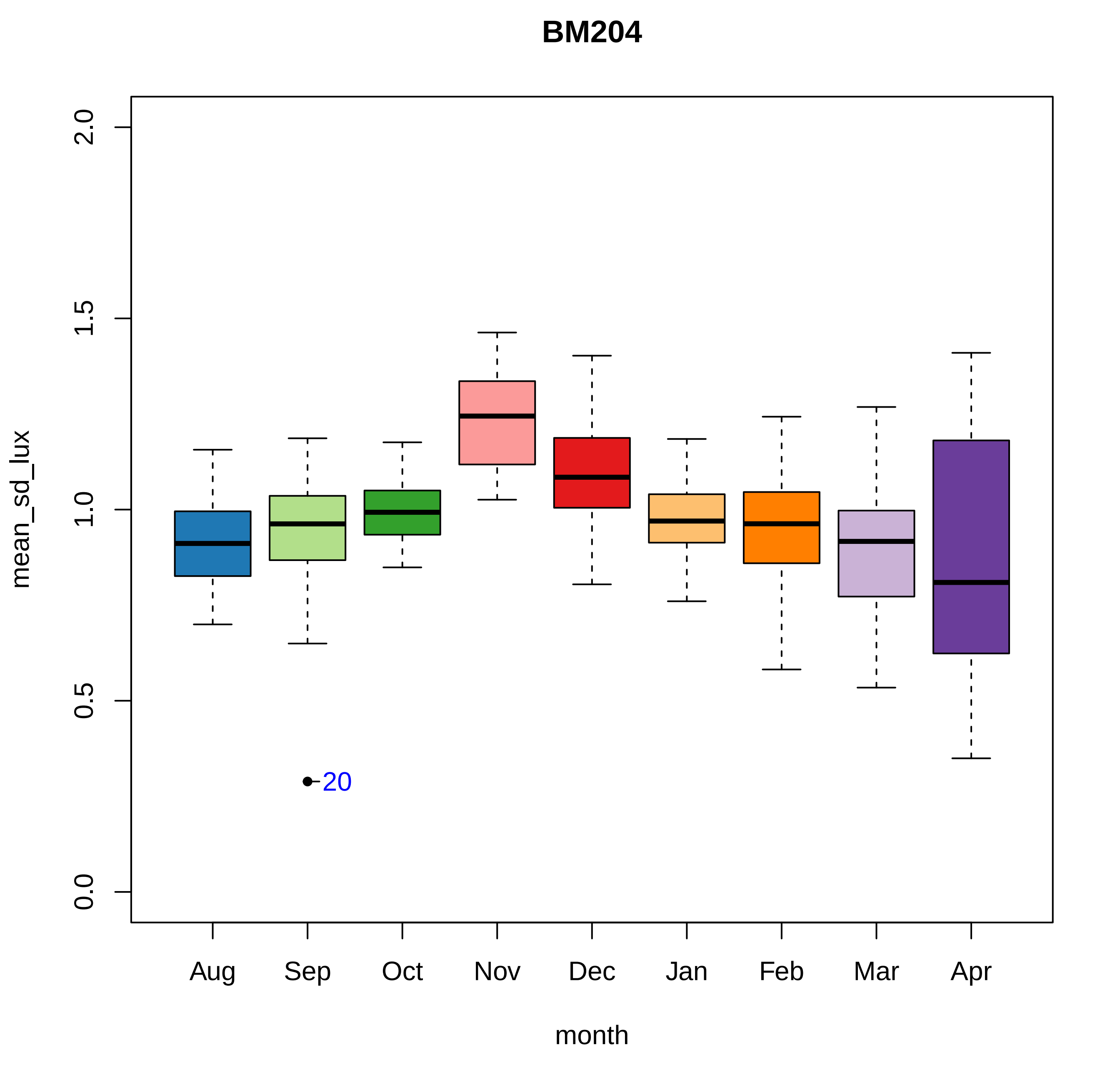
BM204
mean_sd_lux
2.0
1.5
1.0
0.5
0.0
20
Aug
Sep
Oct
Nov
Dec
Jan
Feb
Mar
Apr
month

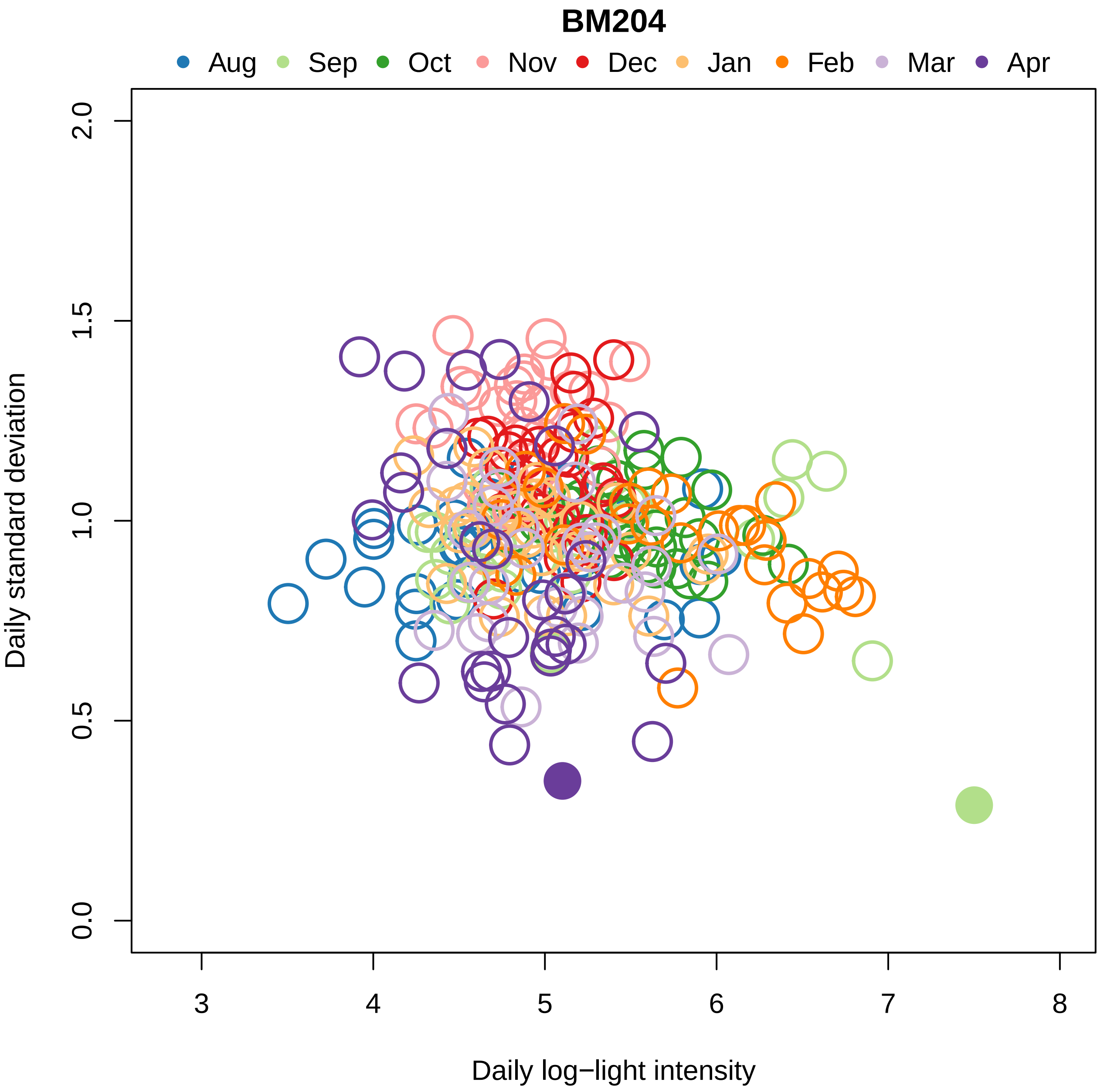

BM204
Aug
Sep
Oct
Nov
Dec
Jan
Feb
Mar
Apr
Daily standard deviation
0.0
0.5
1.0
1.5
2.0
3
4
5
6
7
8
Daily log−light intensity

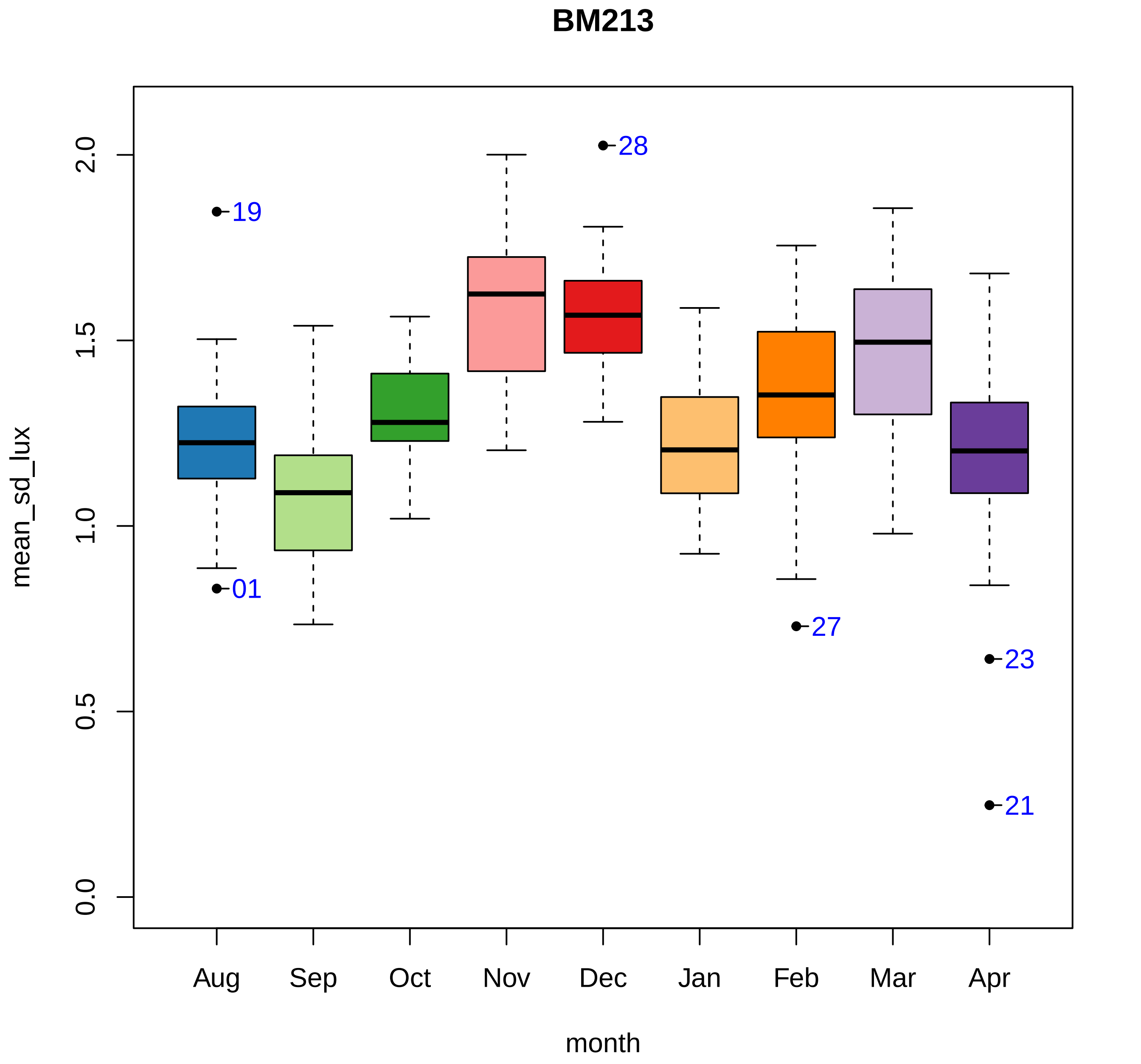

BM213
mean_sd_lux
0.0
0.5
1.0
1.5
2.0
19
01
28
27
23
21
Aug
Sep
Oct
Nov
Dec
Jan
Feb
Mar
Apr
month

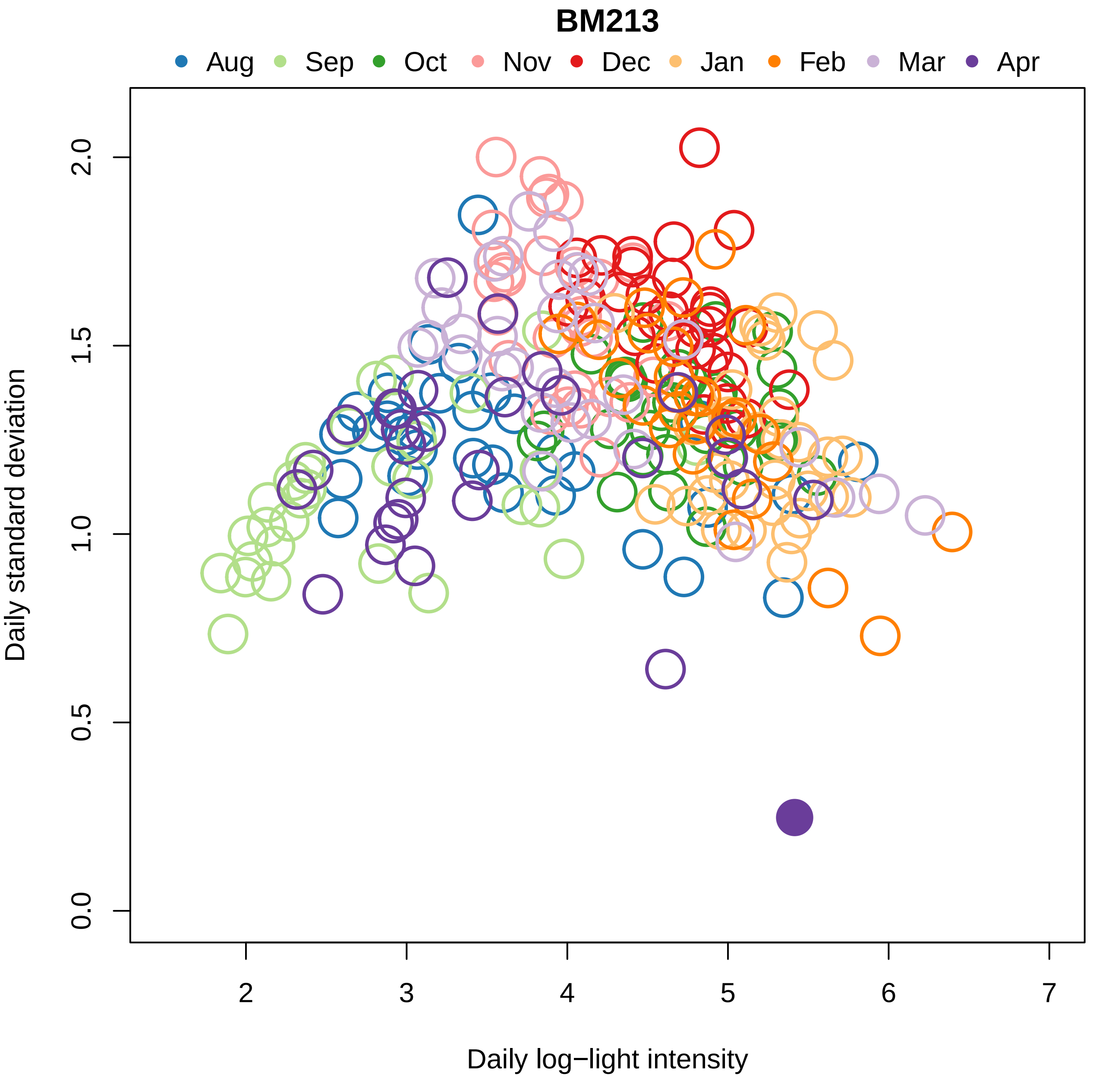
BM213
Aug
Sep
Oct
Nov
Dec
Jan
Feb
Mar
Apr
Daily standard deviation
0.0
0.5
1.0
1.5
2.0
2
3
4
5
6
7
Daily log−light intensity

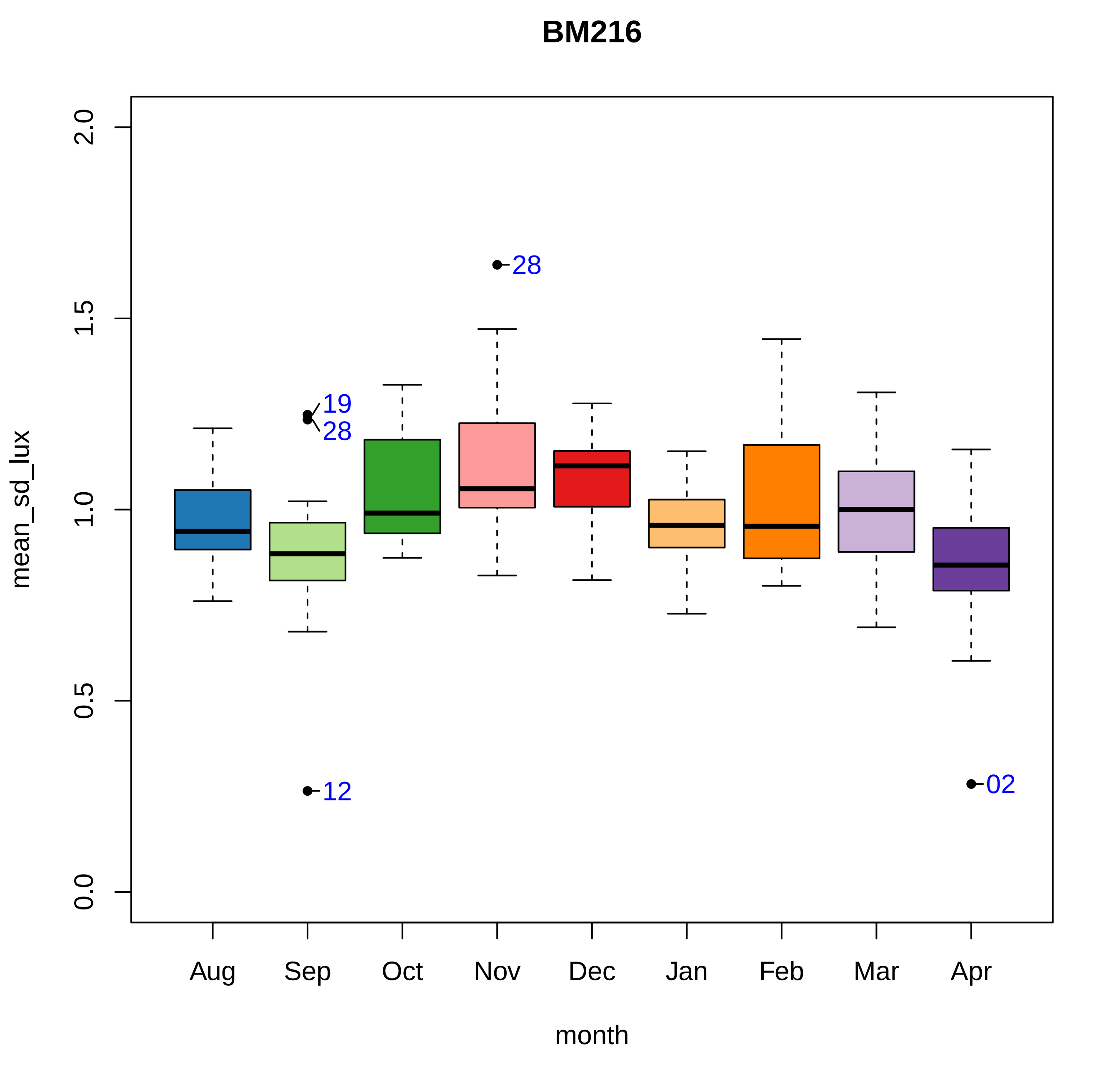
BM216
mean_sd_lux
0.0
0.5
1.0
1.5
2.0
Aug
Sep
Oct
Nov
Dec
Jan
Feb
Mar
Apr
month
19
28
12
28
02

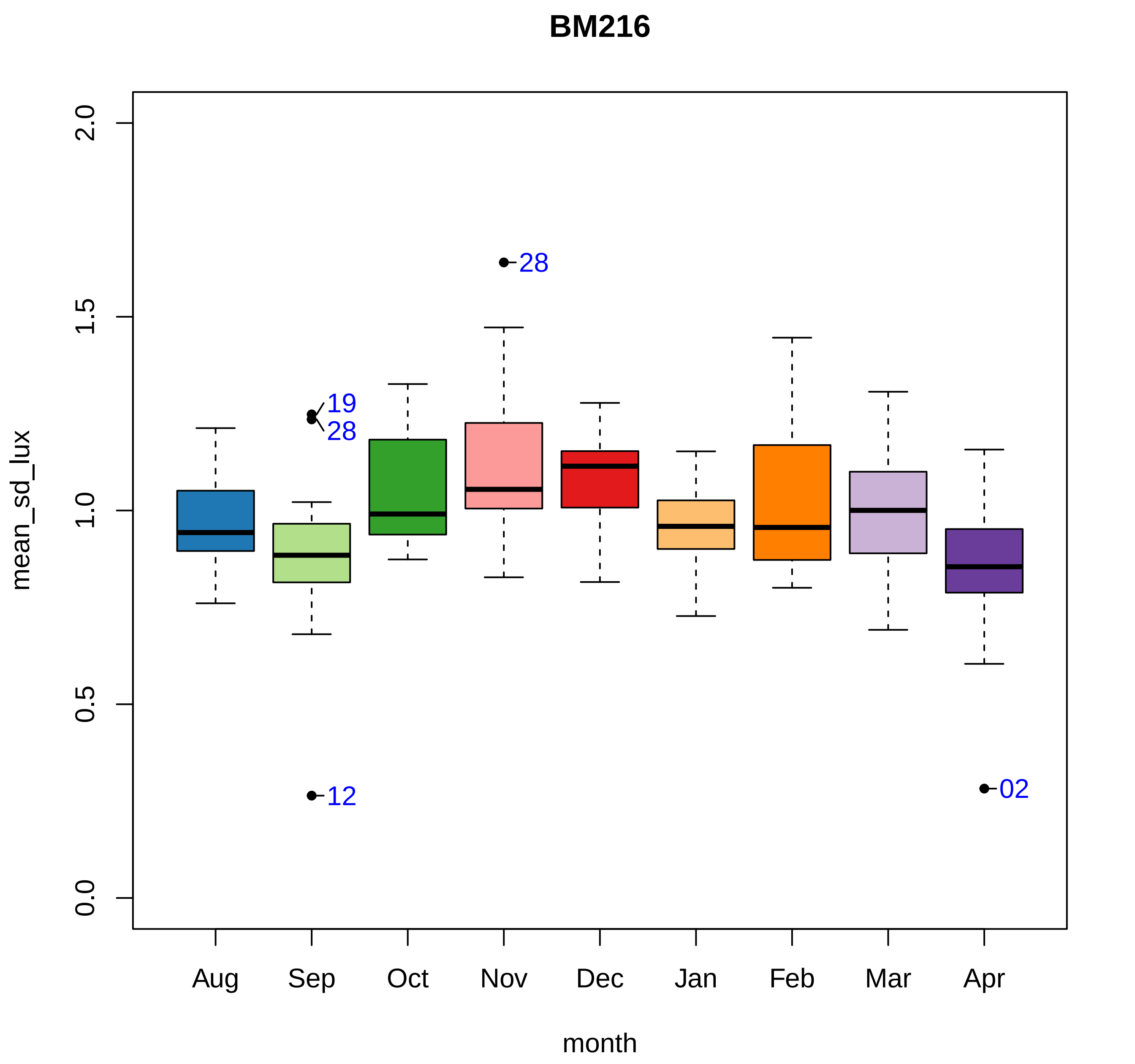

BM216
mean_sd_lux
0.0
0.5
1.0
1.5
2.0
month
Aug
Sep
Oct
Nov
Dec
Jan
Feb
Mar
Apr
19
28
12
28
02

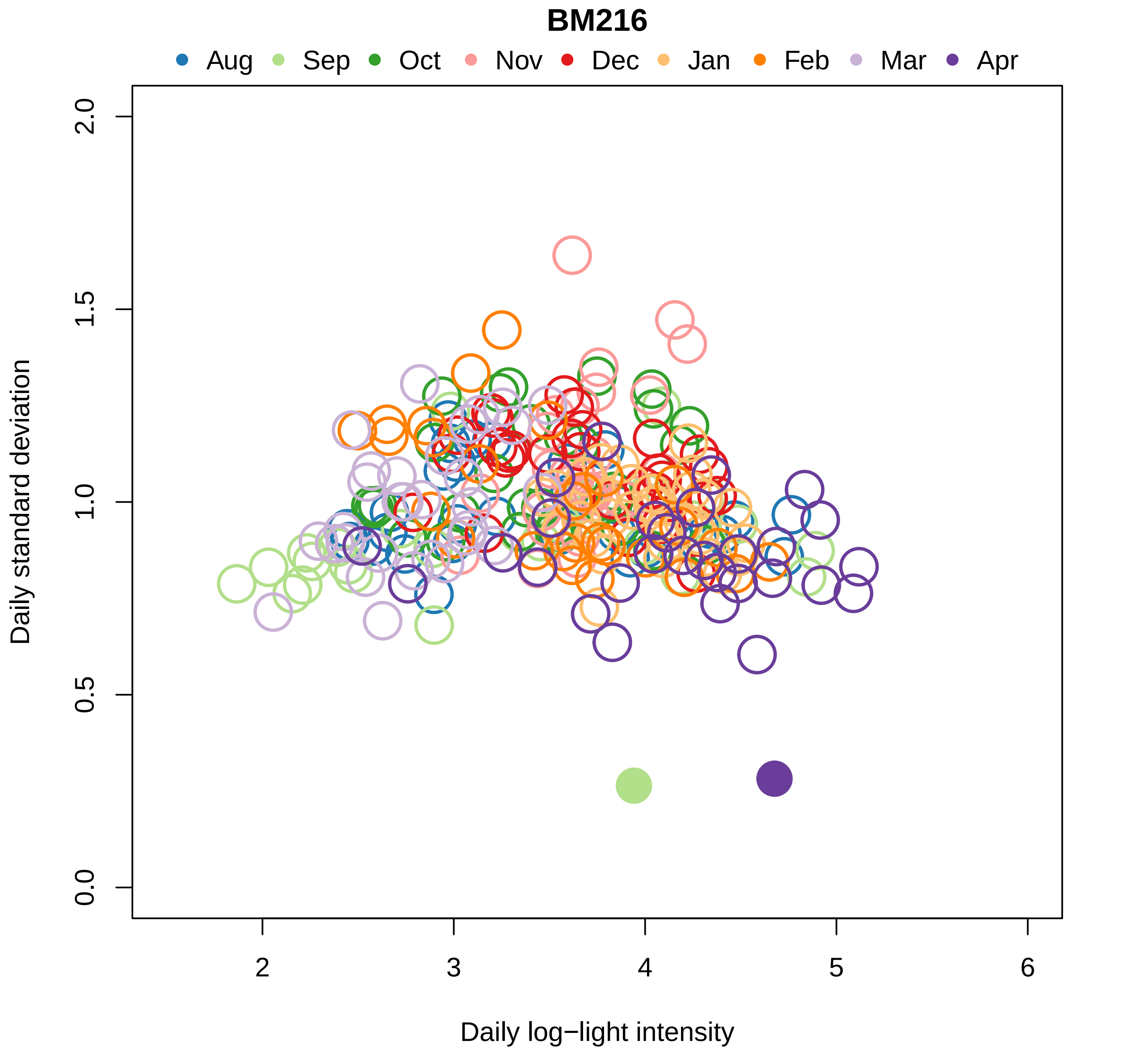

BM216
Aug
Sep
Oct
Nov
Dec
Jan
Feb
Mar
Apr
Daily standard deviation
0.0
0.5
1.0
1.5
2.0
2
3
4
5
6
Daily log−light intensity

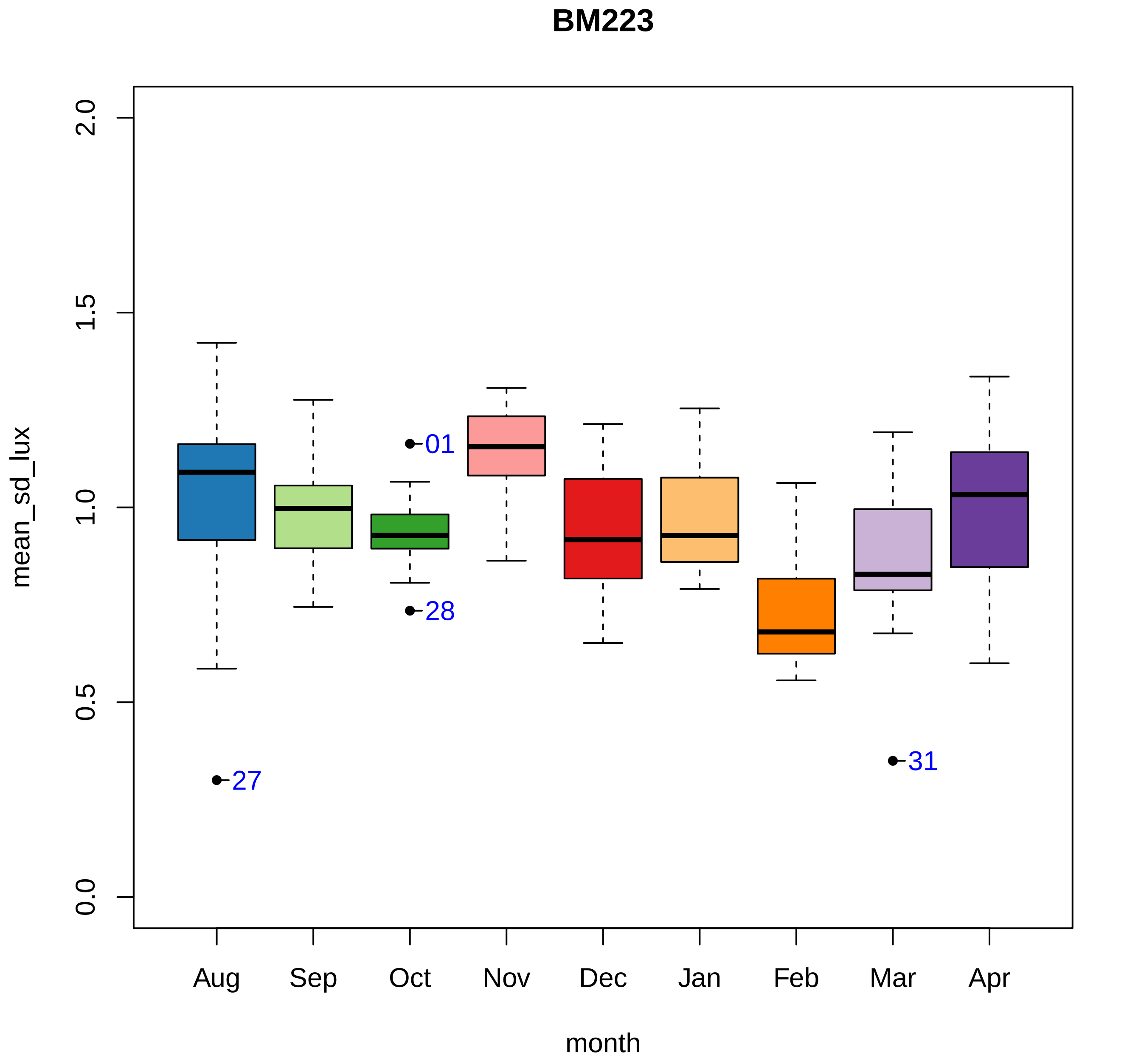
BM223
mean_sd_lux
2.0
1.5
1.0
0.5
0.0
01
28
27
31
Aug
Sep
Oct
Nov
Dec
Jan
Feb
Mar
Apr
month

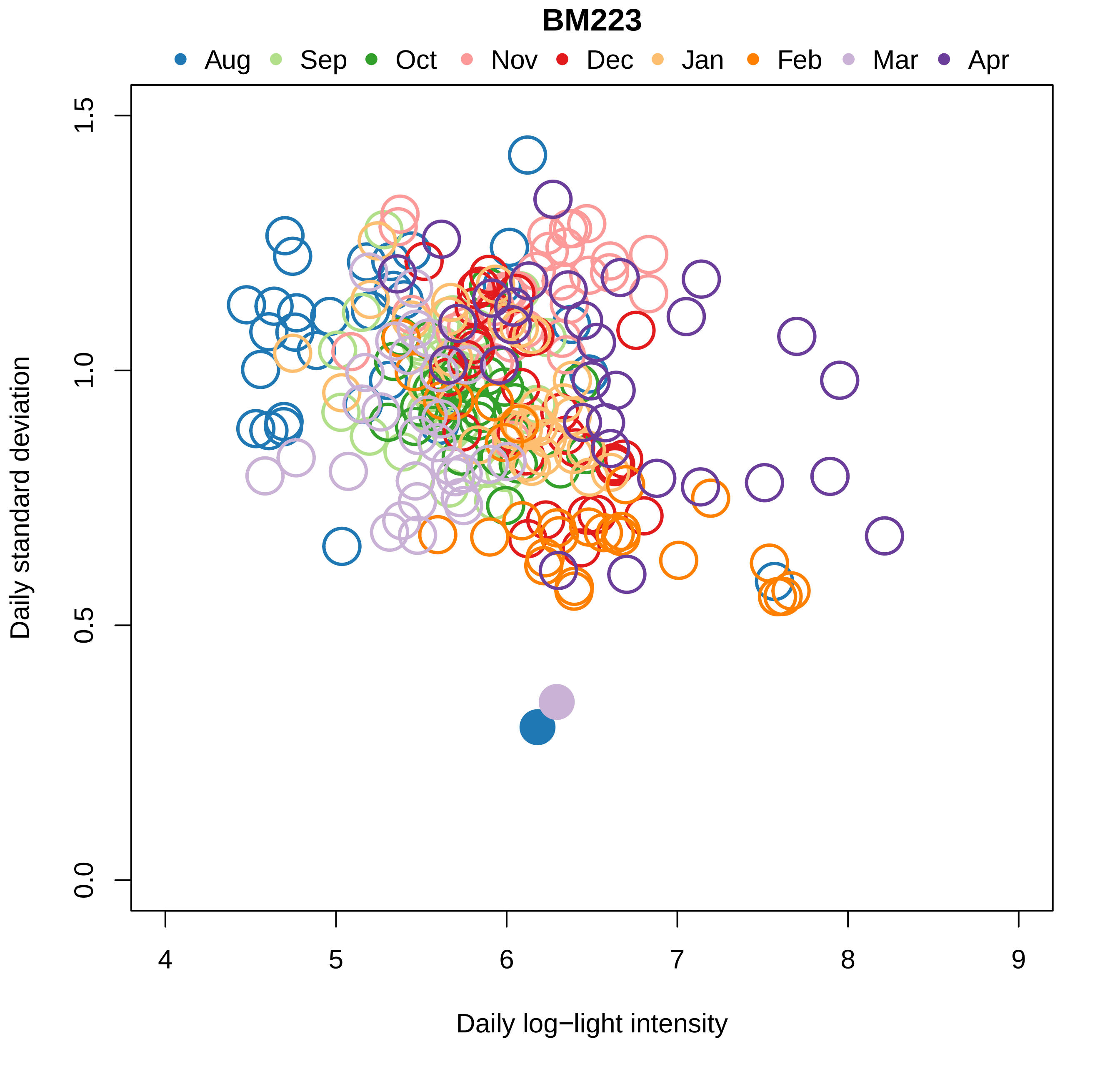
BM223
Aug
Sep
Oct
Nov
Dec
Jan
Feb
Mar
Apr
Daily standard deviation
Daily log−light intensity
0.0
0.5
1.0
1.5
4
5
6
7
8
9

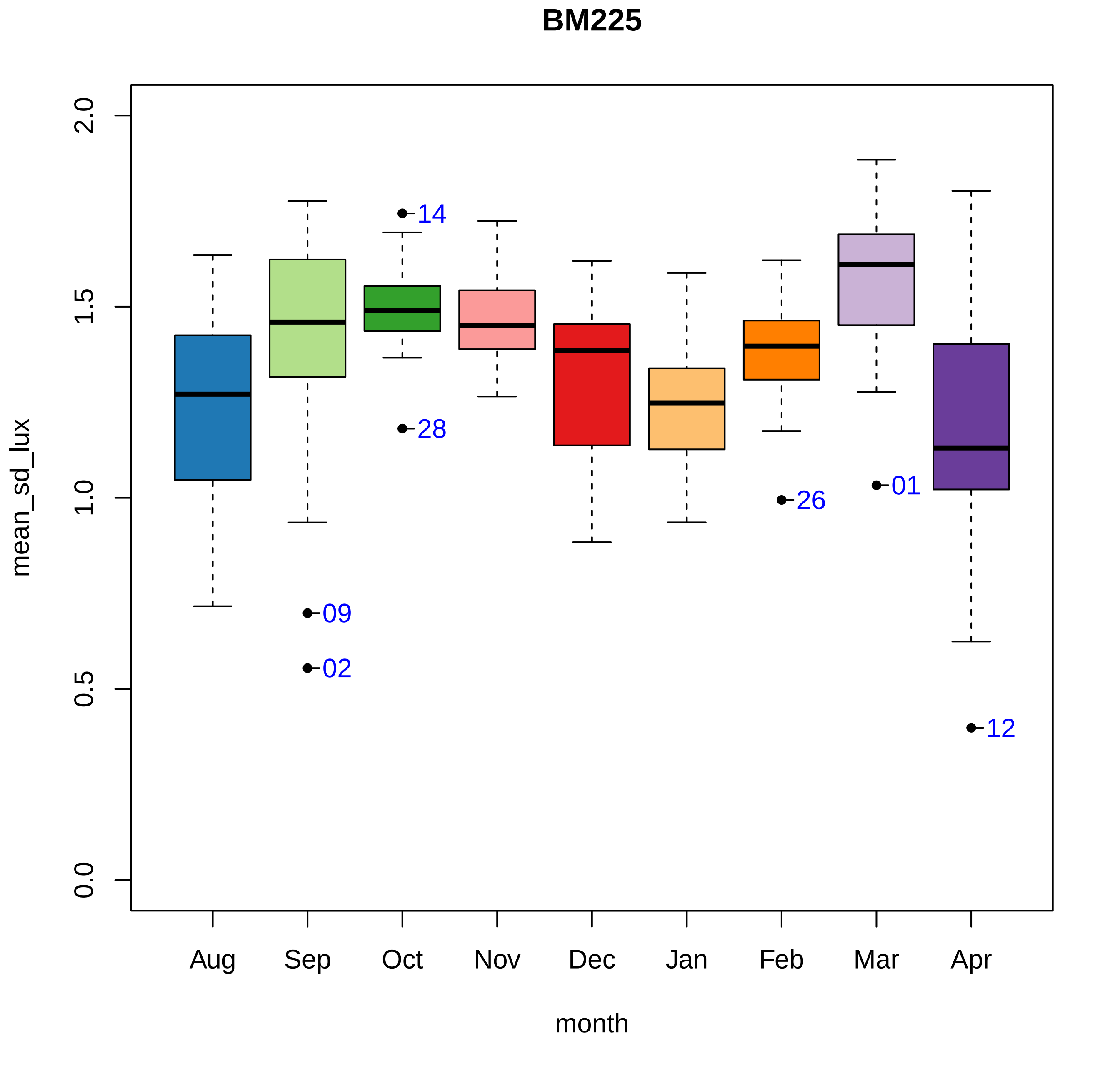
BM225
mean_sd_lux
month
0.0
0.5
1.0
1.5
2.0
Aug
Sep
Oct
Nov
Dec
Jan
Feb
Mar
Apr
14
28
09
02
26
01
12

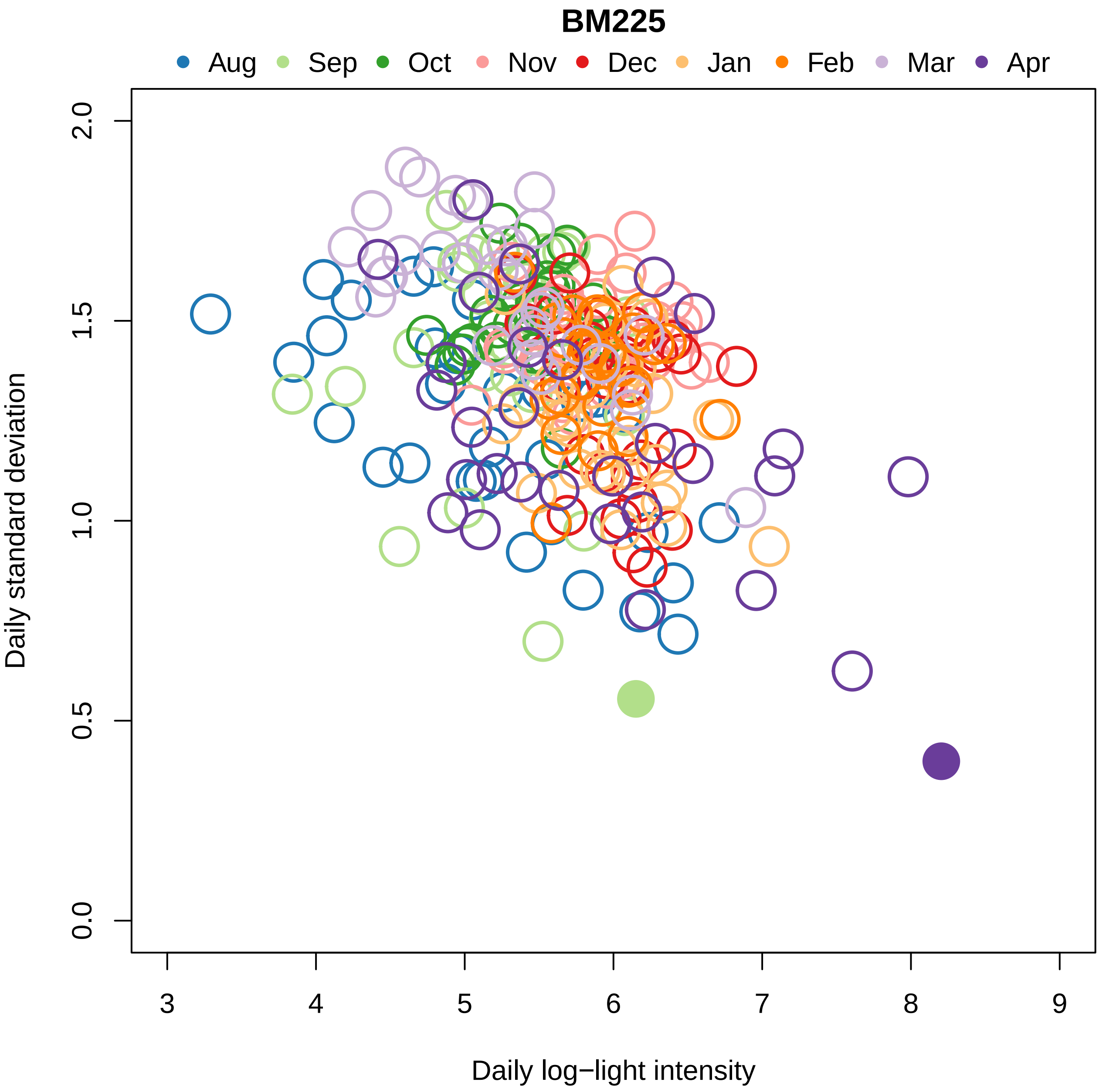
BM225
Aug
Sep
Oct
Nov
Dec
Jan
Feb
Mar
Apr
Daily standard deviation
Daily log−light intensity
0.0
0.5
1.0
1.5
2.0
3
4
5
6
7
8
9

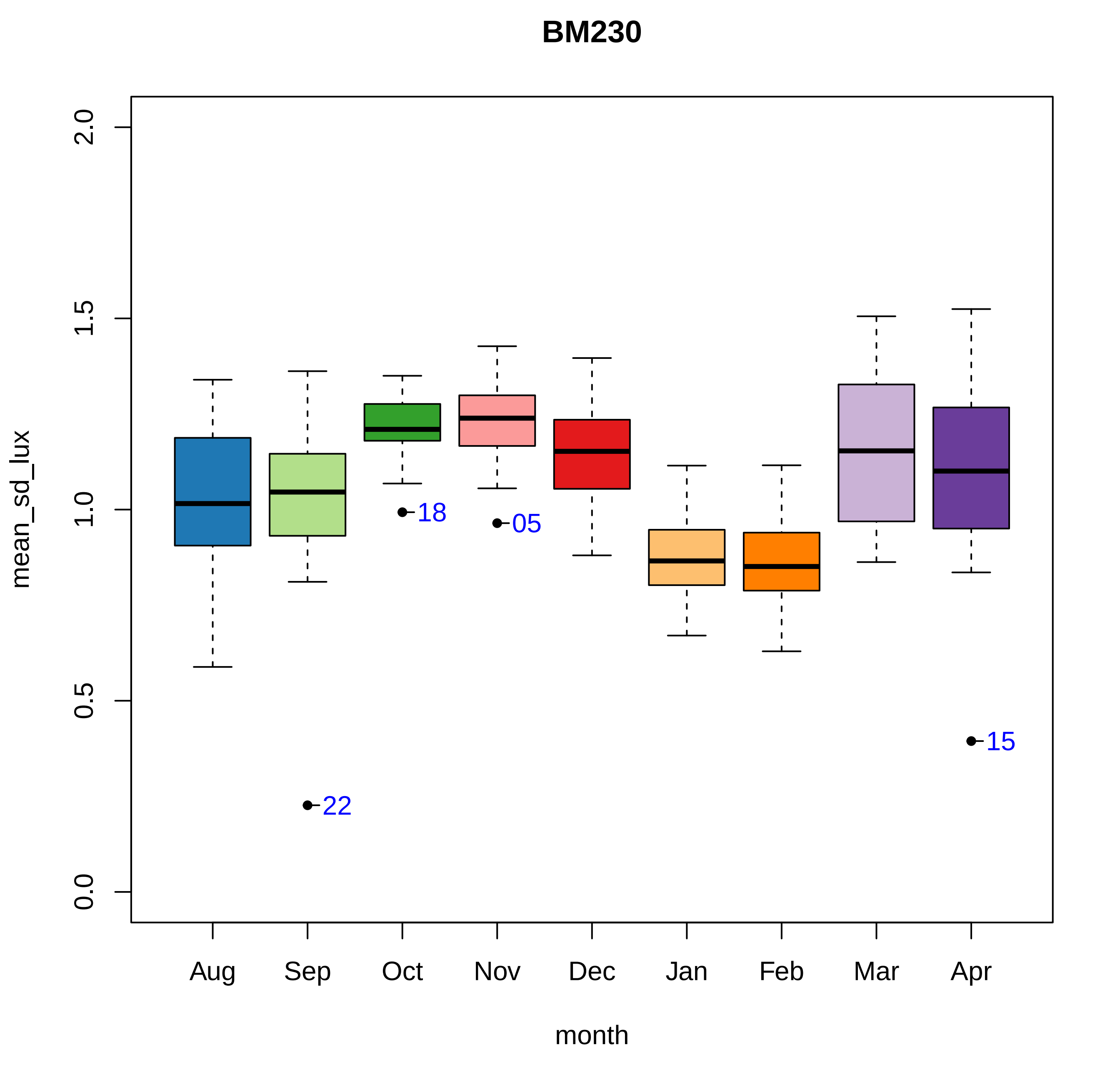
BM230
mean_sd_lux
0.0
0.5
1.0
1.5
2.0
22
18
05
15
Aug
Sep
Oct
Nov
Dec
Jan
Feb
Mar
Apr
month

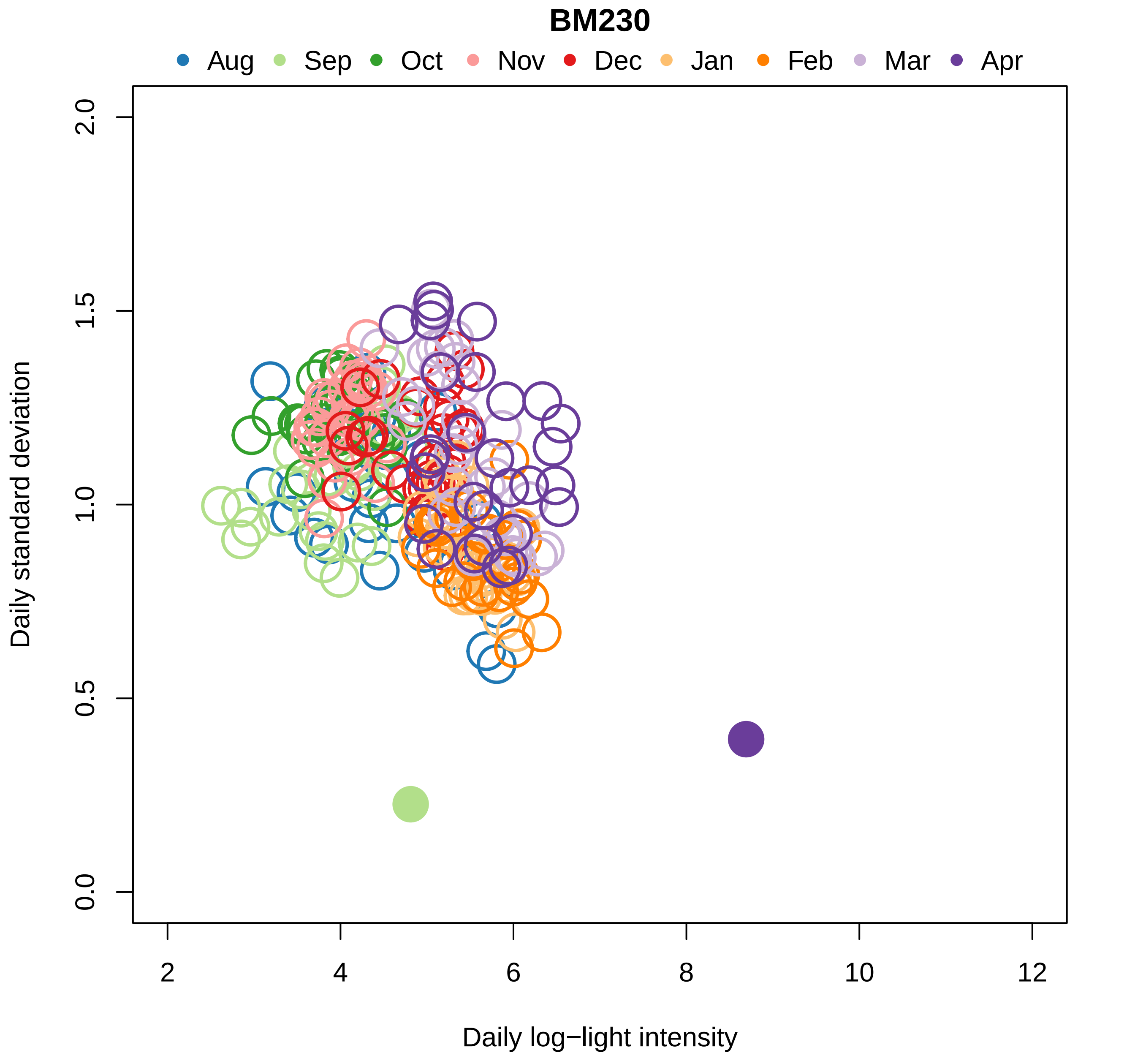
BM230
Aug
Sep
Oct
Nov
Dec
Jan
Feb
Mar
Apr
Daily standard deviation
0.0
0.5
1.0
1.5
2.0
2
4
6
8
10
12
Daily log−light intensity

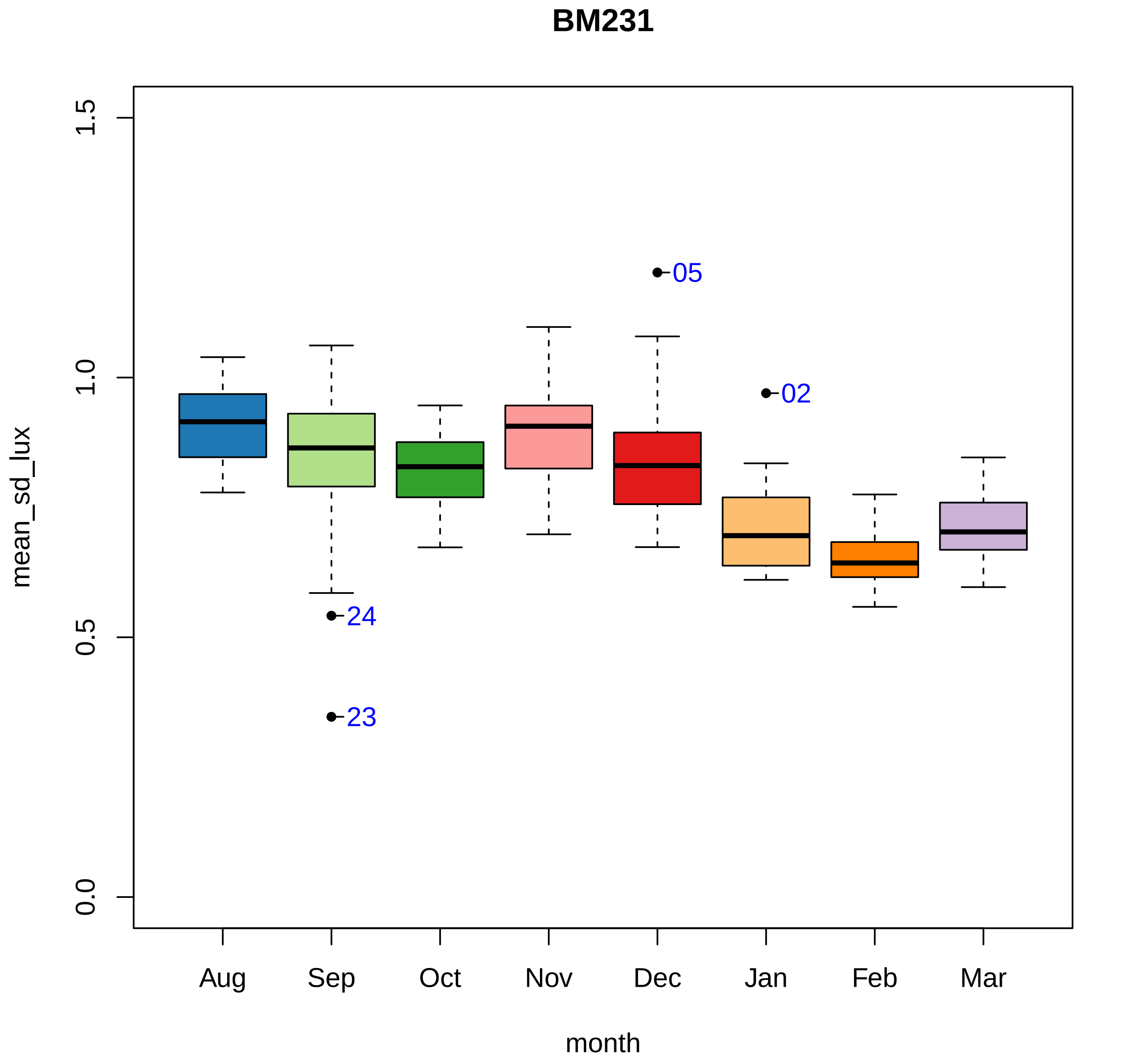

BM231
mean_sd_lux
0.0
0.5
1.0
1.5
05
02
24
23
Aug
Sep
Oct
Nov
Dec
Jan
Feb
Mar
month

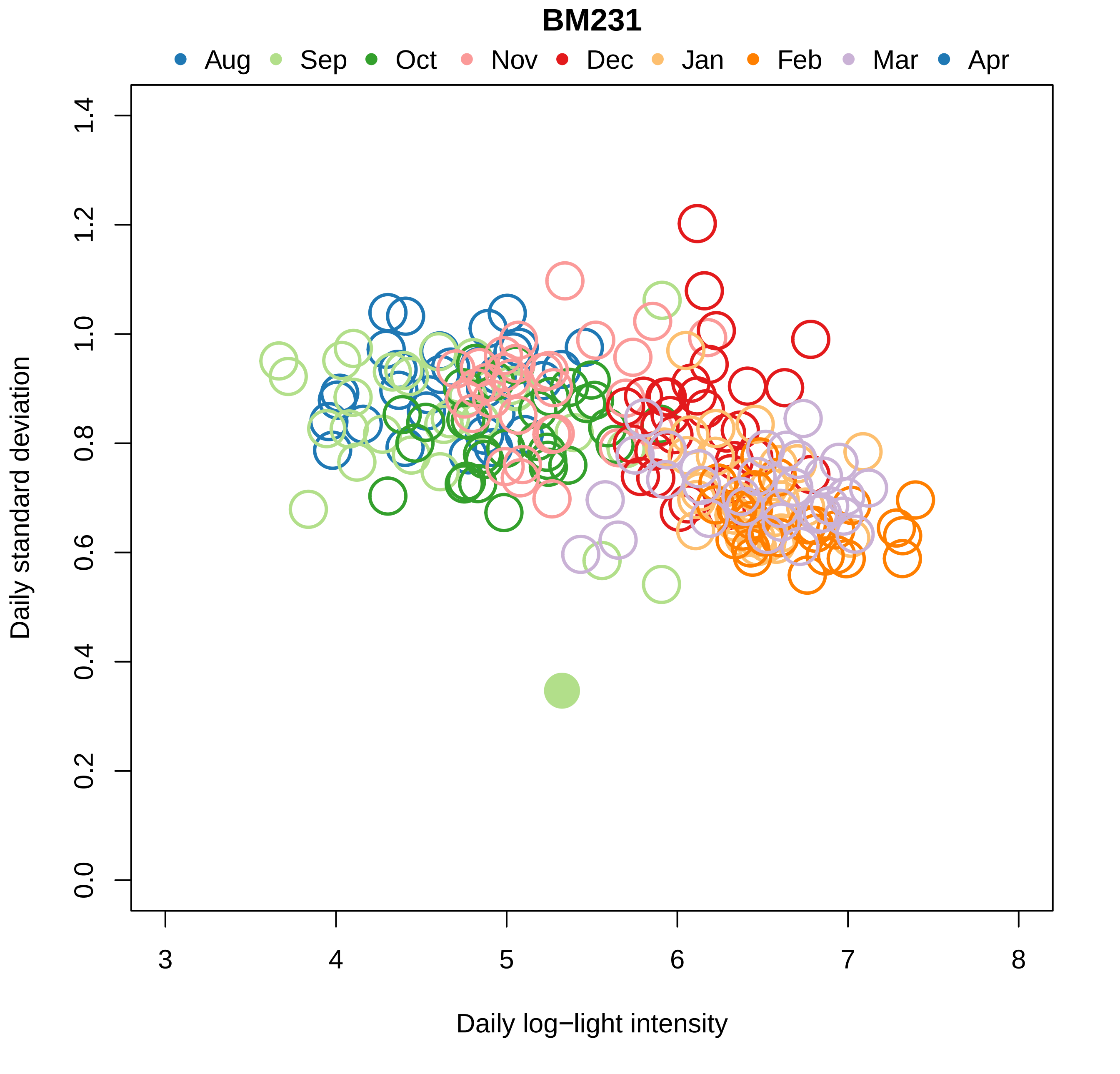
BM231
Aug
Sep
Oct
Nov
Dec
Jan
Feb
Mar
Apr
Daily standard deviation
0.0
0.2
0.4
0.6
0.8
1.0
1.2
1.4
3
4
5
6
7
8
Daily log−light intensity

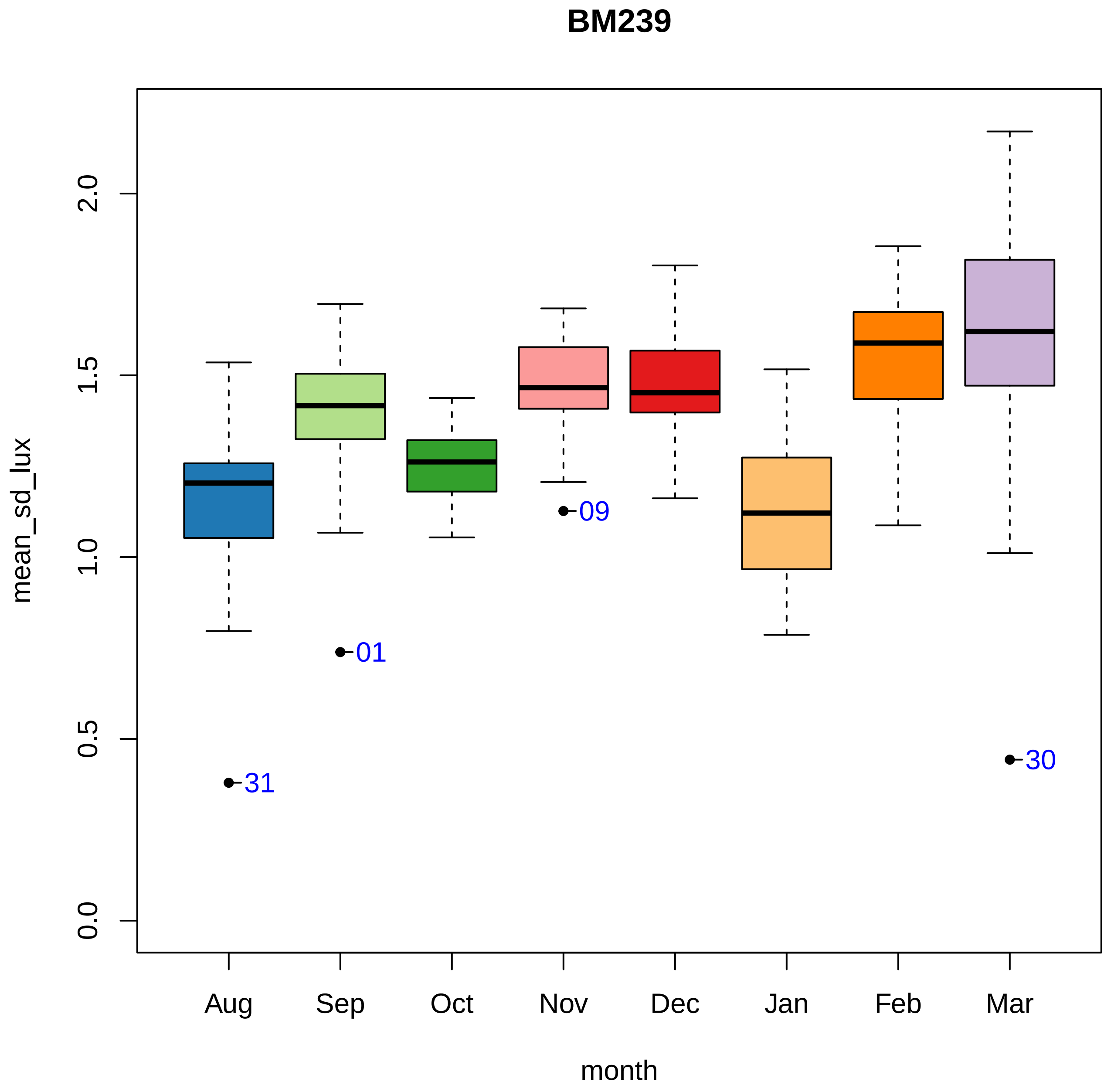
BM239
mean_sd_lux
0.0
0.5
1.0
1.5
2.0
31
01
09
30
Aug
Sep
Oct
Nov
Dec
Jan
Feb
Mar
month

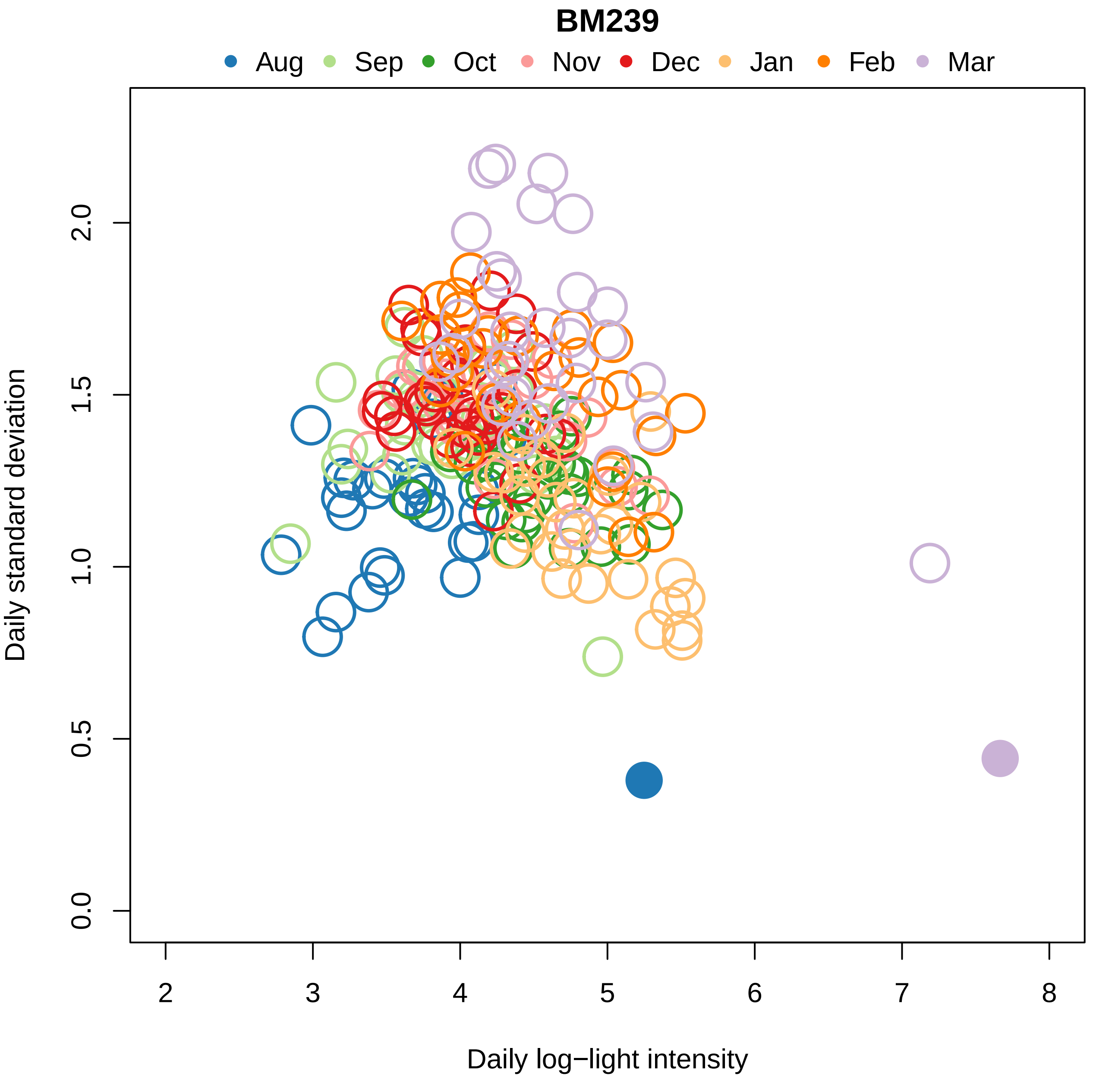

BM239
Aug
Sep
Oct
Nov
Dec
Jan
Feb
Mar
Daily standard deviation
0.0
0.5
1.0
1.5
2.0
2
3
4
5
6
7
8
Daily log−light intensity

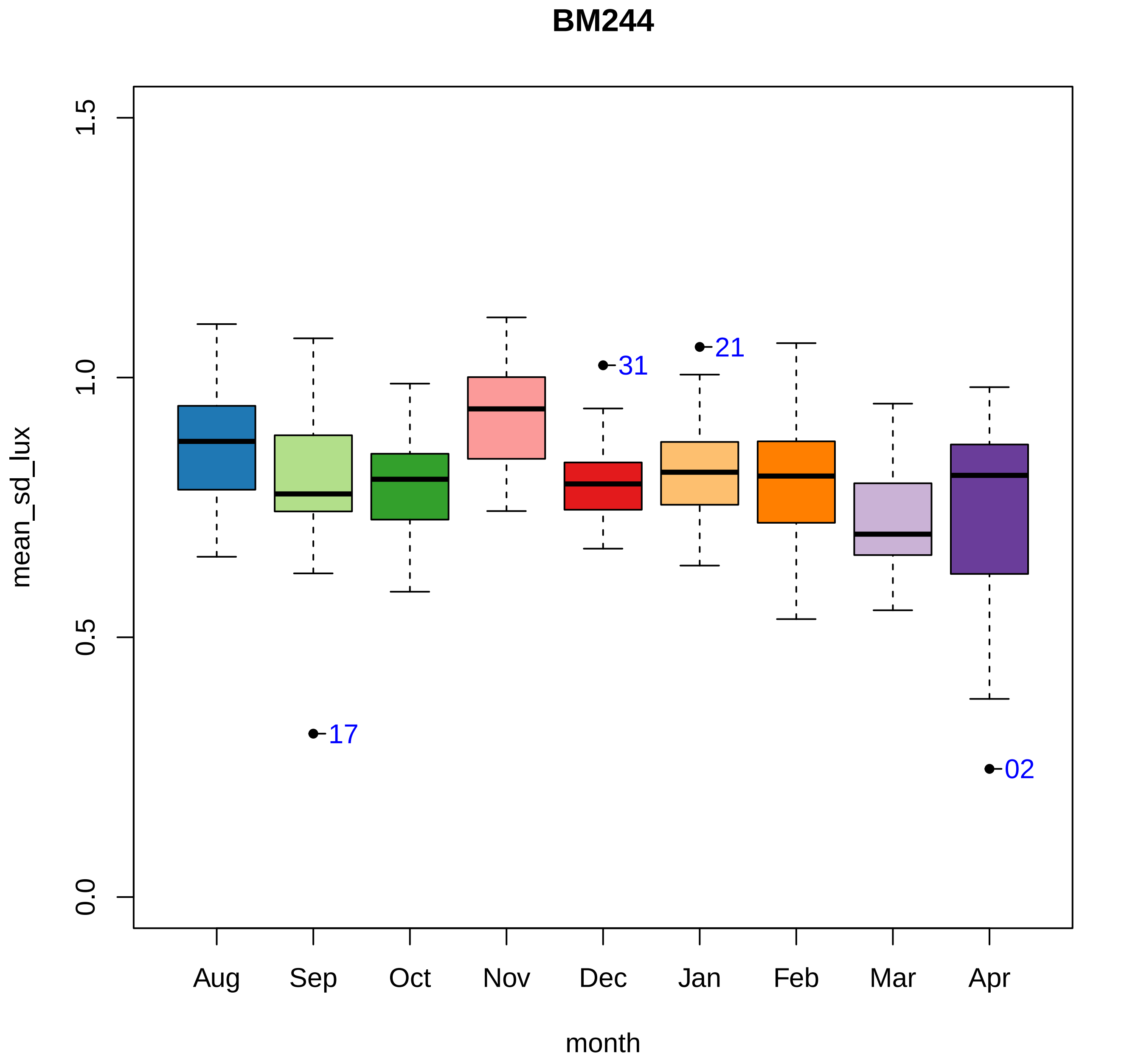
BM244
mean_sd_lux
0.0
0.5
1.0
1.5
month
Aug
Sep
Oct
Nov
Dec
Jan
Feb
Mar
Apr
17
31
21
02

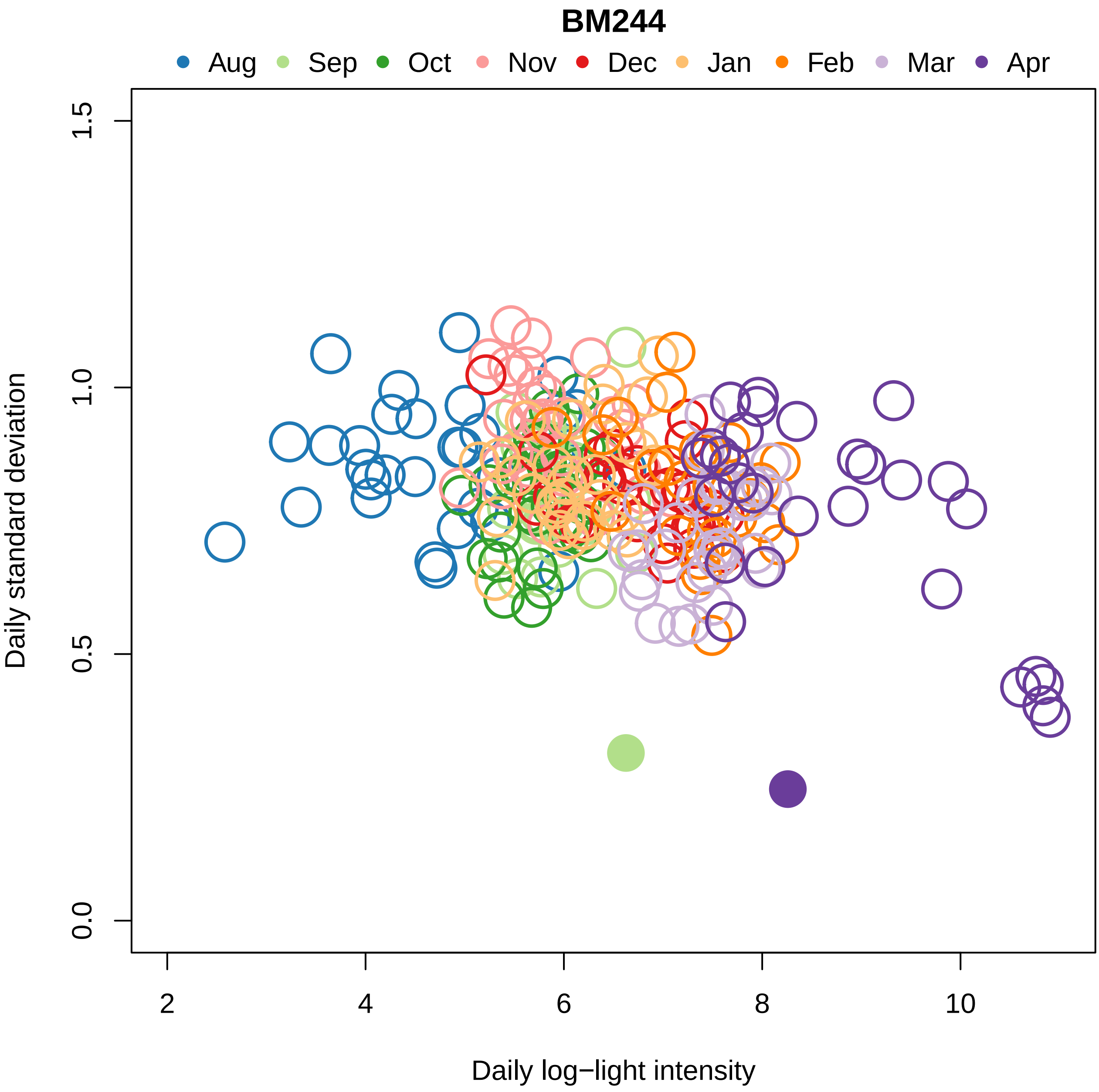
BM244
Aug
Sep
Oct
Nov
Dec
Jan
Feb
Mar
Apr
Daily standard deviation
0.0
0.5
1.0
1.5
2
4
6
8
10
Daily log−light intensity

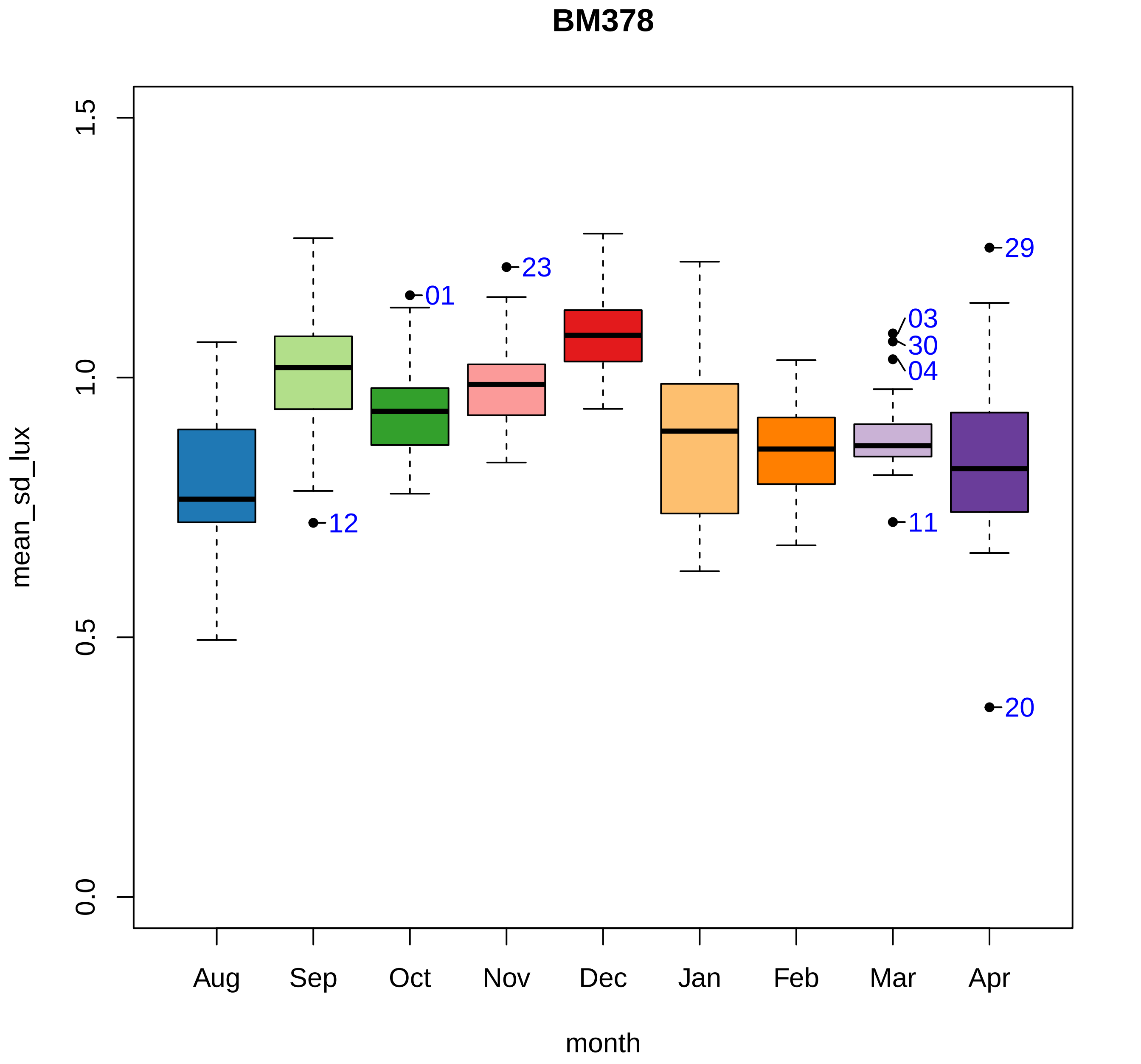
BM378
mean_sd_lux
0.0
0.5
1.0
1.5
month
Aug
Sep
Oct
Nov
Dec
Jan
Feb
Mar
Apr
12
01
23
03
30
04
11
29
20

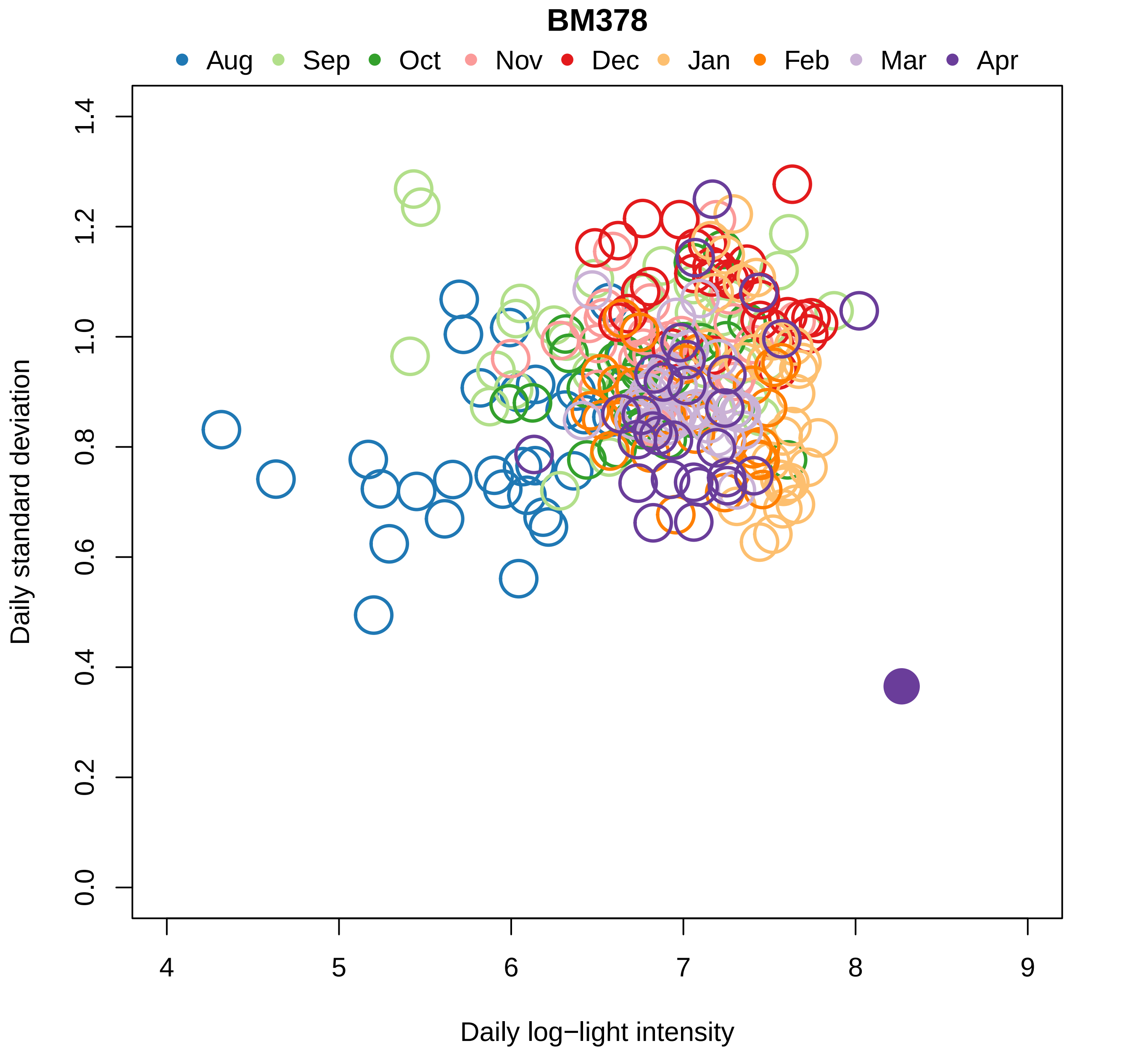
BM378
Aug
Sep
Oct
Nov
Dec
Jan
Feb
Mar
Apr
Daily standard deviation
0.0
0.2
0.4
0.6
0.8
1.0
1.2
1.4
4
5
6
7
8
9
Daily log−light intensity

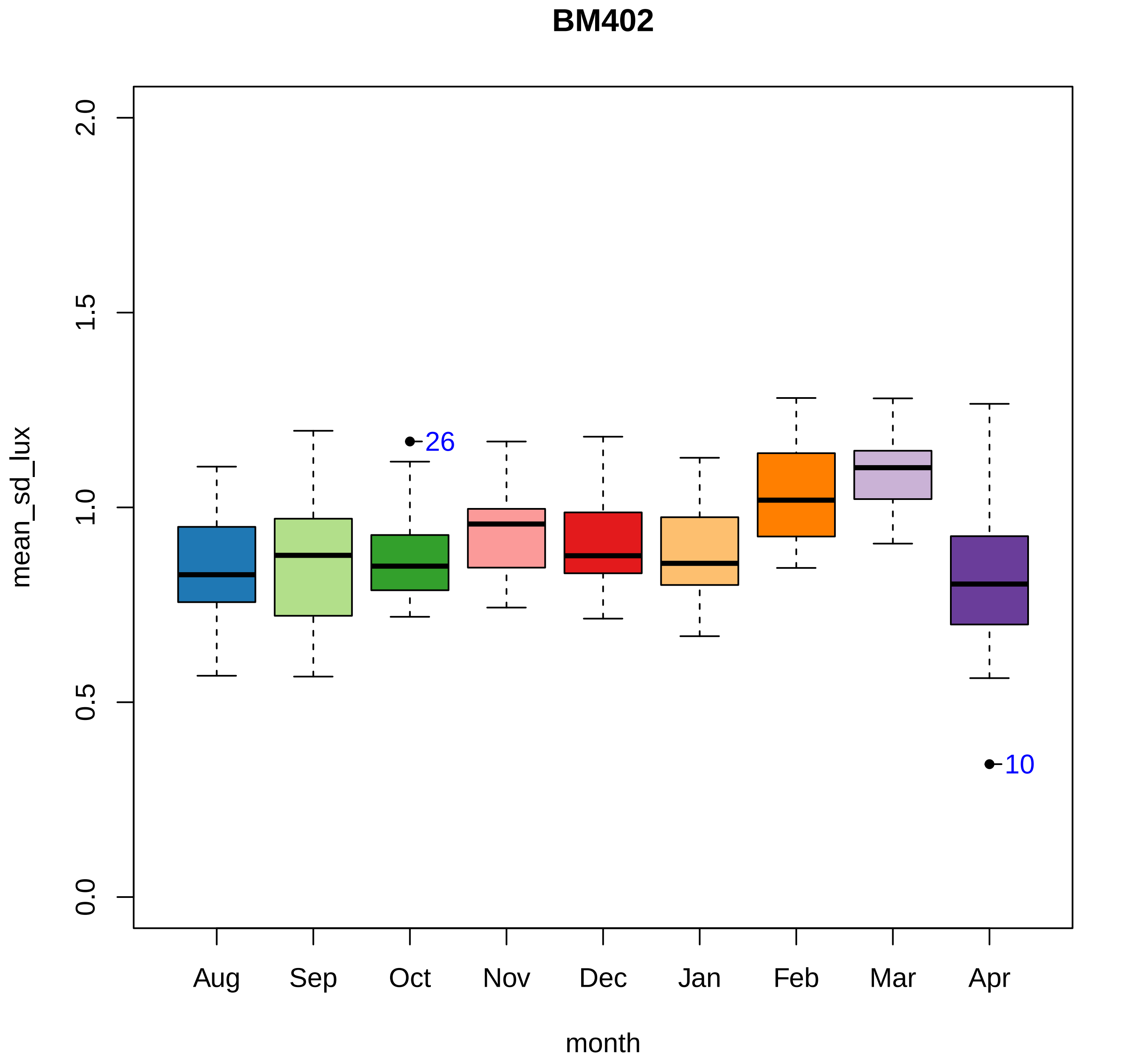

BM402
mean_sd_lux
month
Aug
Sep
Oct
Nov
Dec
Jan
Feb
Mar
Apr
0.0
0.5
1.0
1.5
2.0
26
10

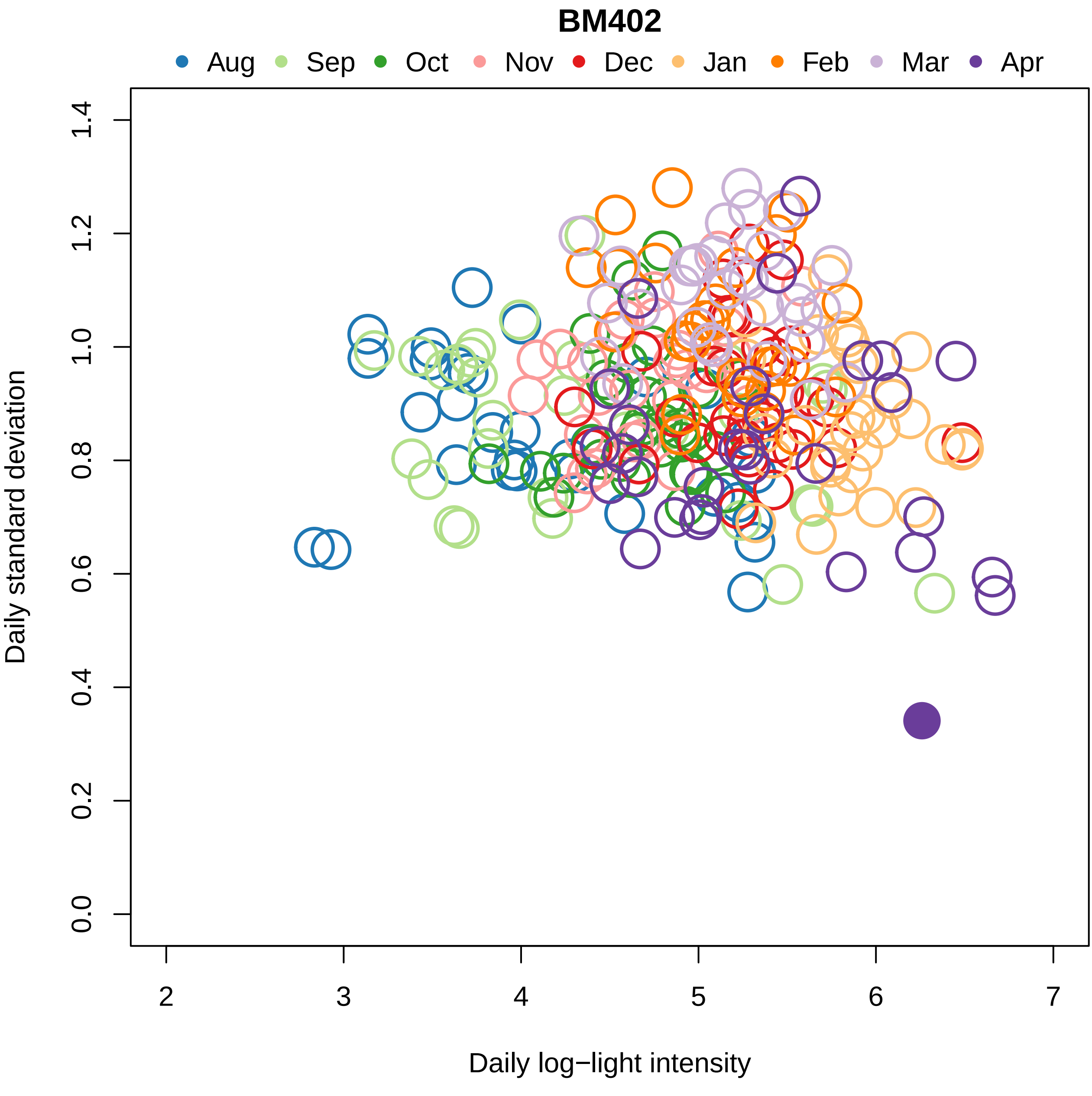

BM402
Aug
Sep
Oct
Nov
Dec
Jan
Feb
Mar
Apr
Daily standard deviation
0.0
0.2
0.4
0.6
0.8
1.0
1.2
1.4
2
3
4
5
6
7
Daily log−light intensity

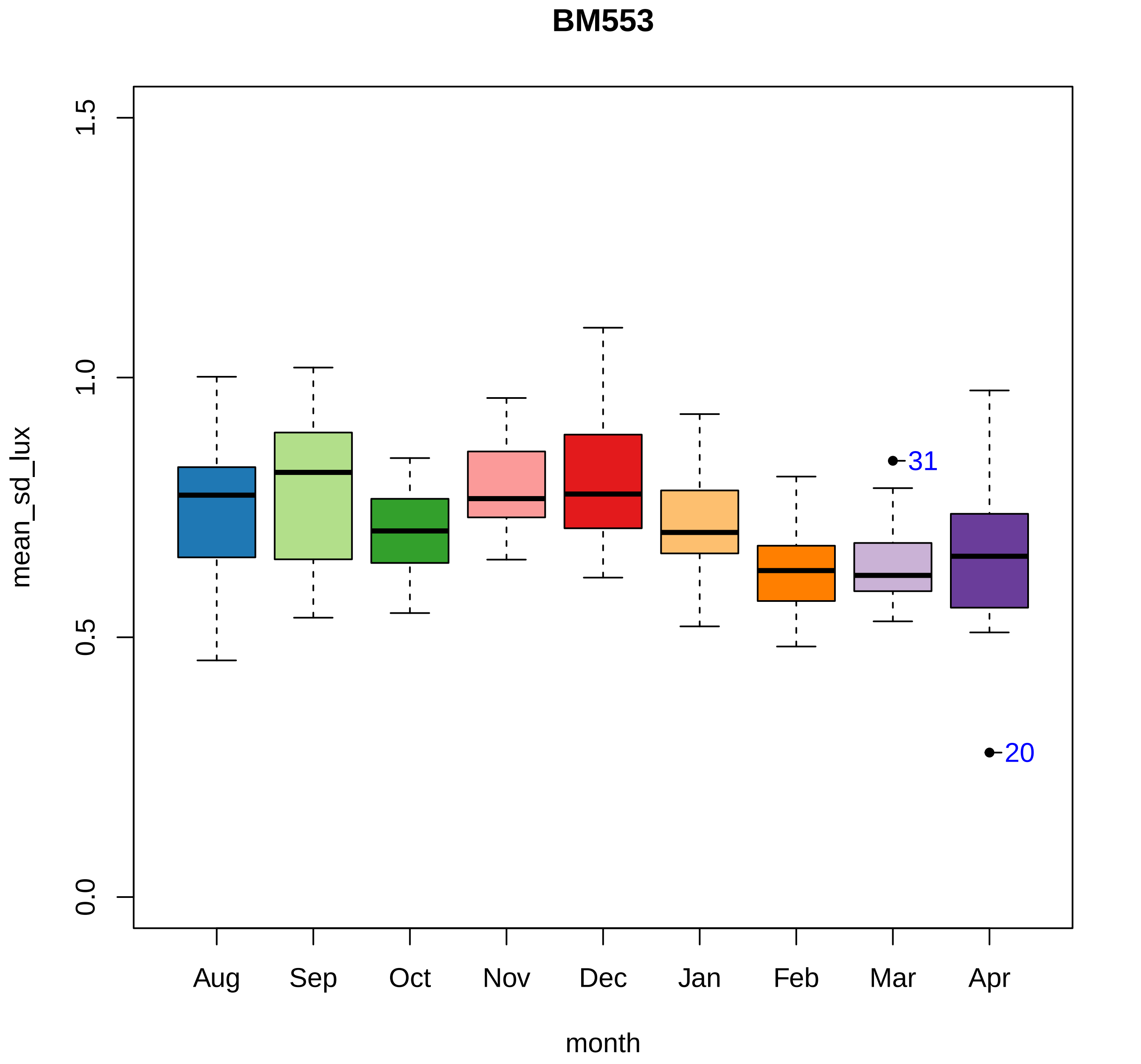
BM553
mean_sd_lux
0.0
0.5
1.0
1.5
month
Aug
Sep
Oct
Nov
Dec
Jan
Feb
Mar
Apr
31
20

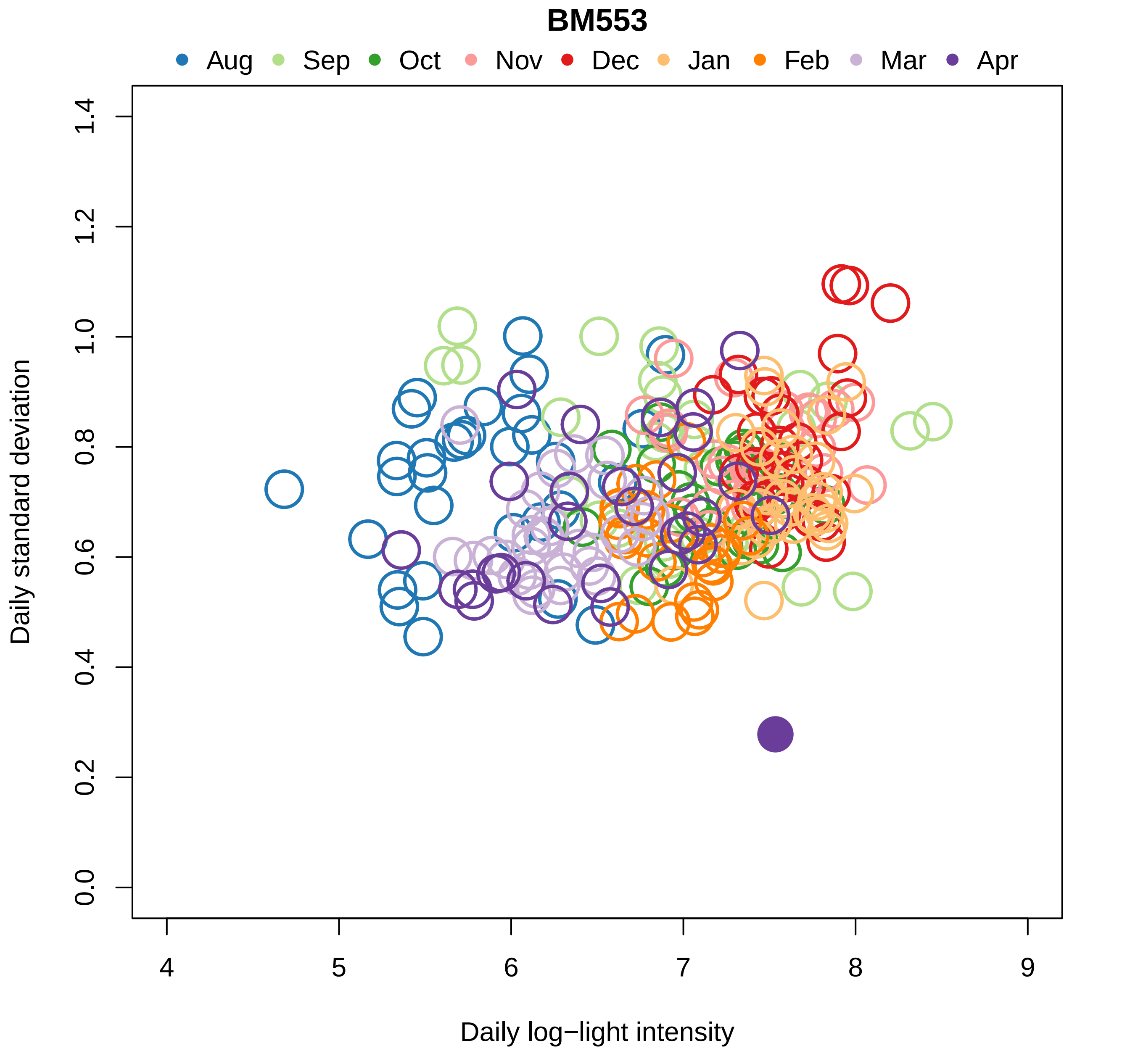
BM553
Aug
Sep
Oct
Nov
Dec
Jan
Feb
Mar
Apr
Daily standard deviation
Daily log−light intensity
0.0
0.2
0.4
0.6
0.8
1.0
1.2
1.4
4
5
6
7
8
9

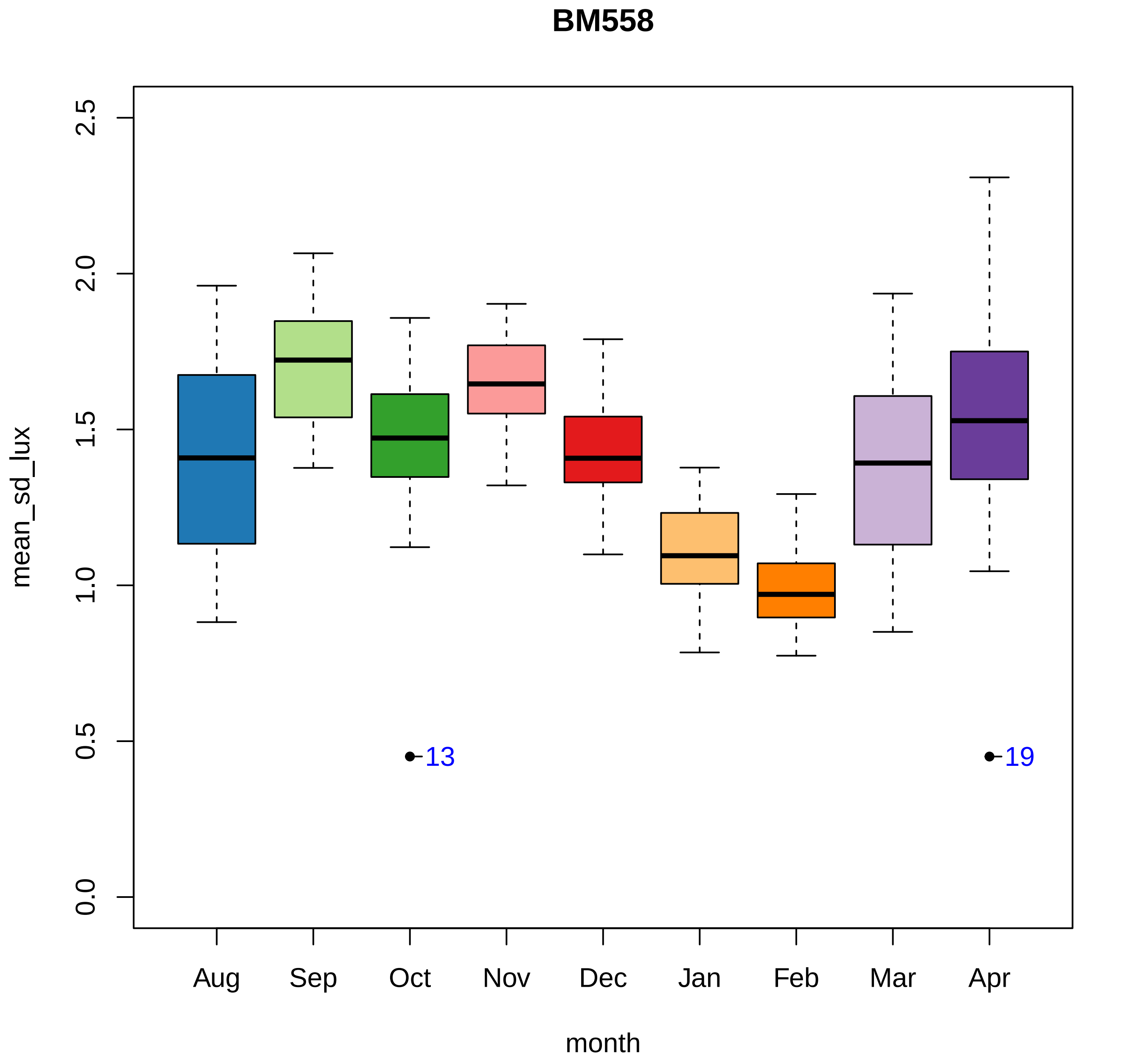

BM558
mean_sd_lux
0.0
0.5
1.0
1.5
2.0
2.5
13
19
Aug
Sep
Oct
Nov
Dec
Jan
Feb
Mar
Apr
month

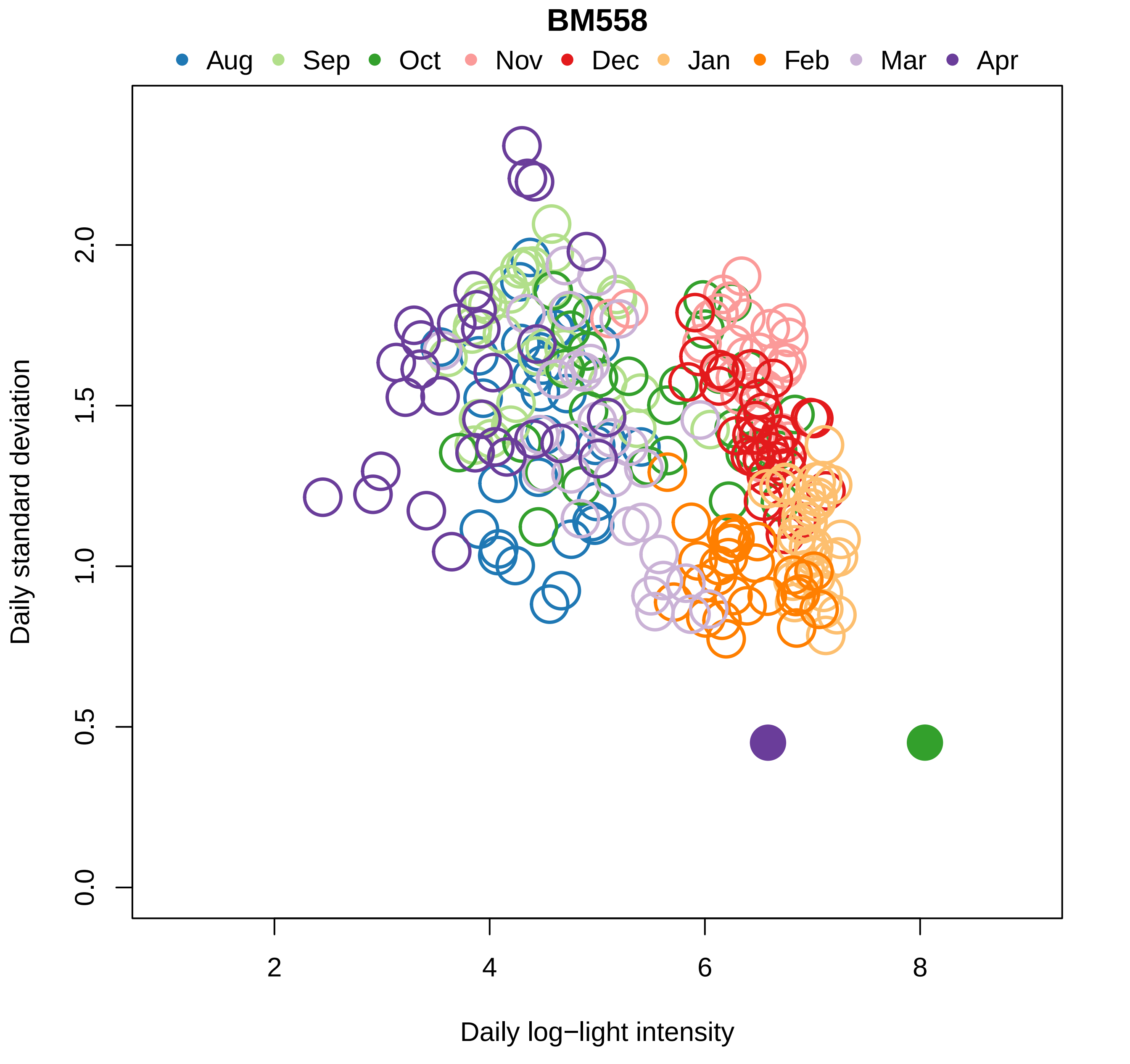
BM558
Aug
Sep
Oct
Nov
Dec
Jan
Feb
Mar
Apr
Daily standard deviation
0.0
0.5
1.0
1.5
2.0
2
4
6
8
Daily log−light intensity

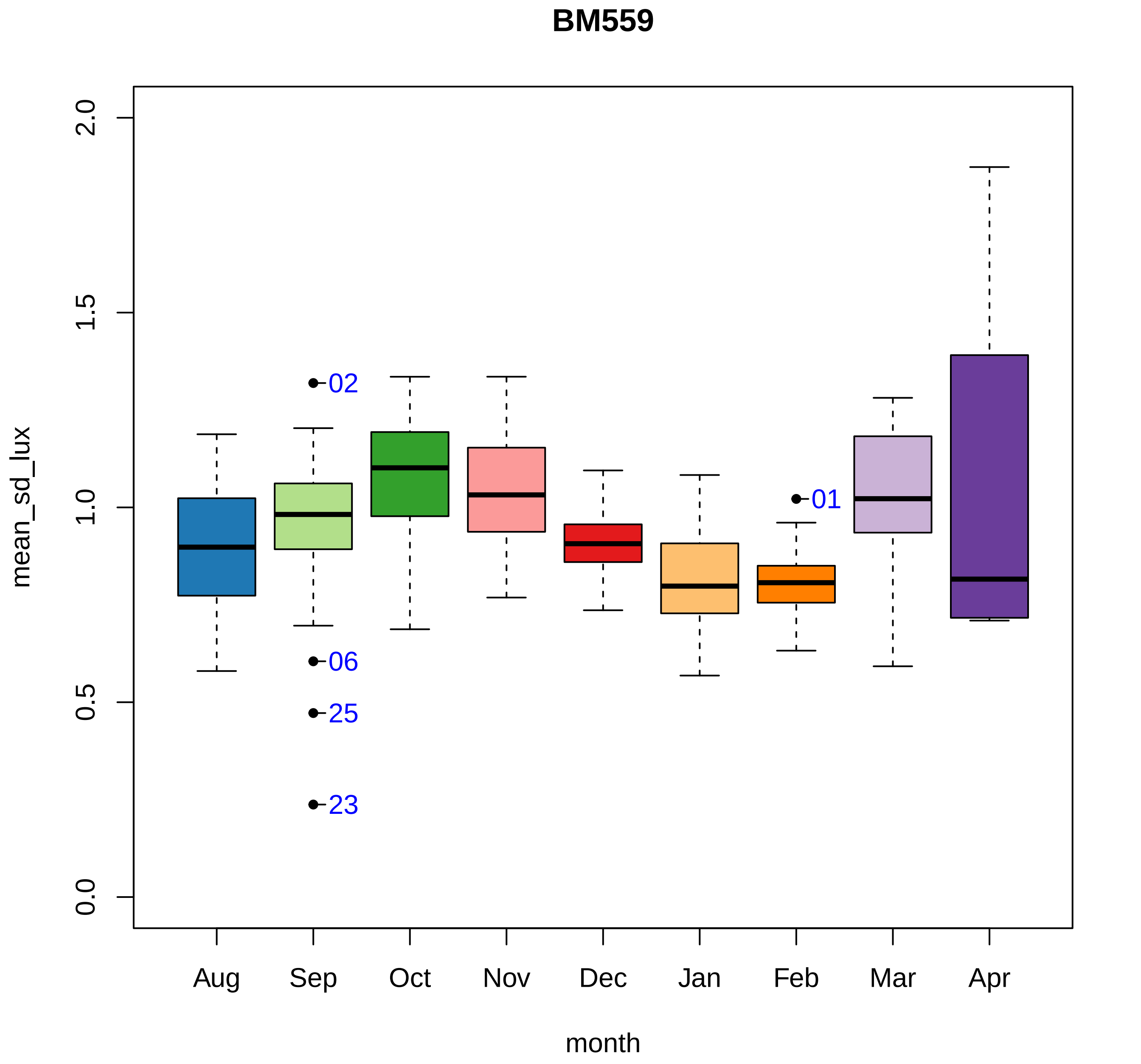
BM559
mean_sd_lux
2.0
1.5
1.0
0.5
0.0
02
06
25
23
01
Aug
Sep
Oct
Nov
Dec
Jan
Feb
Mar
Apr
month

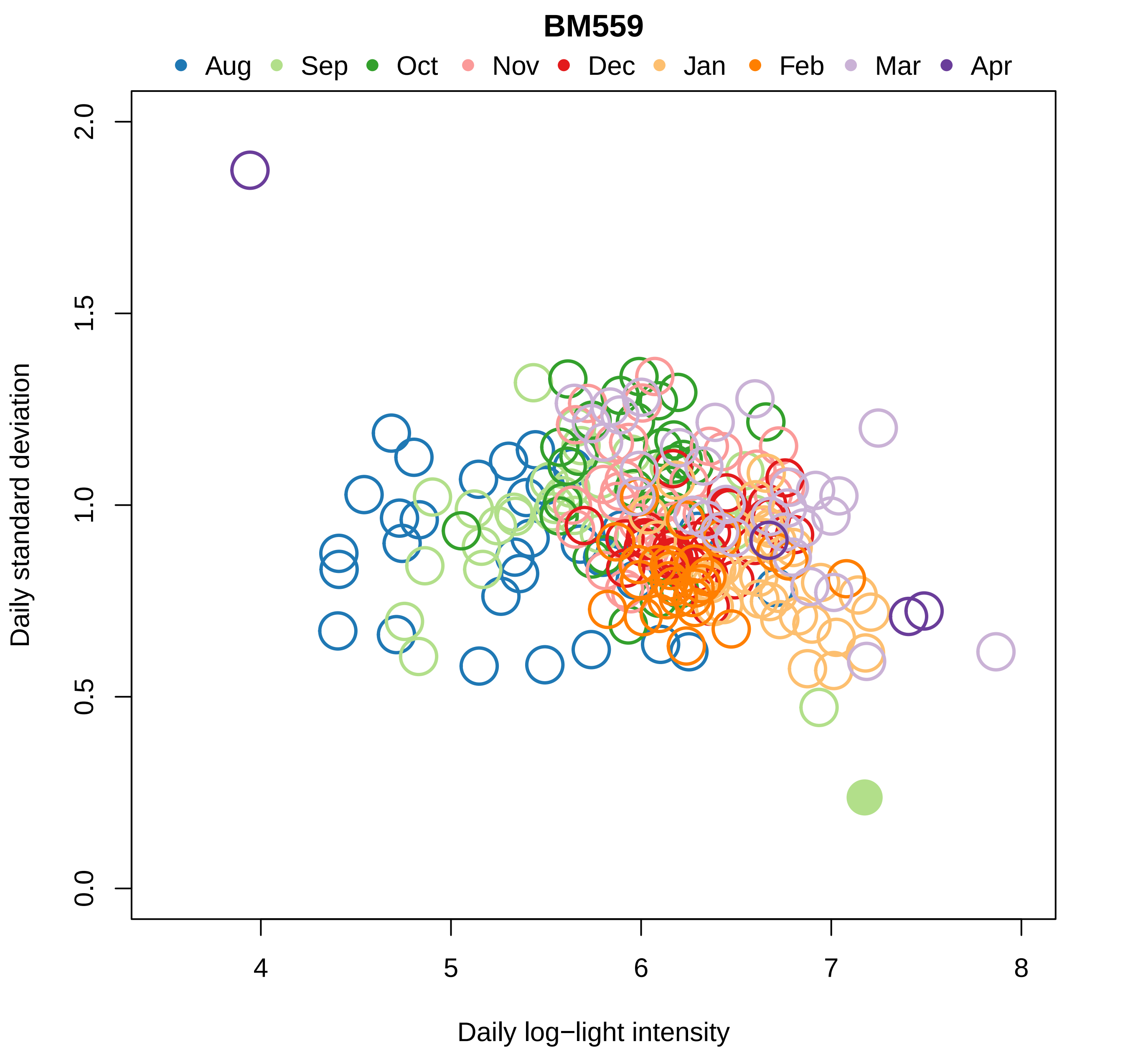

BM559
Aug
Sep
Oct
Nov
Dec
Jan
Feb
Mar
Apr
Daily standard deviation
0.0
0.5
1.0
1.5
2.0
4
5
6
7
8
Daily log−light intensity

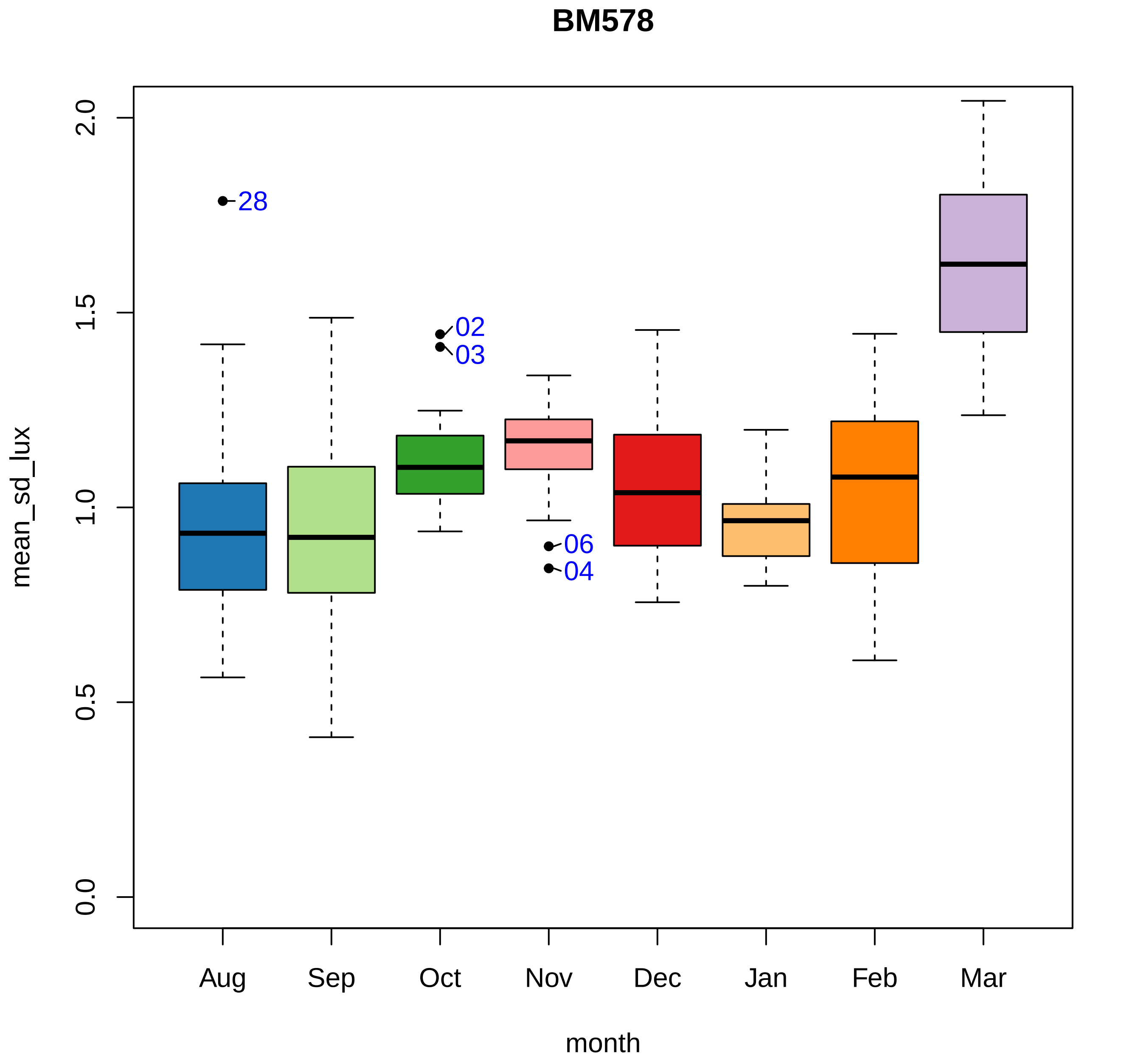
BM578
mean_sd_lux
month
0.0
0.5
1.0
1.5
2.0
Aug
Sep
Oct
Nov
Dec
Jan
Feb
Mar
28
02
03
06
04

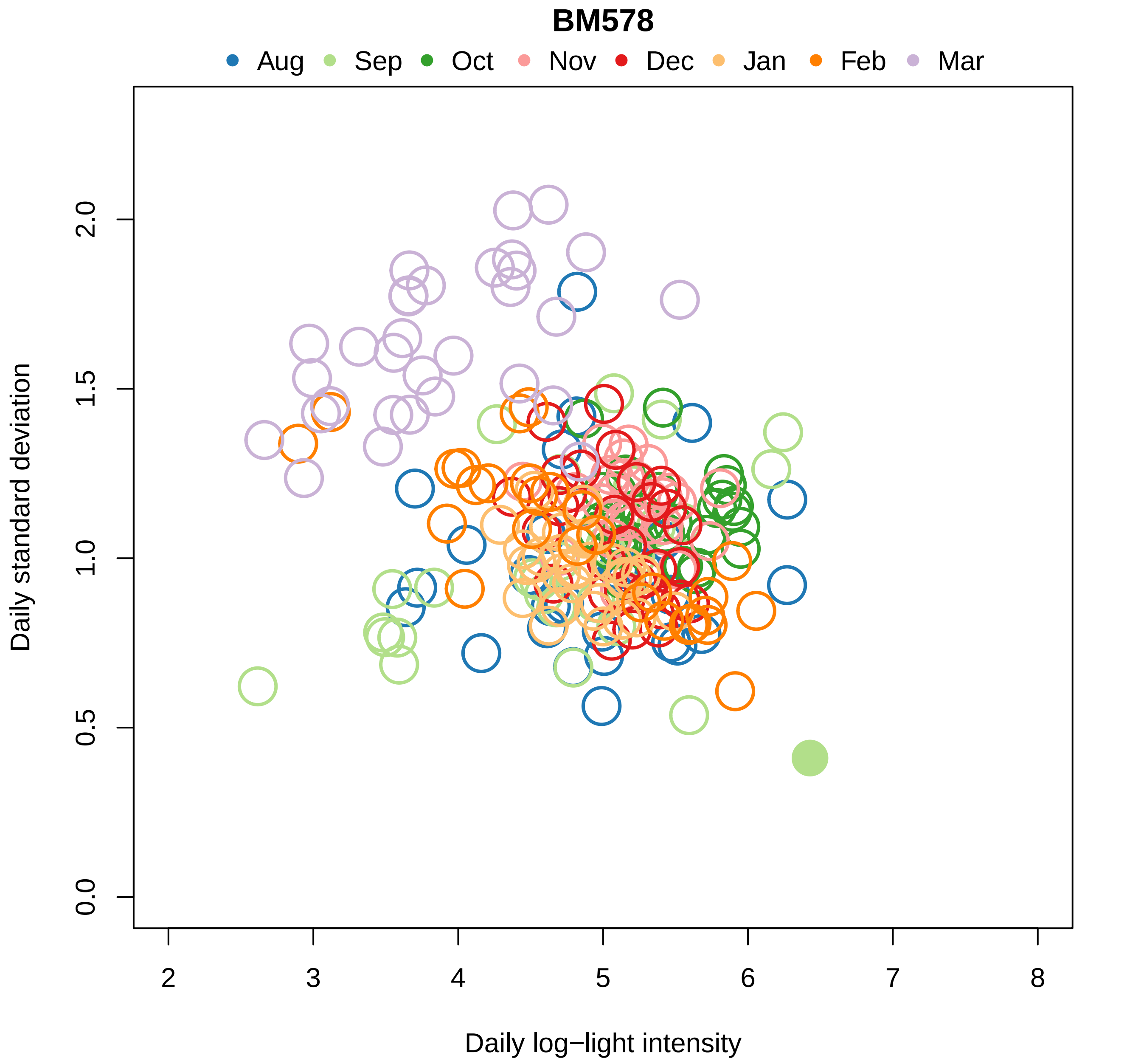

BM578
Aug
Sep
Oct
Nov
Dec
Jan
Feb
Mar
Daily standard deviation
0.0
0.5
1.0
1.5
2.0
2
3
4
5
6
7
8
Daily log−light intensity

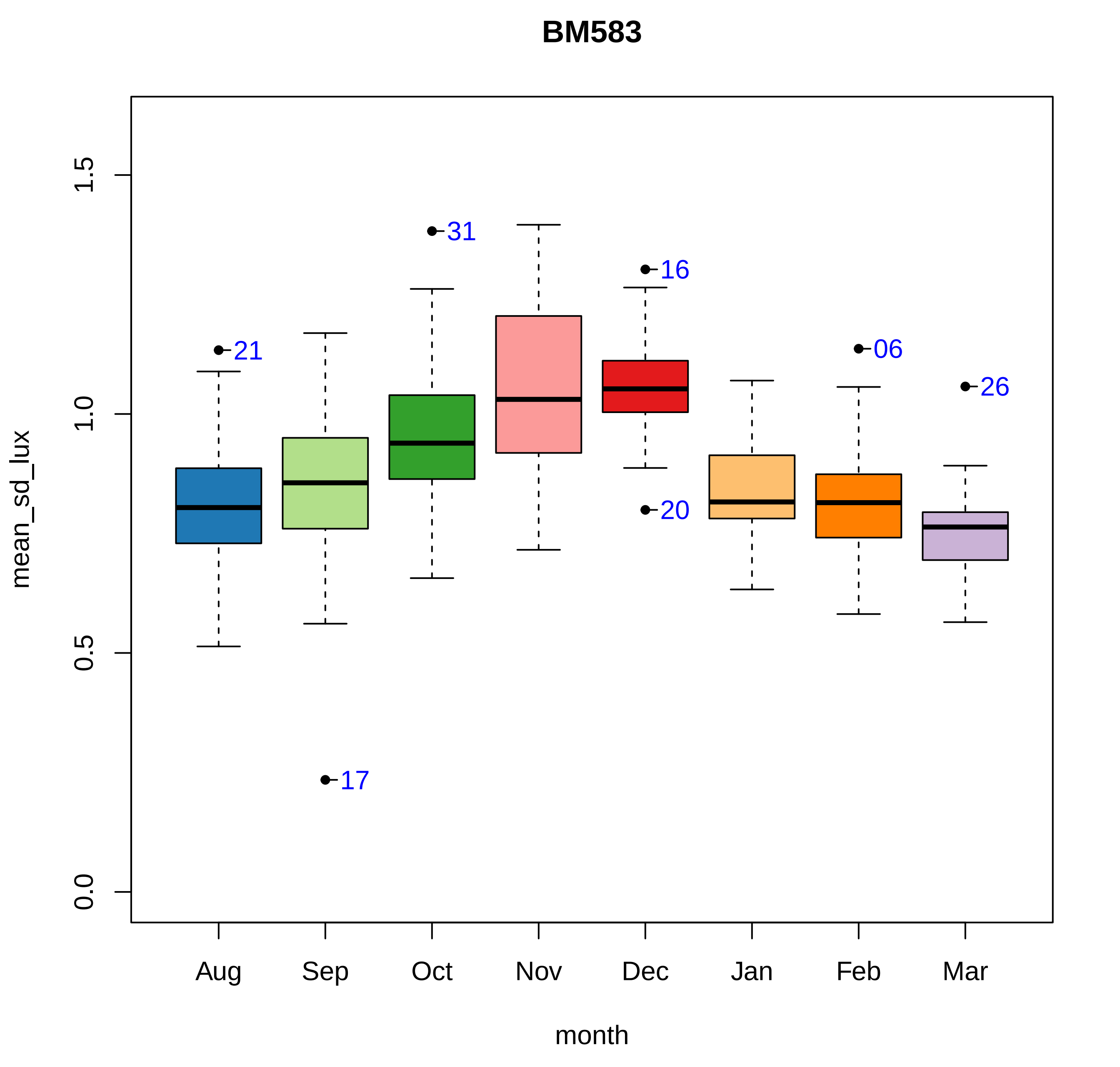

BM583
mean_sd_lux
0.0
0.5
1.0
1.5
month
Aug
Sep
Oct
Nov
Dec
Jan
Feb
Mar
21
17
31
16
20
06
26

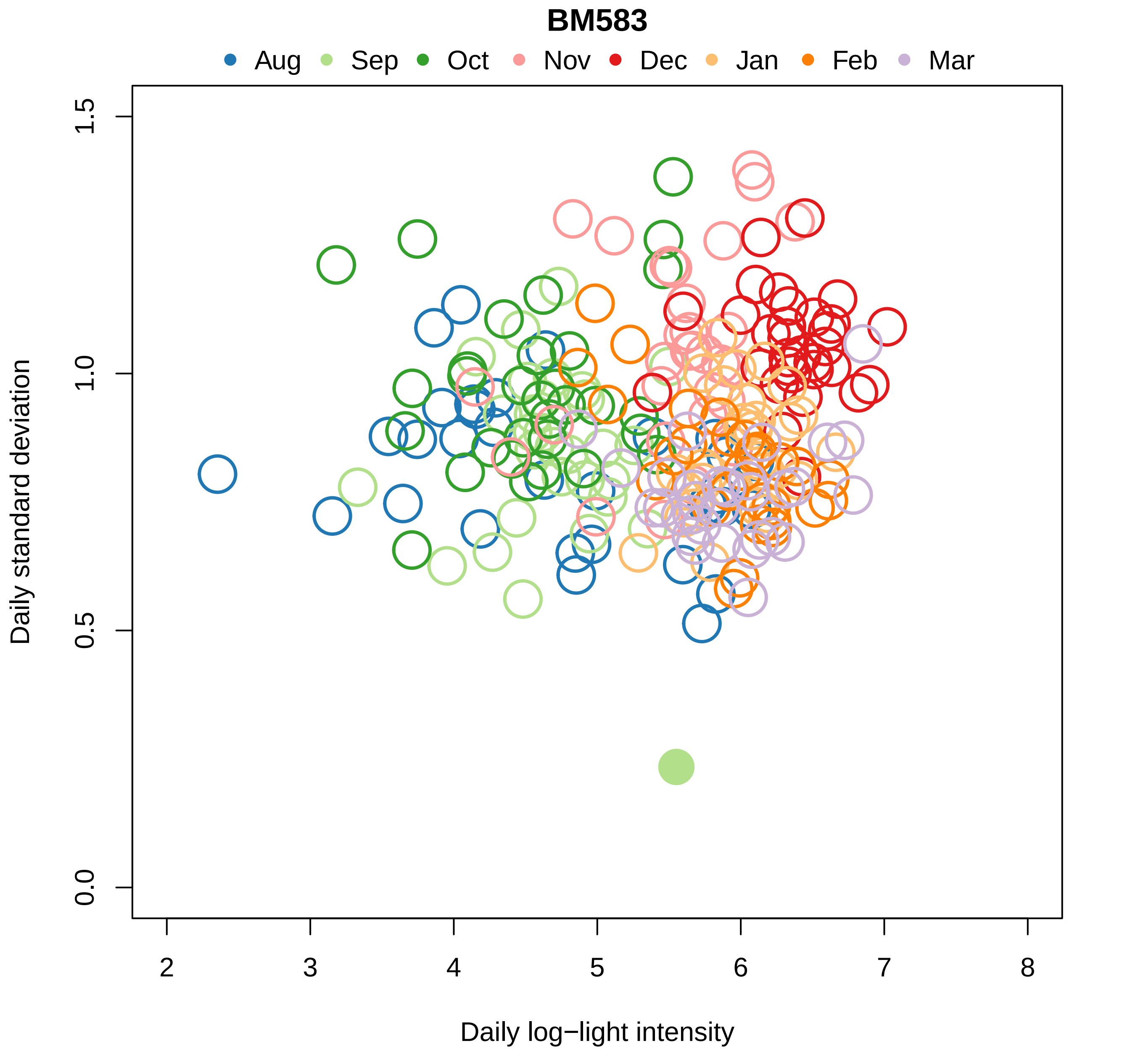

BM583
Aug
Sep
Oct
Nov
Dec
Jan
Feb
Mar
Daily standard deviation
0.0
0.5
1.0
1.5
2
3
4
5
6
7
8
Daily log−light intensity

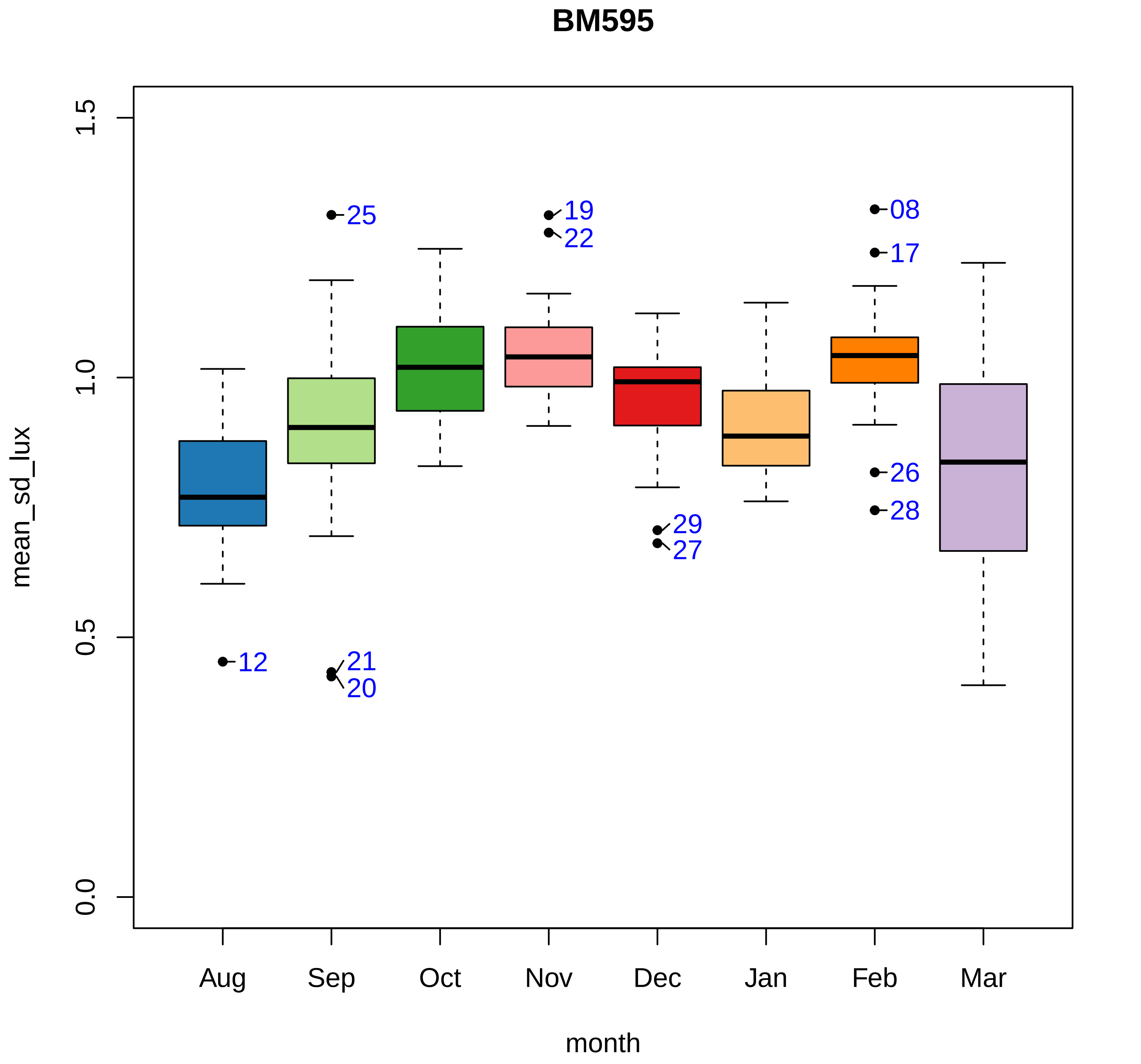

BM595
mean_sd_lux
month
0.0
0.5
1.0
1.5
Aug
Sep
Oct
Nov
Dec
Jan
Feb
Mar
25
19
22
08
17
26
28
29
27
12
21
20

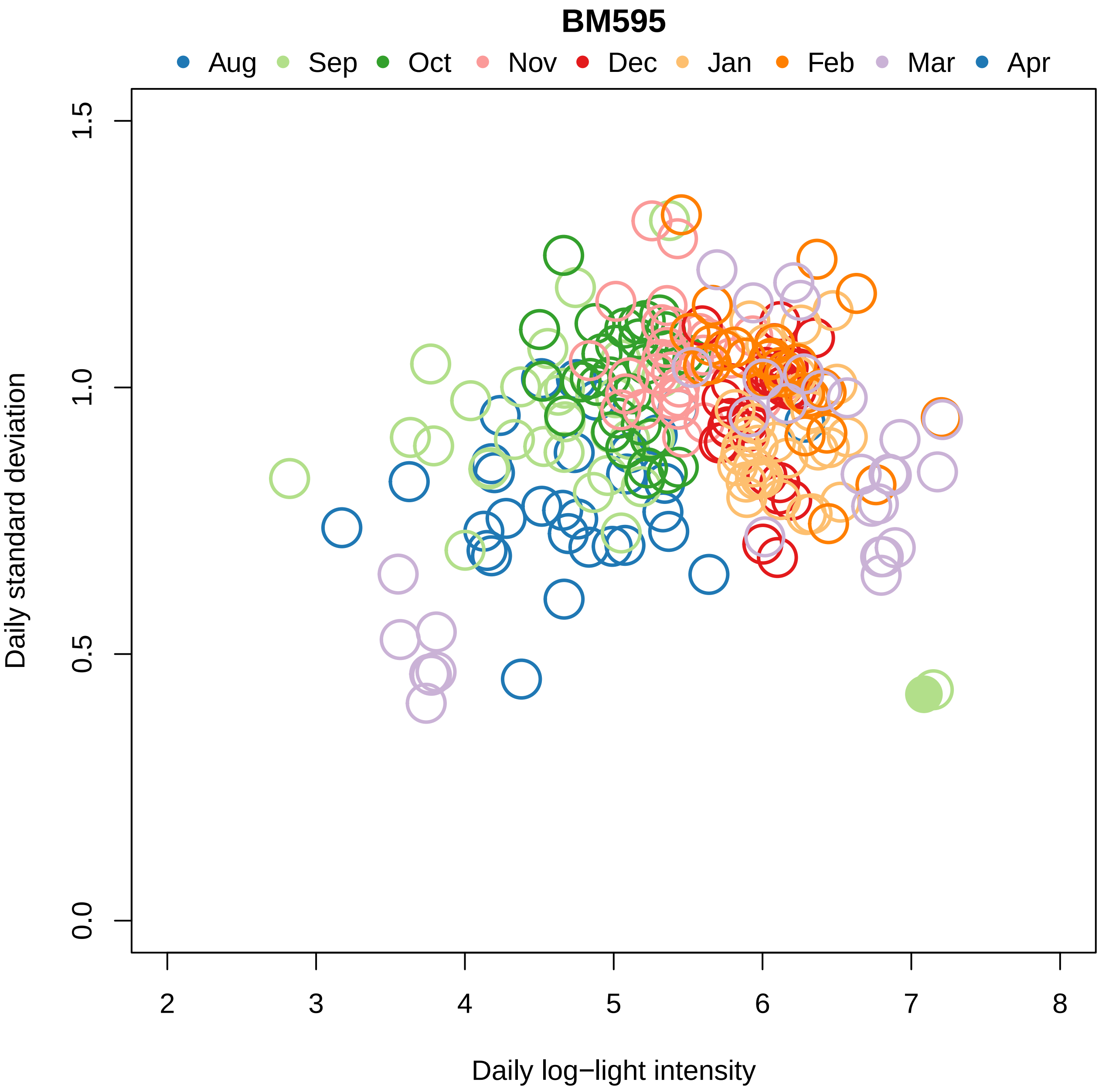
BM595
Aug
Sep
Oct
Nov
Dec
Jan
Feb
Mar
Apr
Daily standard deviation
0.0
0.5
1.0
1.5
2
3
4
5
6
7
8
Daily log−light intensity

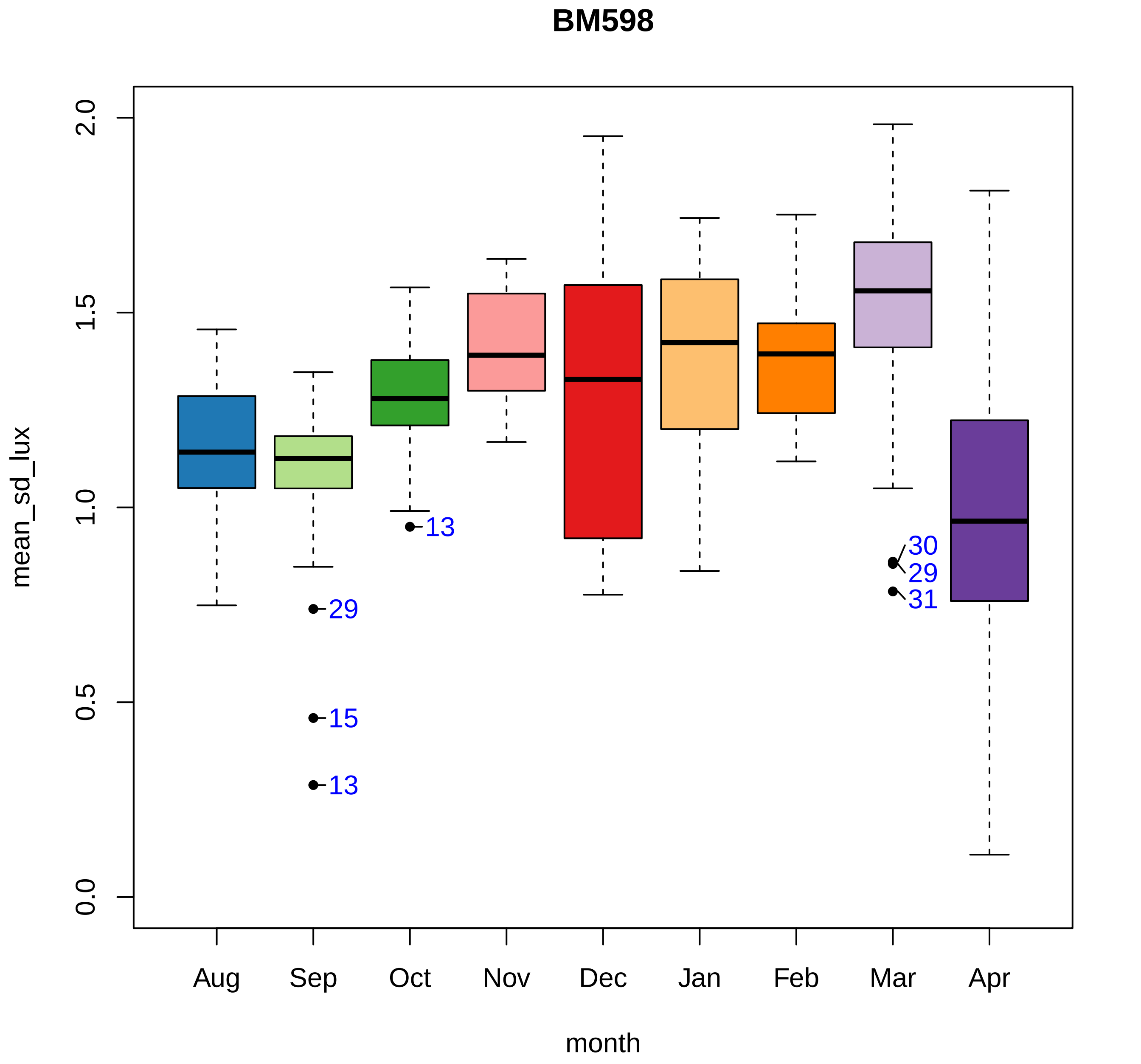
BM598
mean_sd_lux
month
0.0
0.5
1.0
1.5
2.0
Aug
Sep
Oct
Nov
Dec
Jan
Feb
Mar
Apr
29
15
13
13
30
29
31

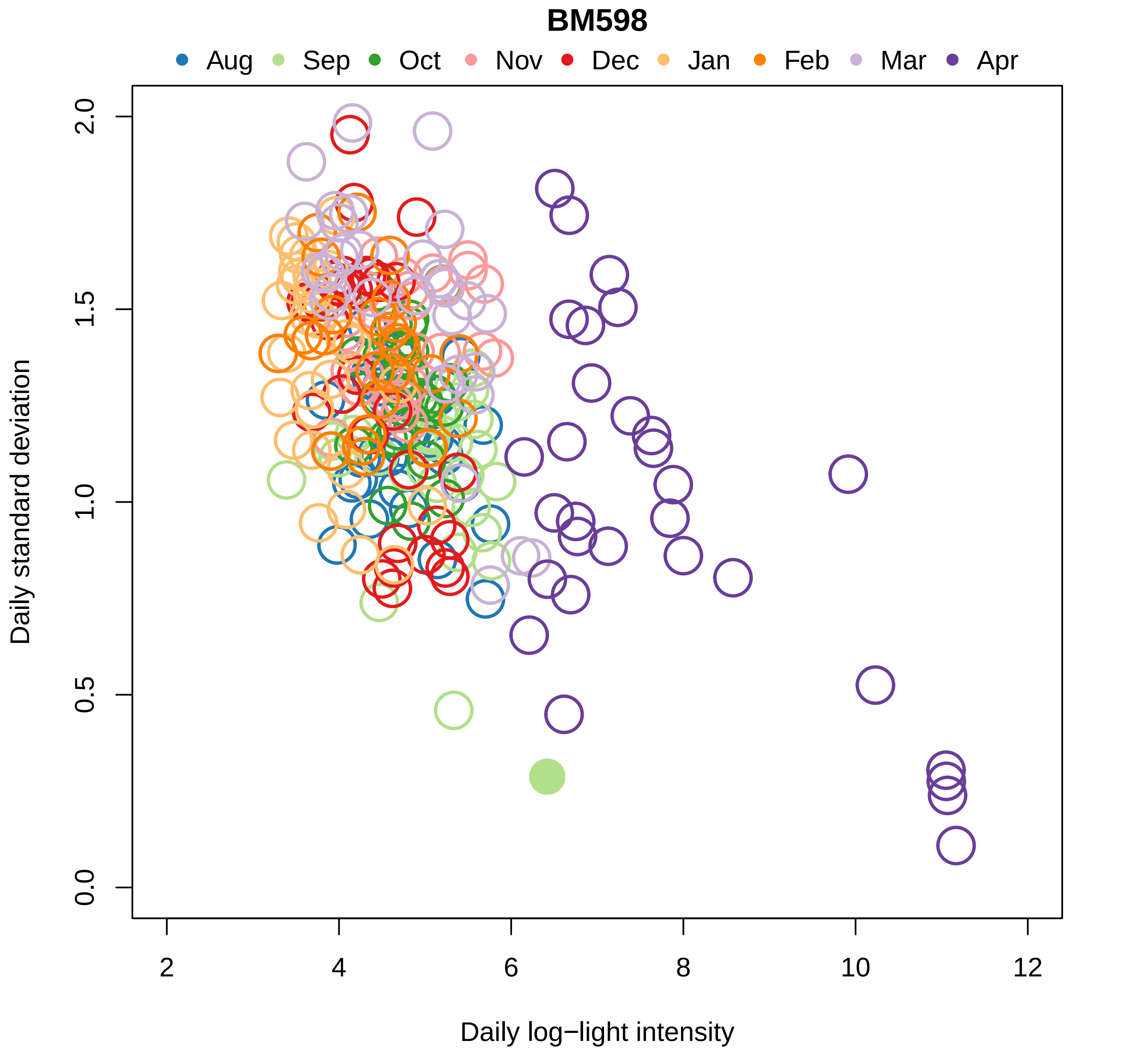
BM598
Aug
Sep
Oct
Nov
Dec
Jan
Feb
Mar
Apr
Daily standard deviation
Daily log−light intensity
0.0
0.5
1.0
1.5
2.0
2
4
6
8
10
12

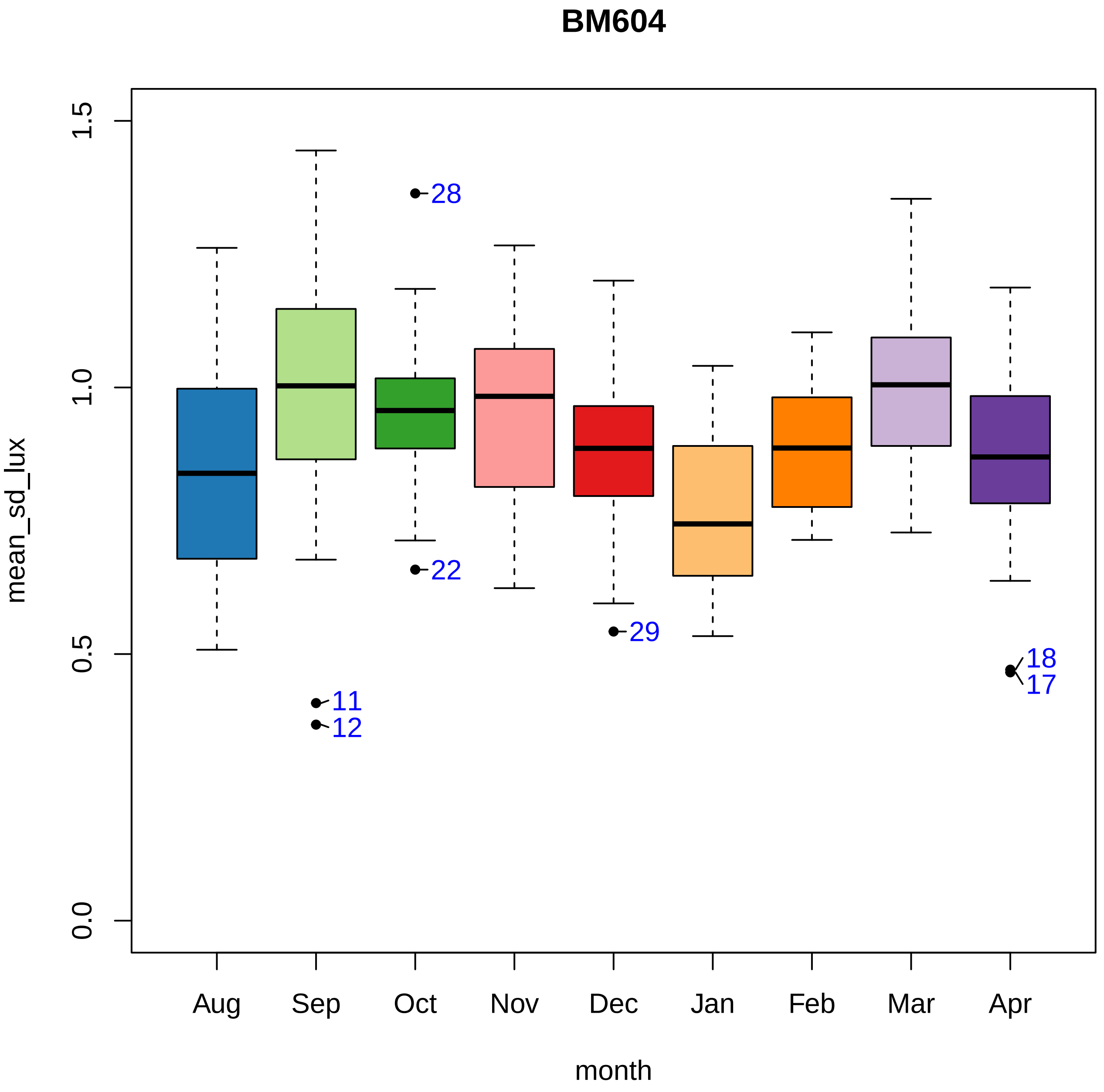
BM604
mean_sd_lux
month
0.0
0.5
1.0
1.5
Aug
Sep
Oct
Nov
Dec
Jan
Feb
Mar
Apr
28
22
11
12
29
18
17

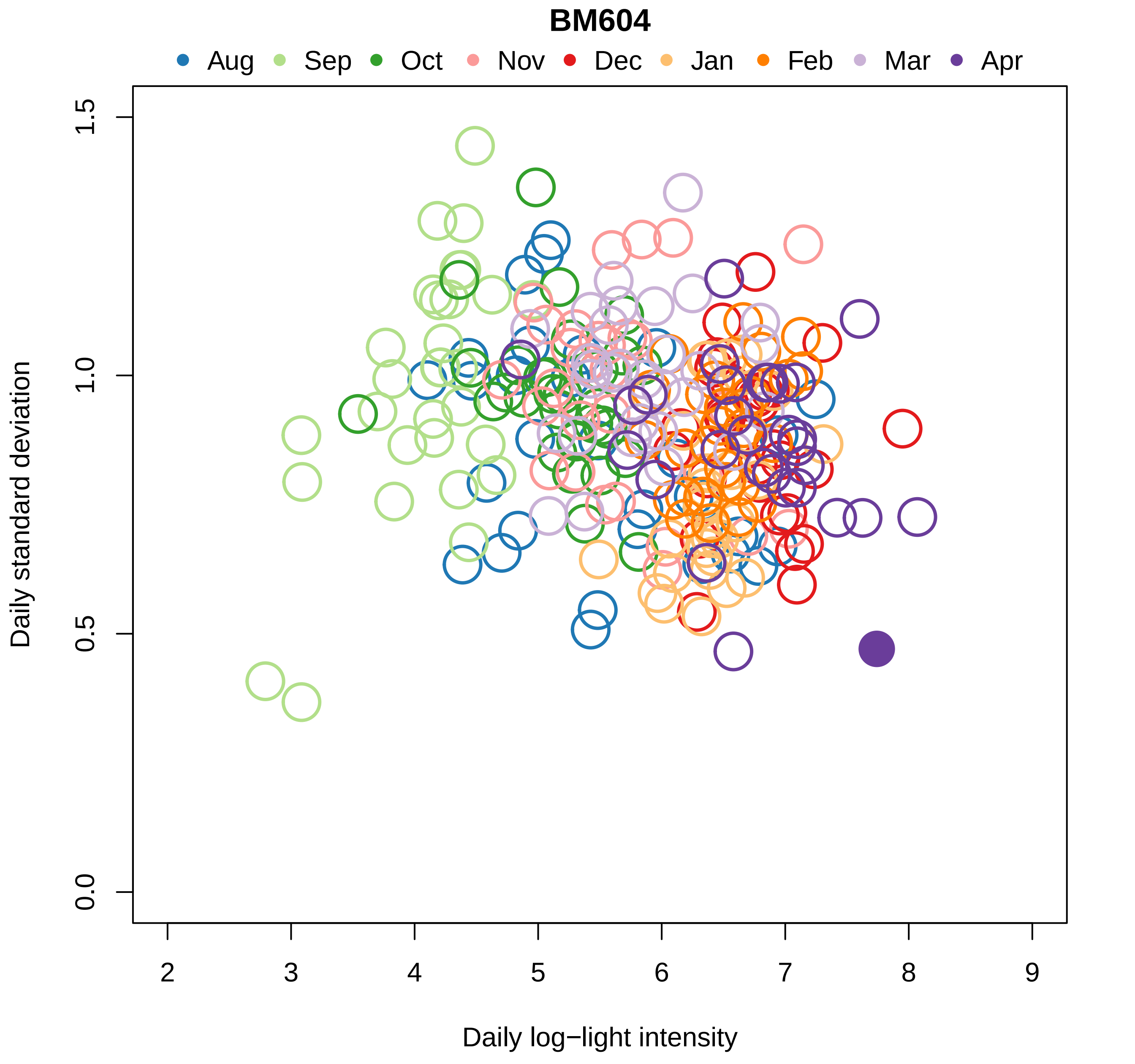
BM604
Aug
Sep
Oct
Nov
Dec
Jan
Feb
Mar
Apr
Daily standard deviation
0.0
0.5
1.0
1.5
2
3
4
5
6
7
8
9
Daily log−light intensity

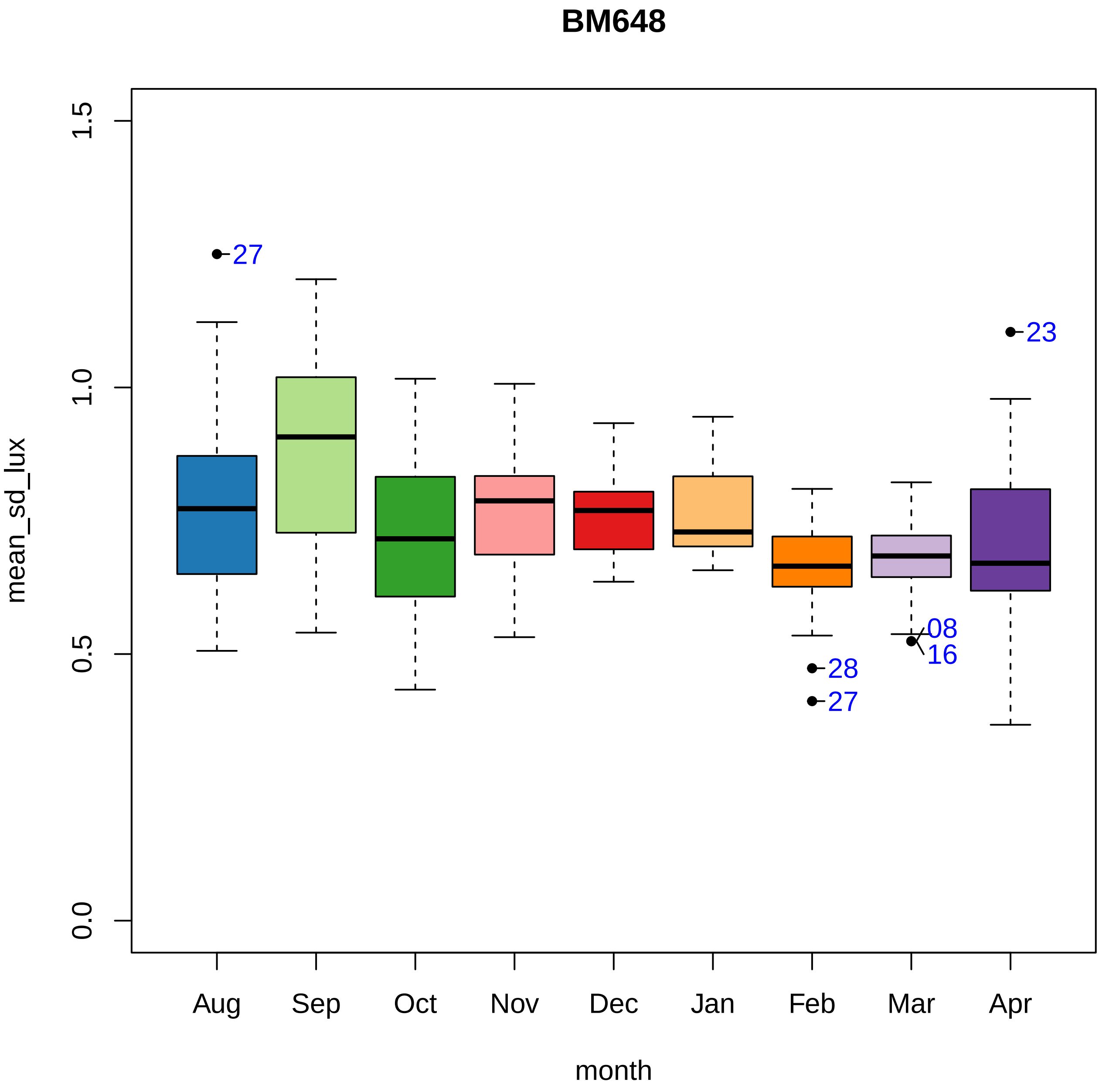

BM648
mean_sd_lux
month
0.0
0.5
1.0
1.5
Aug
Sep
Oct
Nov
Dec
Jan
Feb
Mar
Apr
27
23
08
16
28
27

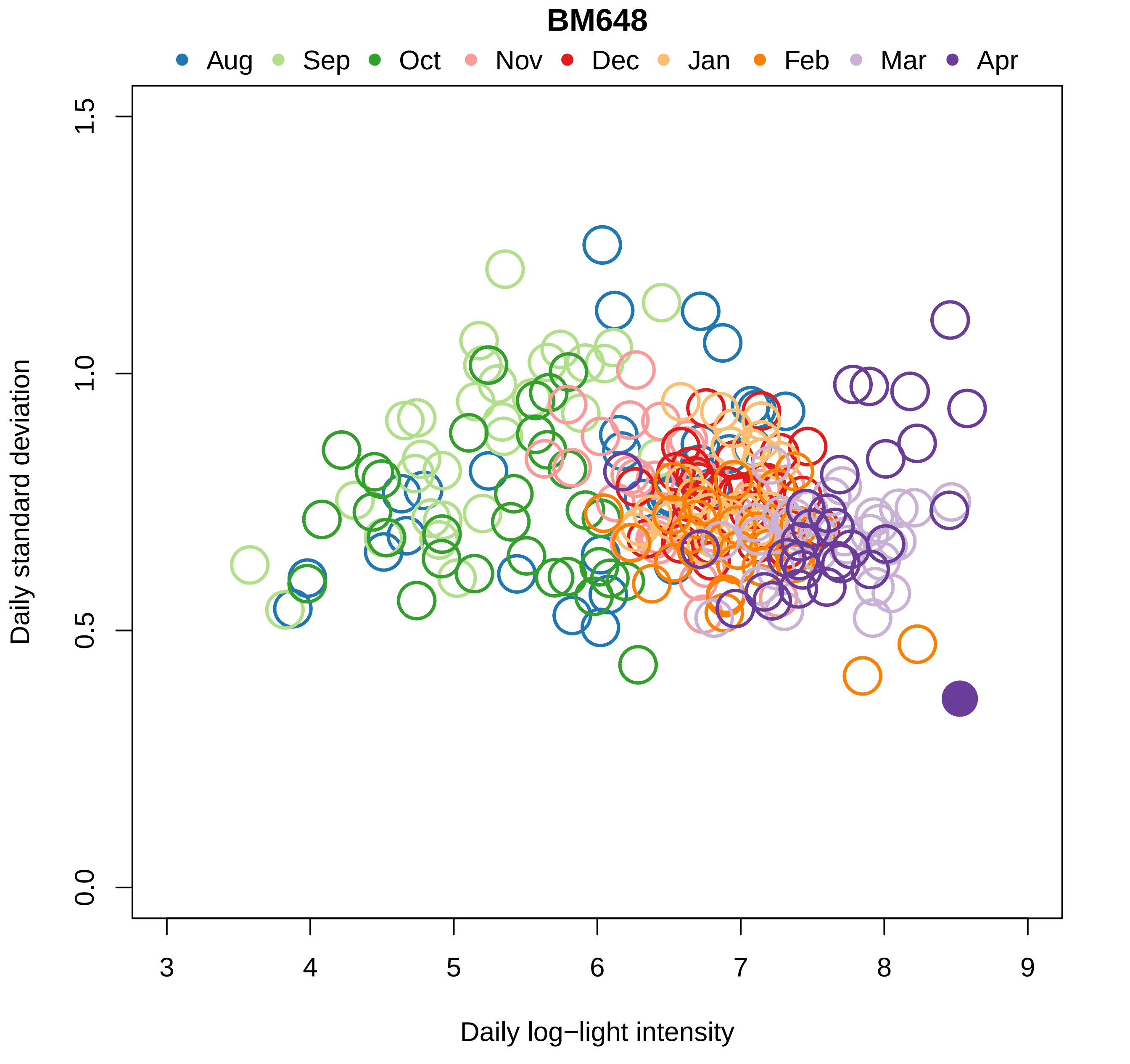
BM648
Aug
Sep
Oct
Nov
Dec
Jan
Feb
Mar
Apr
Daily standard deviation
0.0
0.5
1.0
1.5
3
4
5
6
7
8
9
Daily log−light intensity

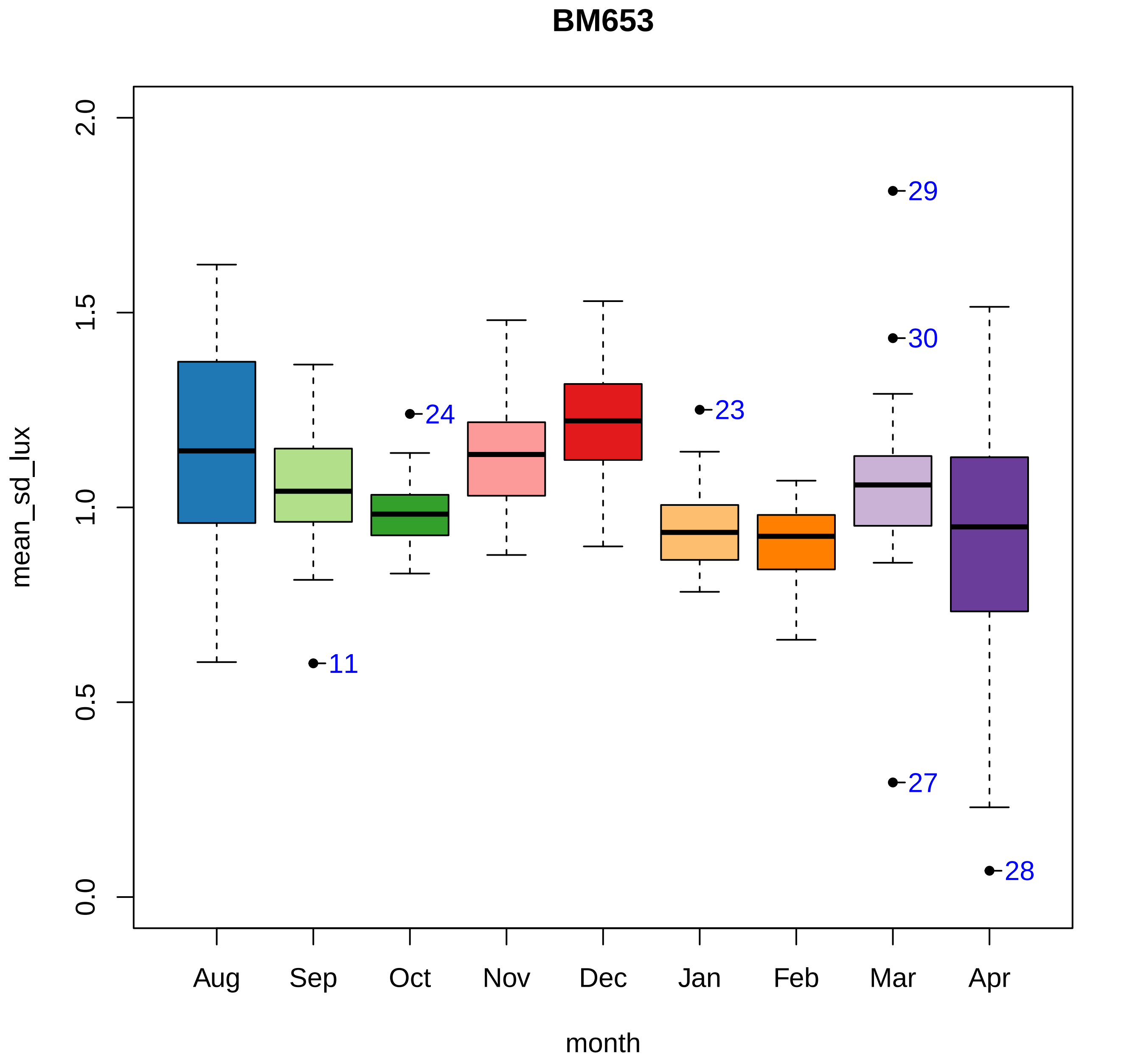
BM653
mean_sd_lux
0.0
0.5
1.0
1.5
2.0
Aug
Sep
Oct
Nov
Dec
Jan
Feb
Mar
Apr
month
29
30
24
23
11
27
28

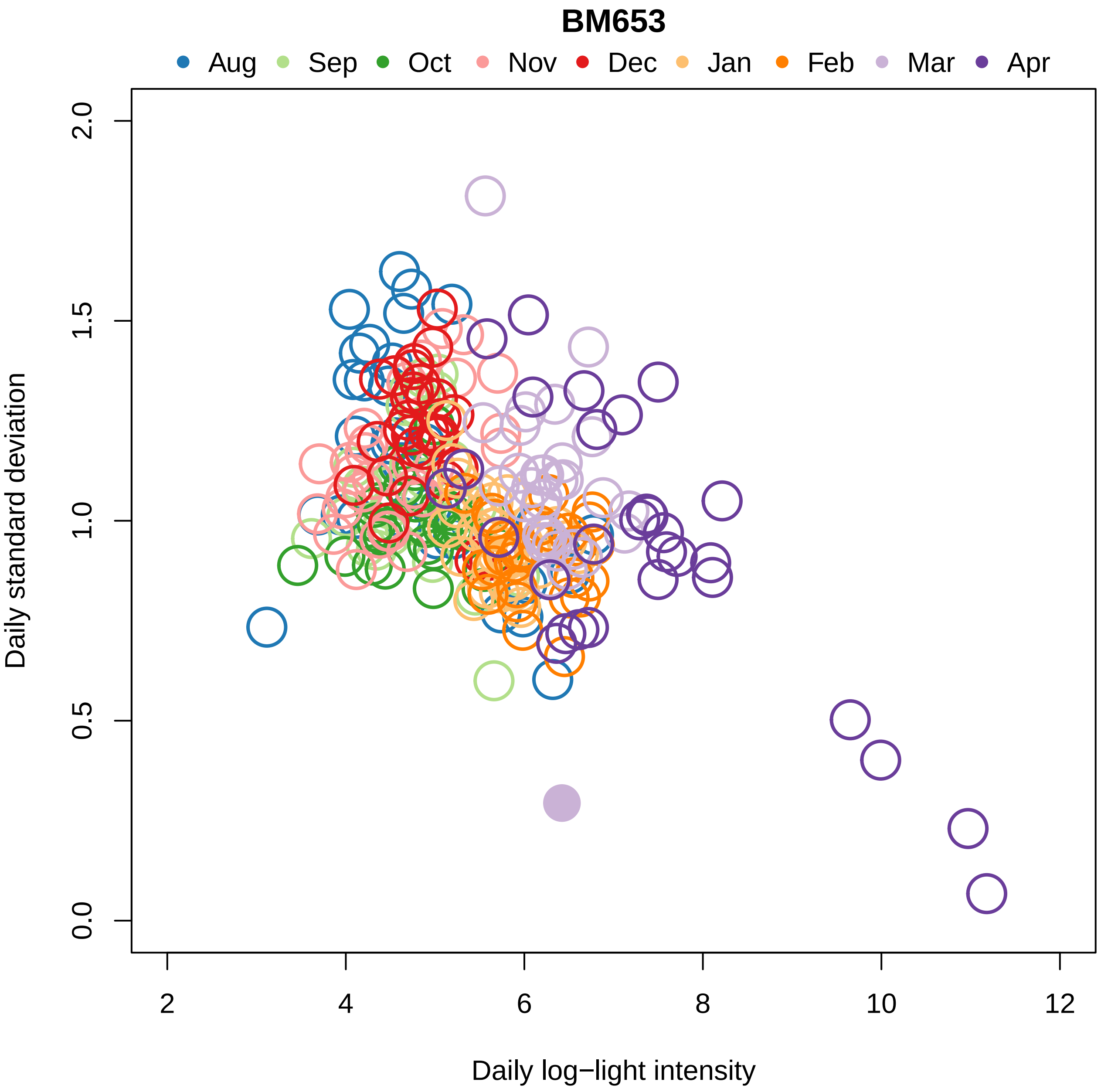
BM653
Aug
Sep
Oct
Nov
Dec
Jan
Feb
Mar
Apr
Daily standard deviation
0.0
0.5
1.0
1.5
2.0
2
4
6
8
10
12
Daily log−light intensity

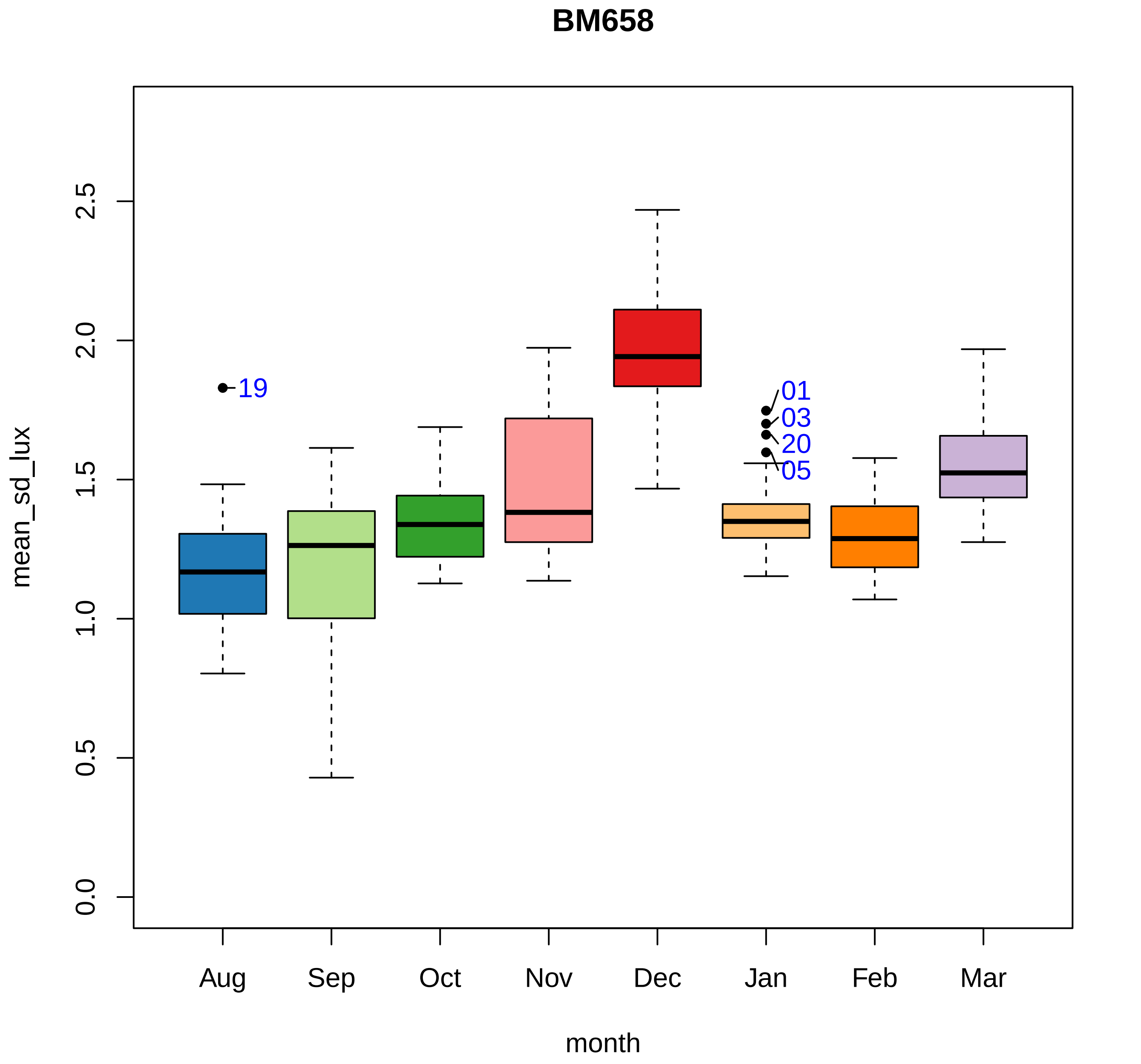

BM658
mean_sd_lux
0.0
0.5
1.0
1.5
2.0
2.5
19
01
03
20
05
Aug
Sep
Oct
Nov
Dec
Jan
Feb
Mar
month

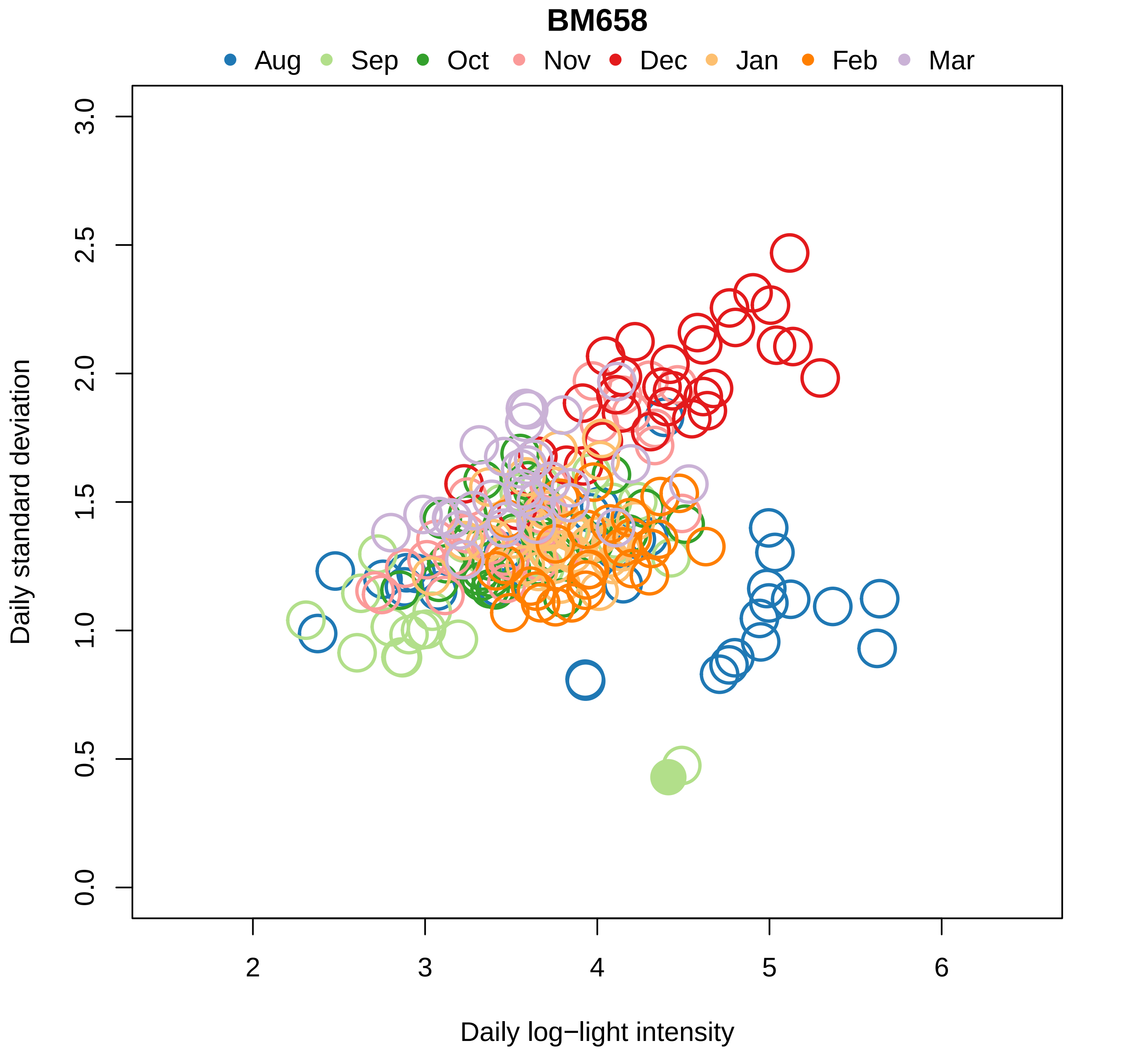
BM658
Aug
Sep
Oct
Nov
Dec
Jan
Feb
Mar
Daily standard deviation
0.0
0.5
1.0
1.5
2.0
2.5
3.0
2
3
4
5
6
Daily log−light intensity

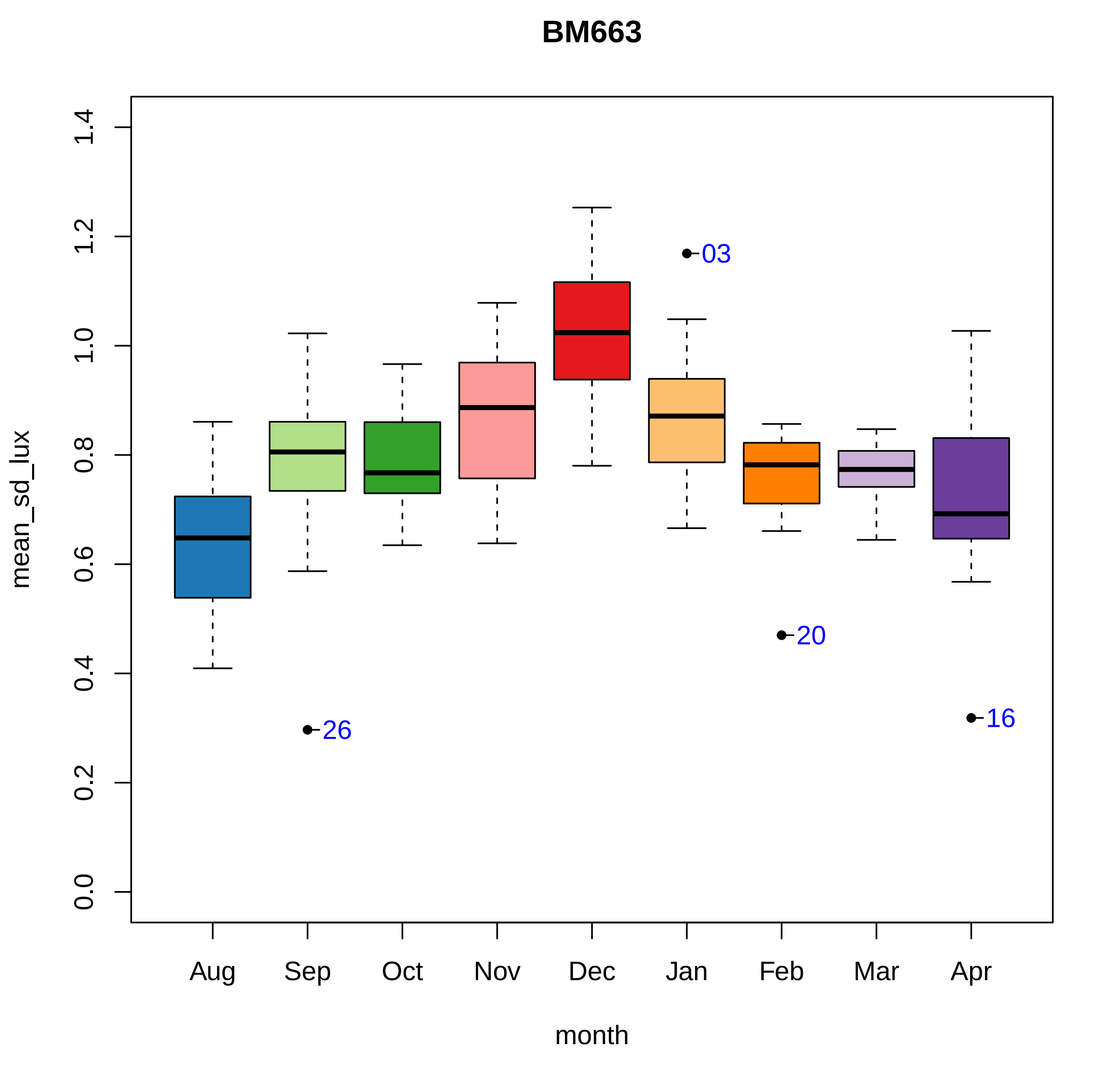
BM663
mean_sd_lux
0.0
0.2
0.4
0.6
0.8
1.0
1.2
1.4
03
26
20
16
Aug
Sep
Oct
Nov
Dec
Jan
Feb
Mar
Apr
month

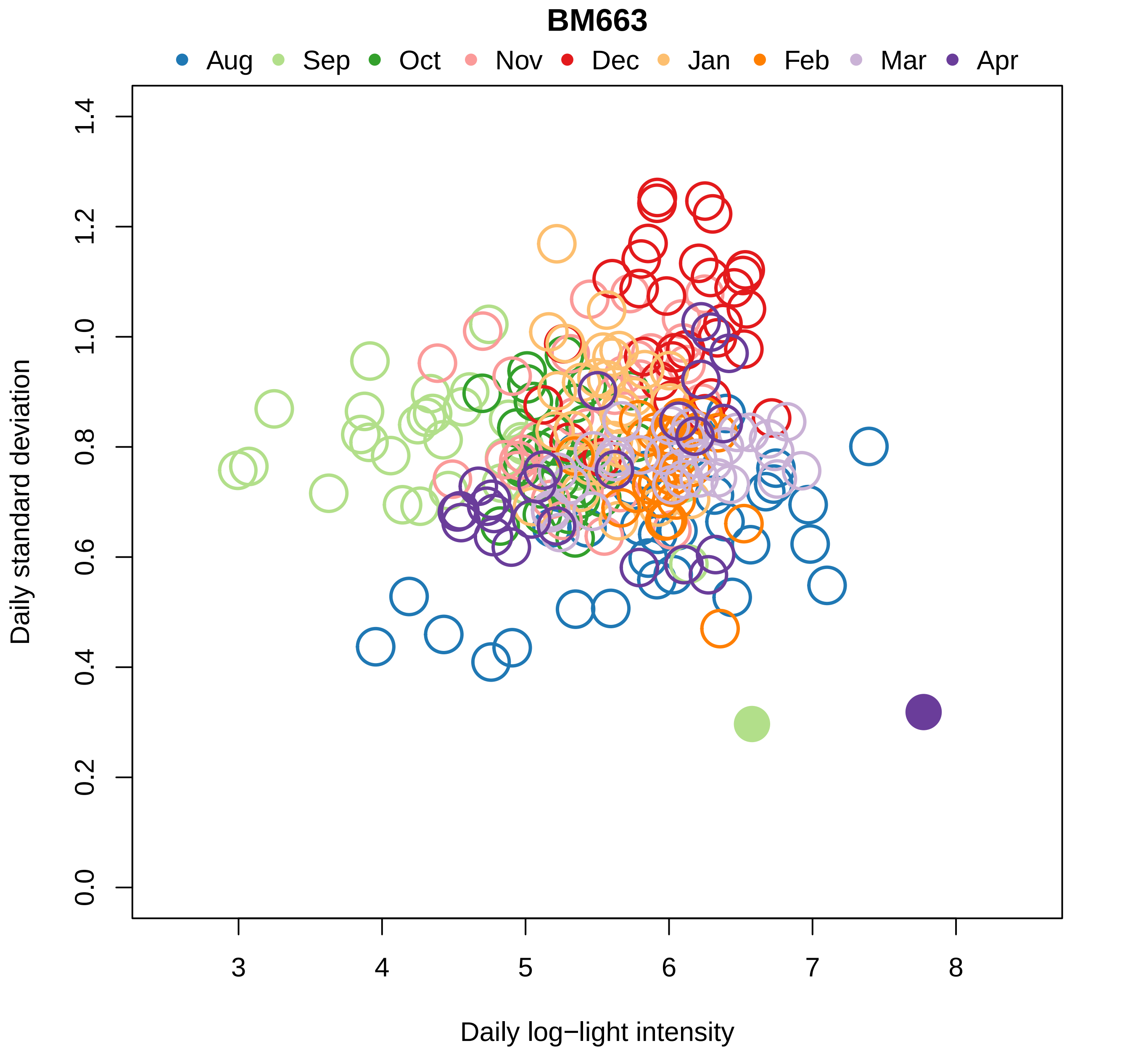
BM663
Aug
Sep
Oct
Nov
Dec
Jan
Feb
Mar
Apr
Daily standard deviation
0.0
0.2
0.4
0.6
0.8
1.0
1.2
1.4
3
4
5
6
7
8
Daily log−light intensity

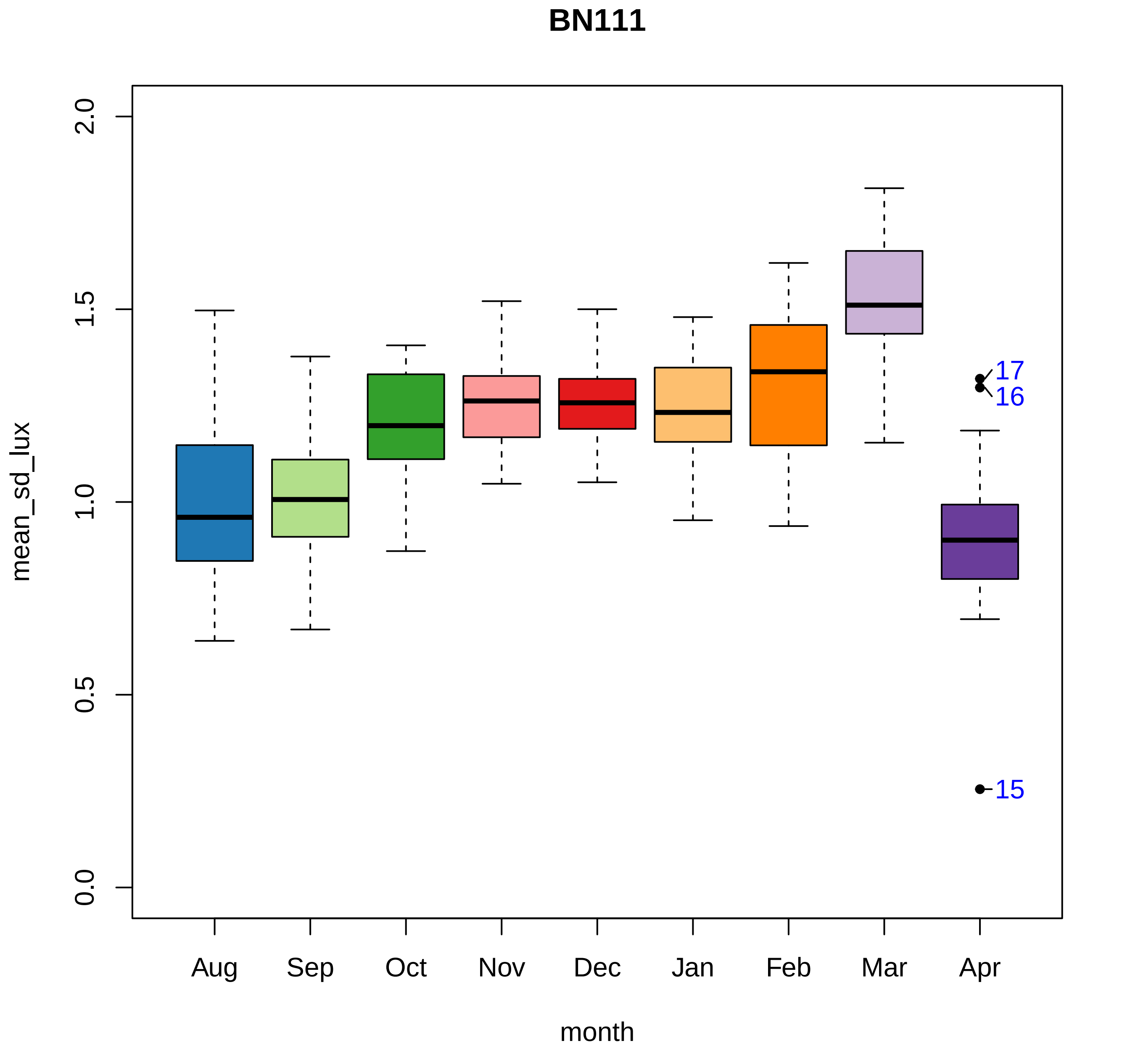
BN111
mean_sd_lux
0.0
0.5
1.0
1.5
2.0
Aug
Sep
Oct
Nov
Dec
Jan
Feb
Mar
Apr
month
17
16
15

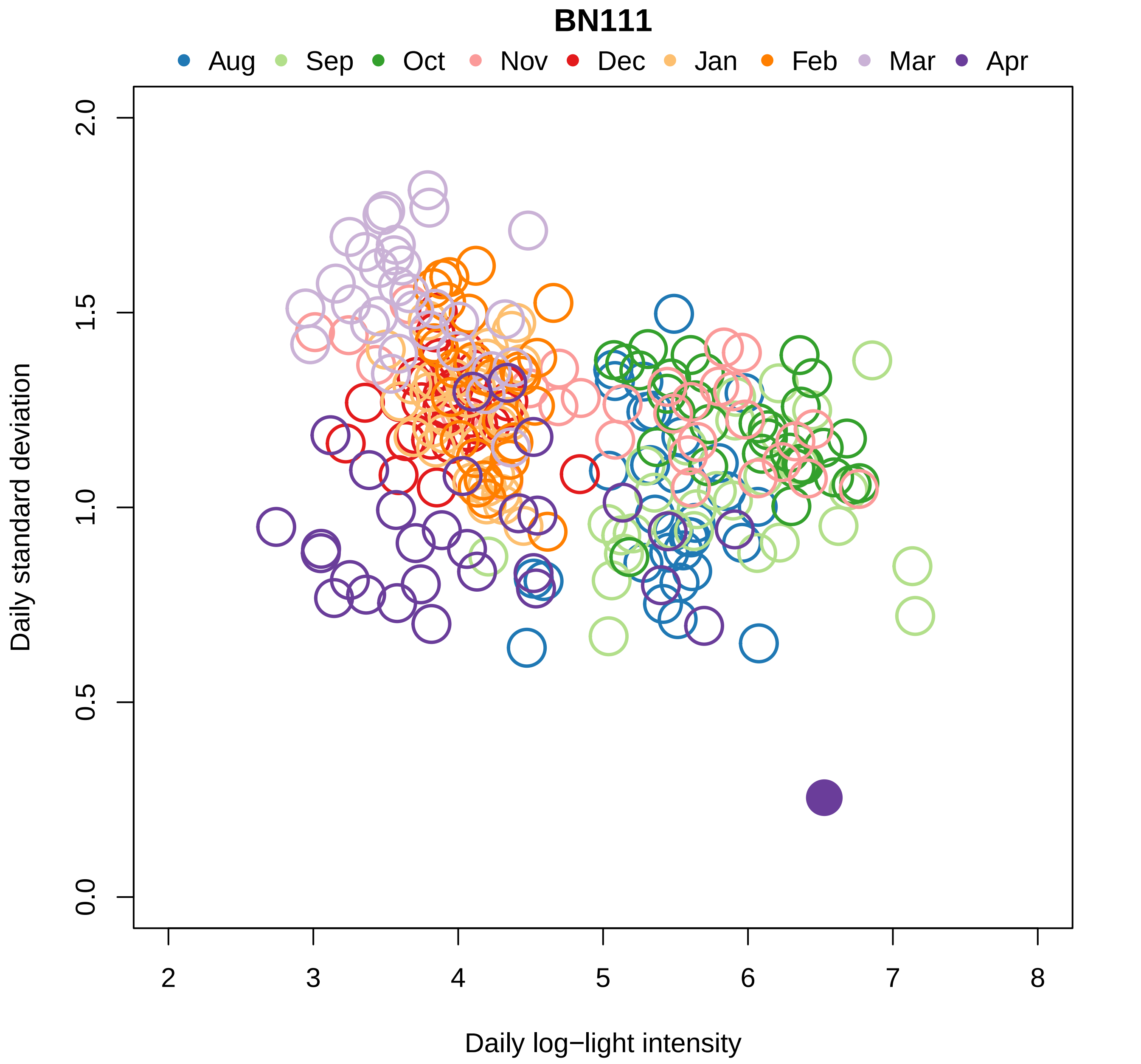
BN111
Aug
Sep
Oct
Nov
Dec
Jan
Feb
Mar
Apr
Daily standard deviation
0.0
0.5
1.0
1.5
2.0
2
3
4
5
6
7
8
Daily log−light intensity

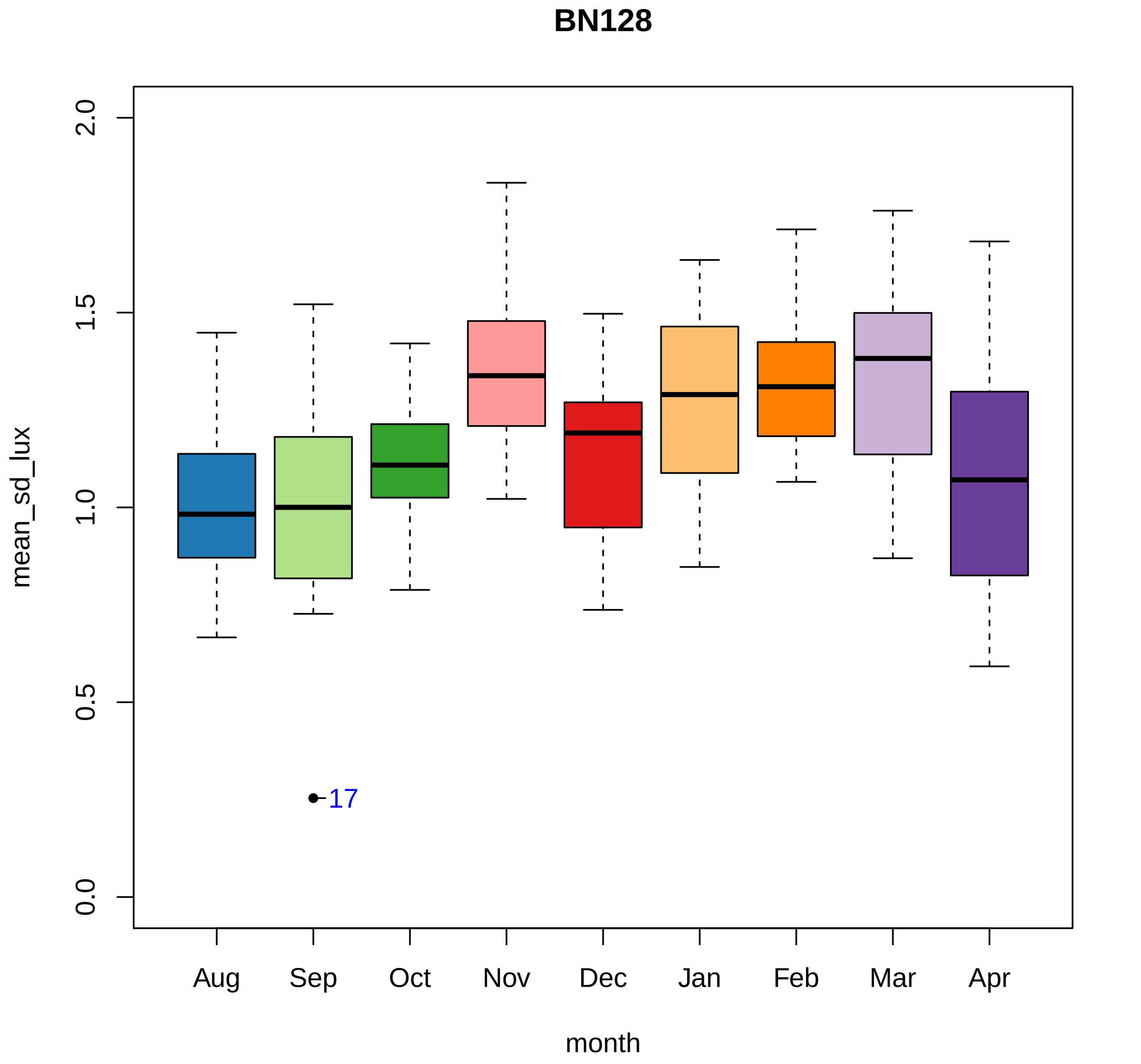
BN128
mean_sd_lux
0.0
0.5
1.0
1.5
2.0
17
Aug
Sep
Oct
Nov
Dec
Jan
Feb
Mar
Apr
month

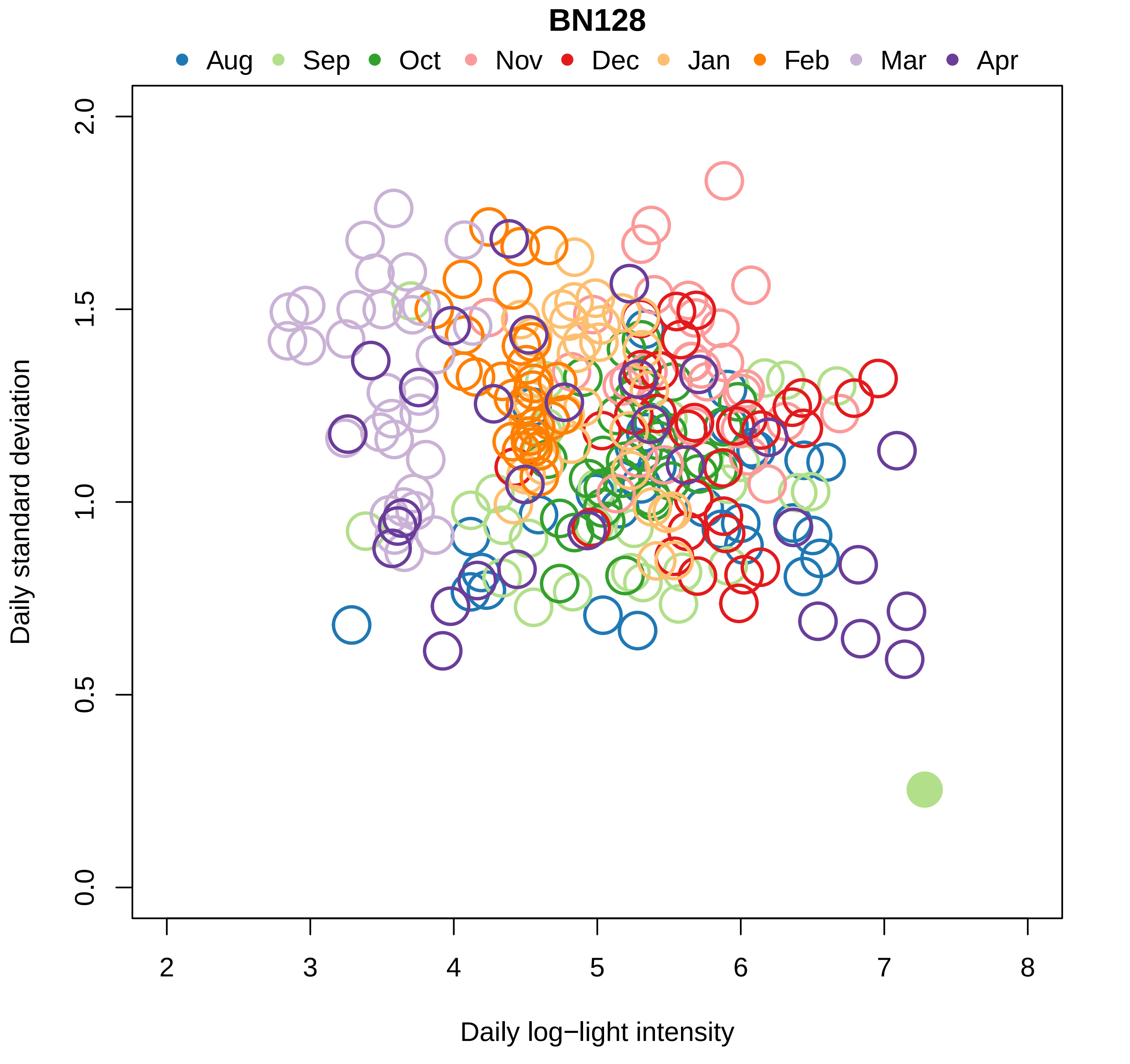

BN128
Aug
Sep
Oct
Nov
Dec
Jan
Feb
Mar
Apr
Daily standard deviation
0.0
0.5
1.0
1.5
2.0
2
3
4
5
6
7
8
Daily log−light intensity

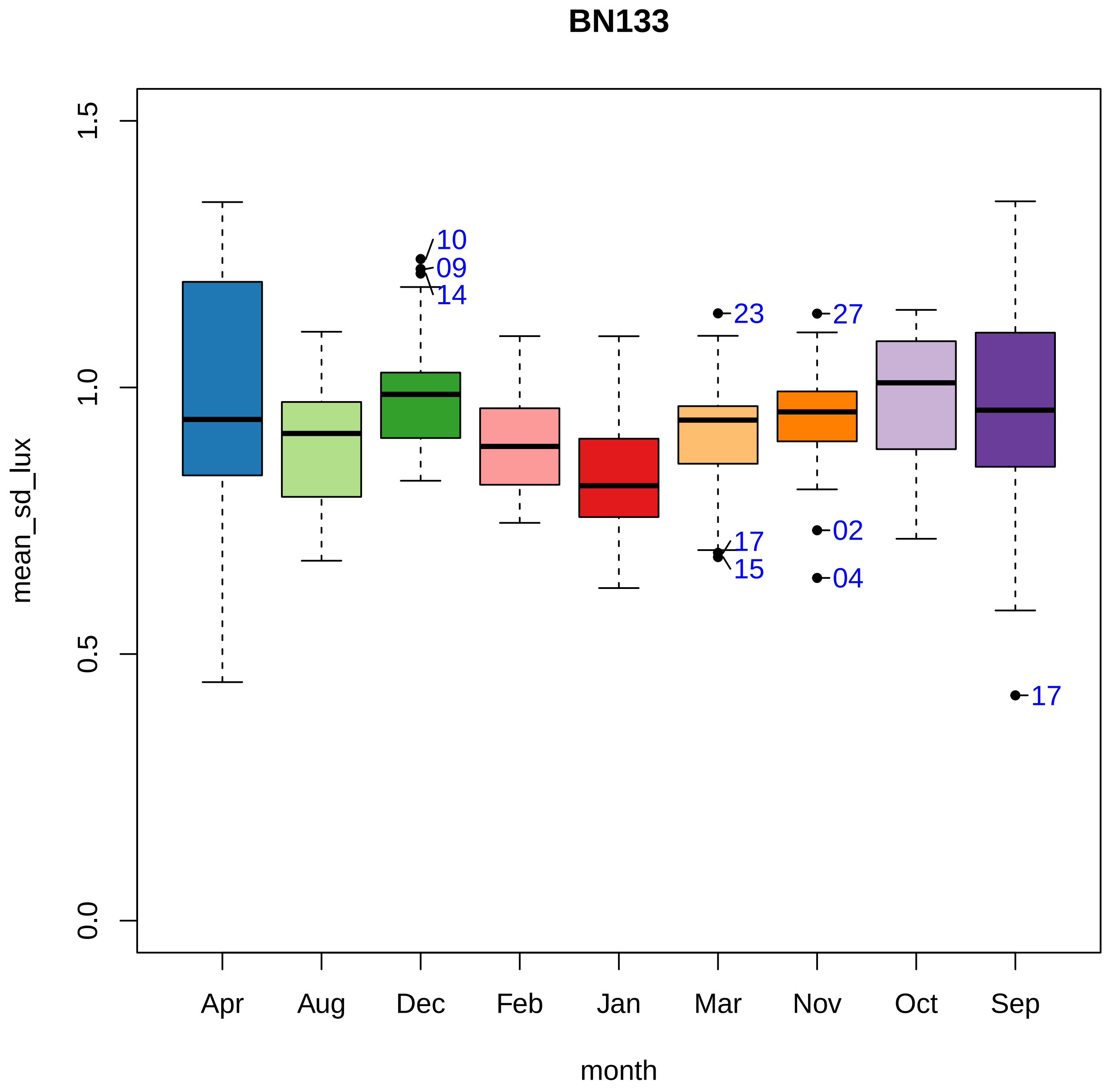
BN133
mean_sd_lux
month
0.0
0.5
1.0
1.5
Apr
Aug
Dec
Feb
Jan
Mar
Nov
Oct
Sep
10
09
14
23
27
17
15
02
04
17

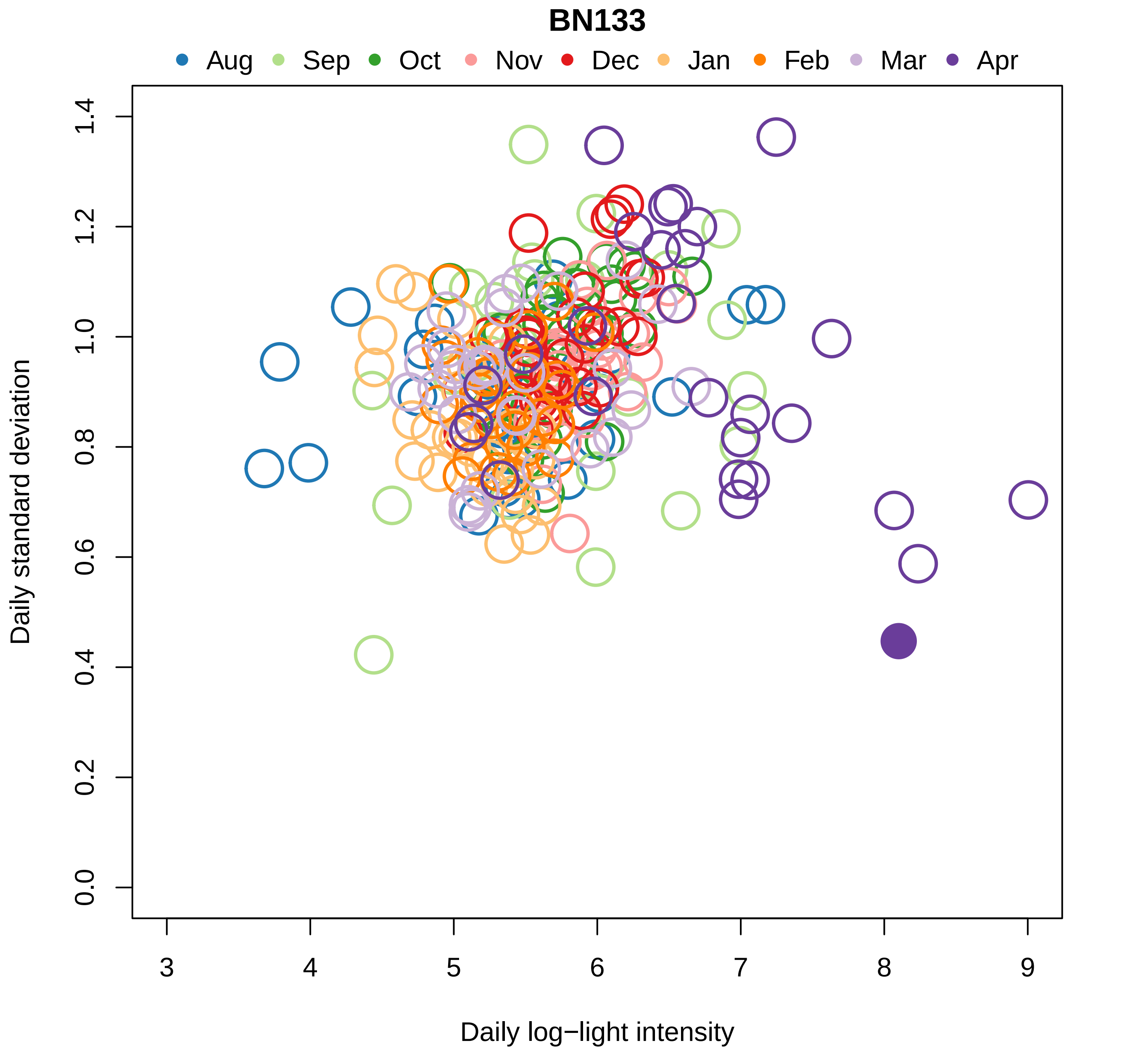
BN133
Aug Sep Oct Nov Dec Jan Feb Mar Apr
Daily standard deviation
0.0 0.2 0.4 0.6 0.8 1.0 1.2 1.4
3 4 5 6 7 8 9
Daily log−light intensity

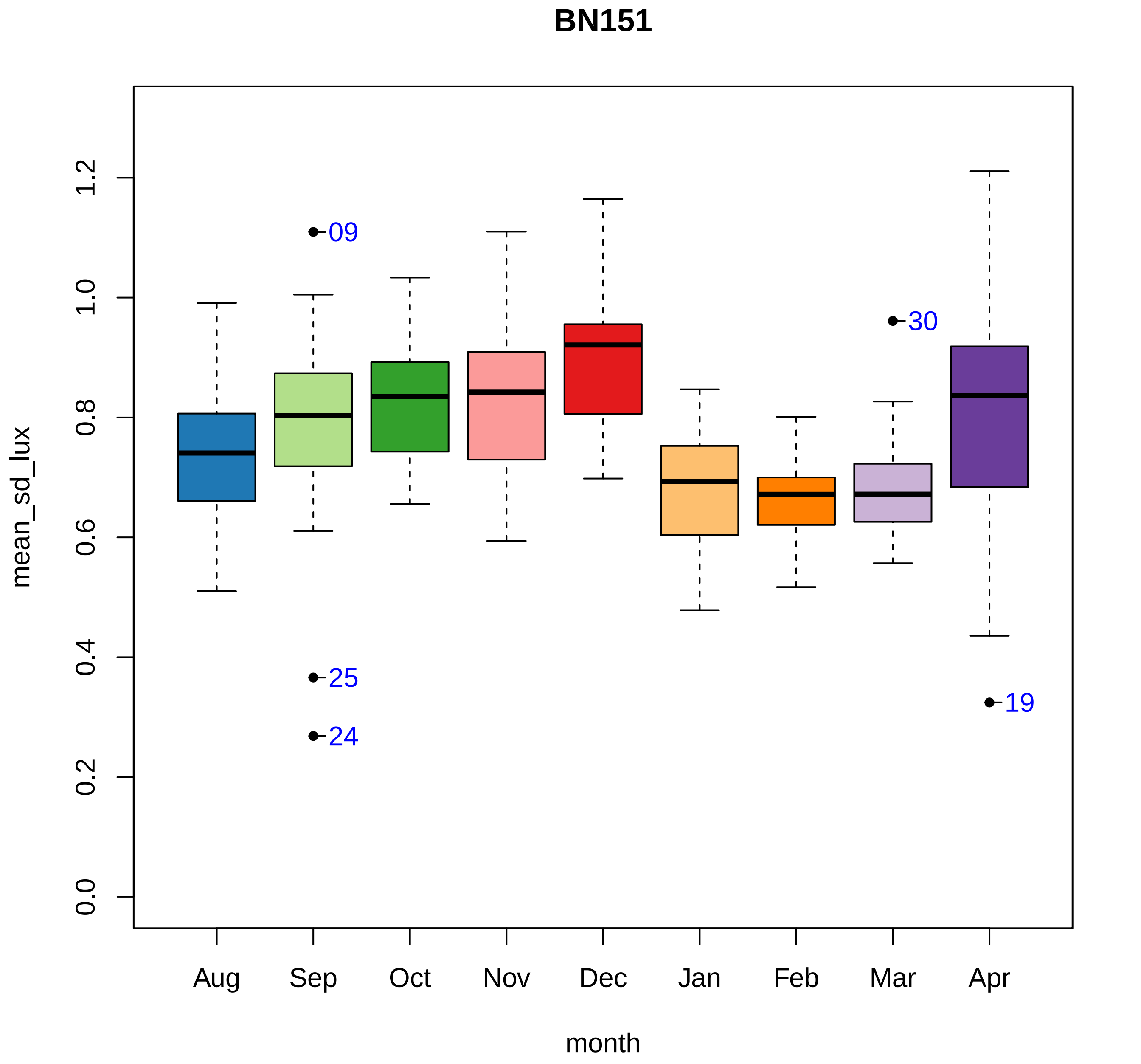
BN151
mean_sd_lux
0.0
0.2
0.4
0.6
0.8
1.0
1.2
09
25
24
30
19
Aug
Sep
Oct
Nov
Dec
Jan
Feb
Mar
Apr
month

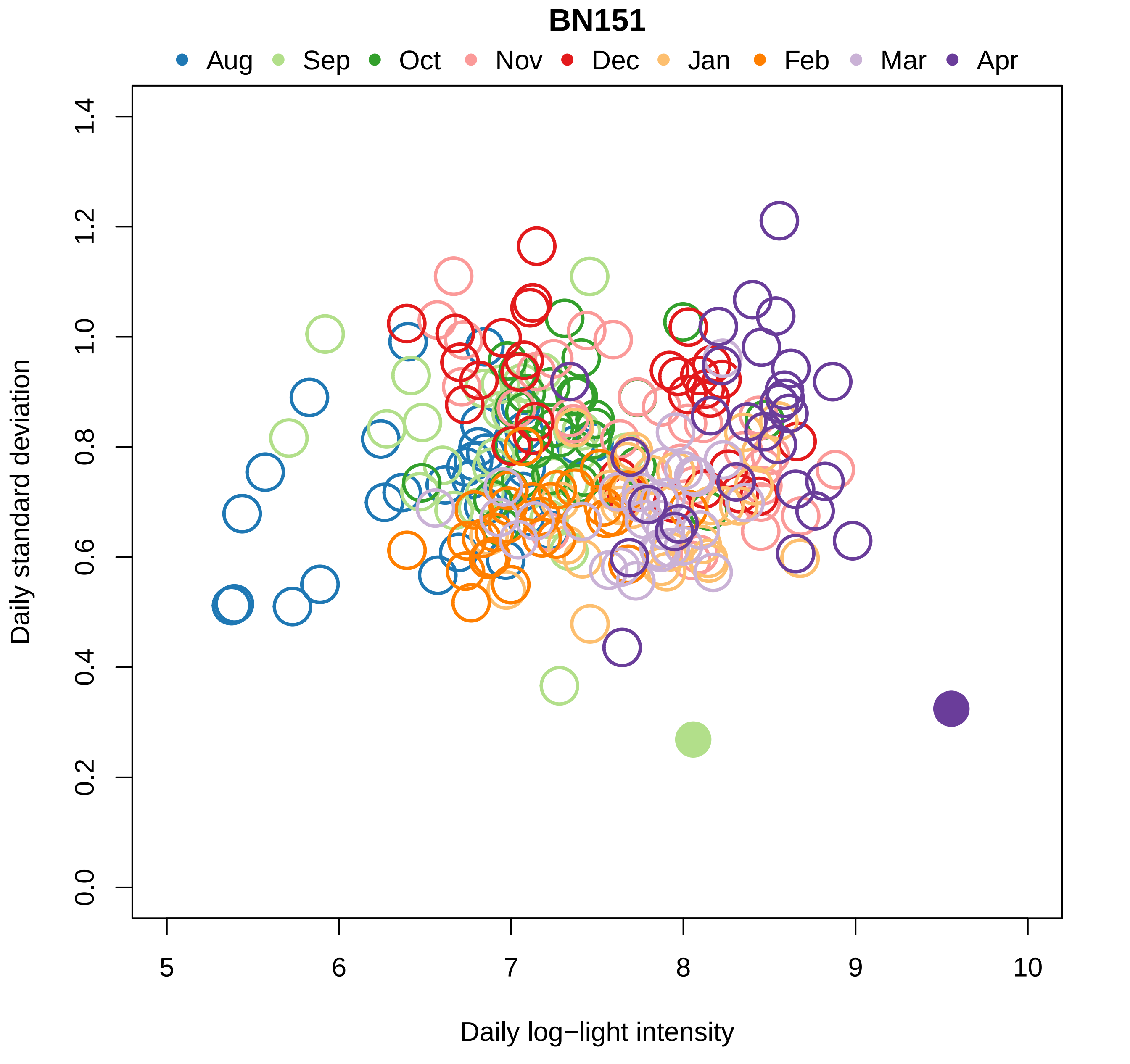
BN151
Aug
Sep
Oct
Nov
Dec
Jan
Feb
Mar
Apr
Daily standard deviation
0.0
0.2
0.4
0.6
0.8
1.0
1.2
1.4
5
6
7
8
9
10
Daily log−light intensity

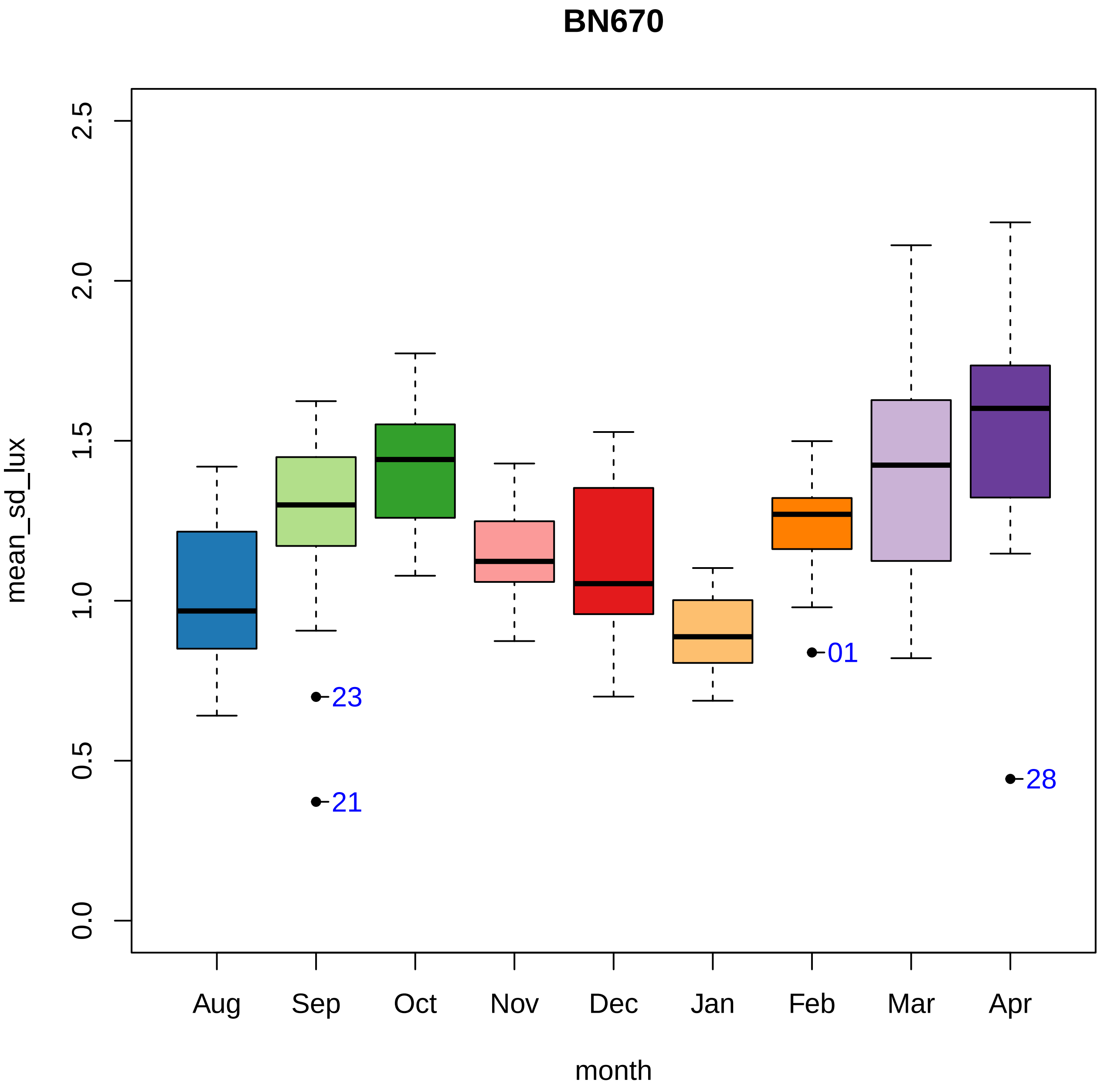
BN670
mean_sd_lux
month
0.0
0.5
1.0
1.5
2.0
2.5
Aug
Sep
Oct
Nov
Dec
Jan
Feb
Mar
Apr
23
21
01
28

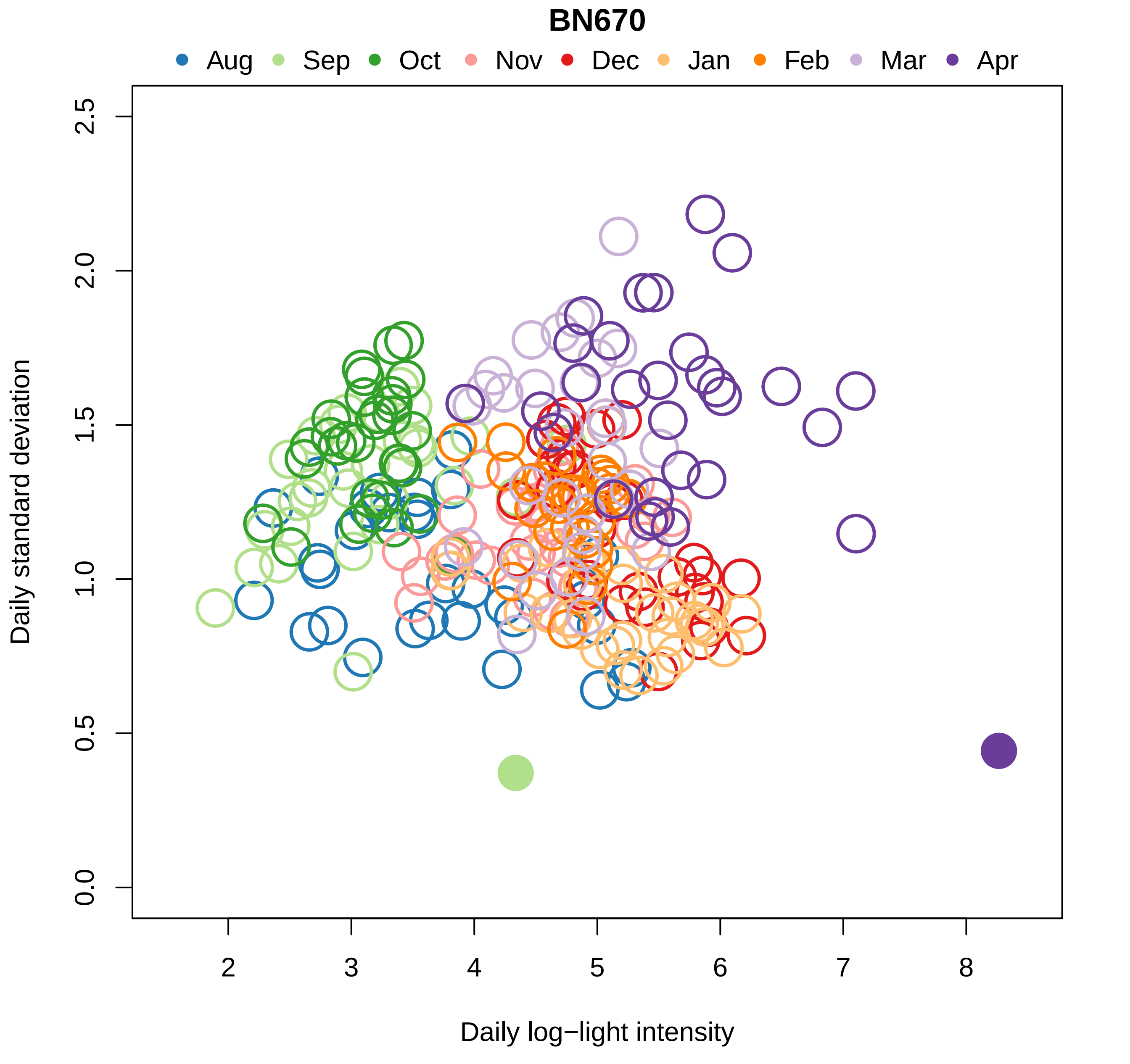
BN670
Aug
Sep
Oct
Nov
Dec
Jan
Feb
Mar
Apr
Daily standard deviation
0.0
0.5
1.0
1.5
2.0
2.5
2
3
4
5
6
7
8
Daily log−light intensity

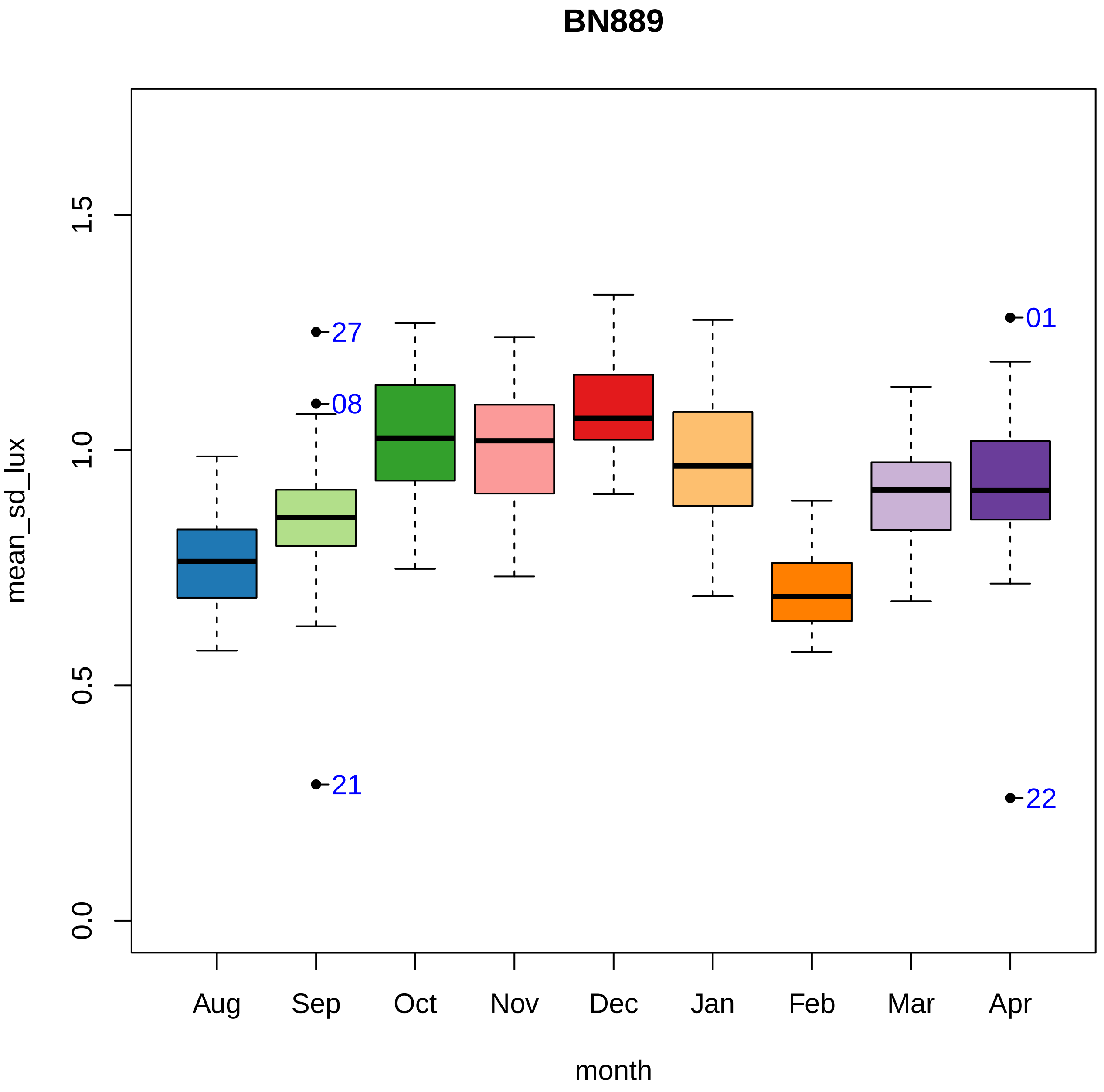

BN889
mean_sd_lux
0.0
0.5
1.0
1.5
27
08
21
01
22
Aug
Sep
Oct
Nov
Dec
Jan
Feb
Mar
Apr
month

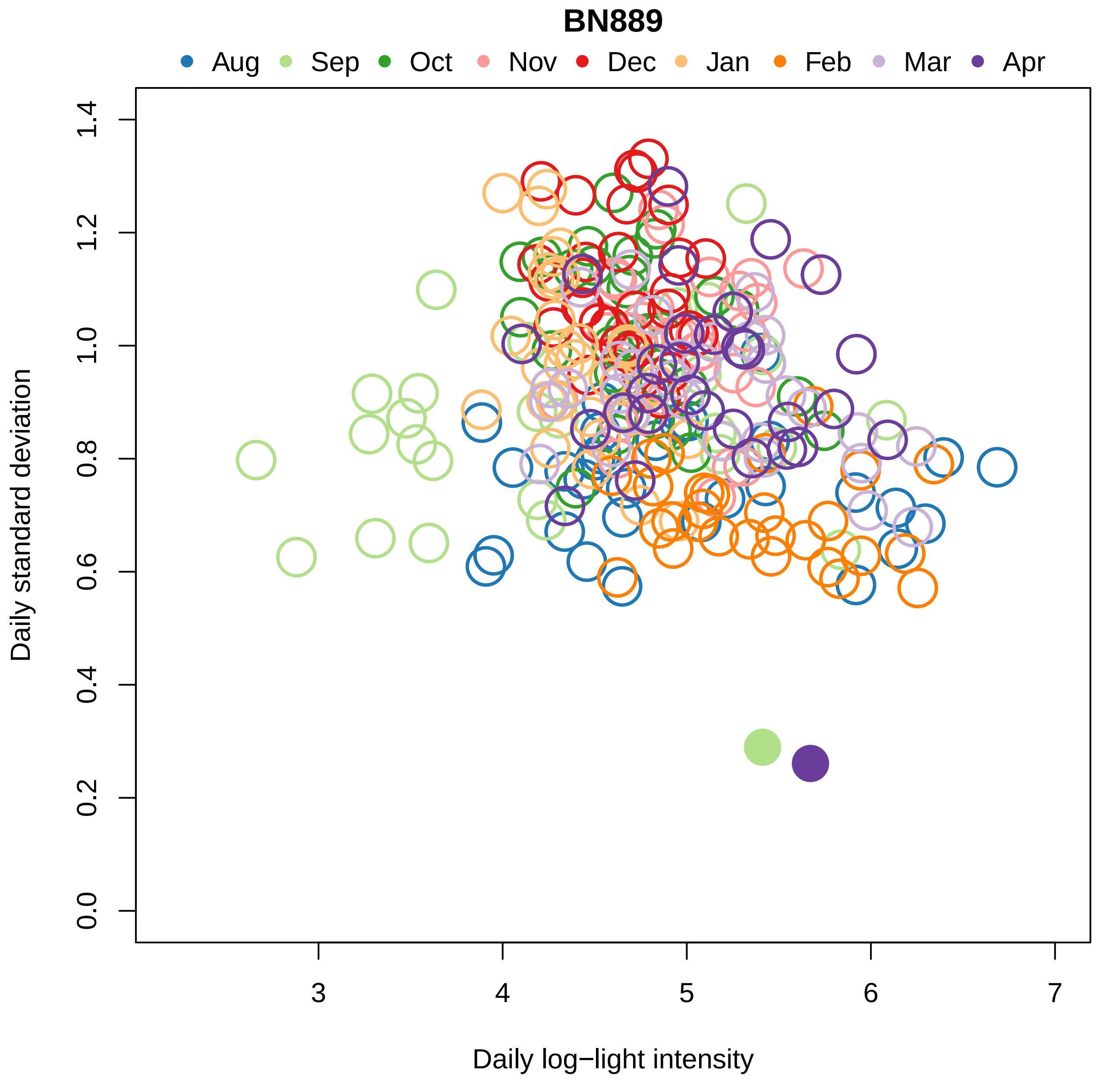
BN889
Aug
Sep
Oct
Nov
Dec
Jan
Feb
Mar
Apr
Daily standard deviation
0.0
0.2
0.4
0.6
0.8
1.0
1.2
1.4
3
4
5
6
7
Daily log−light intensity

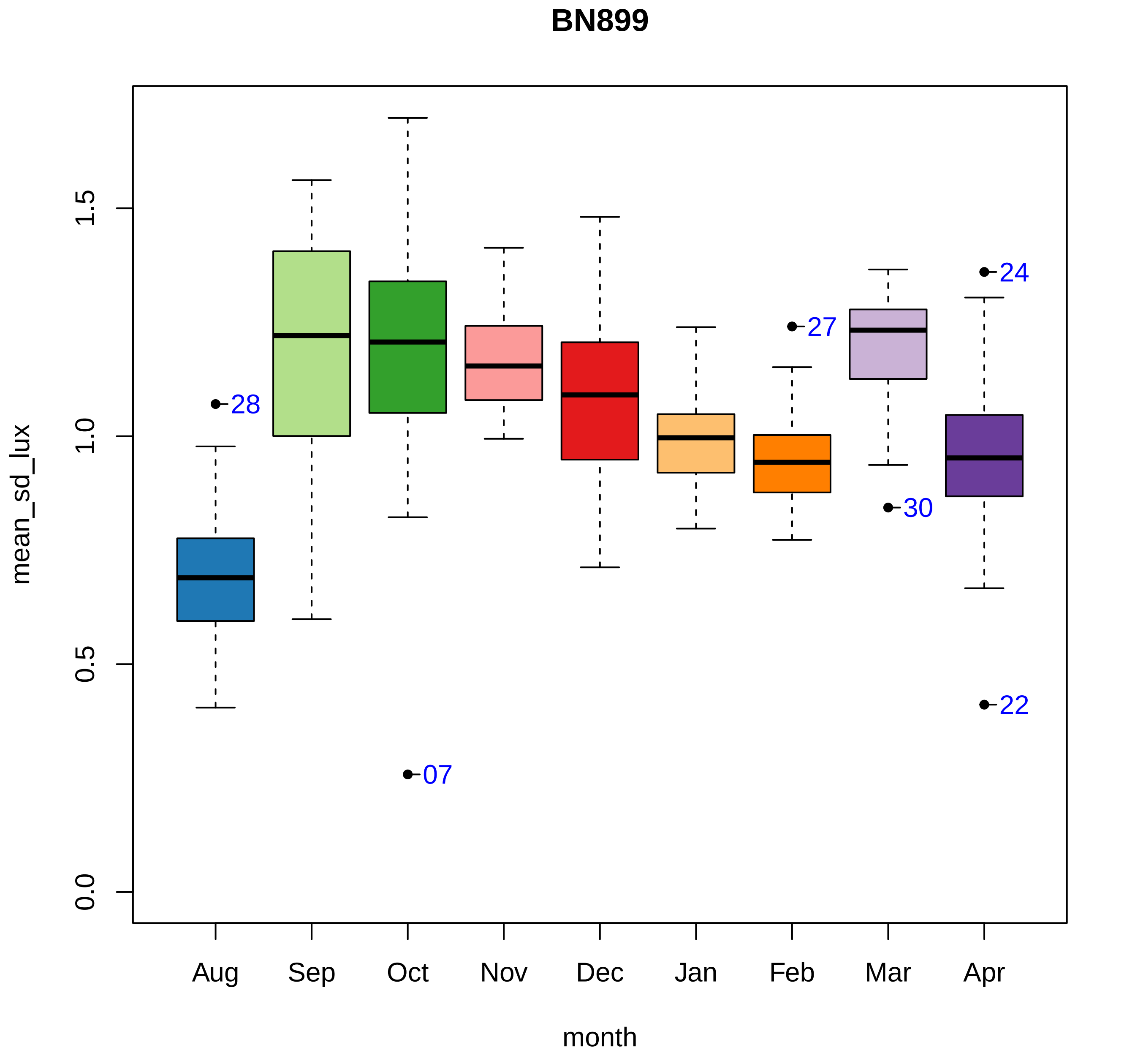
BN899
mean_sd_lux
0.0
0.5
1.0
1.5
28
07
27
30
24
22
Aug
Sep
Oct
Nov
Dec
Jan
Feb
Mar
Apr
month

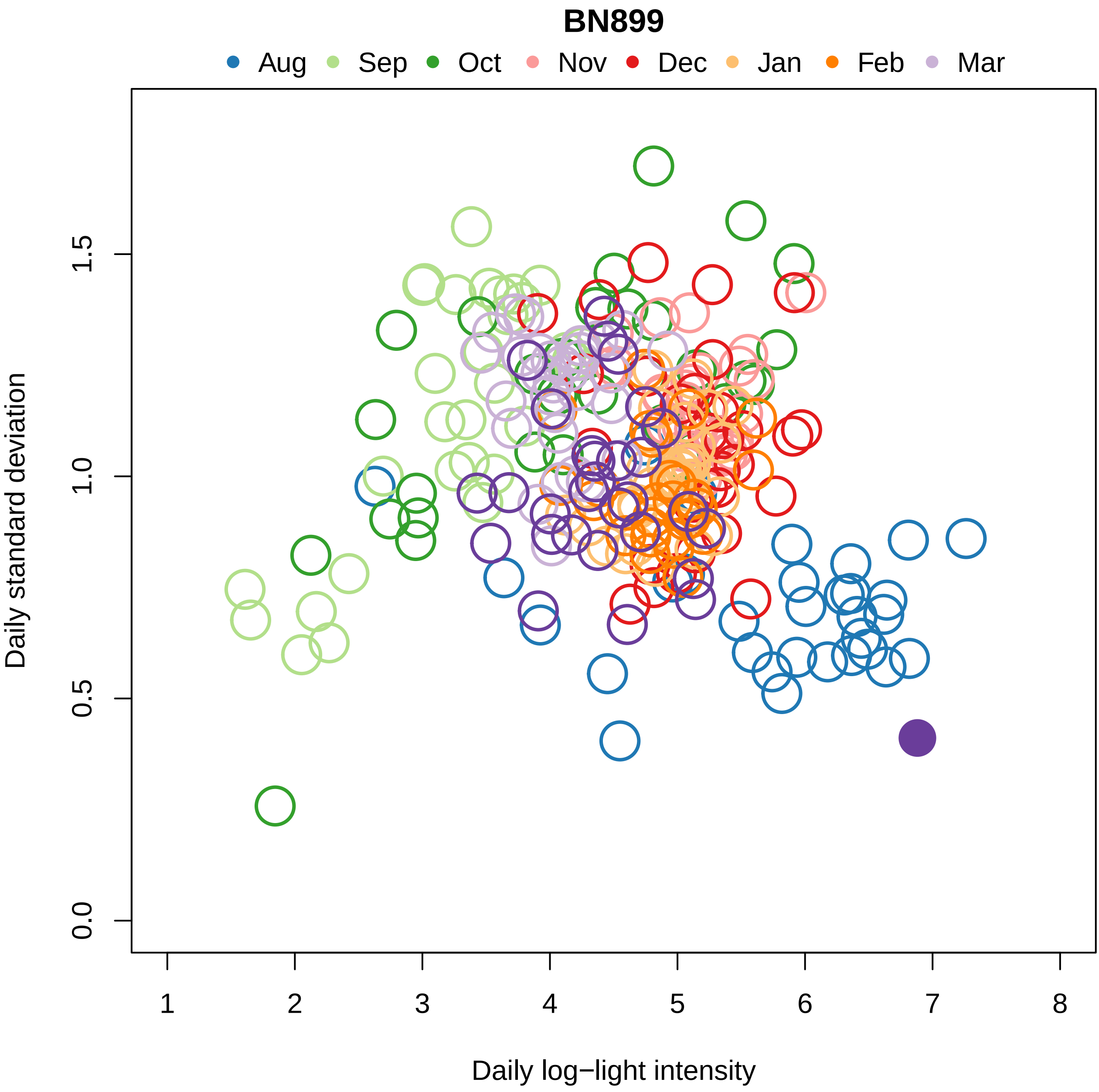
BN899
Aug
Sep
Oct
Nov
Dec
Jan
Feb
Mar
Daily standard deviation
0.0
0.5
1.0
1.5
1
2
3
4
5
6
7
8
Daily log−light intensity

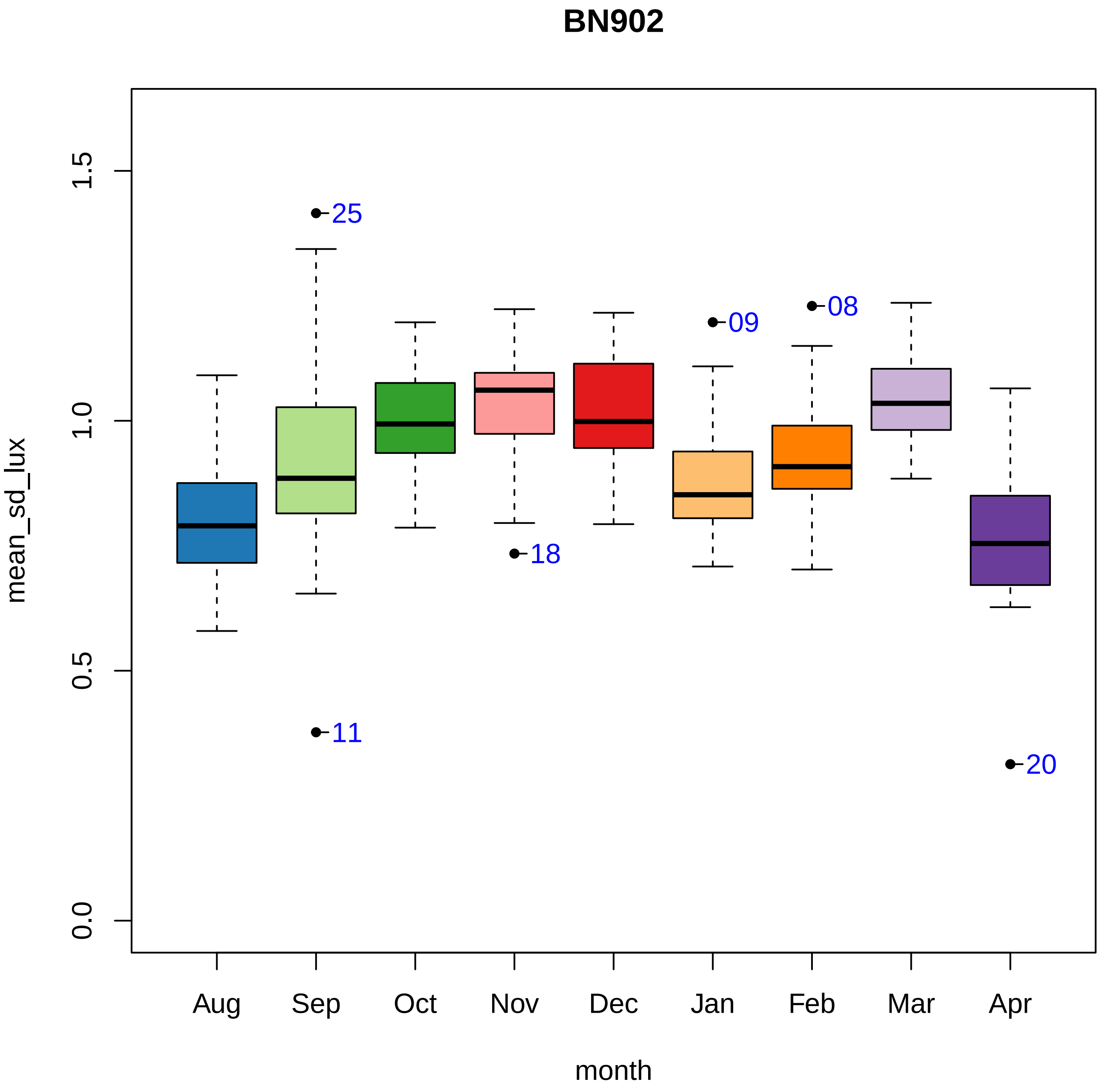

BN902
mean_sd_lux
0.0
0.5
1.0
1.5
month
Aug
Sep
Oct
Nov
Dec
Jan
Feb
Mar
Apr
25
11
18
09
08
20

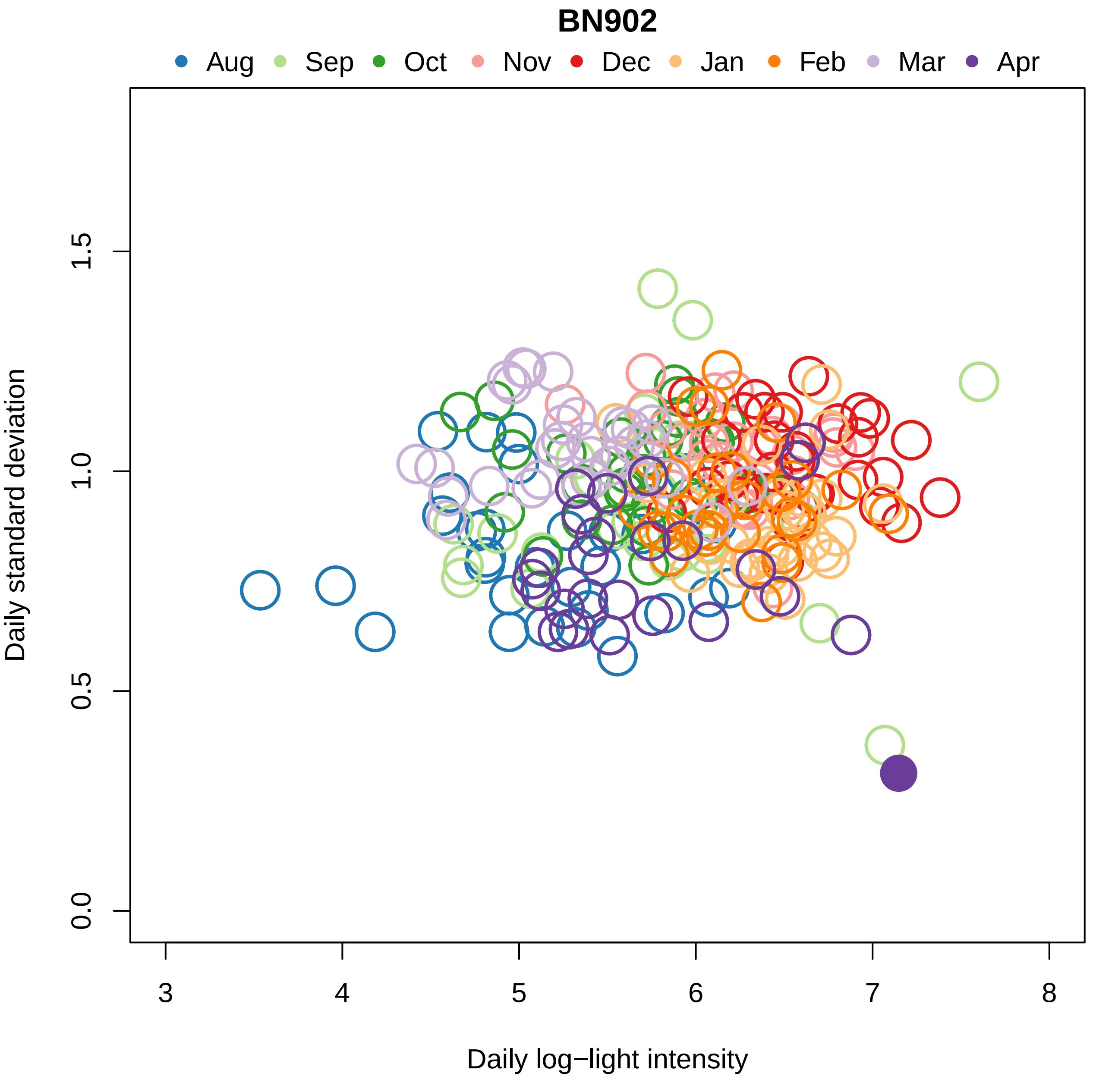
BN902
Aug
Sep
Oct
Nov
Dec
Jan
Feb
Mar
Apr
Daily standard deviation
0.0
0.5
1.0
1.5
3
4
5
6
7
8
Daily log−light intensity

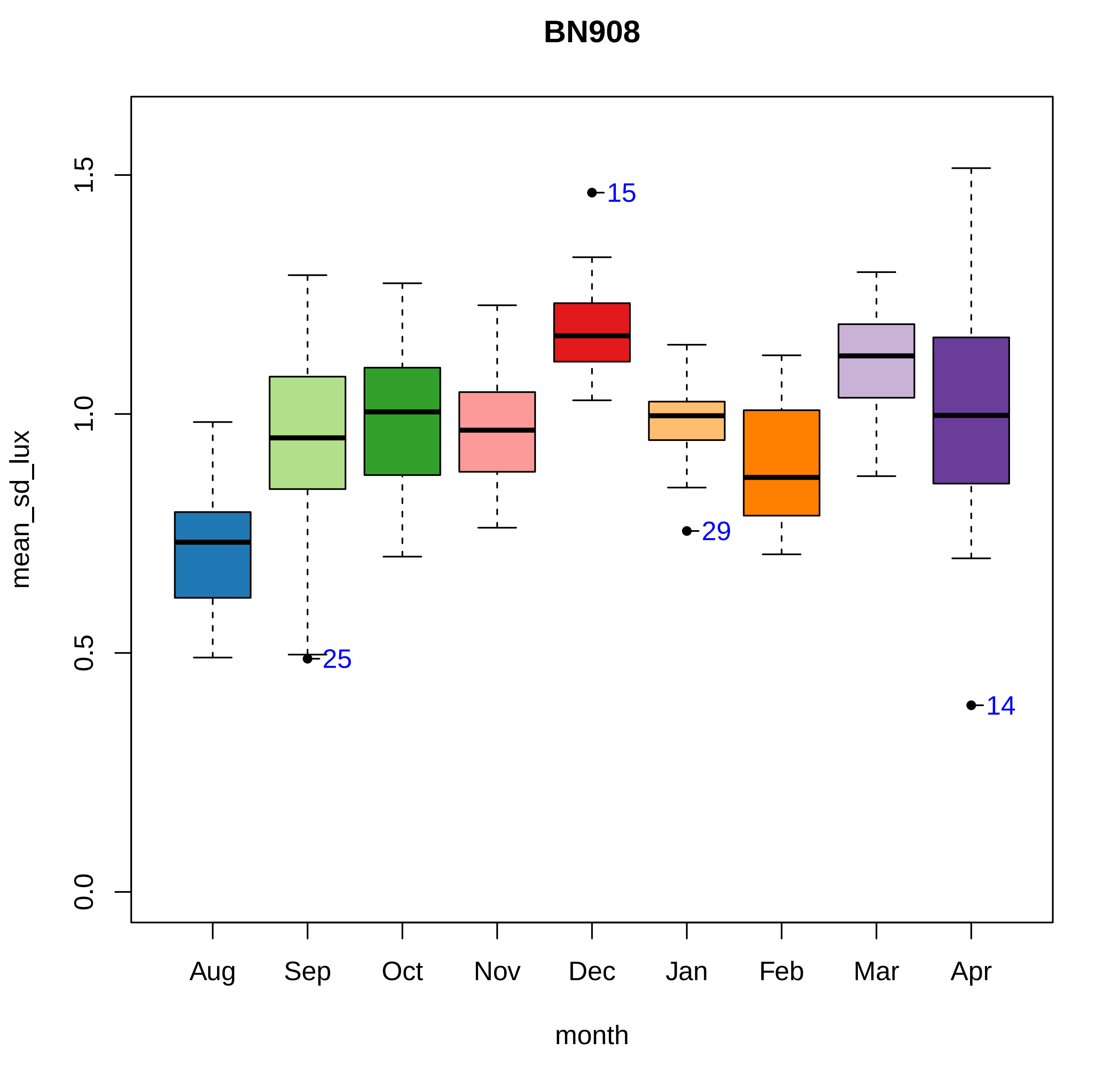
BN908
mean_sd_lux
0.0
0.5
1.0
1.5
month
Aug
Sep
Oct
Nov
Dec
Jan
Feb
Mar
Apr
15
25
29
14

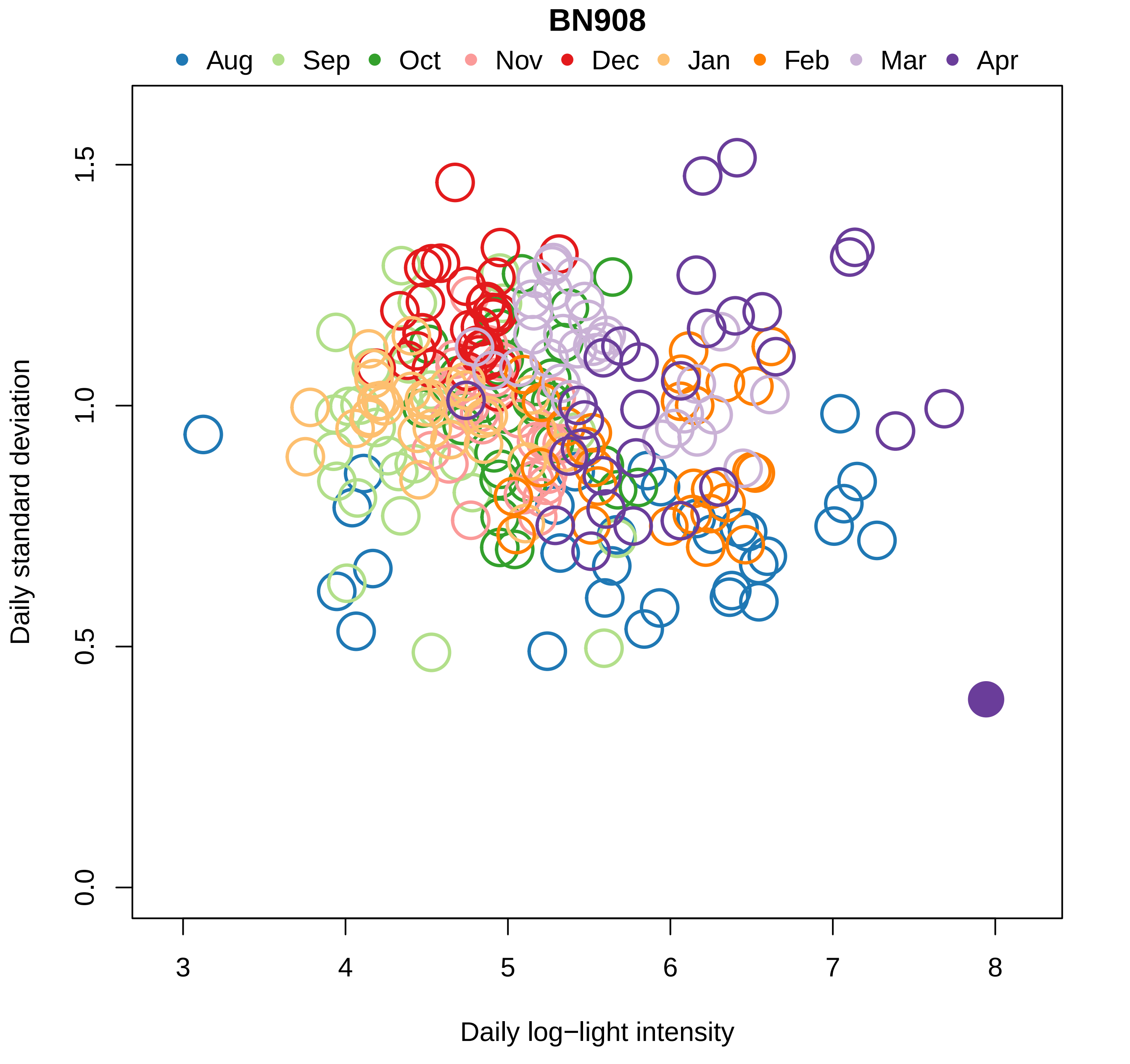
BN908
Aug
Sep
Oct
Nov
Dec
Jan
Feb
Mar
Apr
Daily standard deviation
0.0
0.5
1.0
1.5
Daily log−light intensity
3
4
5
6
7
8

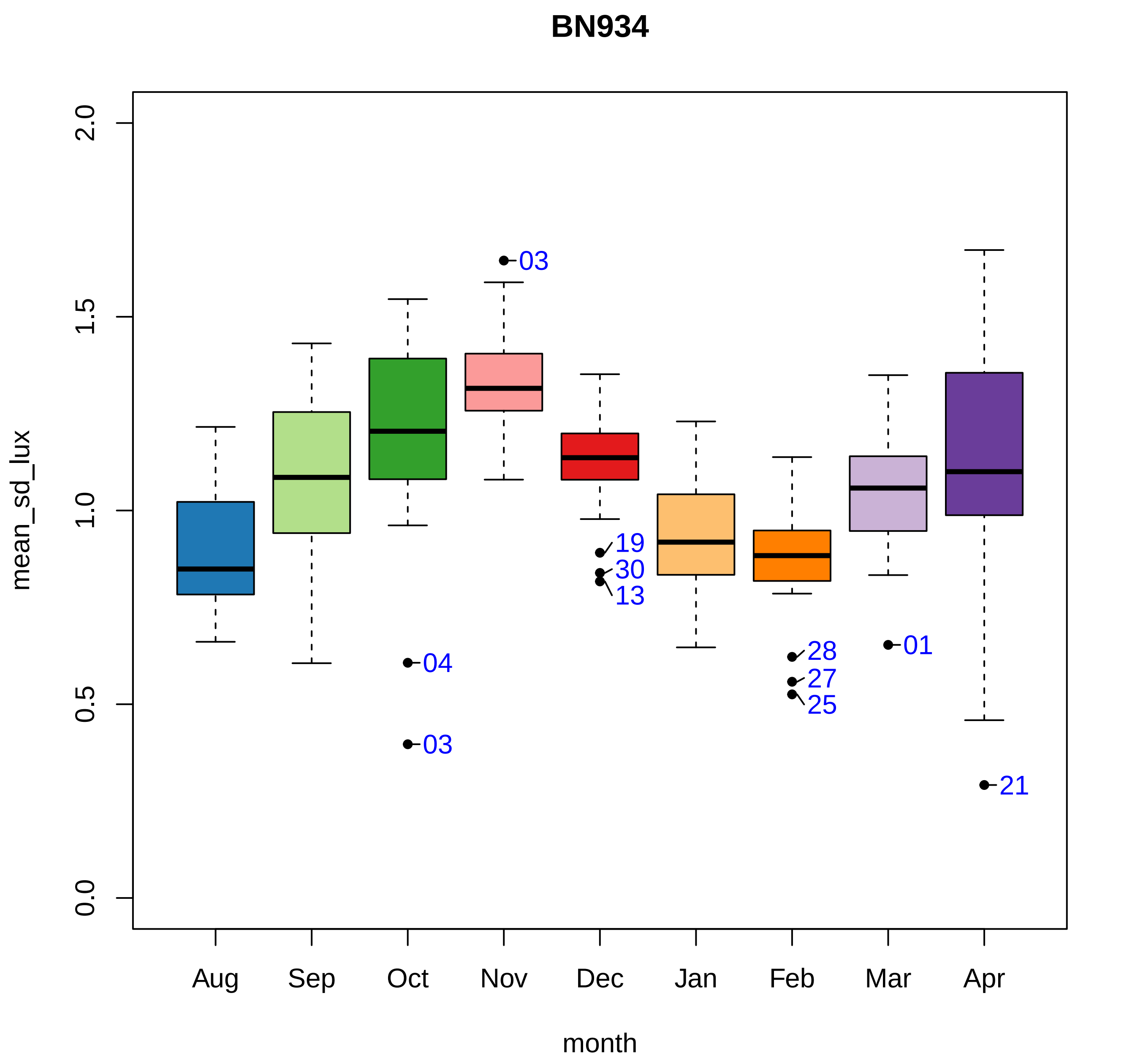

BN934
mean_sd_lux
month
0.0
0.5
1.0
1.5
2.0
Aug
Sep
Oct
Nov
Dec
Jan
Feb
Mar
Apr
03
04
03
19
30
13
28
27
25
01
21

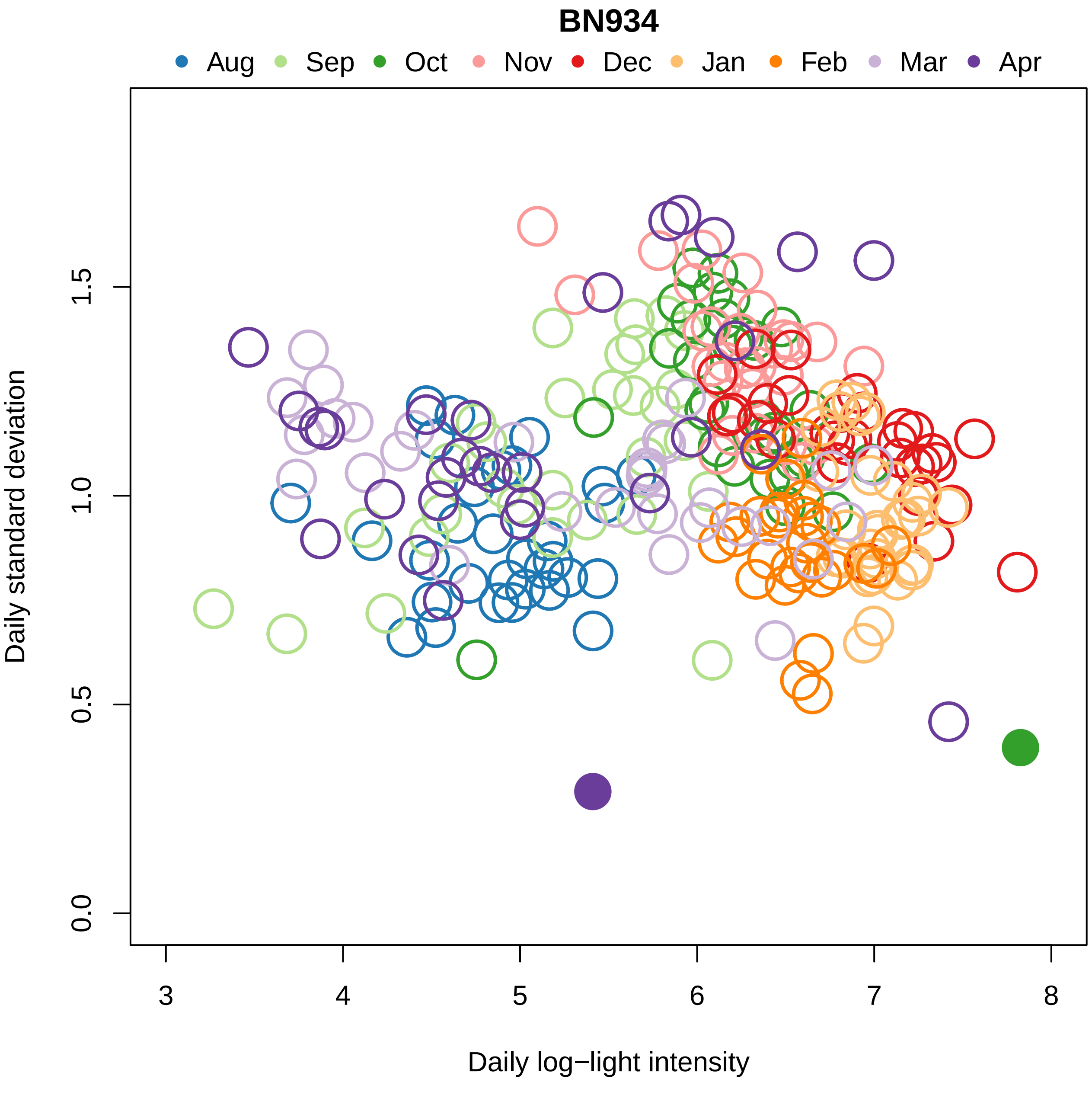

BN934
Aug
Sep
Oct
Nov
Dec
Jan
Feb
Mar
Apr
Daily standard deviation
0.0
0.5
1.0
1.5
3
4
5
6
7
8
Daily log−light intensity

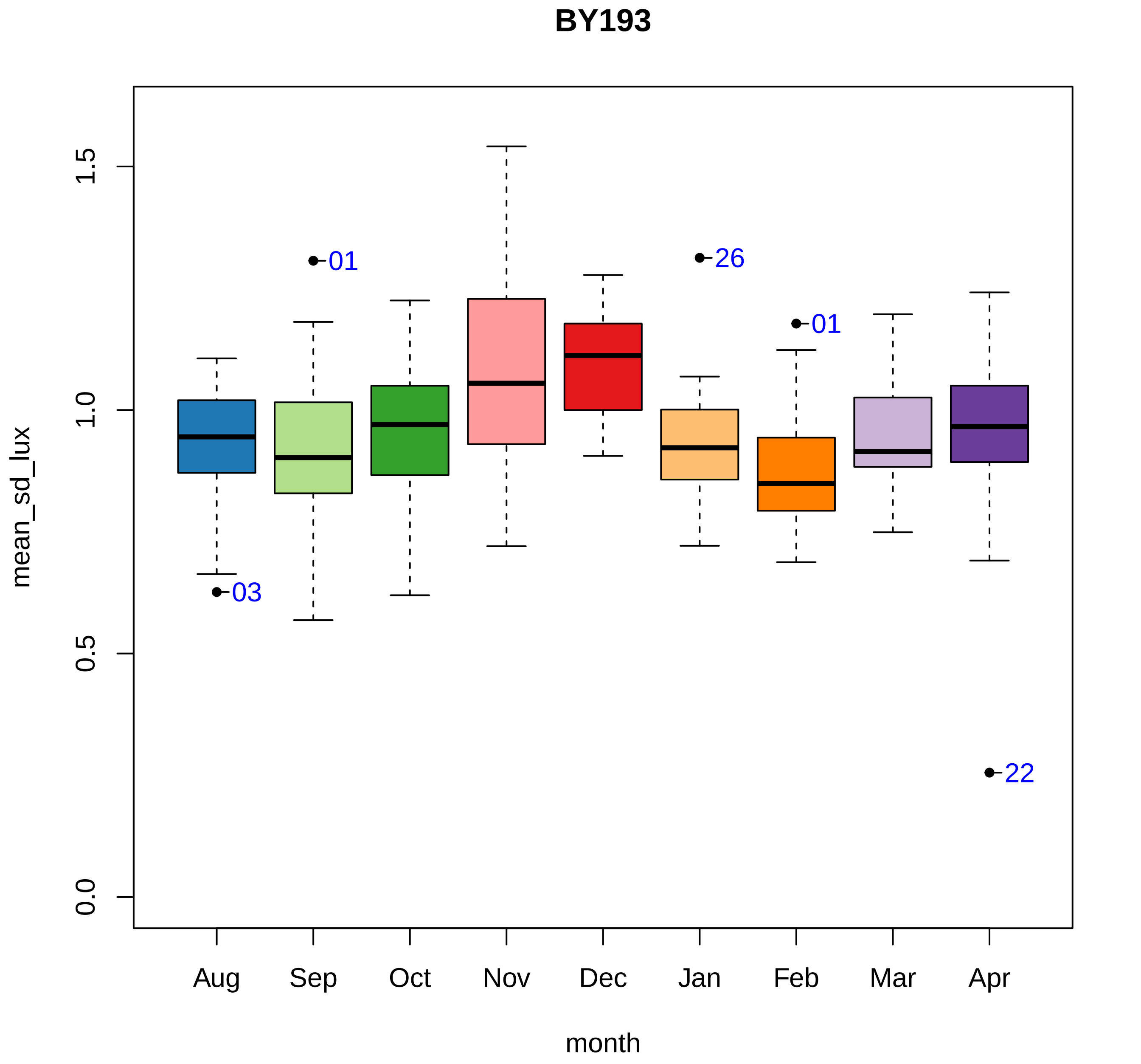

BY193
mean_sd_lux
0.0
0.5
1.0
1.5
01
26
01
03
22
Aug
Sep
Oct
Nov
Dec
Jan
Feb
Mar
Apr
month

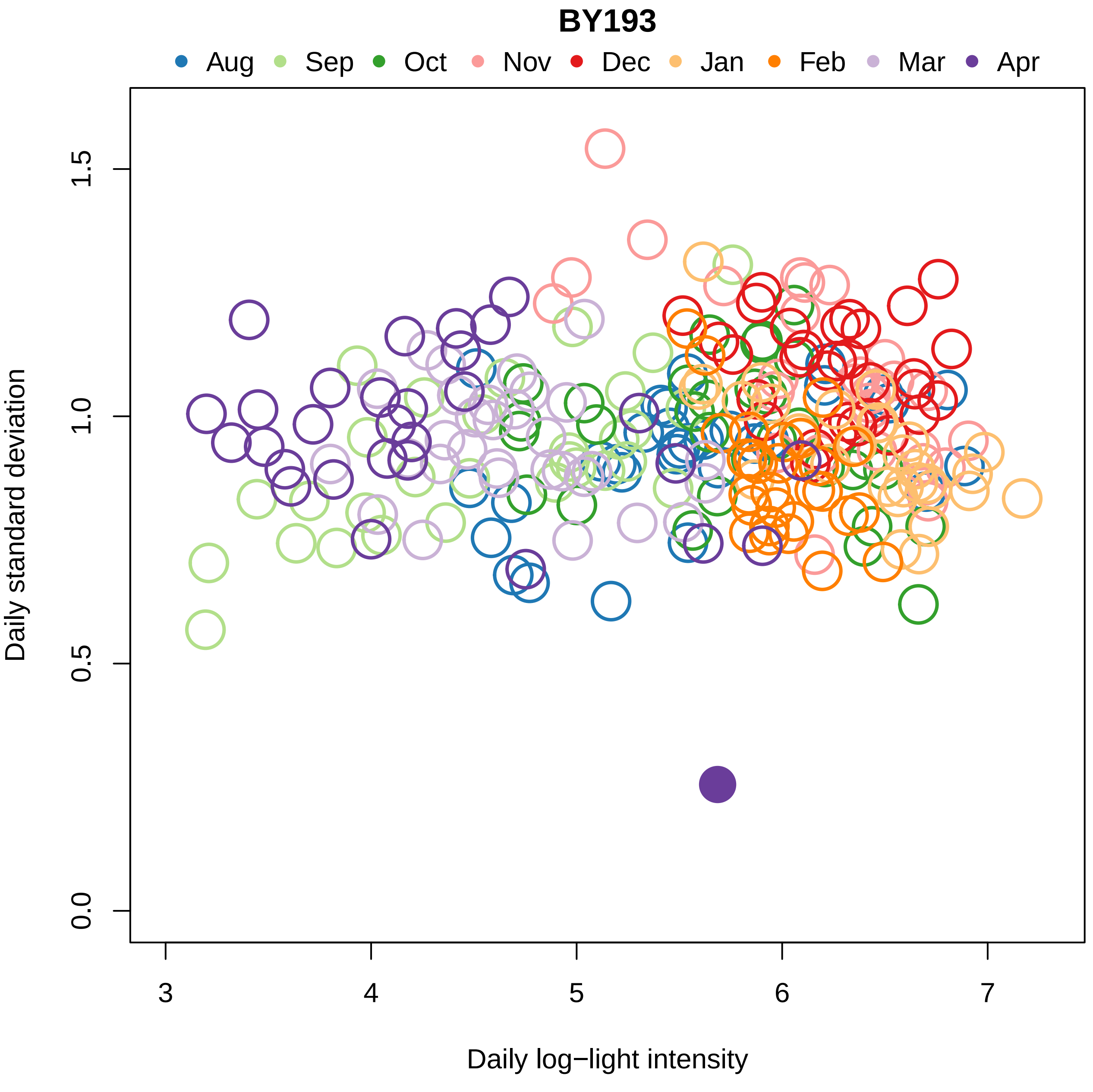
BY193
Aug
Sep
Oct
Nov
Dec
Jan
Feb
Mar
Apr
Daily standard deviation
0.0
0.5
1.0
1.5
3
4
5
6
7
Daily log−light intensity

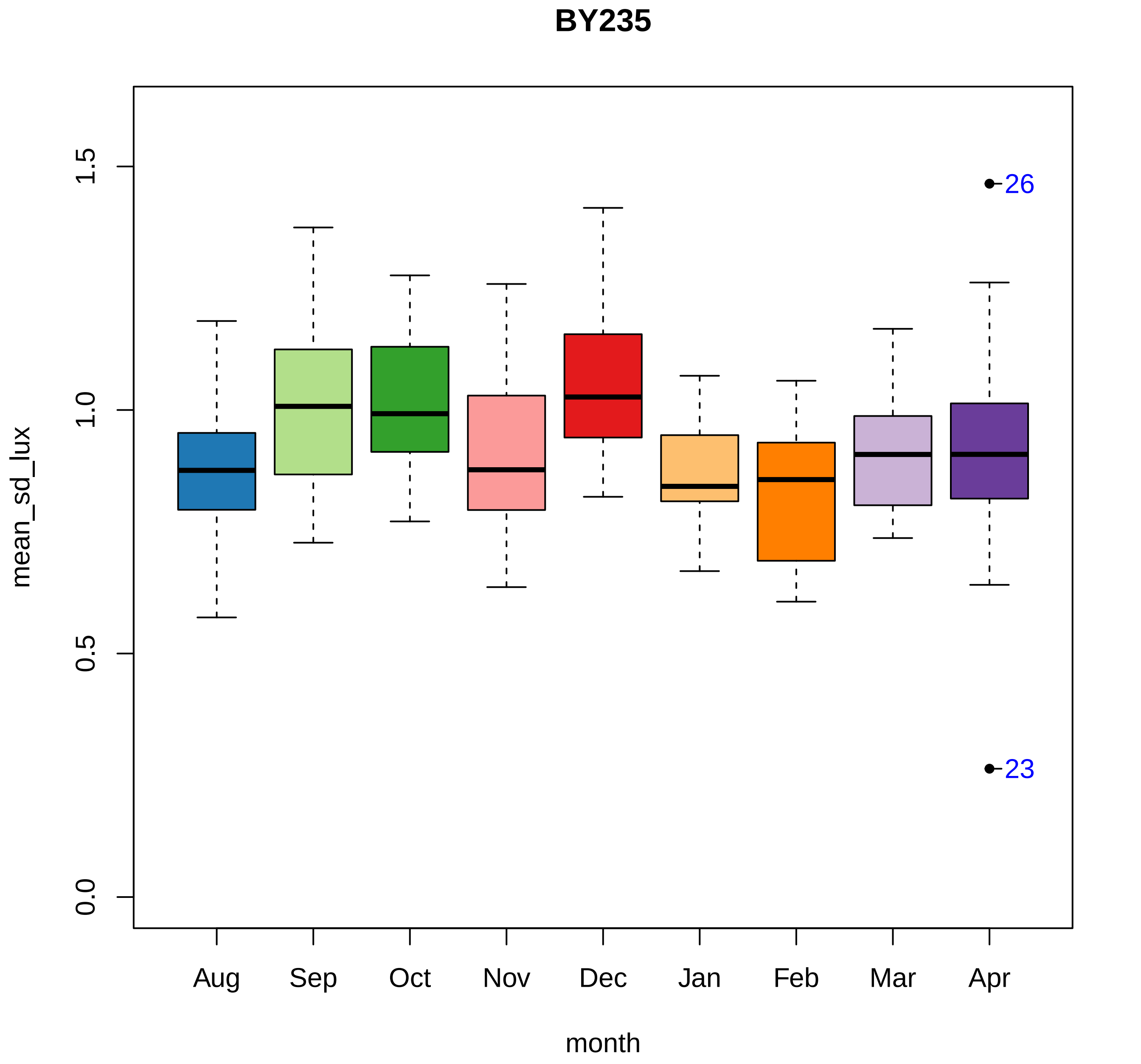

BY235
mean_sd_lux
0.0
0.5
1.0
1.5
26
23
Aug
Sep
Oct
Nov
Dec
Jan
Feb
Mar
Apr
month

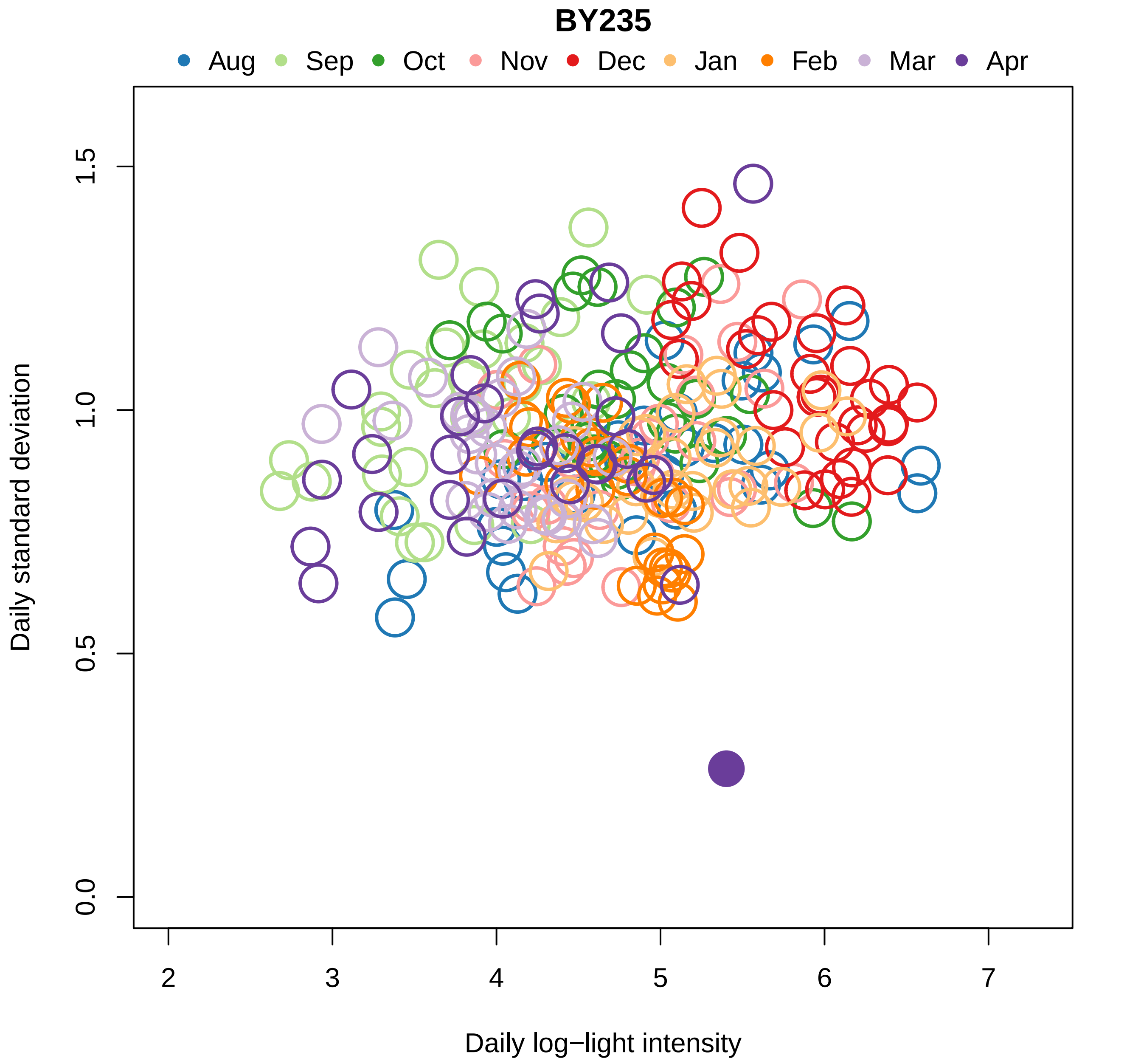
BY235
Aug
Sep
Oct
Nov
Dec
Jan
Feb
Mar
Apr
Daily standard deviation
0.0
0.5
1.0
1.5
Daily log−light intensity
2
3
4
5
6
7

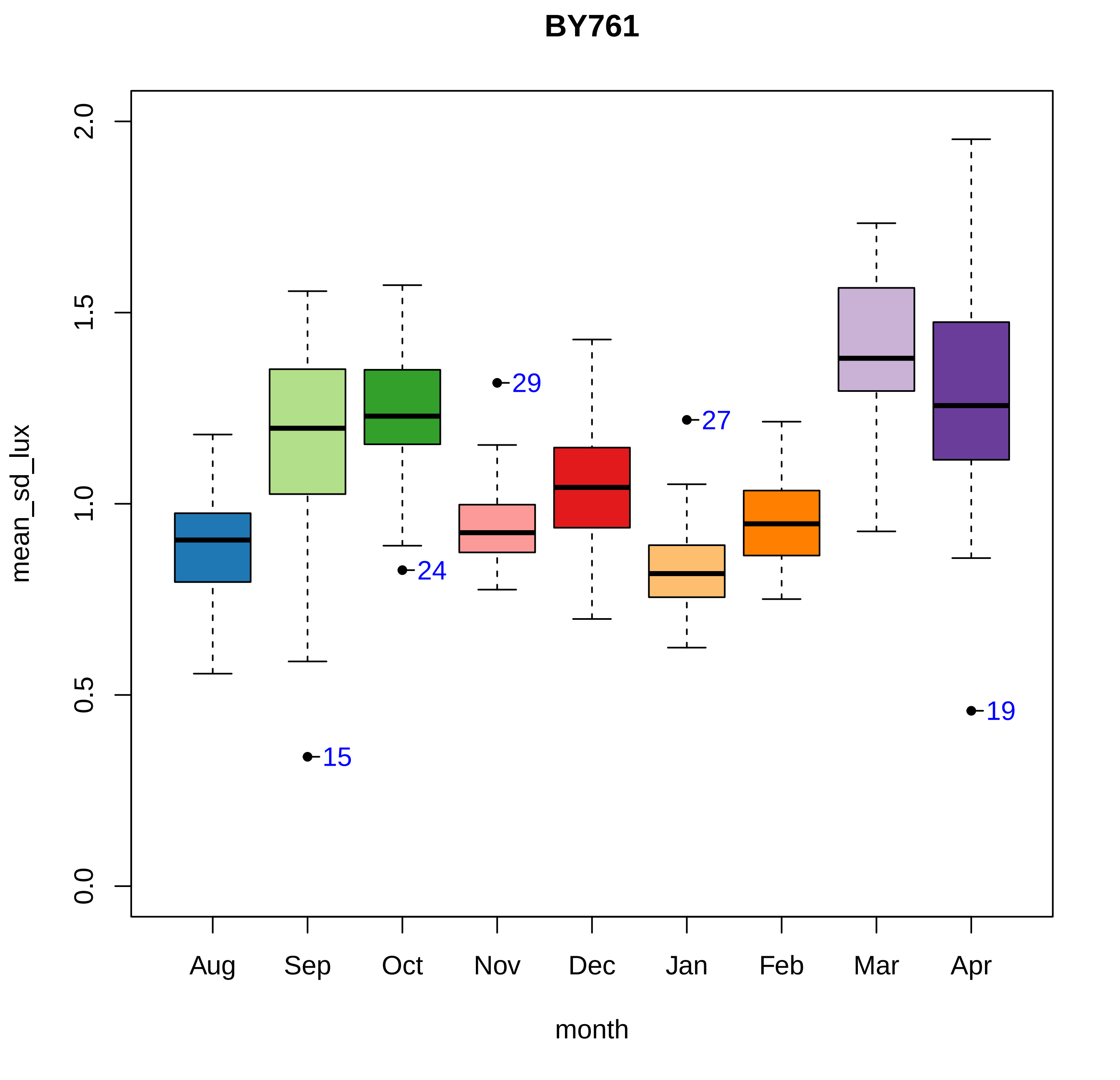
BY761
mean_sd_lux
month
0.0
0.5
1.0
1.5
2.0
Aug
Sep
Oct
Nov
Dec
Jan
Feb
Mar
Apr
15
24
29
27
19

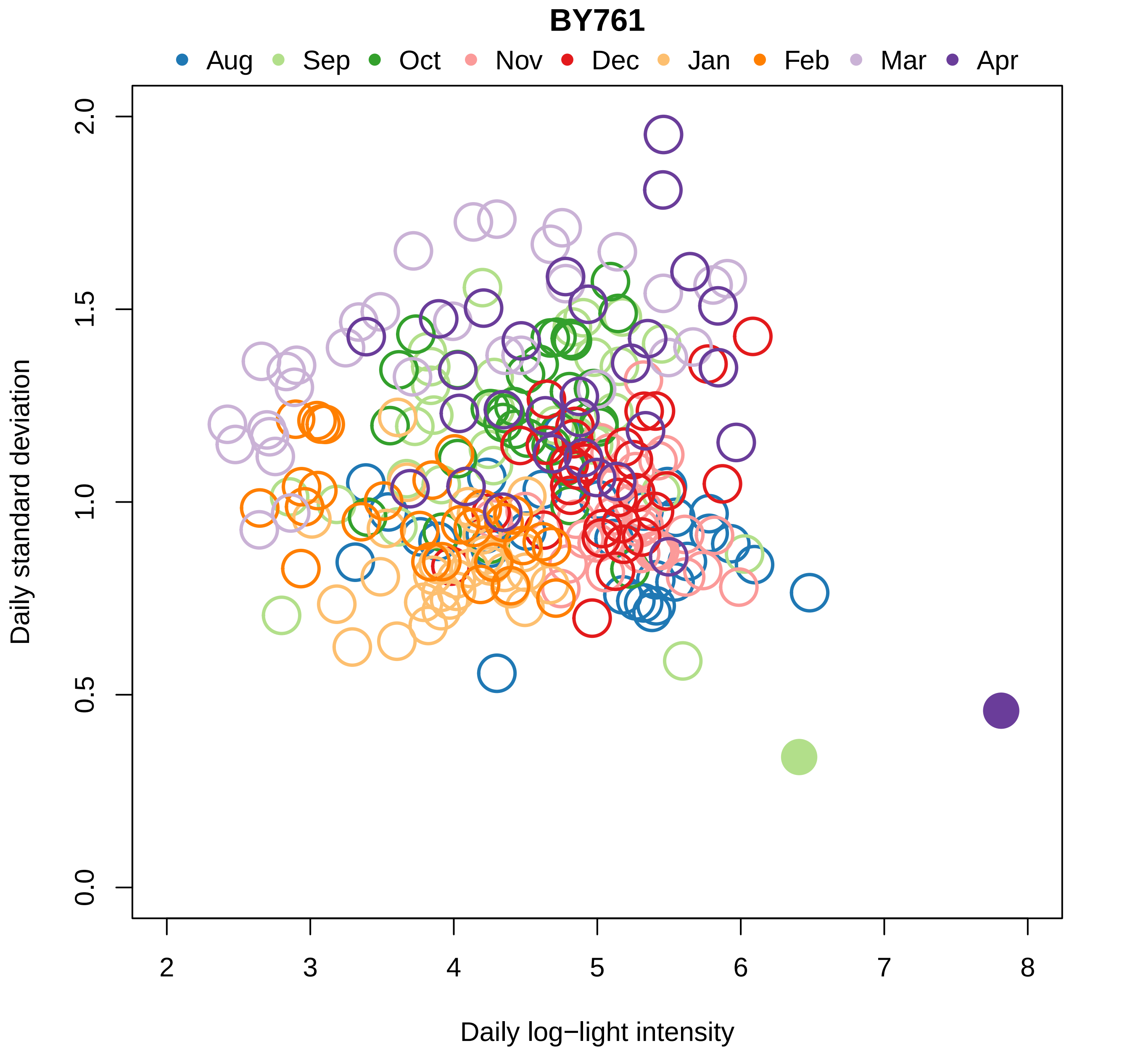

BY761
Aug
Sep
Oct
Nov
Dec
Jan
Feb
Mar
Apr
Daily standard deviation
0.0
0.5
1.0
1.5
2.0
2
3
4
5
6
7
8
Daily log−light intensity

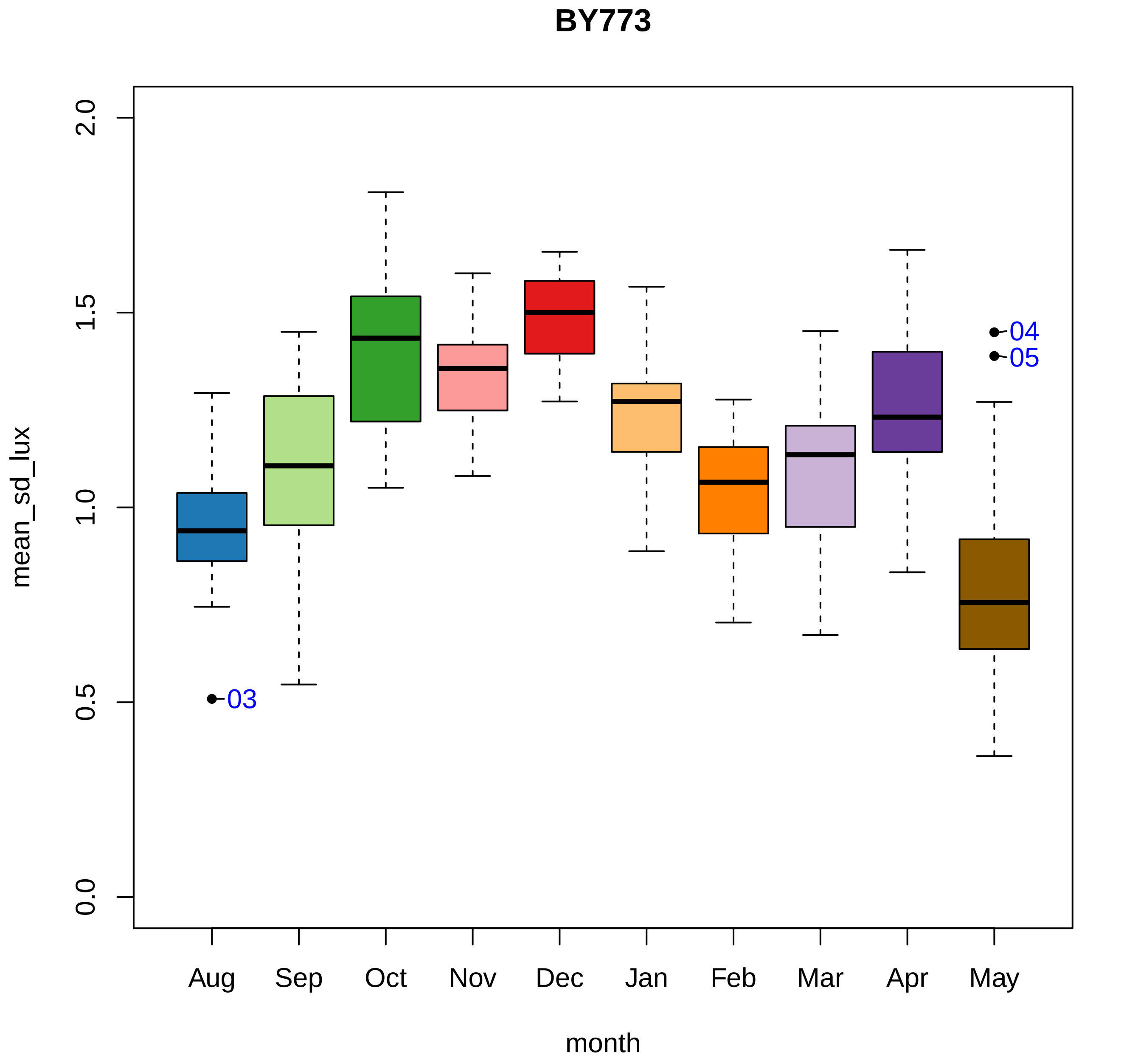
BY773
mean_sd_lux
0.0
0.5
1.0
1.5
2.0
03
04
05
Aug
Sep
Oct
Nov
Dec
Jan
Feb
Mar
Apr
May
month

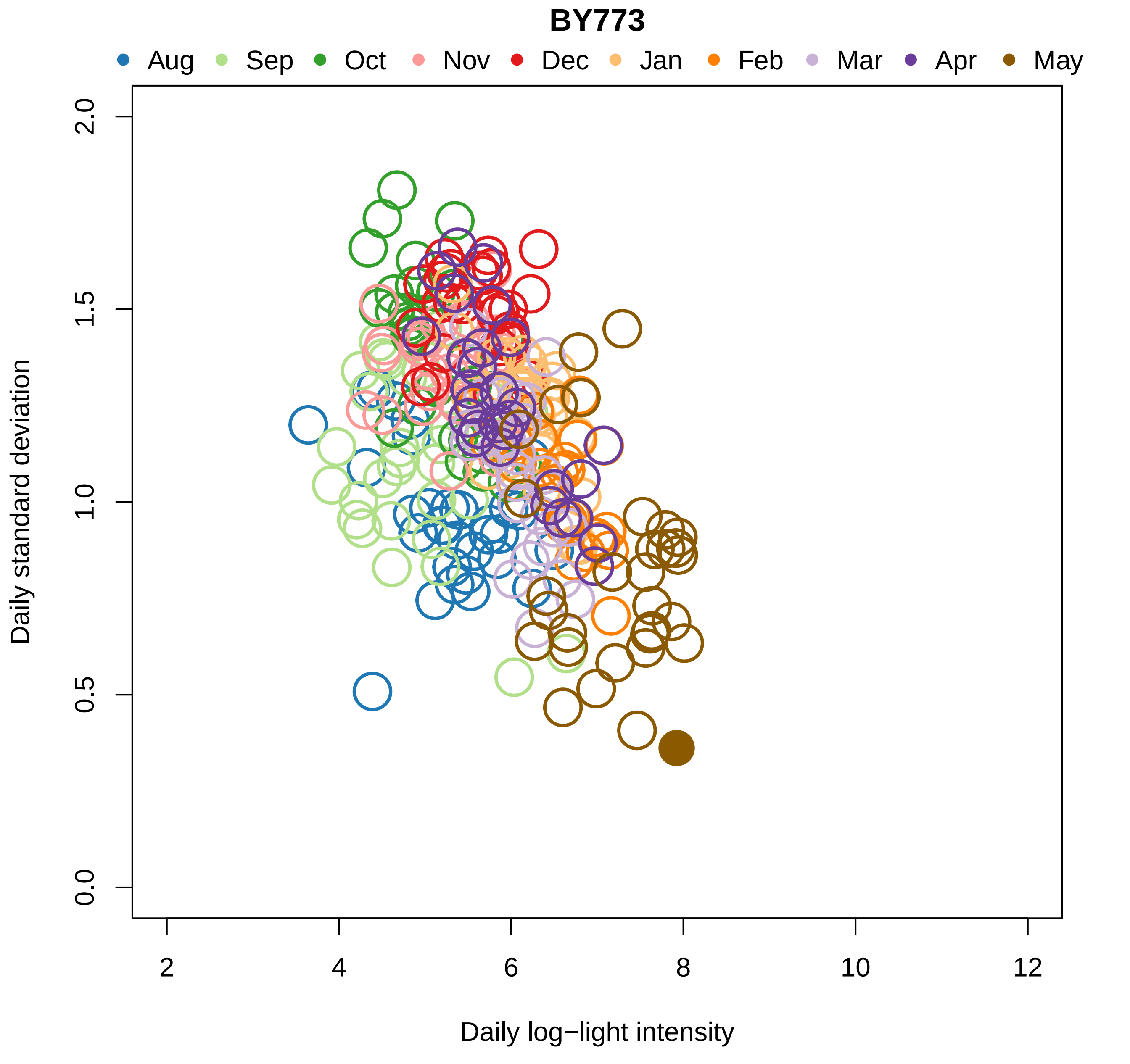
BY773
Aug
Sep
Oct
Nov
Dec
Jan
Feb
Mar
Apr
May
Daily standard deviation
0.0
0.5
1.0
1.5
2.0
2
4
6
8
10
12
Daily log−light intensity

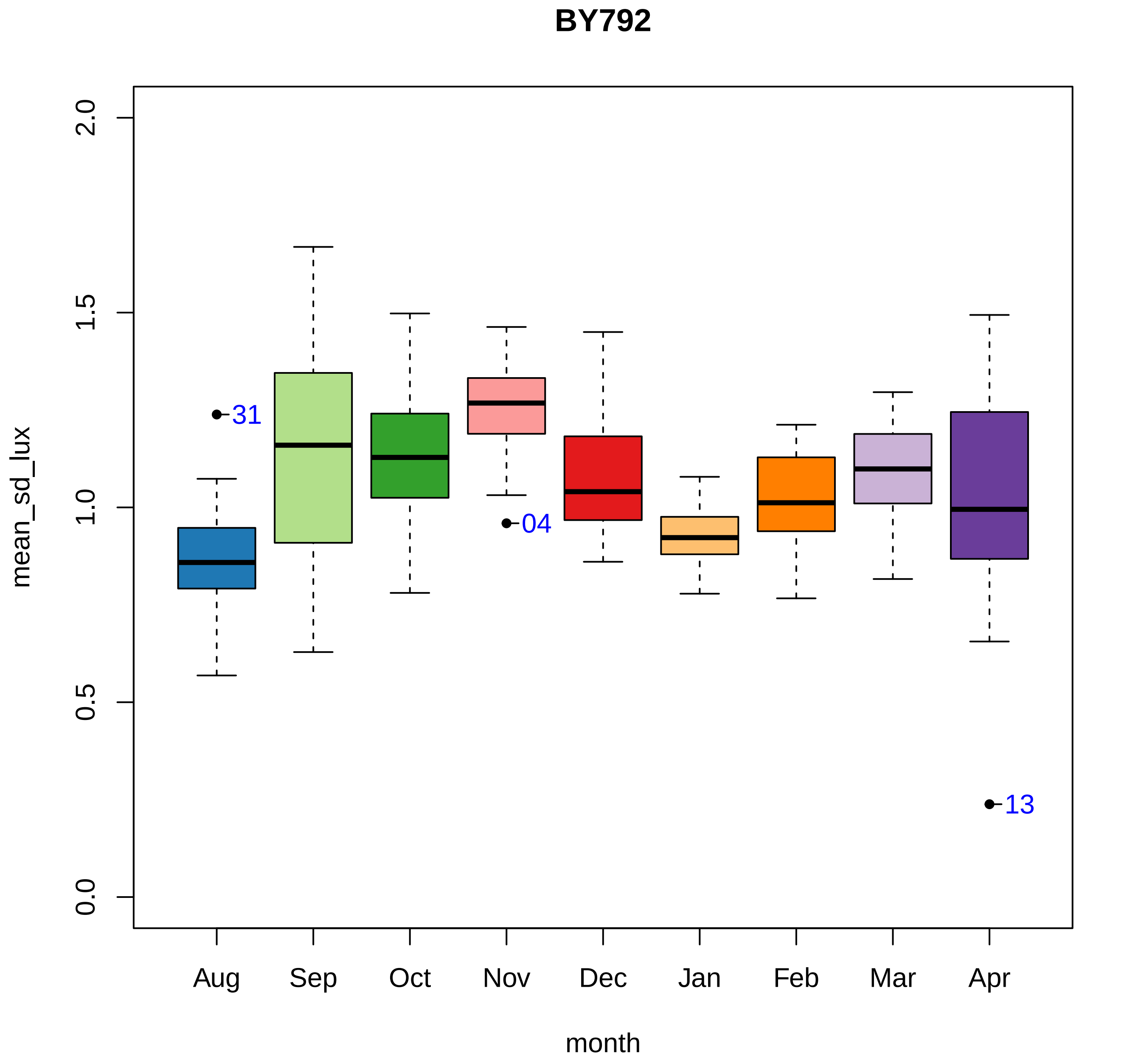
BY792
mean_sd_lux
month
0.0
0.5
1.0
1.5
2.0
Aug
Sep
Oct
Nov
Dec
Jan
Feb
Mar
Apr
31
04
13

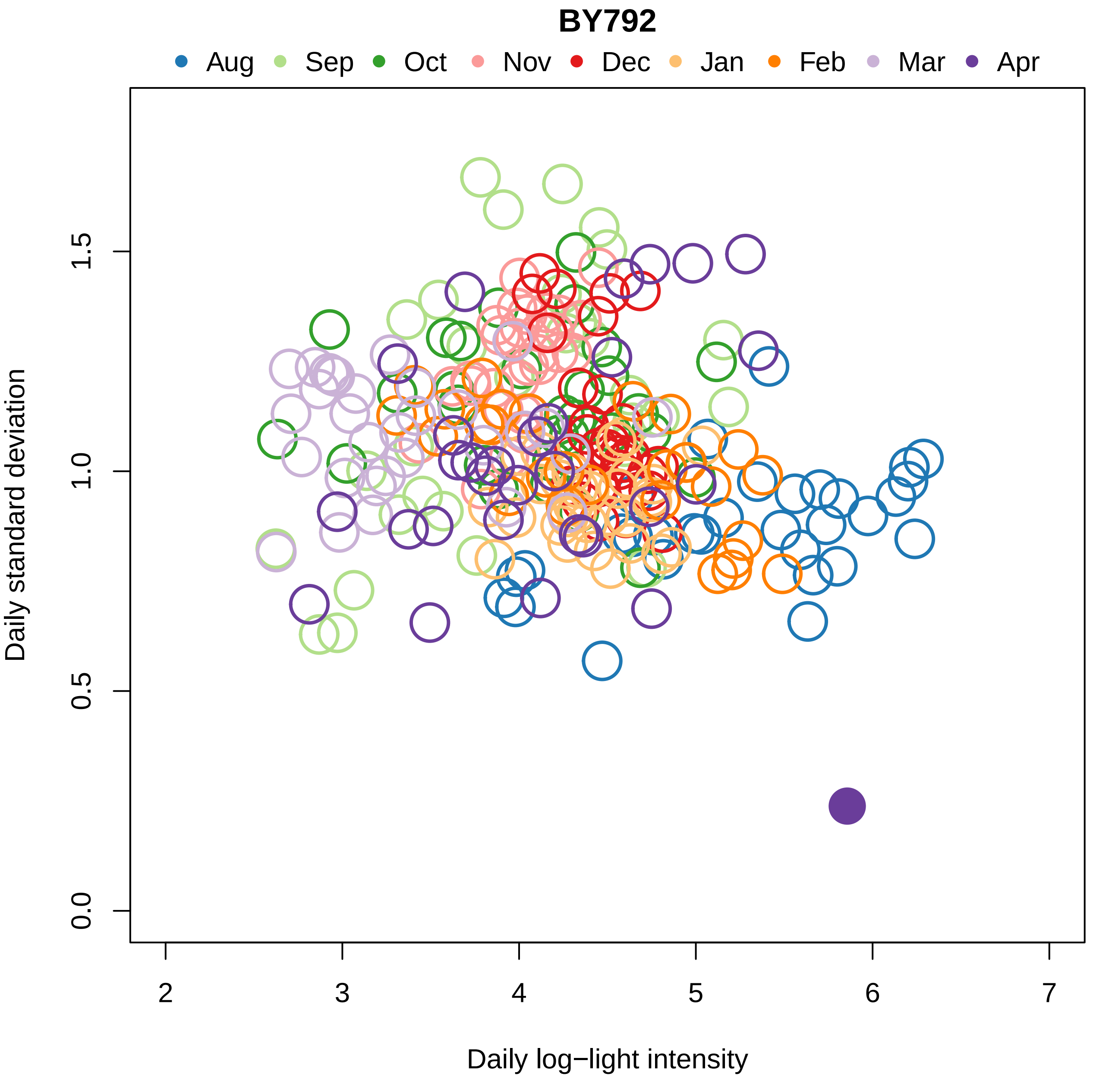

BY792
Aug
Sep
Oct
Nov
Dec
Jan
Feb
Mar
Apr
Daily standard deviation
0.0
0.5
1.0
1.5
2
3
4
5
6
7
Daily log−light intensity

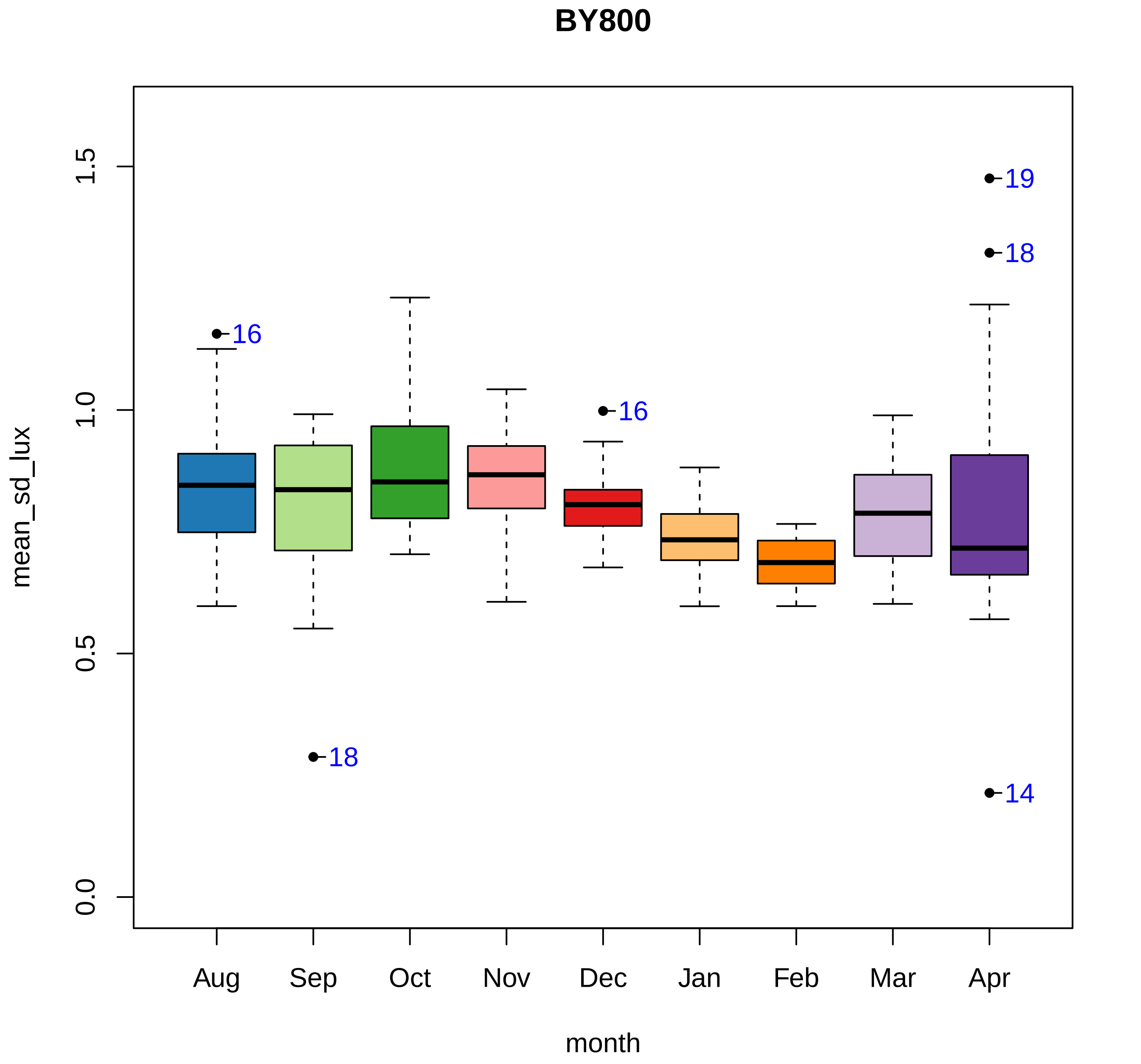

BY800
mean_sd_lux
0.0
0.5
1.0
1.5
16
18
16
19
18
14
Aug
Sep
Oct
Nov
Dec
Jan
Feb
Mar
Apr
month

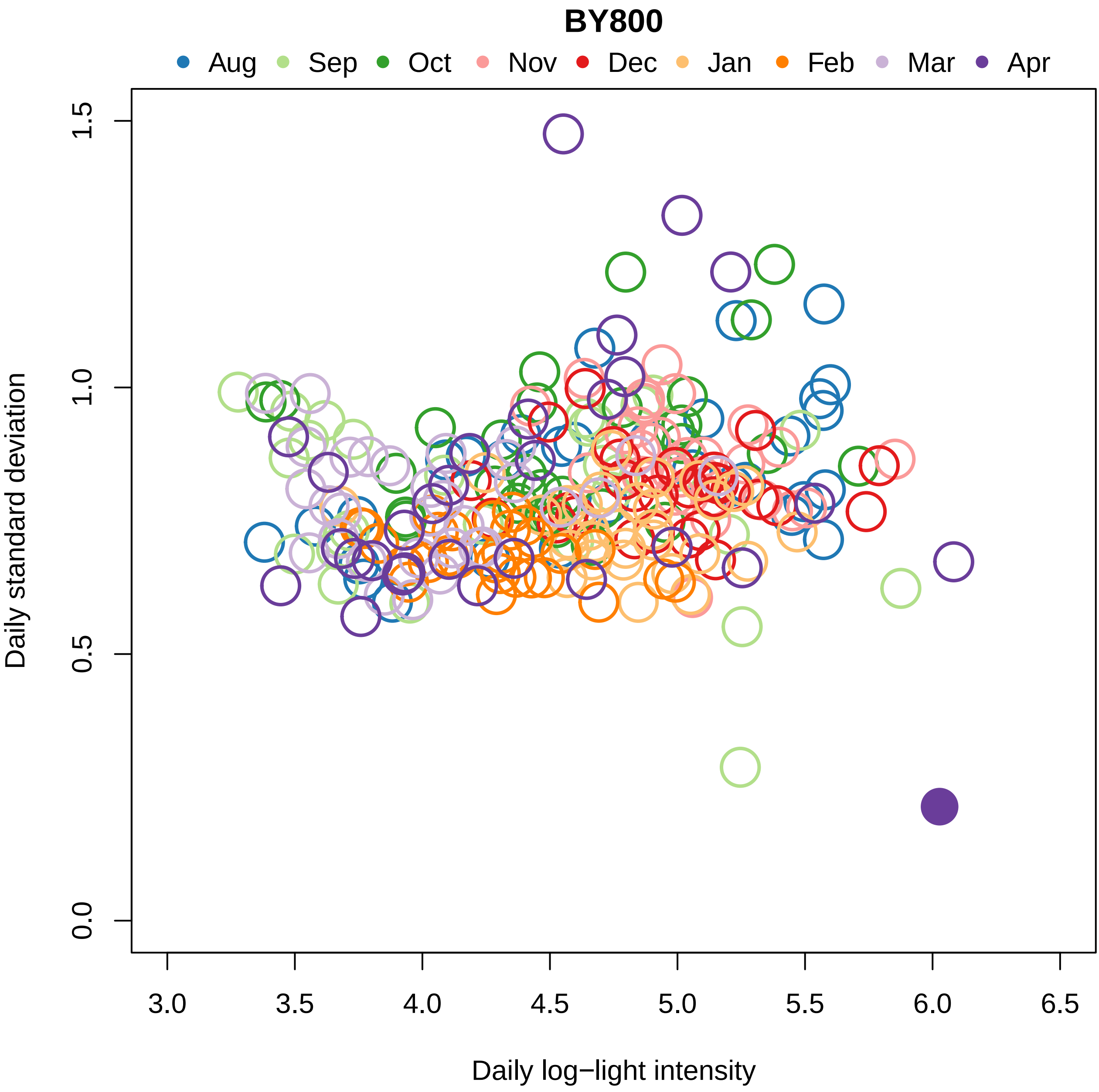

BY800
Aug
Sep
Oct
Nov
Dec
Jan
Feb
Mar
Apr
Daily standard deviation
0.0
0.5
1.0
1.5
3.0
3.5
4.0
4.5
5.0
5.5
6.0
6.5
Daily log−light intensity

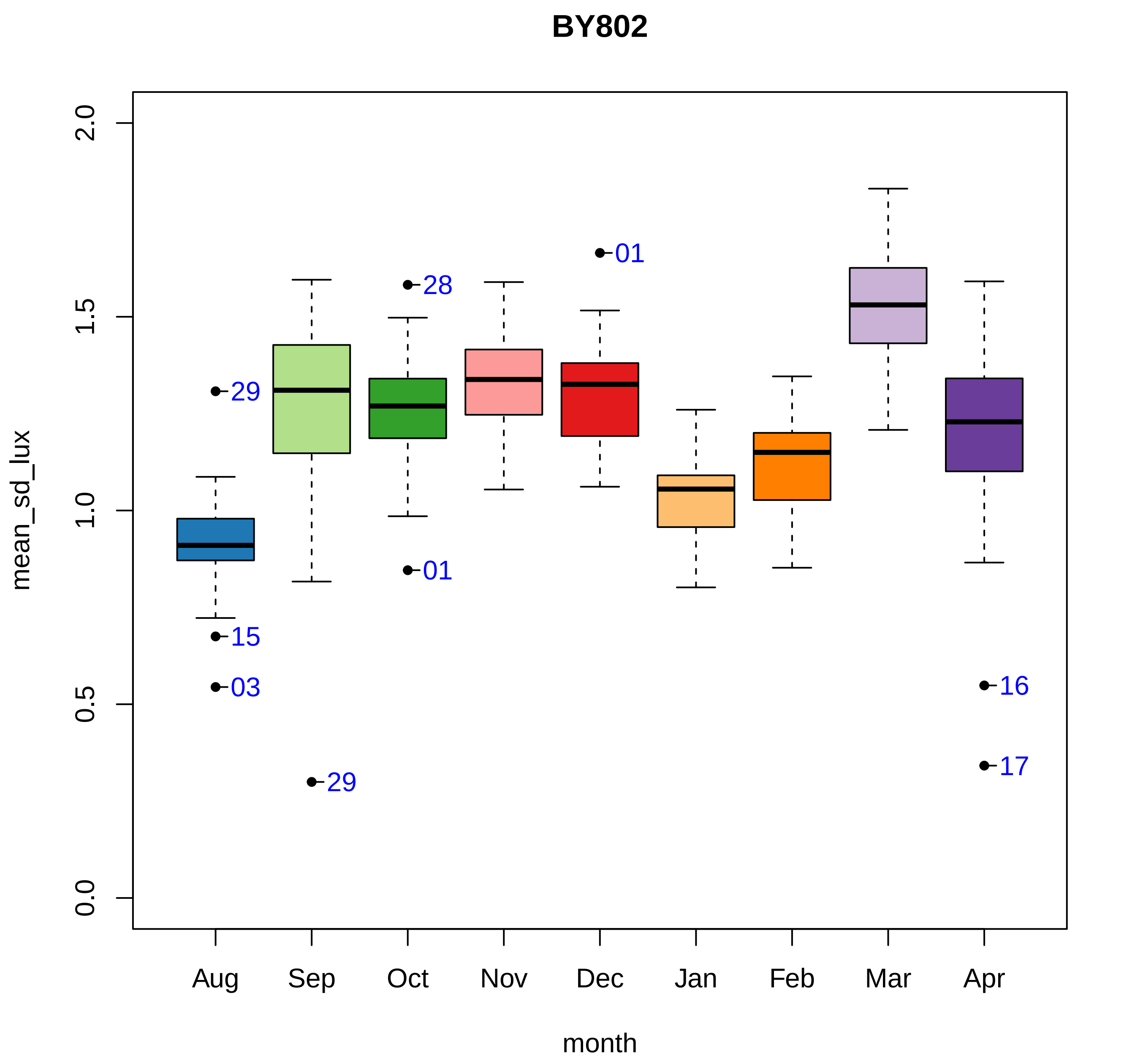
BY802
mean_sd_lux
month
0.0
0.5
1.0
1.5
2.0
Aug
Sep
Oct
Nov
Dec
Jan
Feb
Mar
Apr
29
15
03
29
28
01
01
16
17

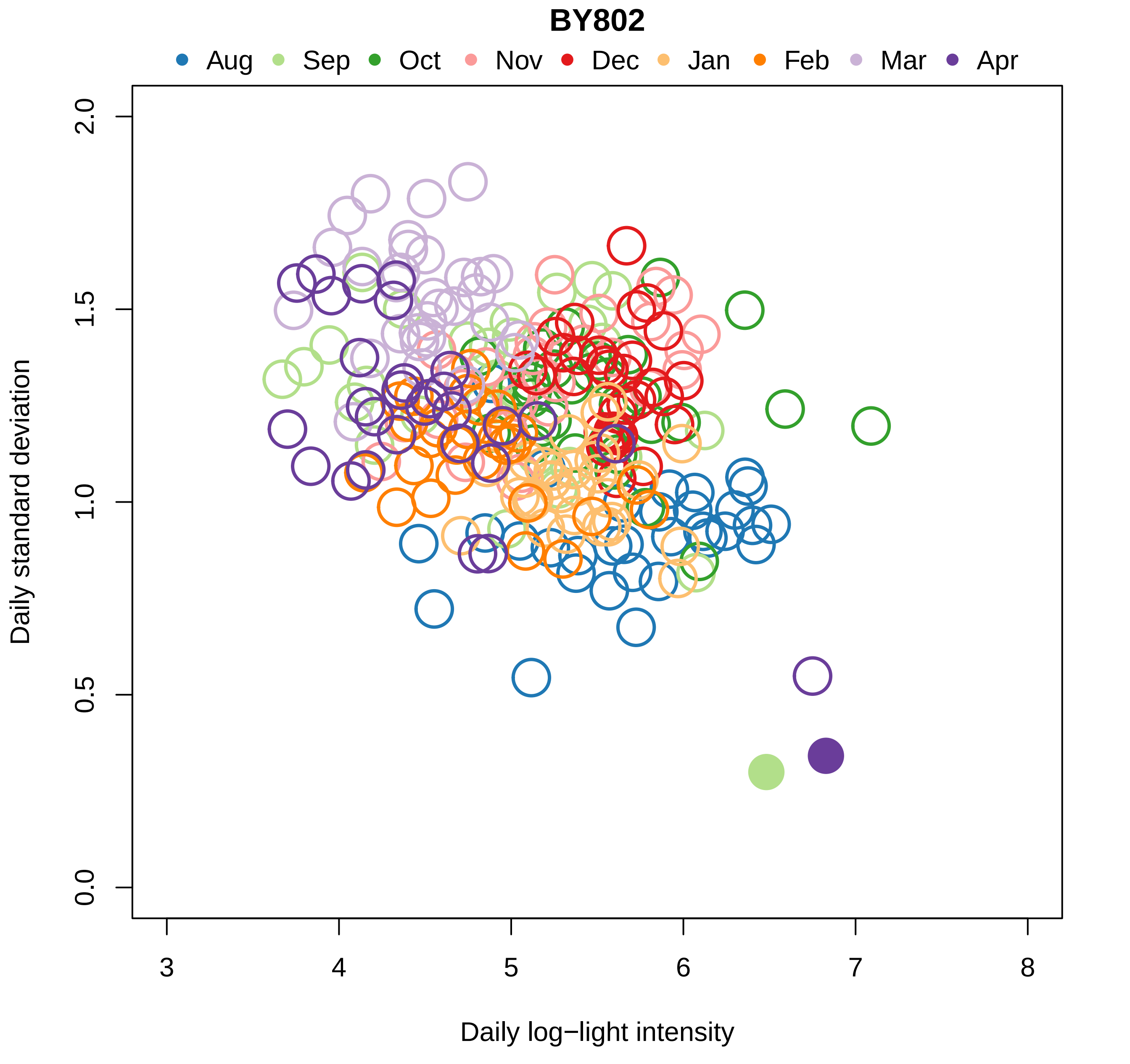

BY802
Aug
Sep
Oct
Nov
Dec
Jan
Feb
Mar
Apr
Daily standard deviation
0.0
0.5
1.0
1.5
2.0
3
4
5
6
7
8
Daily log−light intensity

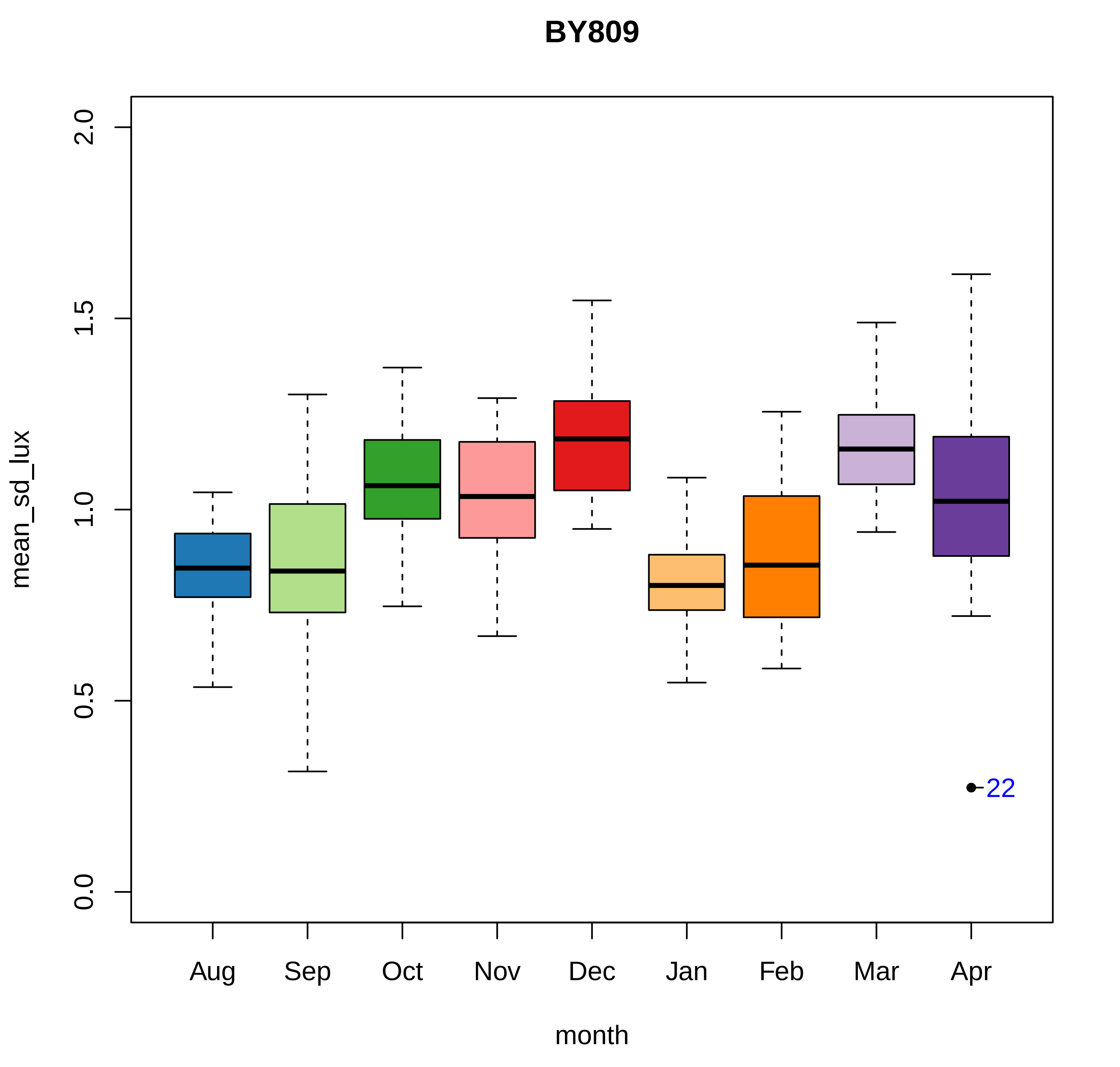
BY809
mean_sd_lux
0.0
0.5
1.0
1.5
2.0
Aug
Sep
Oct
Nov
Dec
Jan
Feb
Mar
Apr
month
22

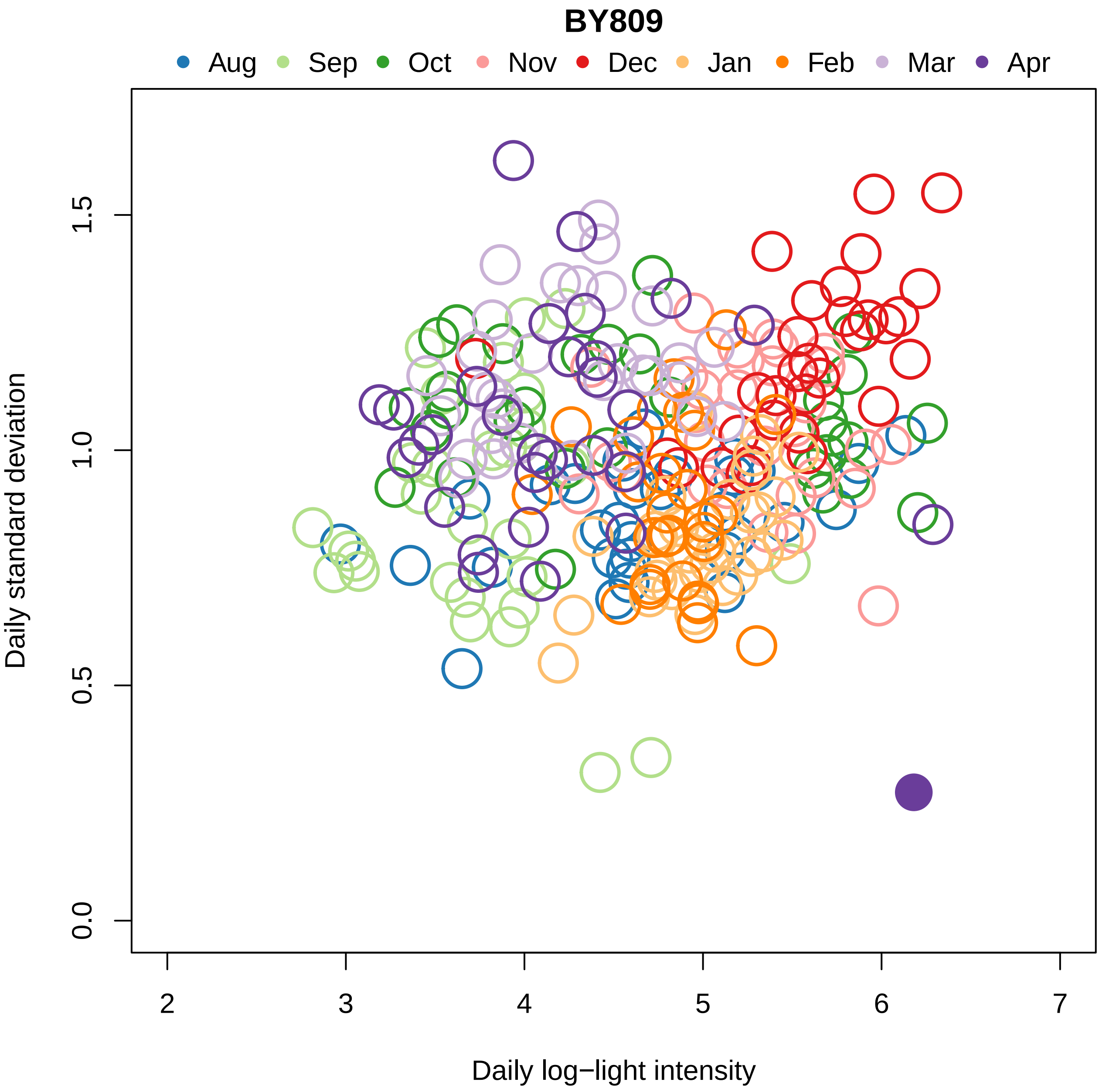
BY809
Aug
Sep
Oct
Nov
Dec
Jan
Feb
Mar
Apr
Daily standard deviation
0.0
0.5
1.0
1.5
2
3
4
5
6
7
Daily log−light intensity

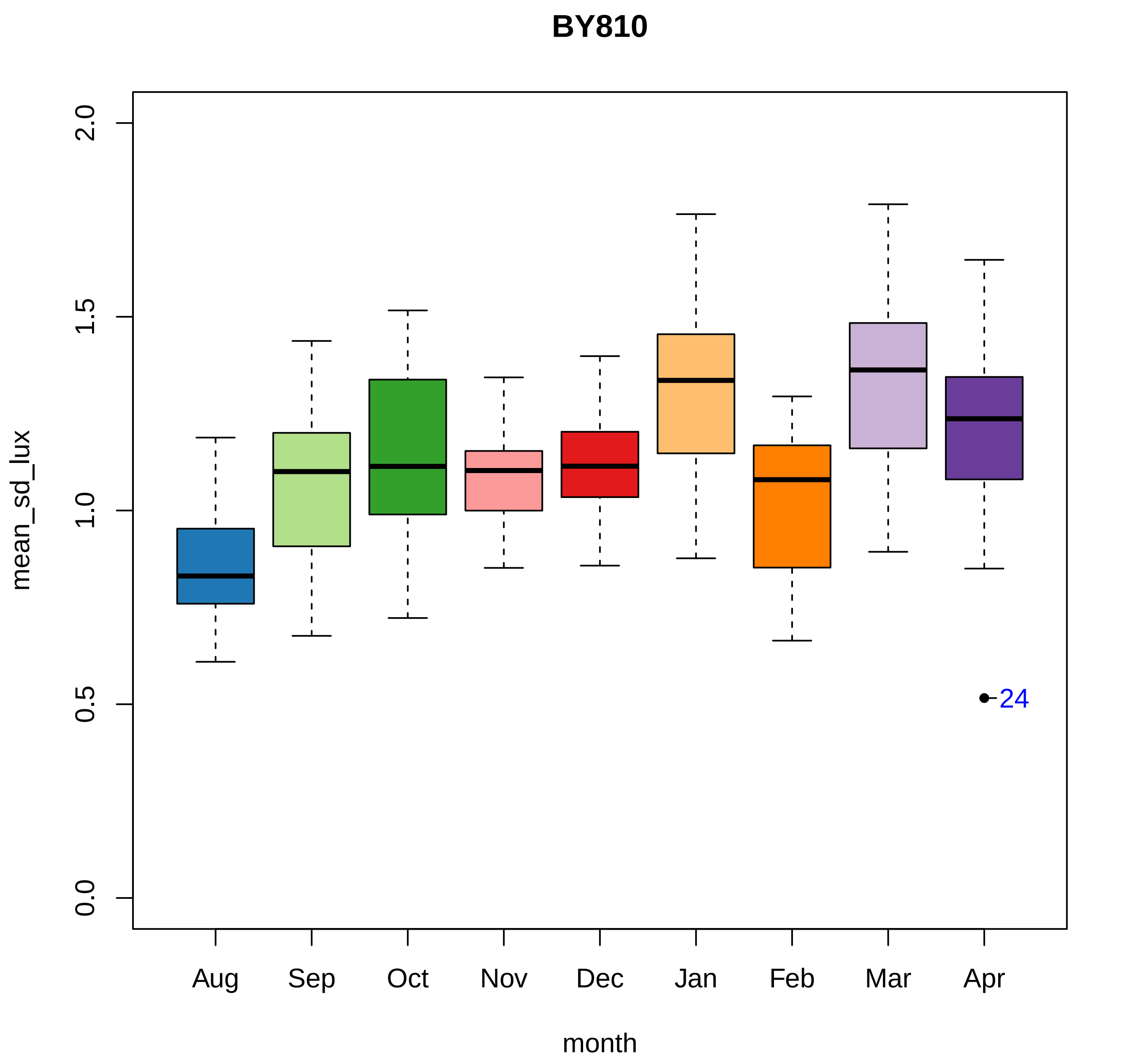
BY810
mean_sd_lux
2.0
1.5
1.0
0.5
0.0
24
Aug
Sep
Oct
Nov
Dec
Jan
Feb
Mar
Apr
month

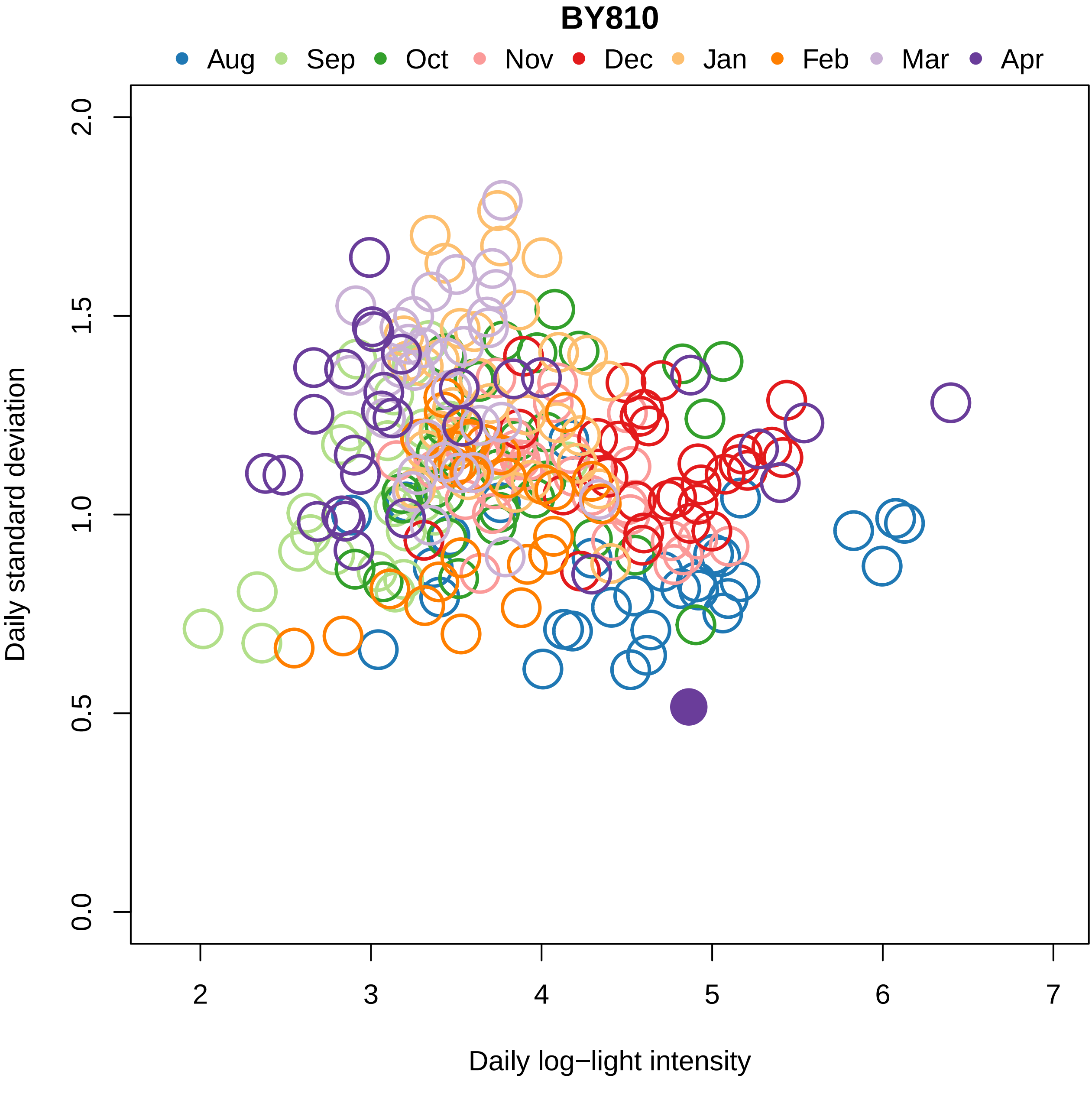

BY810
Aug
Sep
Oct
Nov
Dec
Jan
Feb
Mar
Apr
Daily standard deviation
0.0
0.5
1.0
1.5
2.0
Daily log−light intensity
2
3
4
5
6
7
