# Supplementary material for: Diurnal migration patterns in willow warblers differ between the western and eastern flyways
Source: Mov Ecol. 2023 Sep 21;11:58. doi: 10.1186/s40462-023-00425-x (PMC10512566; doi:10.1186/s40462-023-00425-x)
Supplement: Supplementary file 4 — Additional file4: Maps (.pdf) depicting locations where all the recorded FDFs took place. Black dots show closest reliable locations before and after the FDF event. The width of the red polygon shows the limits of standard deviation in longitude during the exact day when FDF took place [file 40462_2023_425_MOESM4_ESM.pdf]

FDF locations for Autumn

Maps depicting locations where all the recorded FDF's took place. Black dots show closest reliable locations before and after the FDF event. Width of the red polygon shows the limits of standard deviation in longitude during the exact day when FDF took place. Logger number noted in top left corner and cross-referenced in the Additional file 2.

BM199_autumn

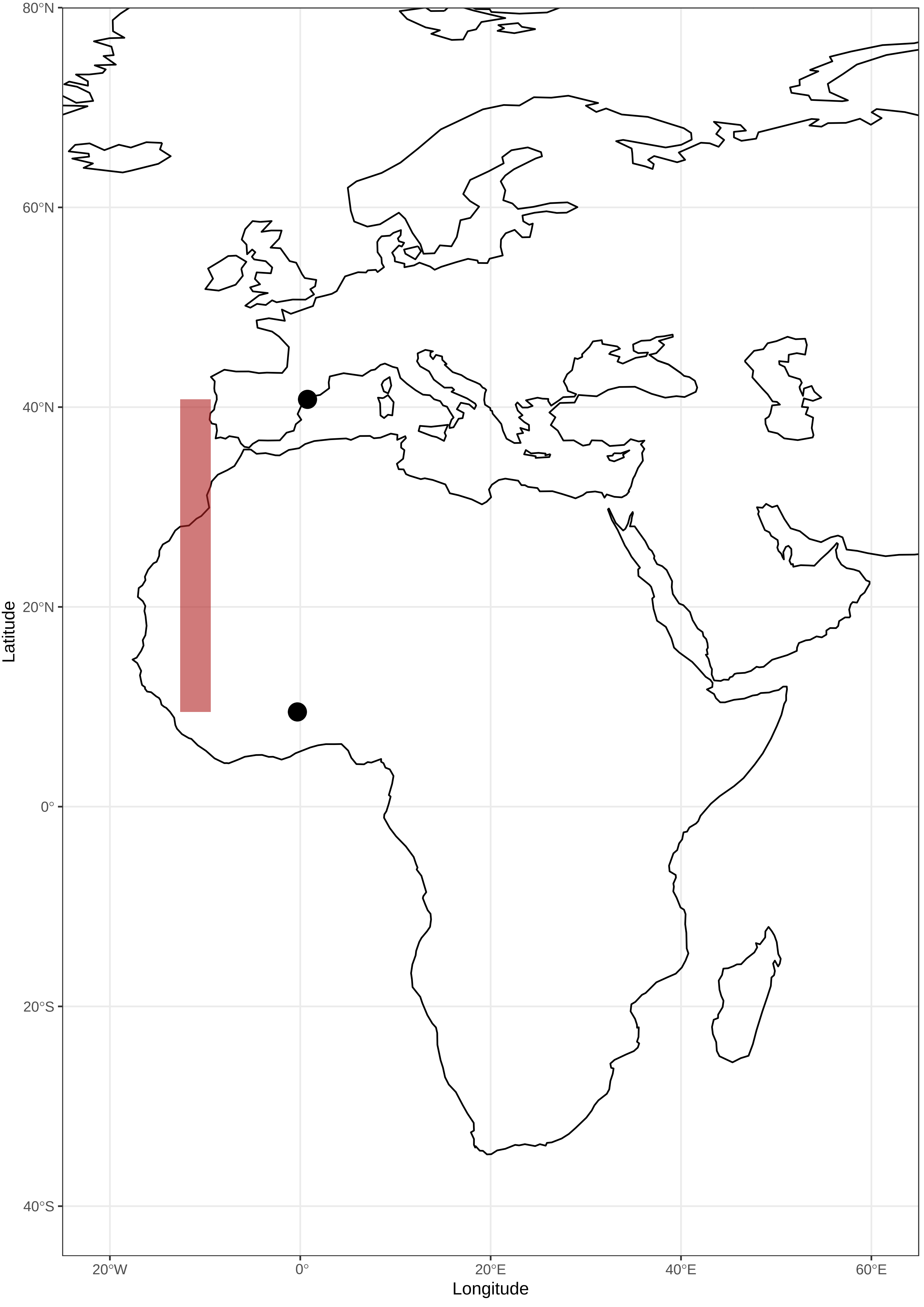

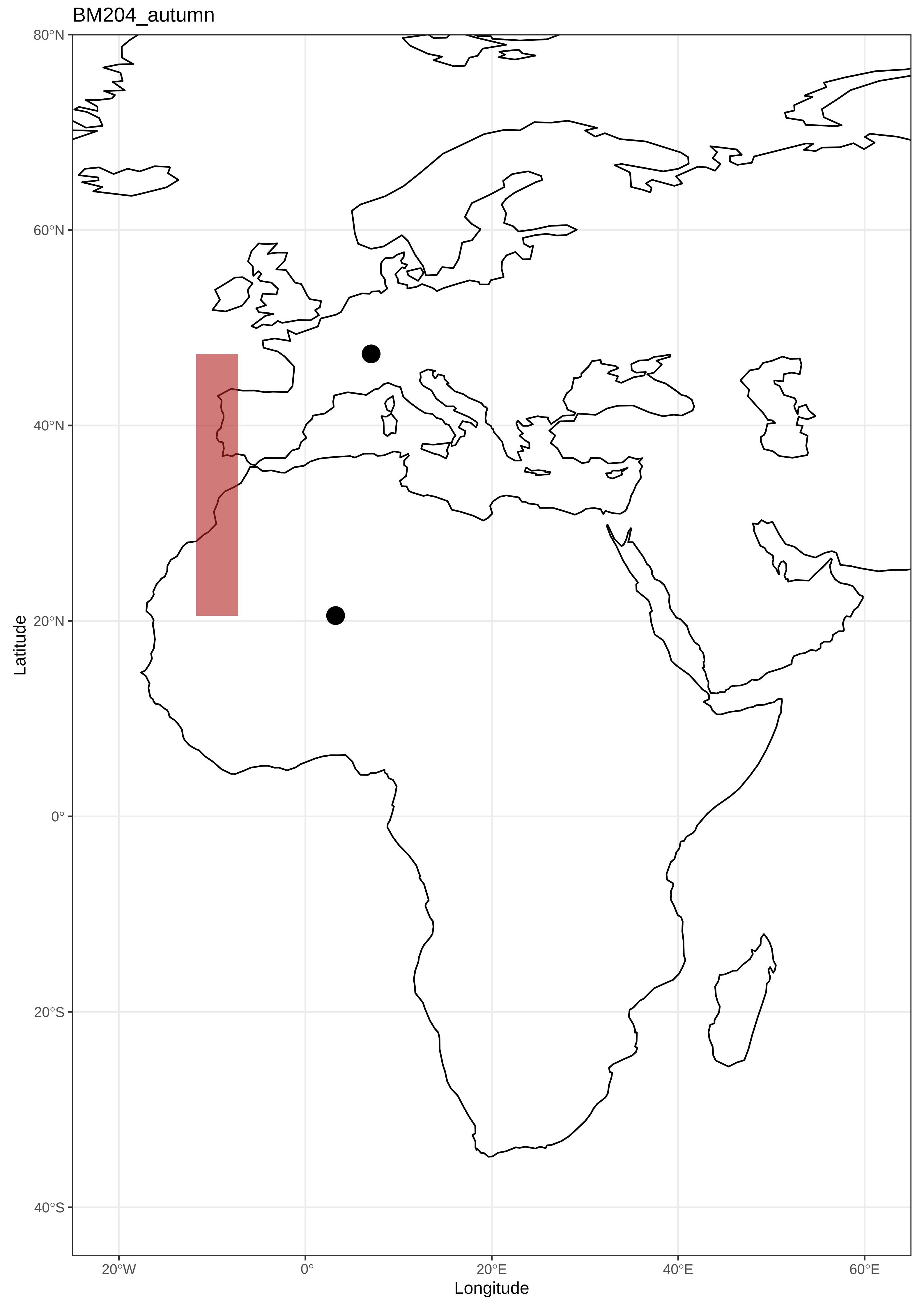
BM204_autumn
80°N
60°N
40°N
20°N
0°
20°S
40°S
Latitude
20°W
0°
20°E
40°E
60°E
Longitude

BM216_autumn

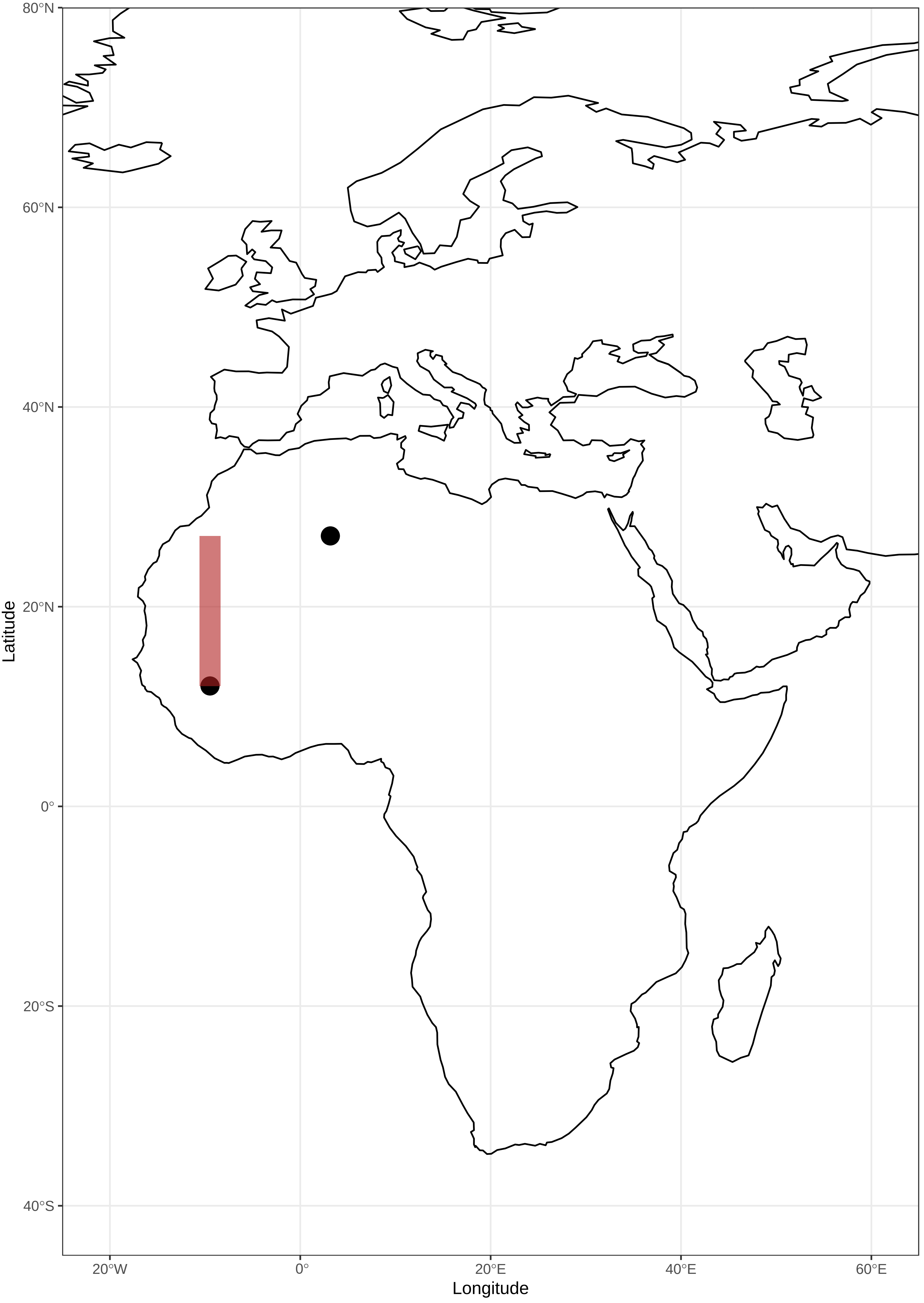

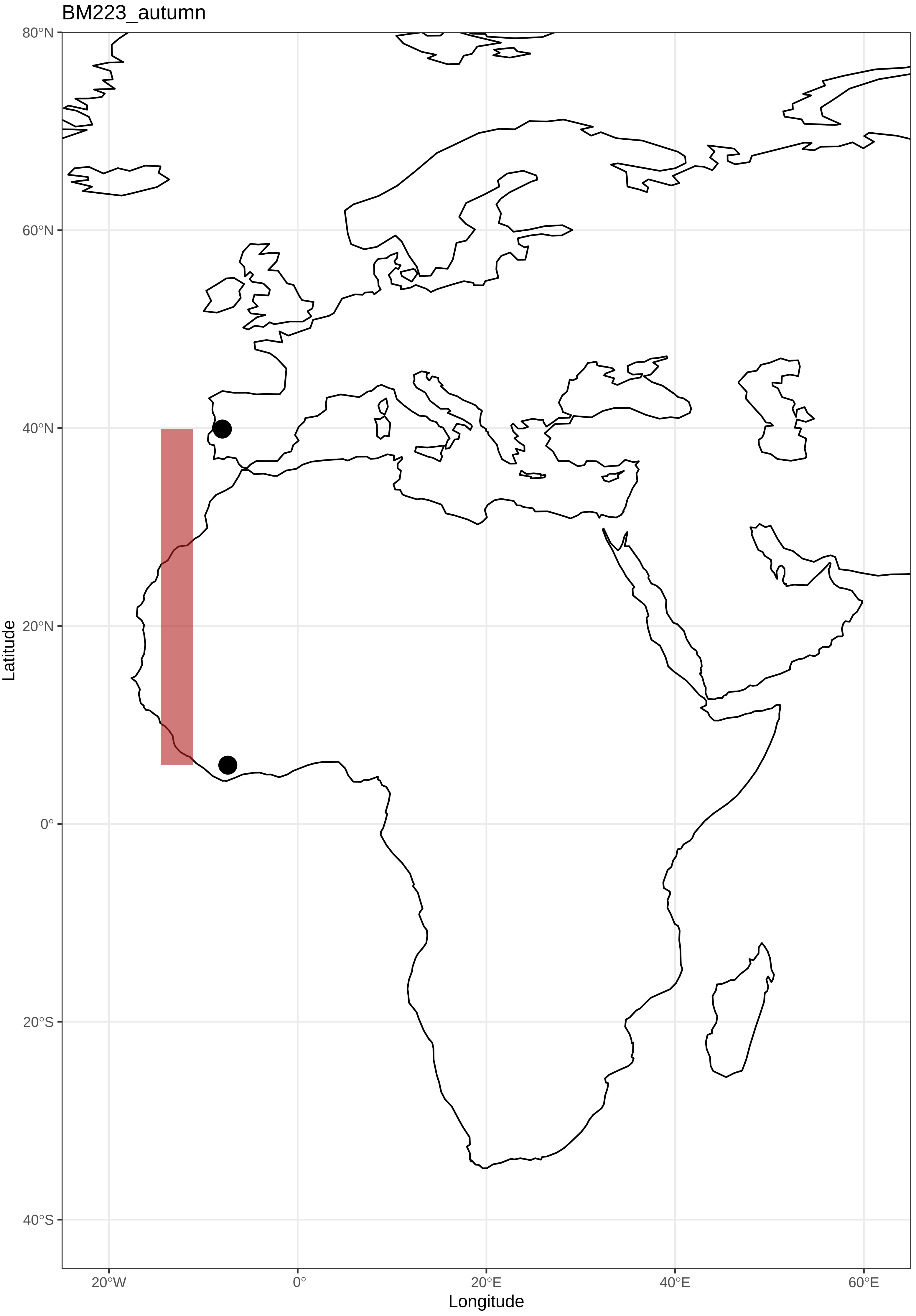

BM223_autumn
80°N
60°N
40°N
20°N
0°
20°S
40°S
20°W
0°
20°E
40°E
60°E
Longitude
Latitude

BM225_aut

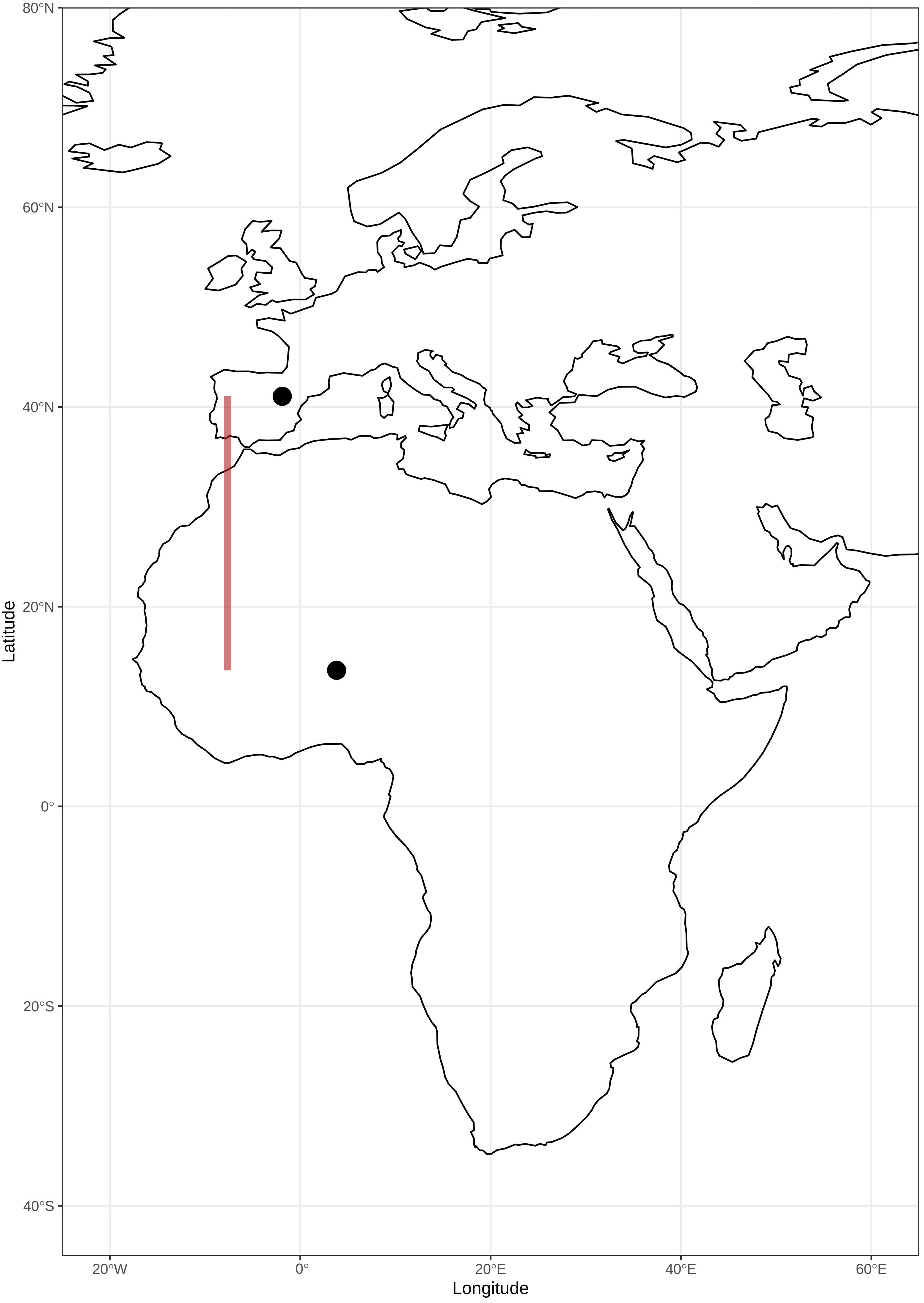

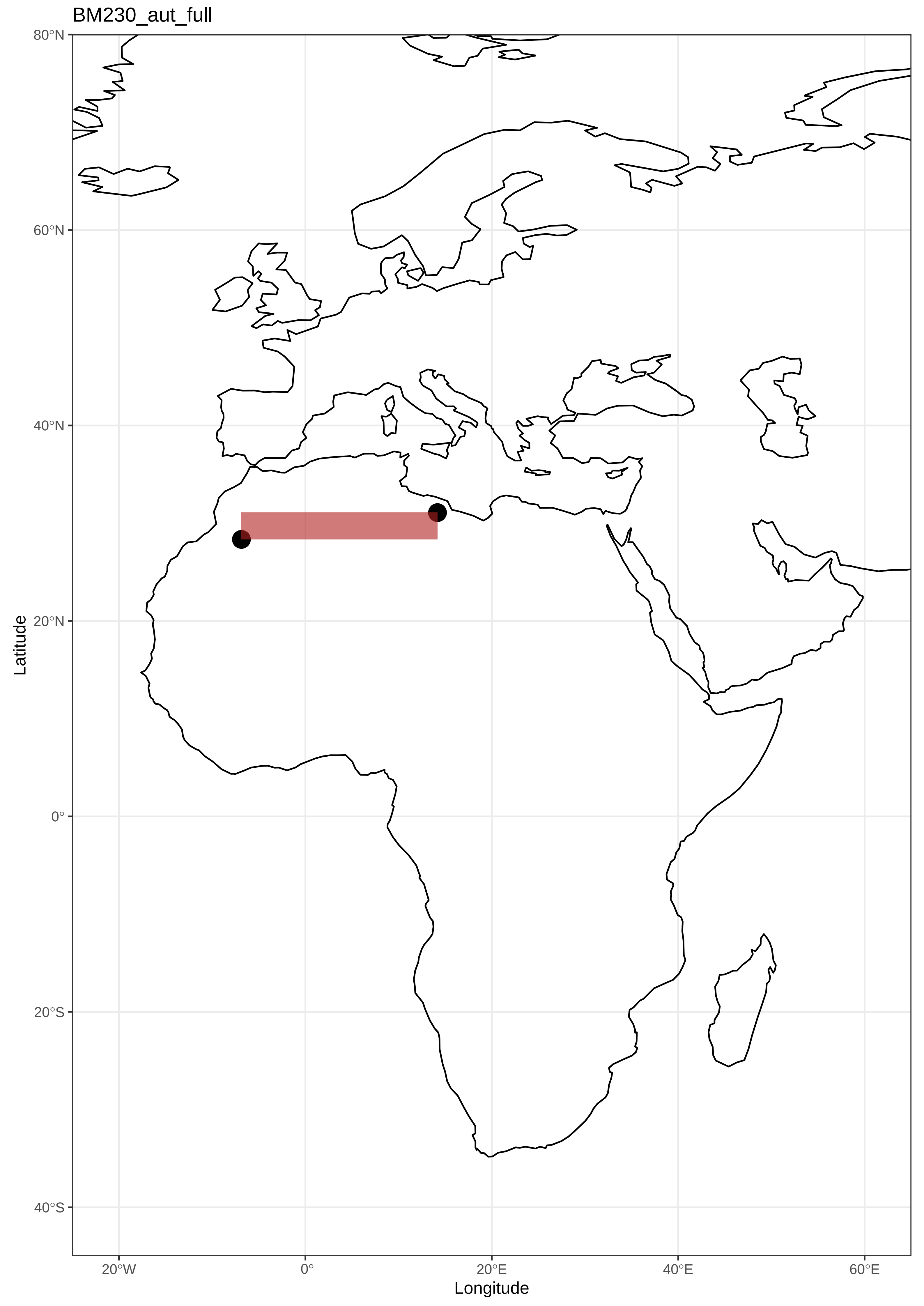

BM230_aut_full
80°N
60°N
40°N
20°N
0°
20°S
40°S
Latitude
20°W
0°
20°E
40°E
60°E
Longitude

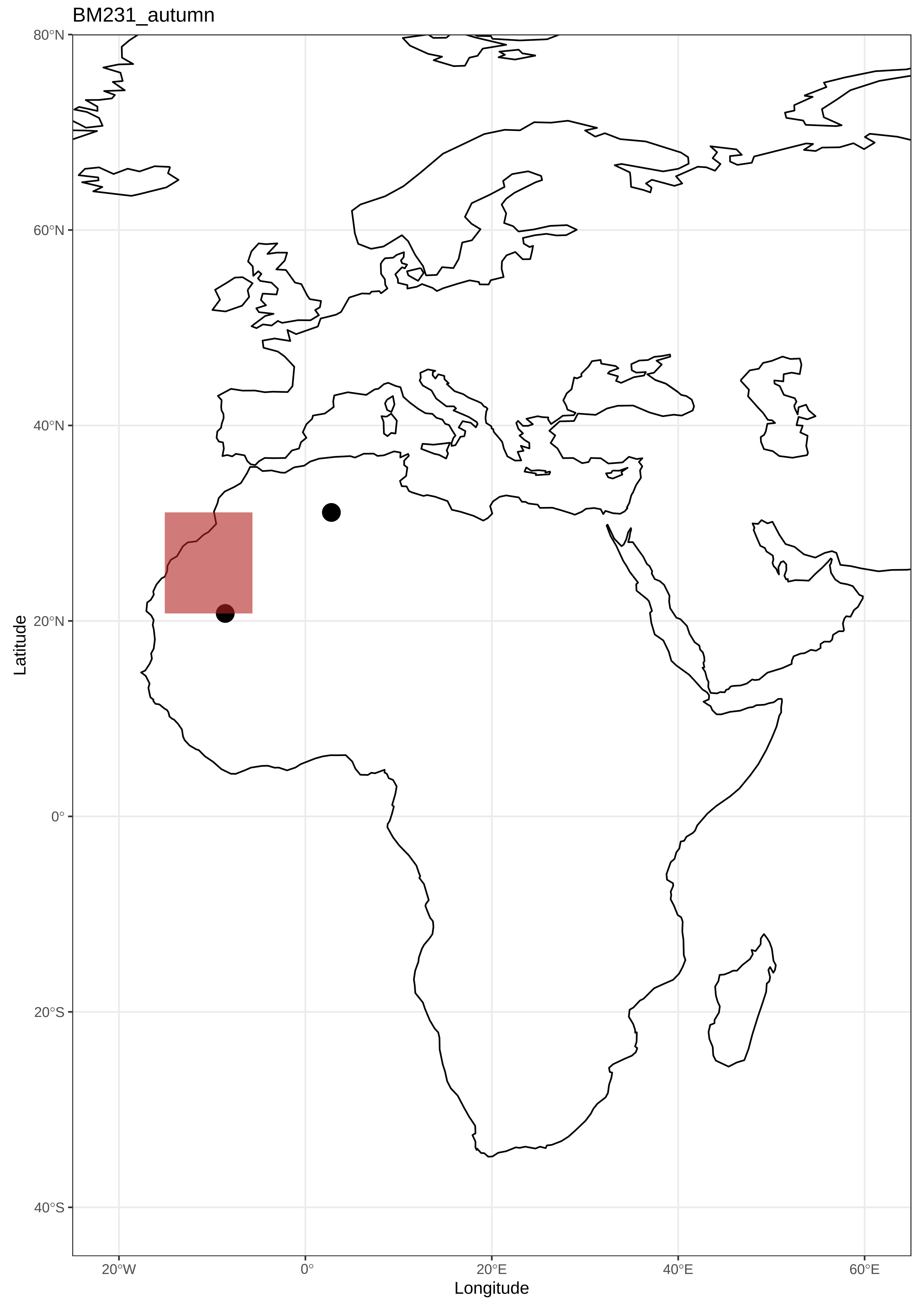
BM231_autumn
80°N
60°N
40°N
20°N
0°
20°S
40°S
Latitude
20°W
0°
20°E
40°E
60°E
Longitude

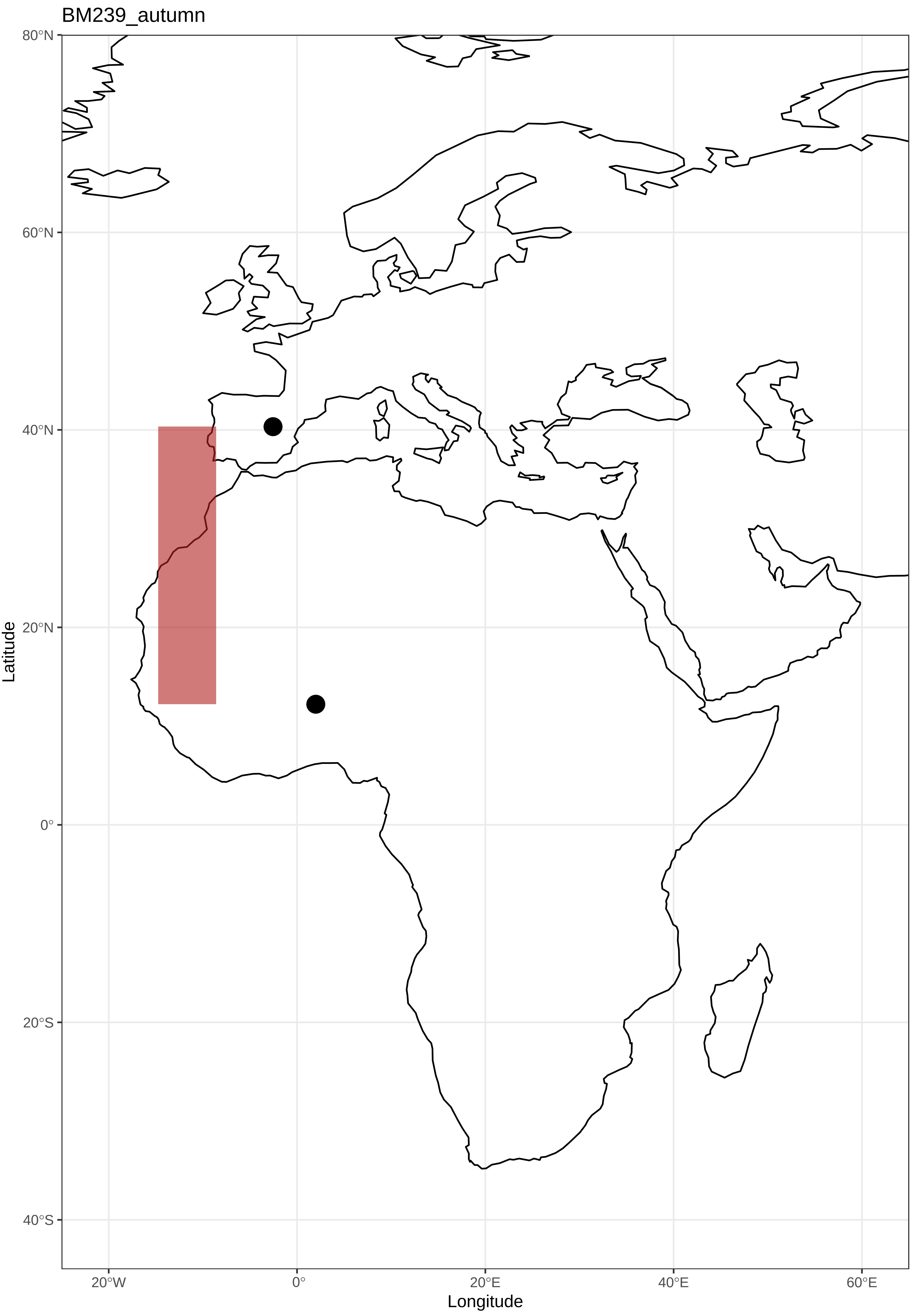
BM239_autumn
80°N
60°N
40°N
20°N
0°
20°S
40°S
Latitude
20°W
0°
20°E
40°E
60°E
Longitude

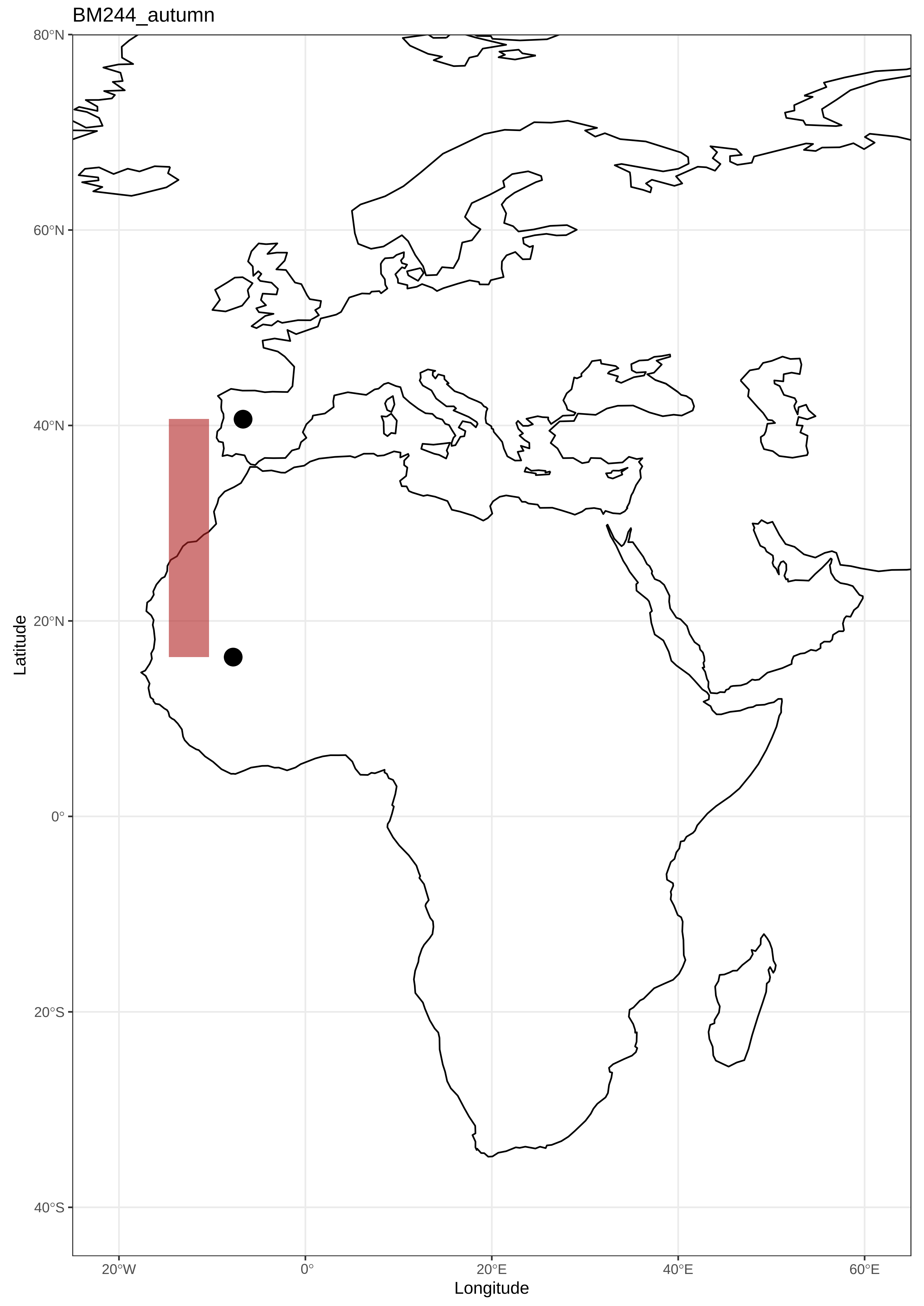
BM244_autumn
80°N
60°N
40°N
20°N
0°
20°S
40°S
Latitude
20°W
0°
20°E
40°E
60°E
Longitude

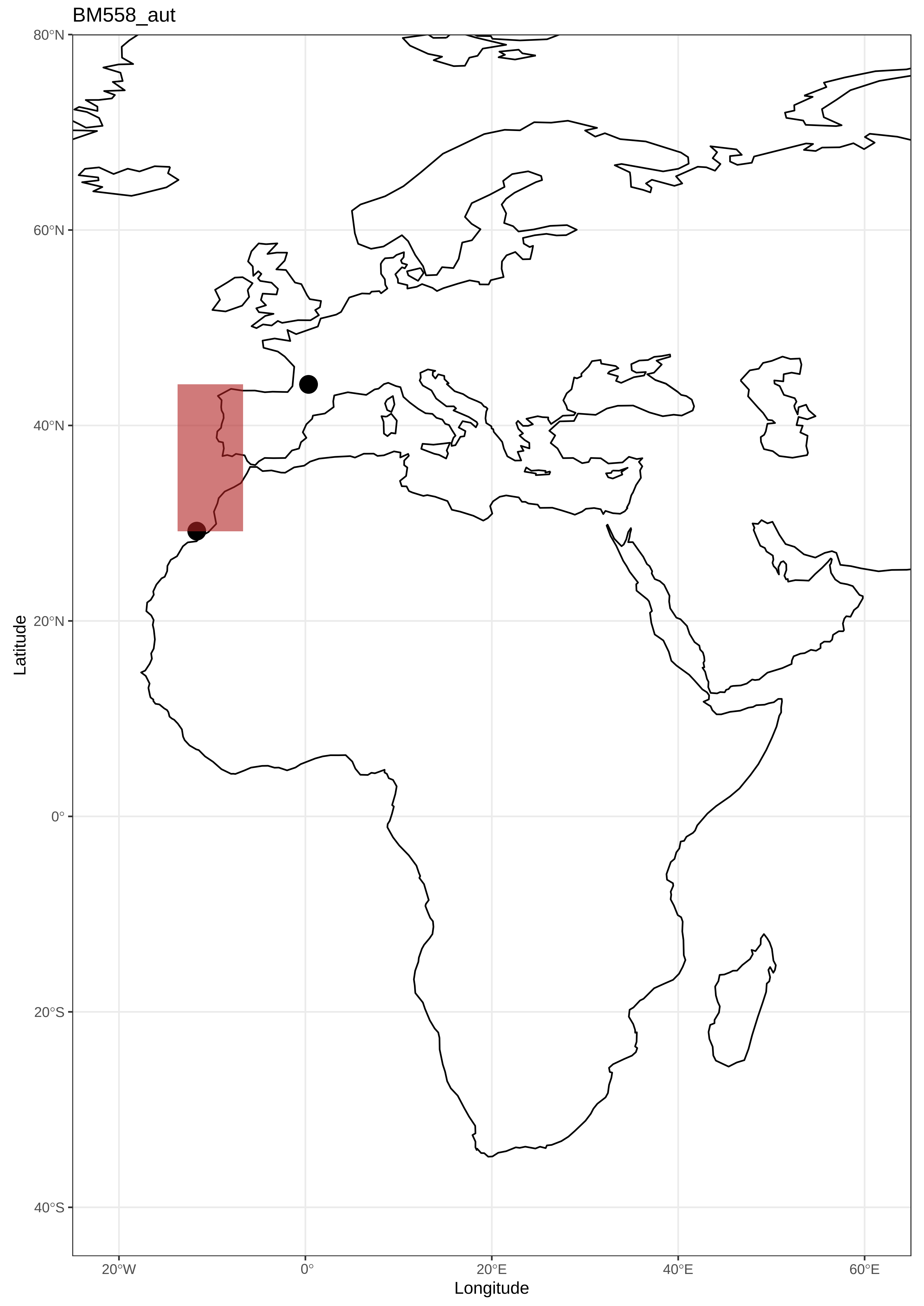
BM558_aut
80°N
60°N
40°N
20°N
0°
20°S
40°S
Latitude
20°W
0°
20°E
40°E
60°E
Longitude

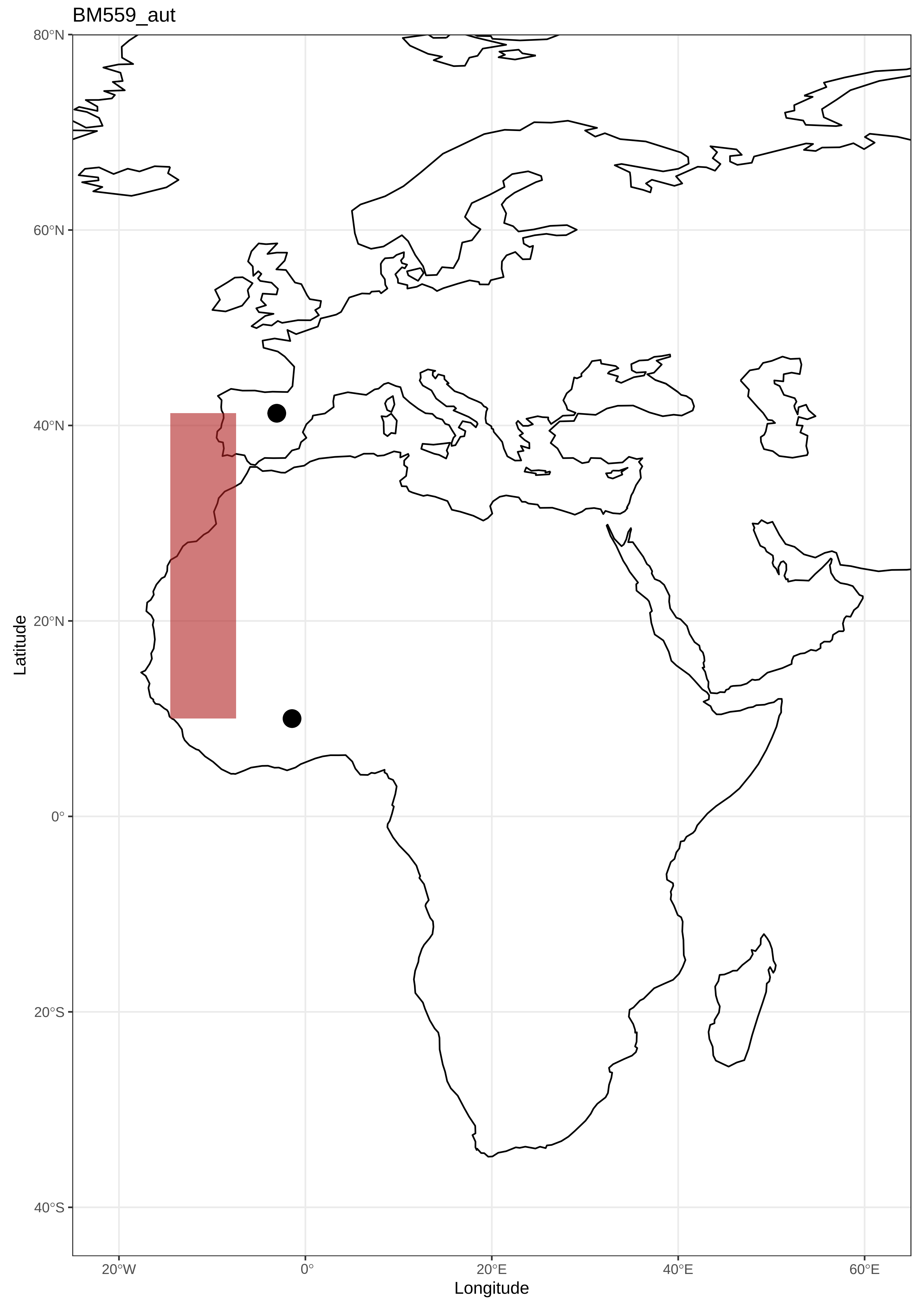

BM559_aut
80°N
60°N
40°N
20°N
0°
20°S
40°S
Latitude
20°W
0°
20°E
40°E
60°E
Longitude

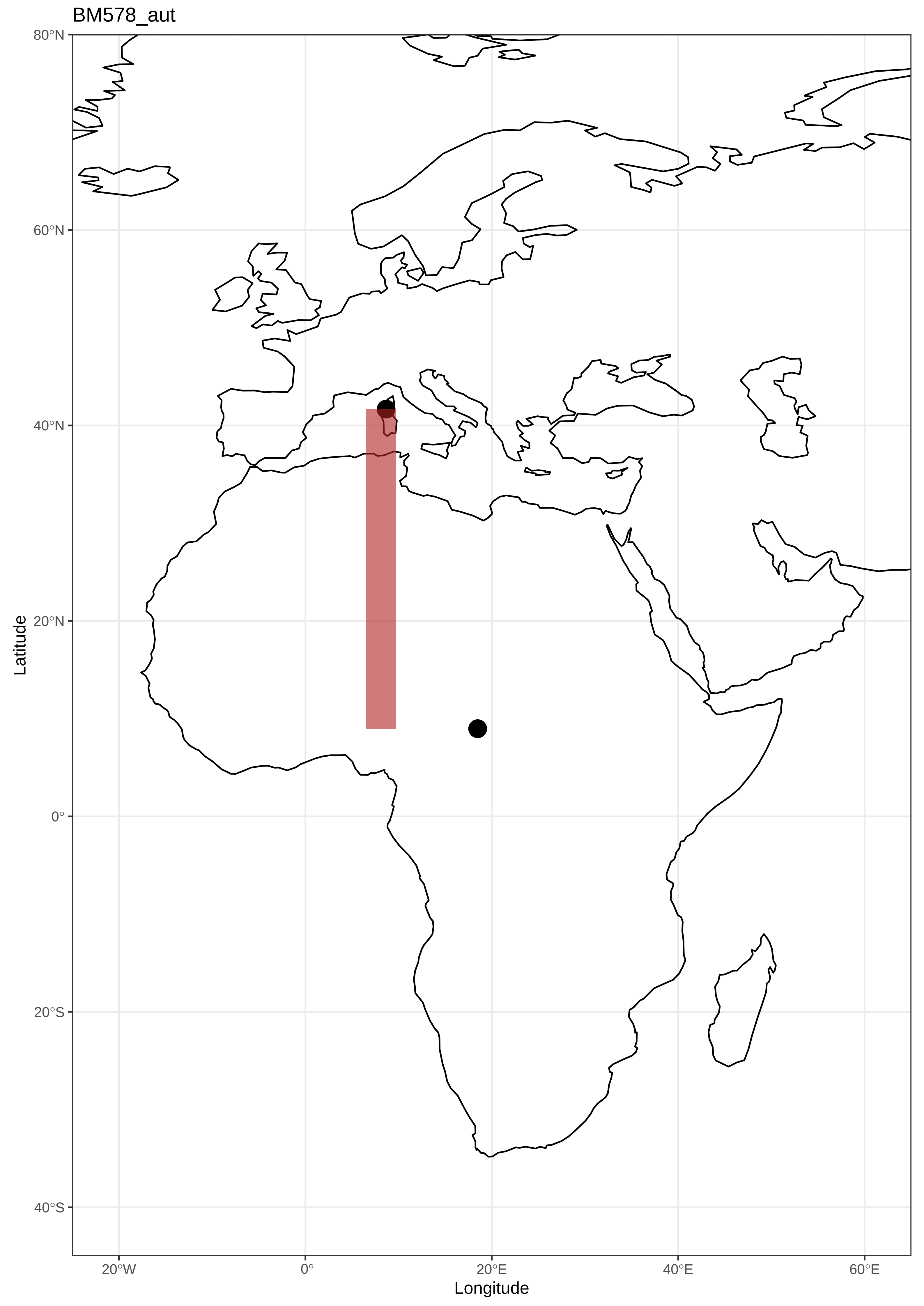
BM578_aut
80°N
60°N
40°N
20°N
0°
20°S
40°S
Latitude
20°W
0°
20°E
40°E
60°E
Longitude

BM583_aut

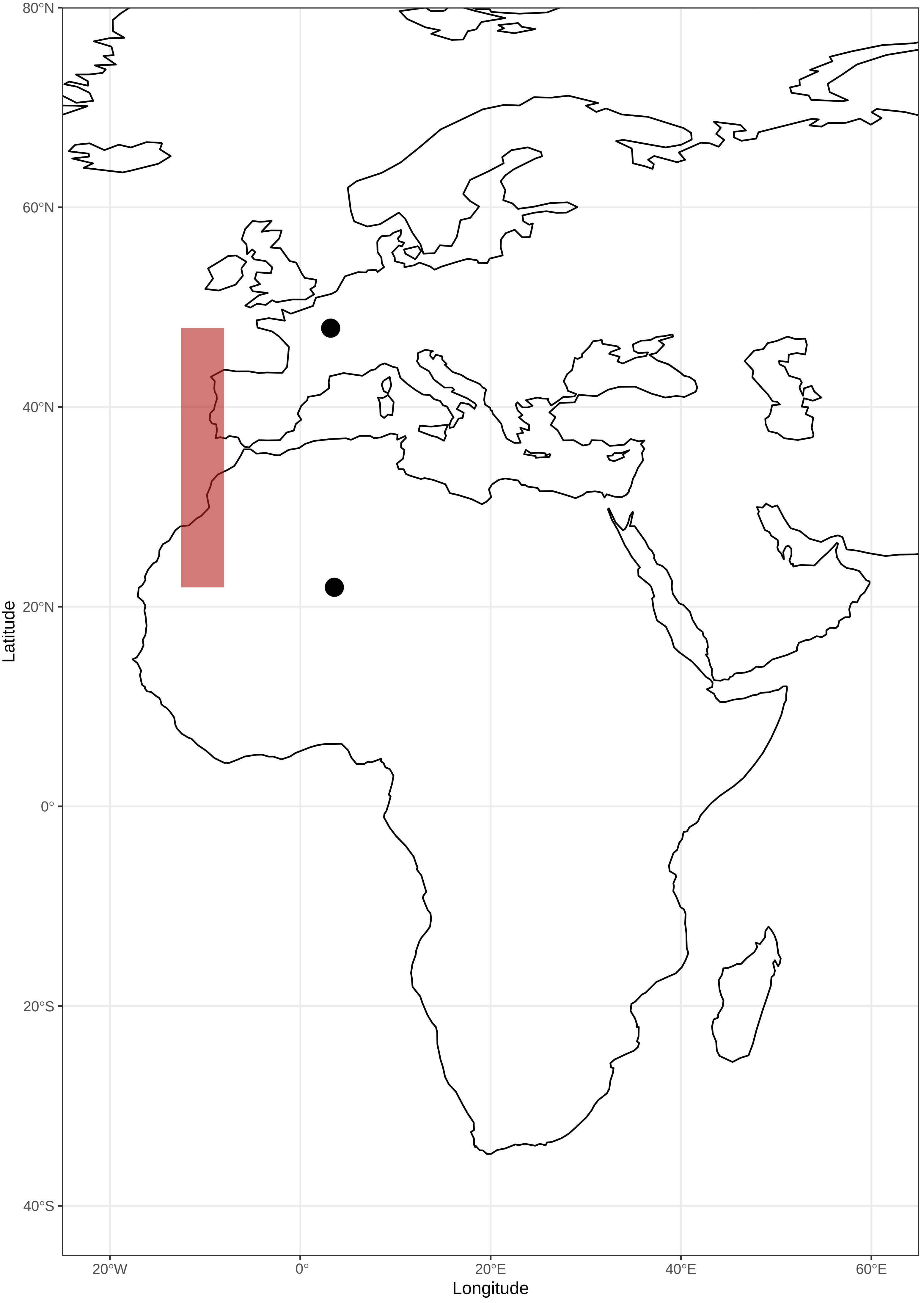

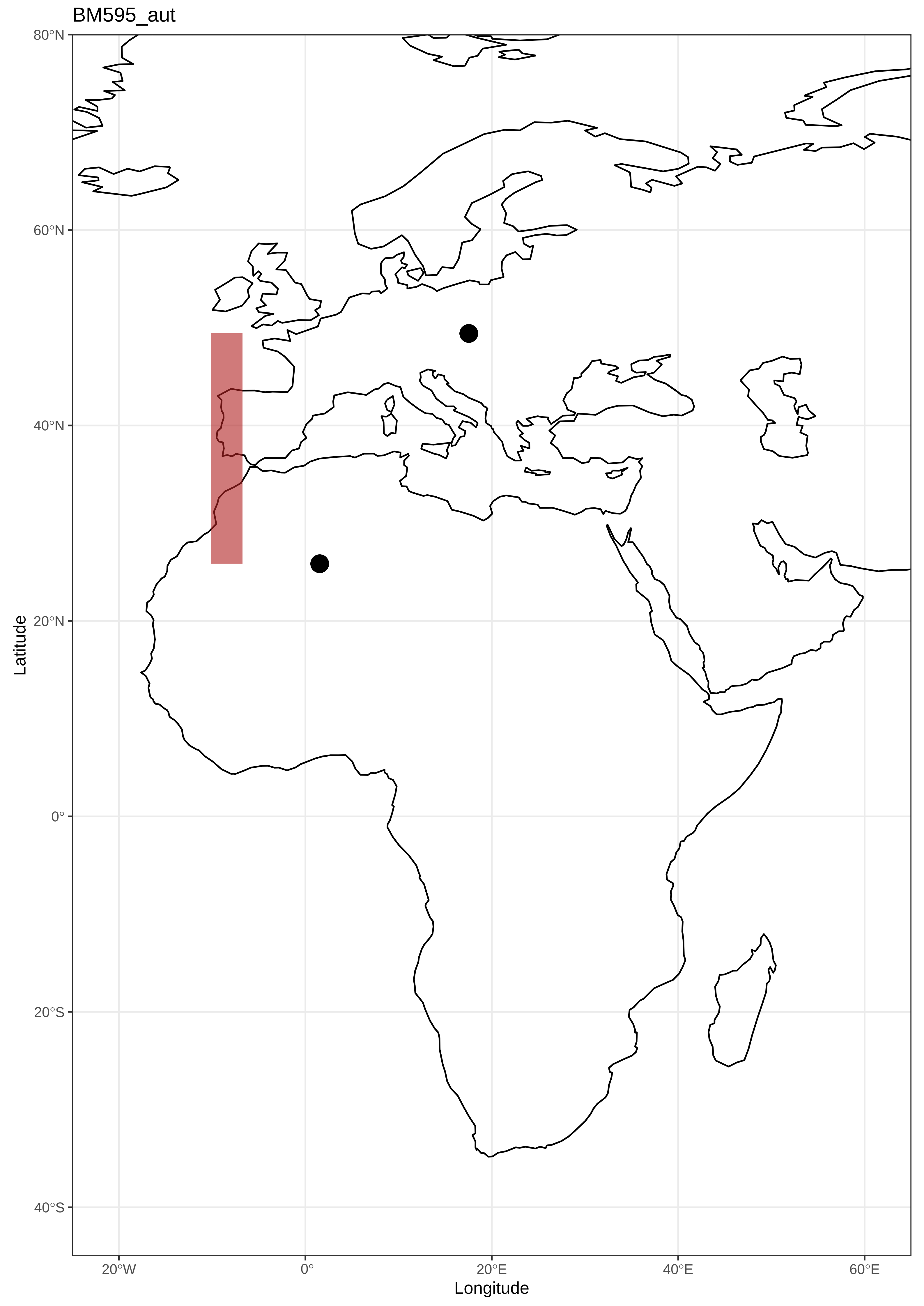

BM595_aut
80°N
60°N
40°N
20°N
0°
20°S
40°S
Latitude
20°W
0°
20°E
40°E
60°E
Longitude

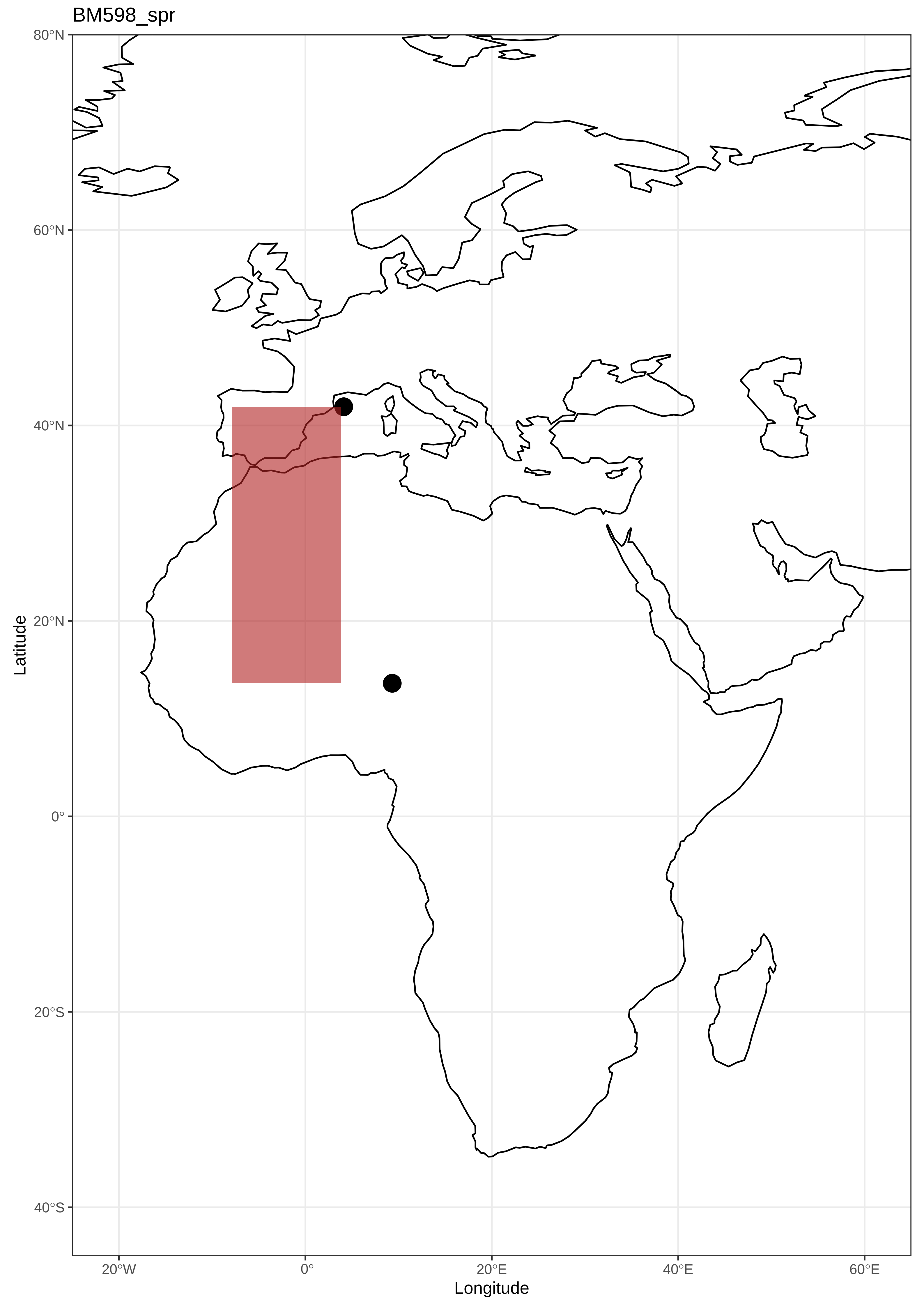

BM598_spr
80°N
60°N
40°N
20°N
0°
20°S
40°S
20°W
0°
20°E
40°E
60°E
Latitude
Longitude

BM658_aut

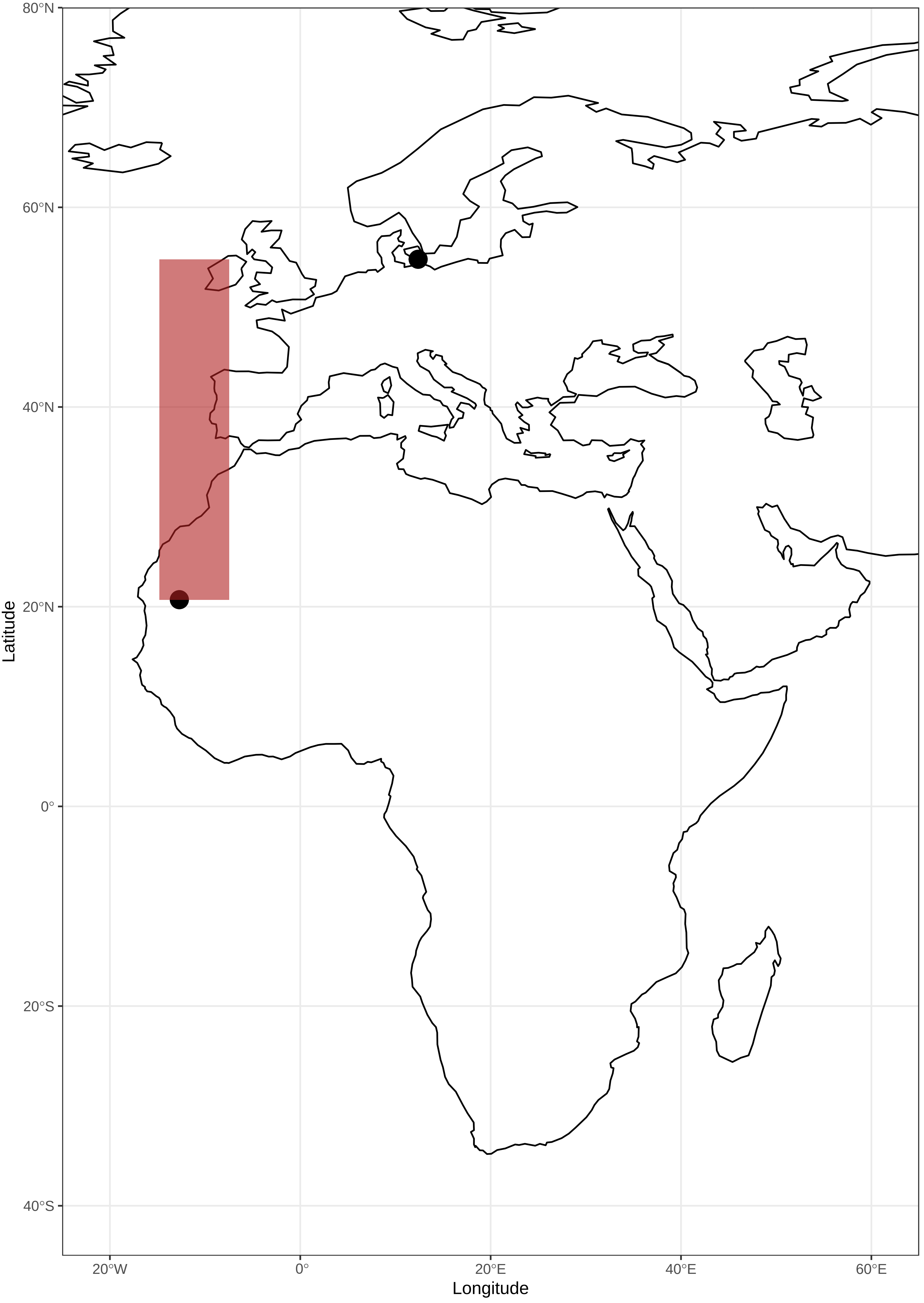

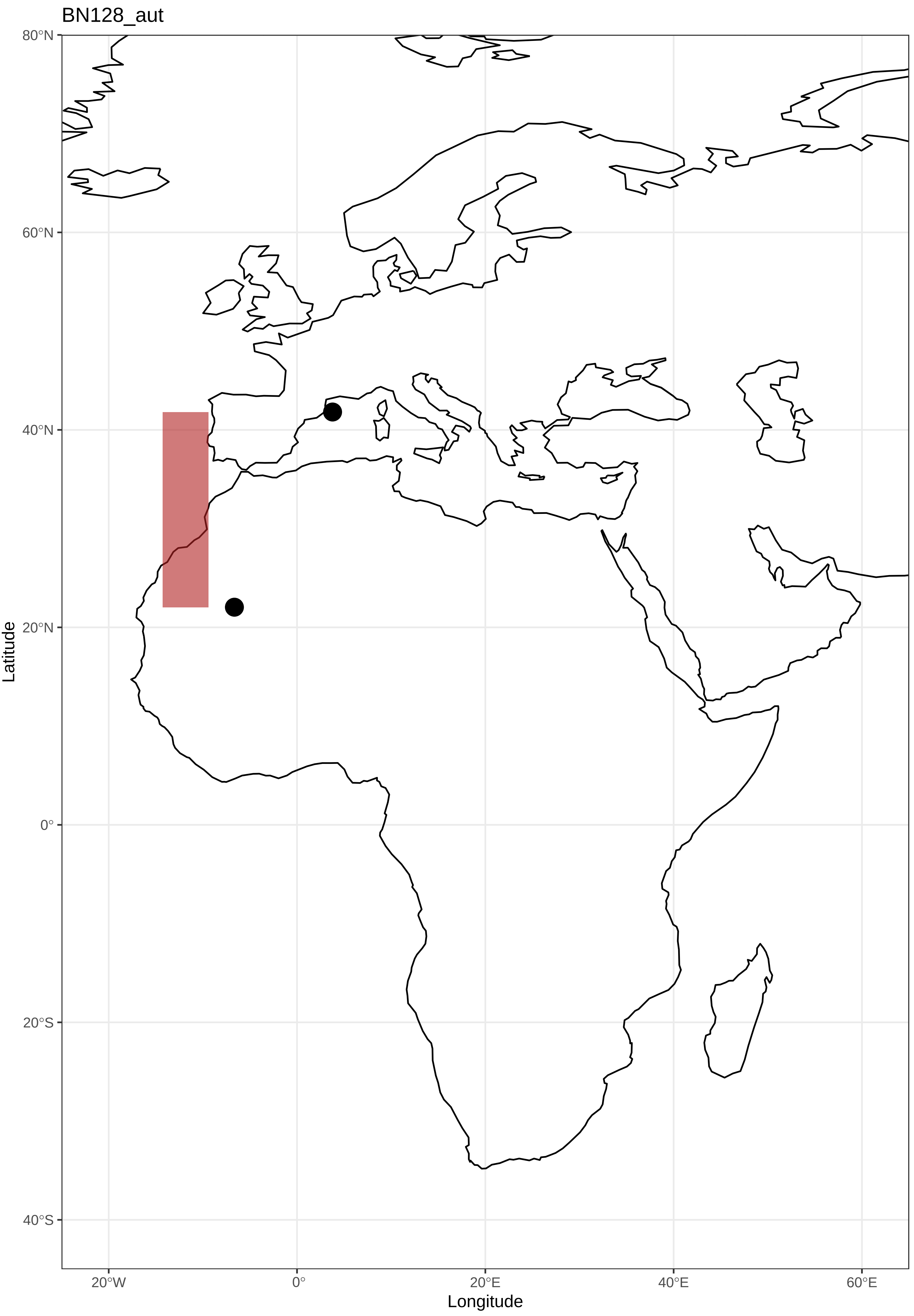
BN128_aut
80°N
60°N
40°N
20°N
0°
20°S
40°S
Latitude
20°W
0°
20°E
40°E
60°E
Longitude

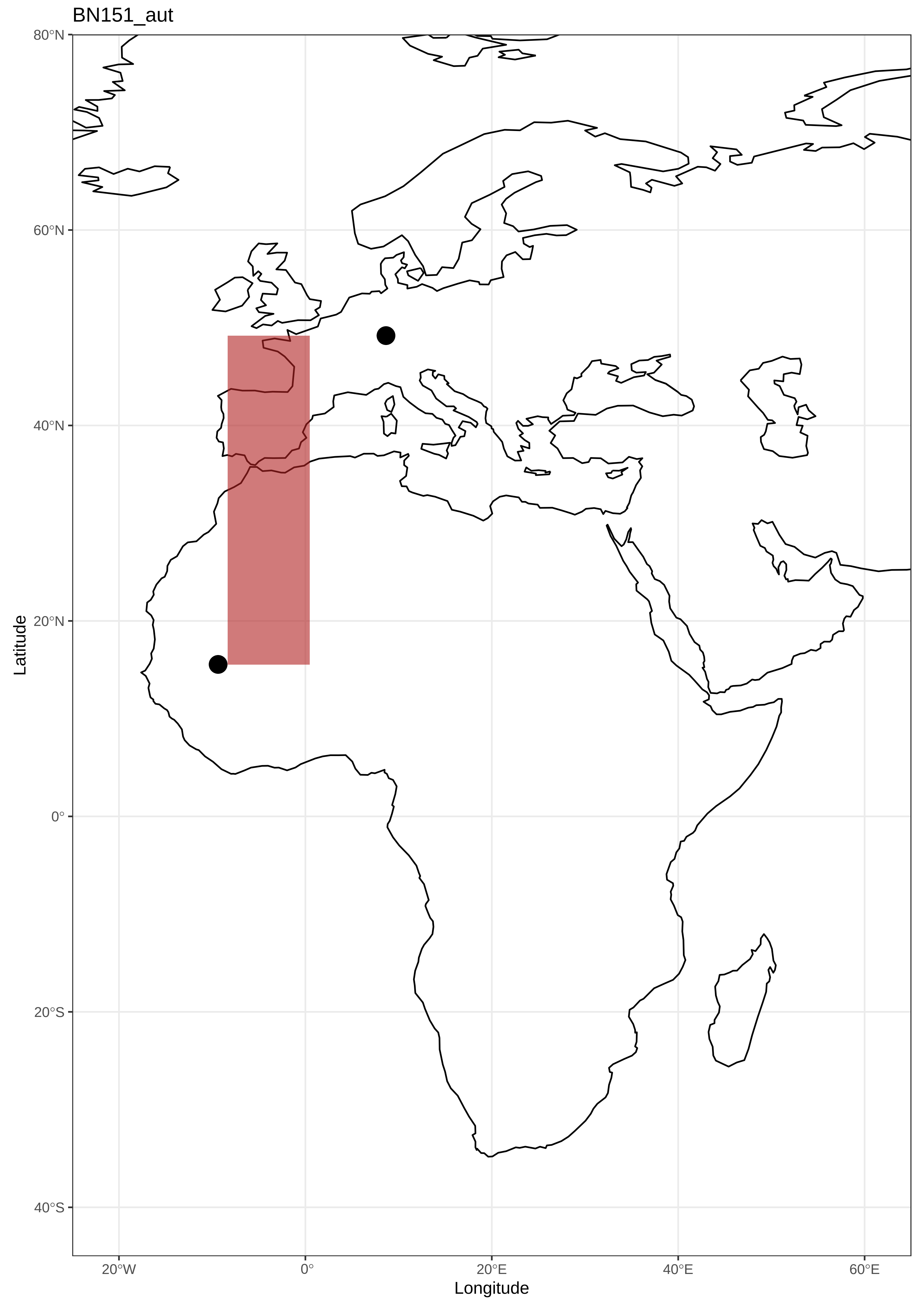

BN151_aut
80°N
60°N
40°N
20°N
0°
20°S
40°S
Latitude
20°W
0°
20°E
40°E
60°E
Longitude

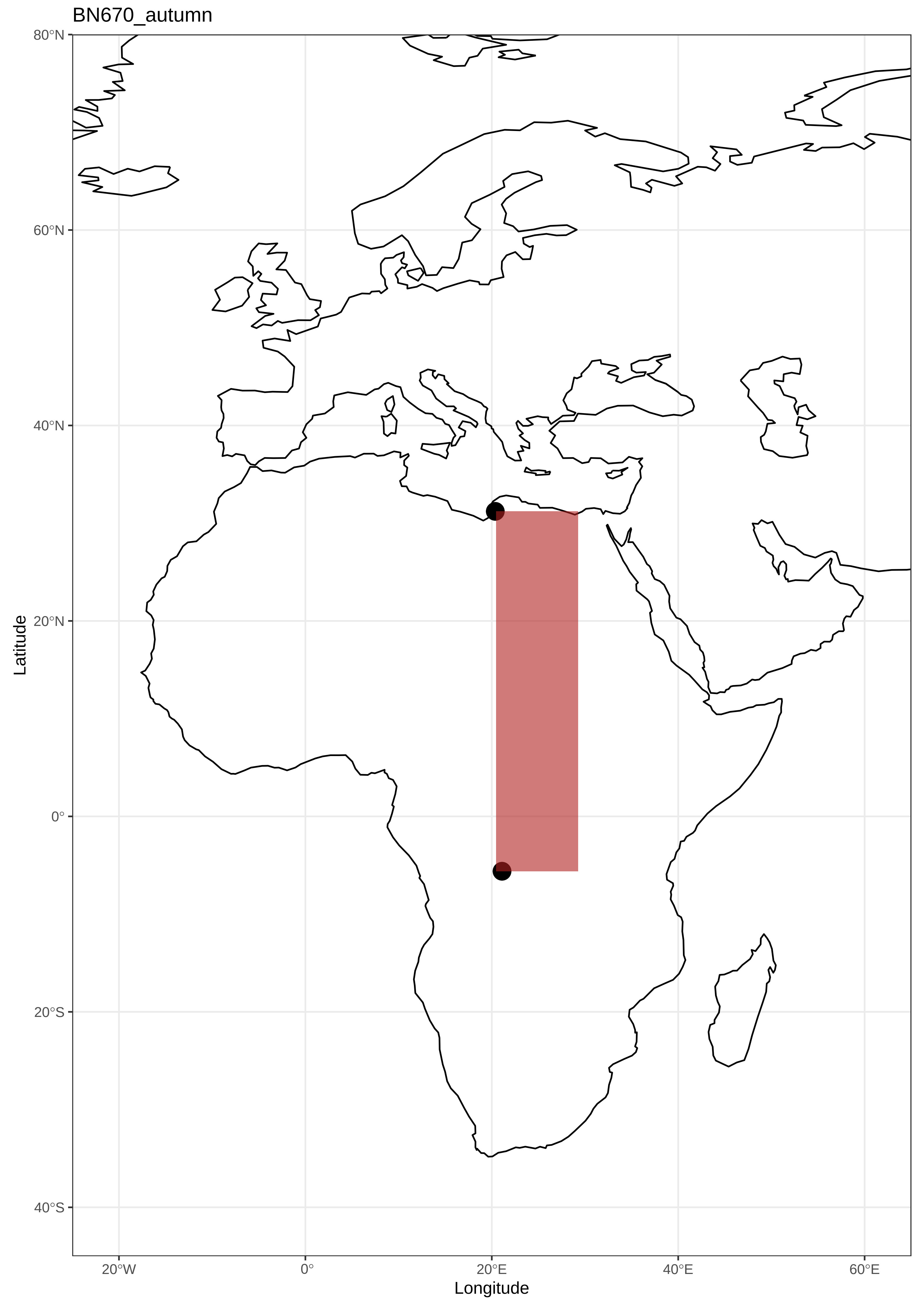
BN670_autumn
80°N
60°N
40°N
20°N
0°
20°S
40°S
Latitude
20°W
0°
20°E
40°E
60°E
Longitude

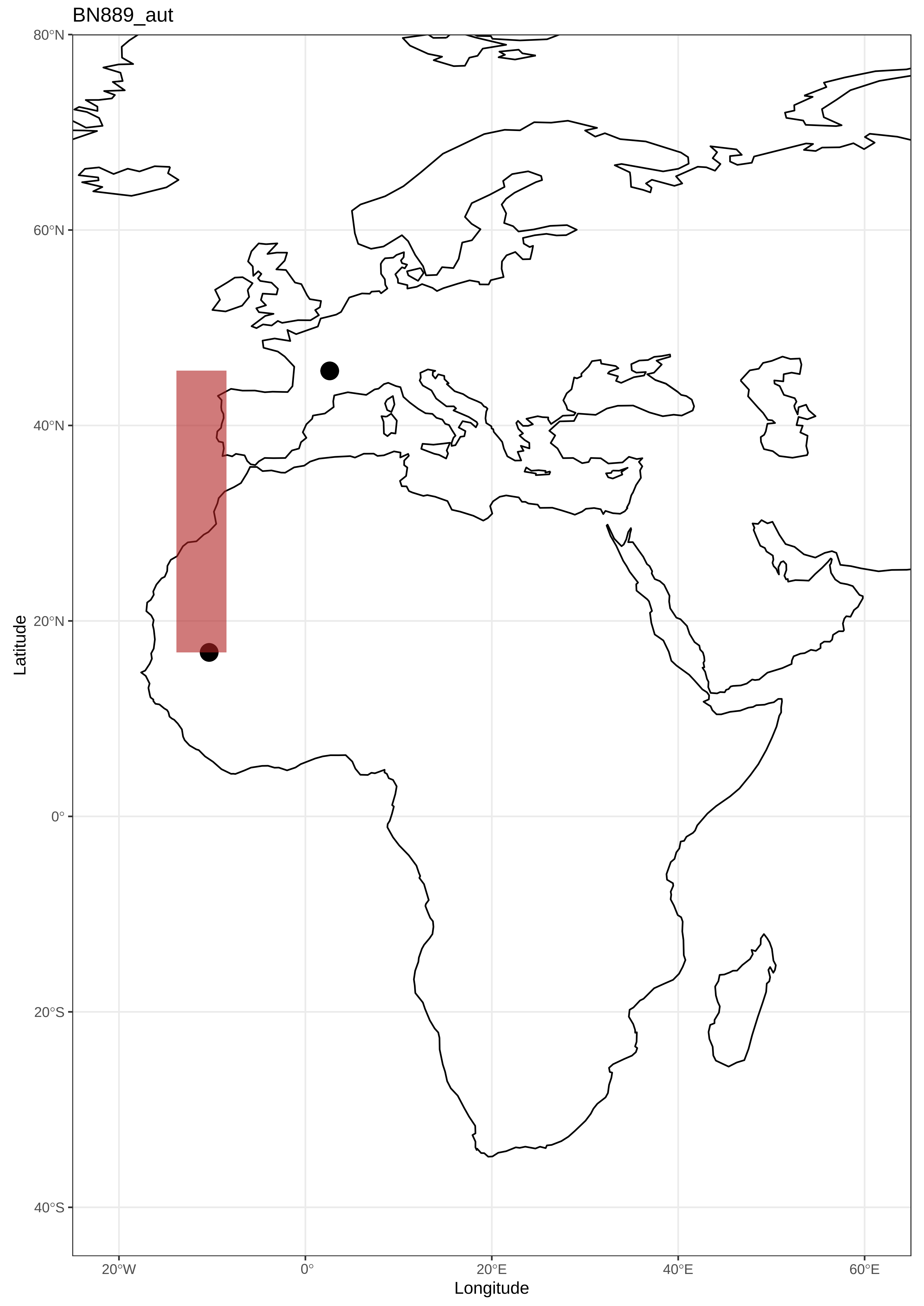

BN889_aut
80°N
60°N
40°N
20°N
0°
20°S
40°S
Latitude
20°W
0°
20°E
40°E
60°E
Longitude

BN902_aut

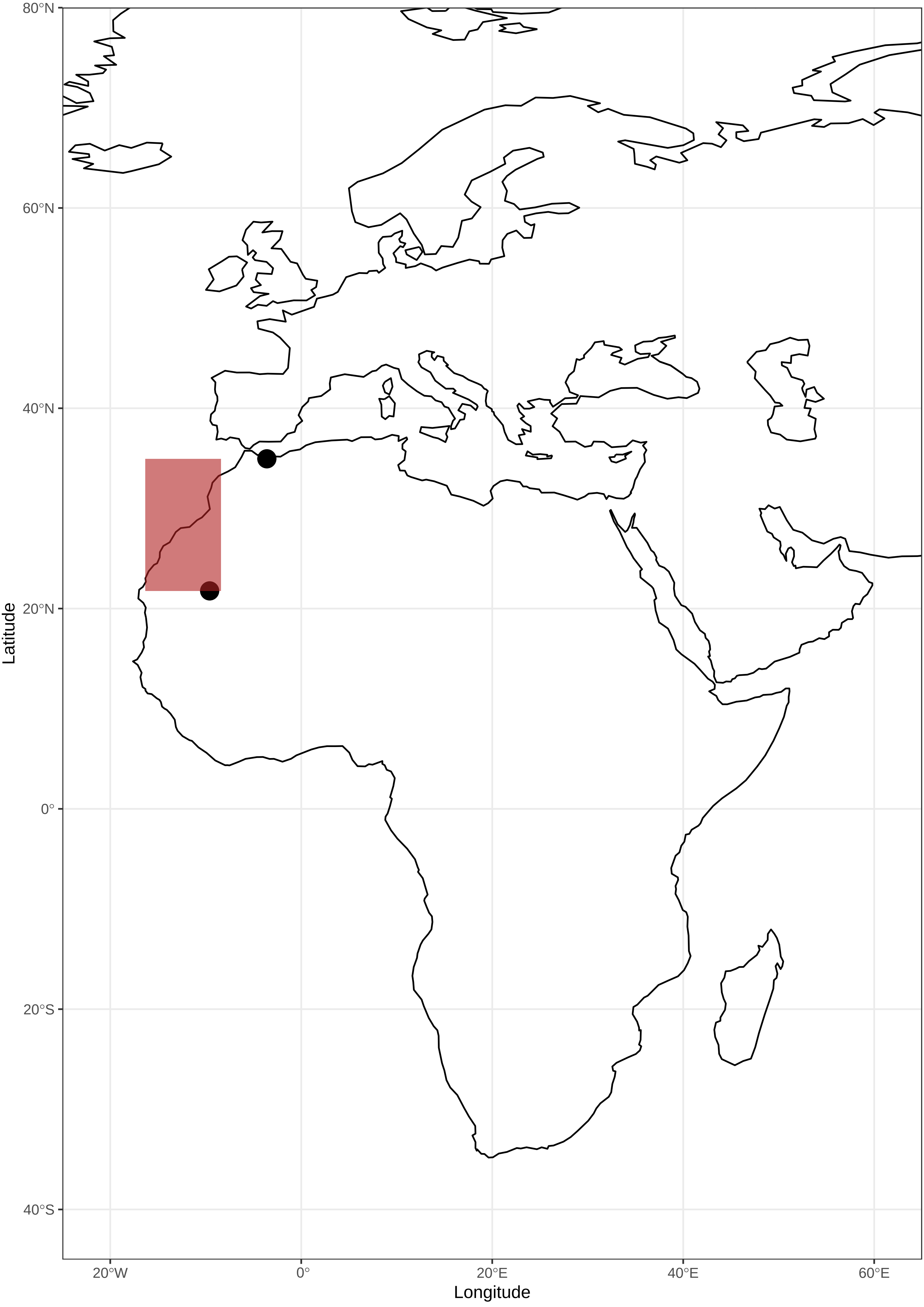

BN934_aut

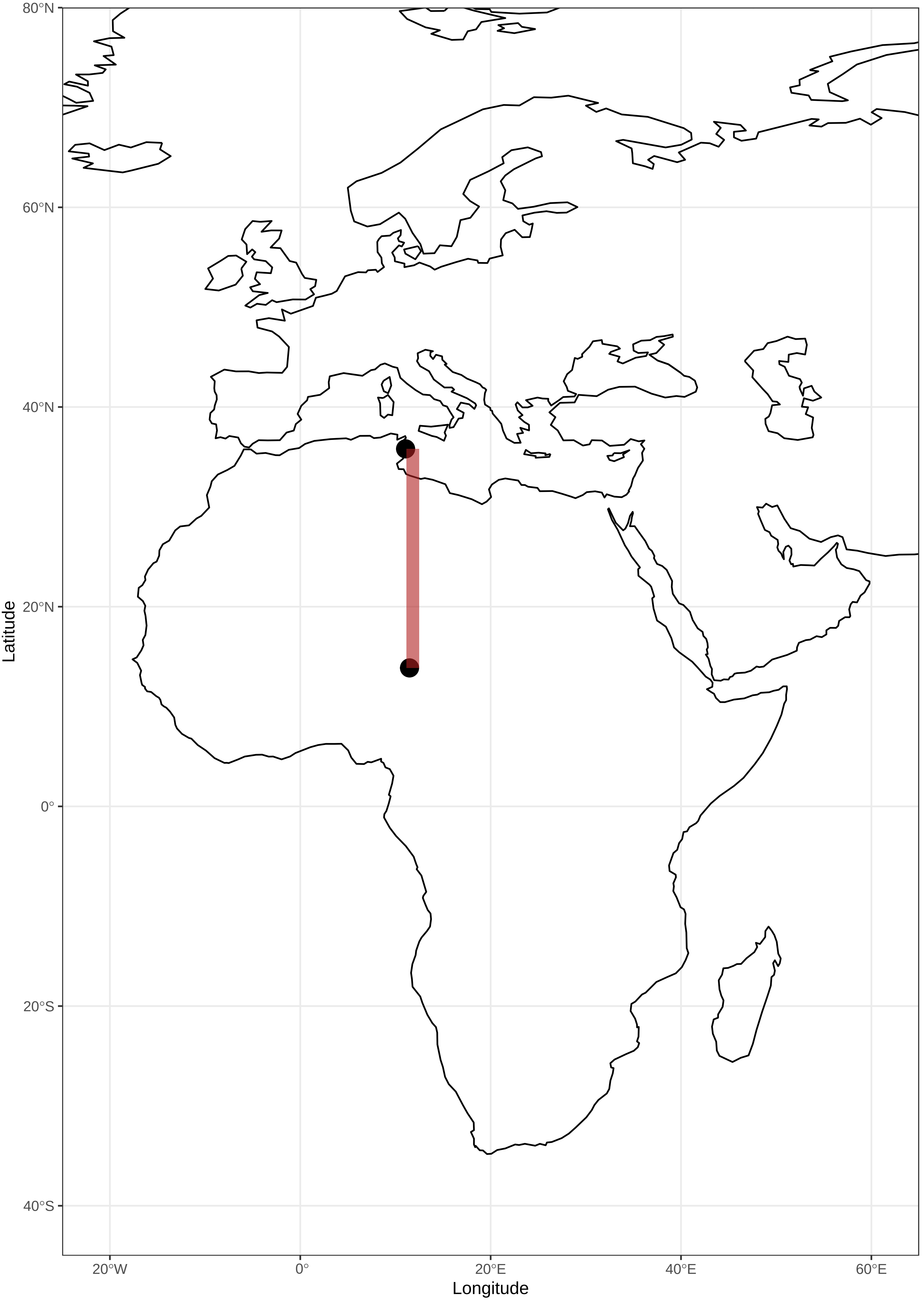

BY761_aut

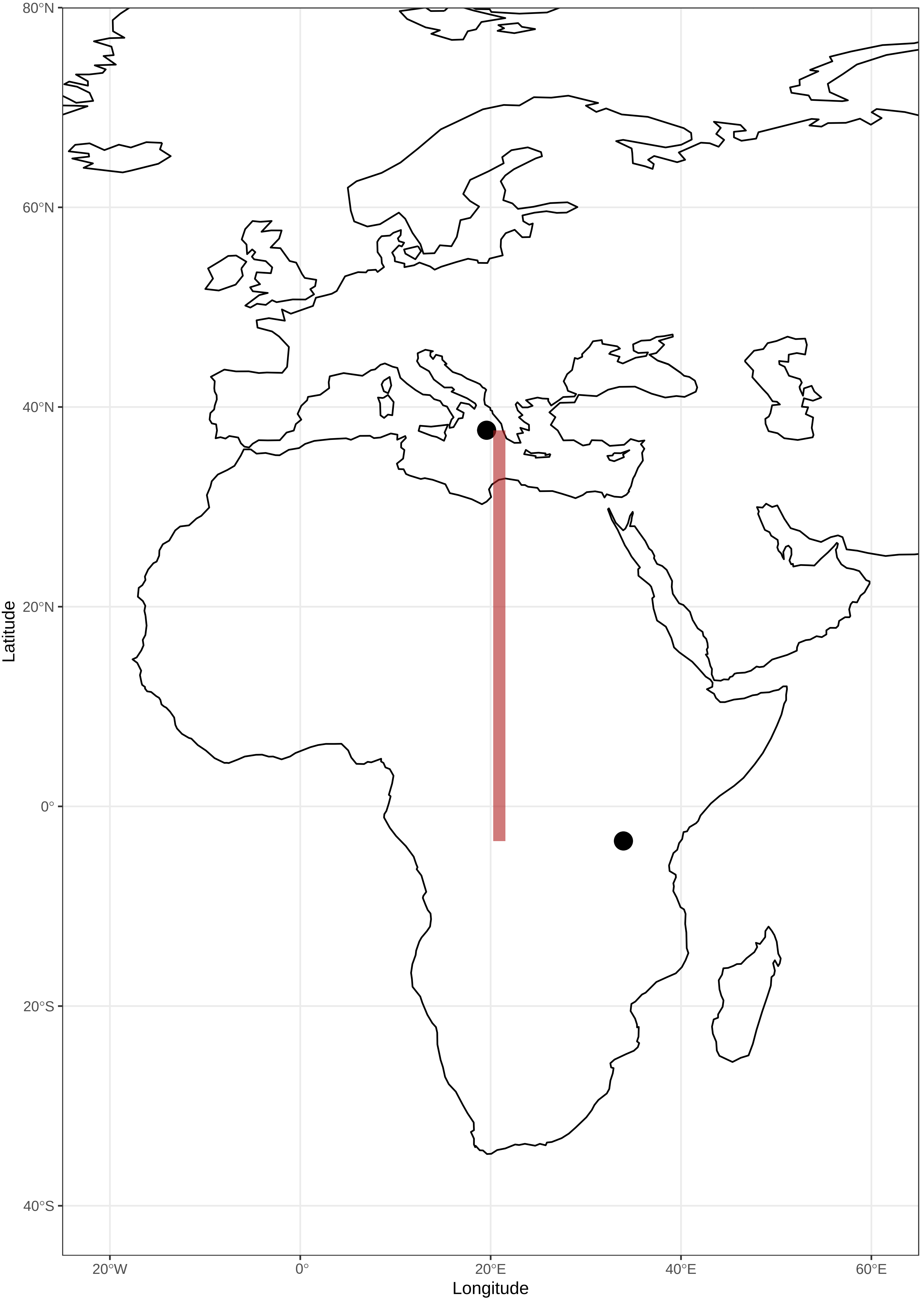

BY802_aut

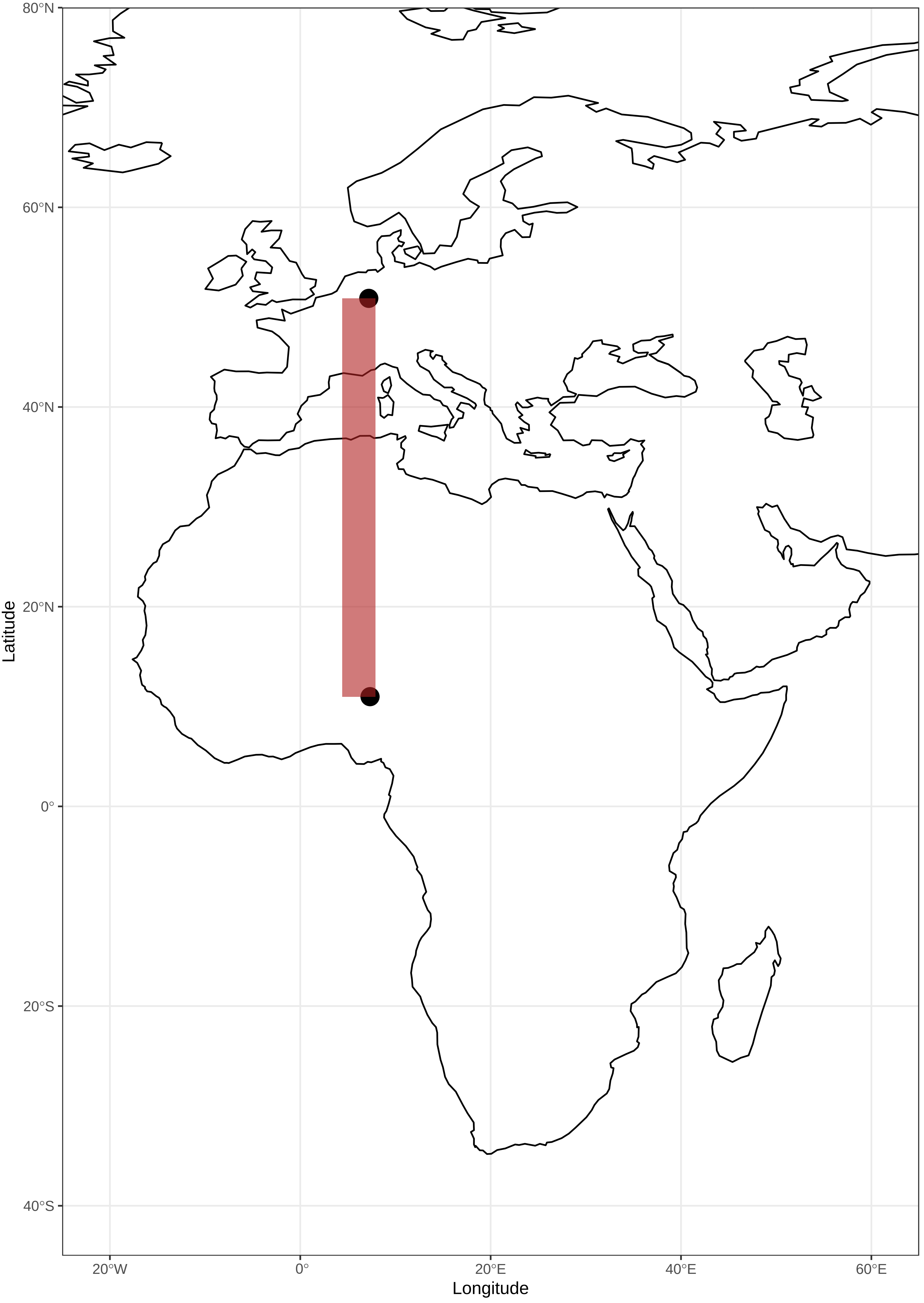

Spring

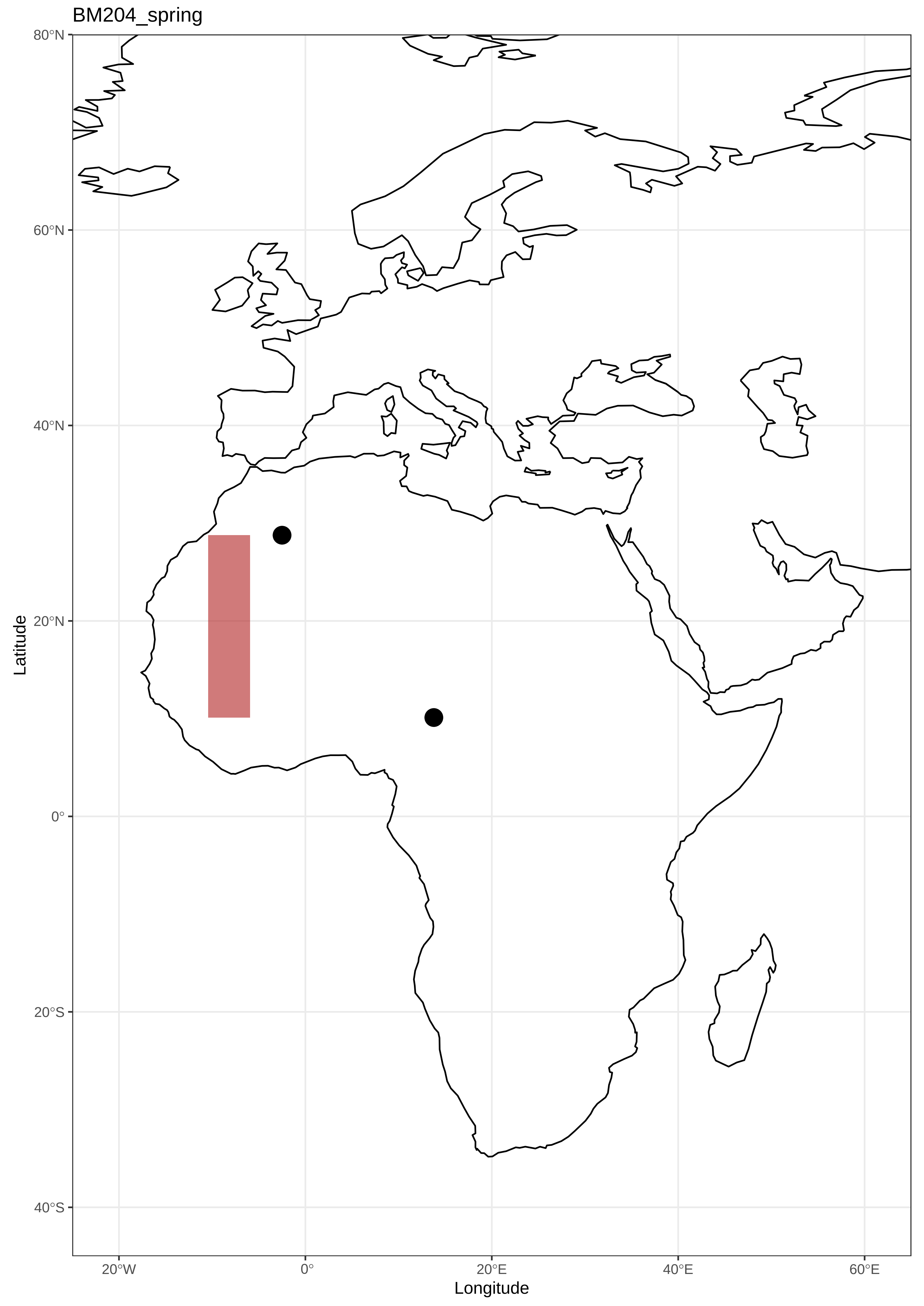
BM204_spring
80°N
60°N
40°N
20°N
0°
20°S
40°S
Latitude
20°W
0°
20°E
40°E
60°E
Longitude

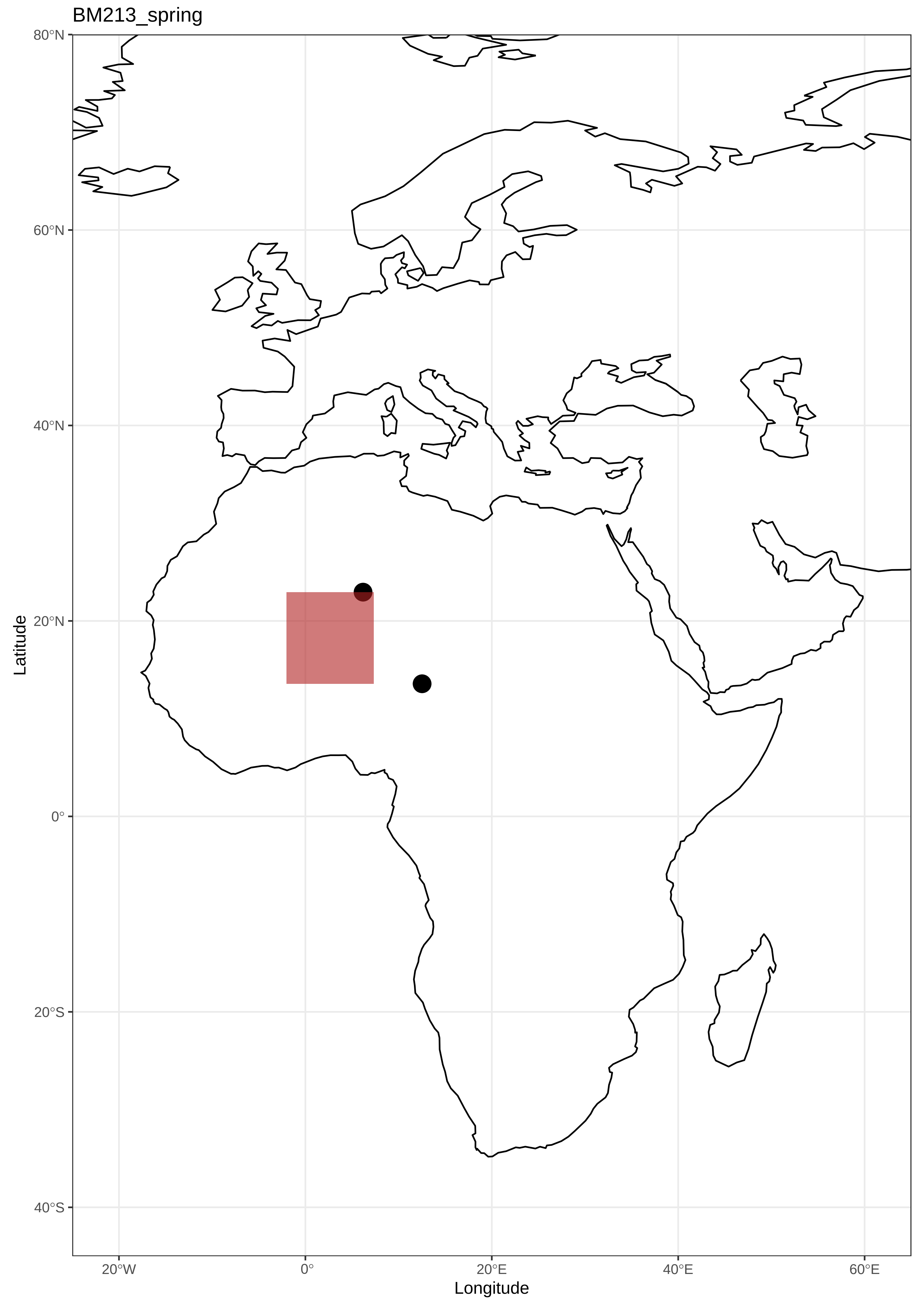
BM213_spring
80°N
60°N
40°N
20°N
0°
20°S
40°S
Latitude
20°W
0°
20°E
40°E
60°E
Longitude

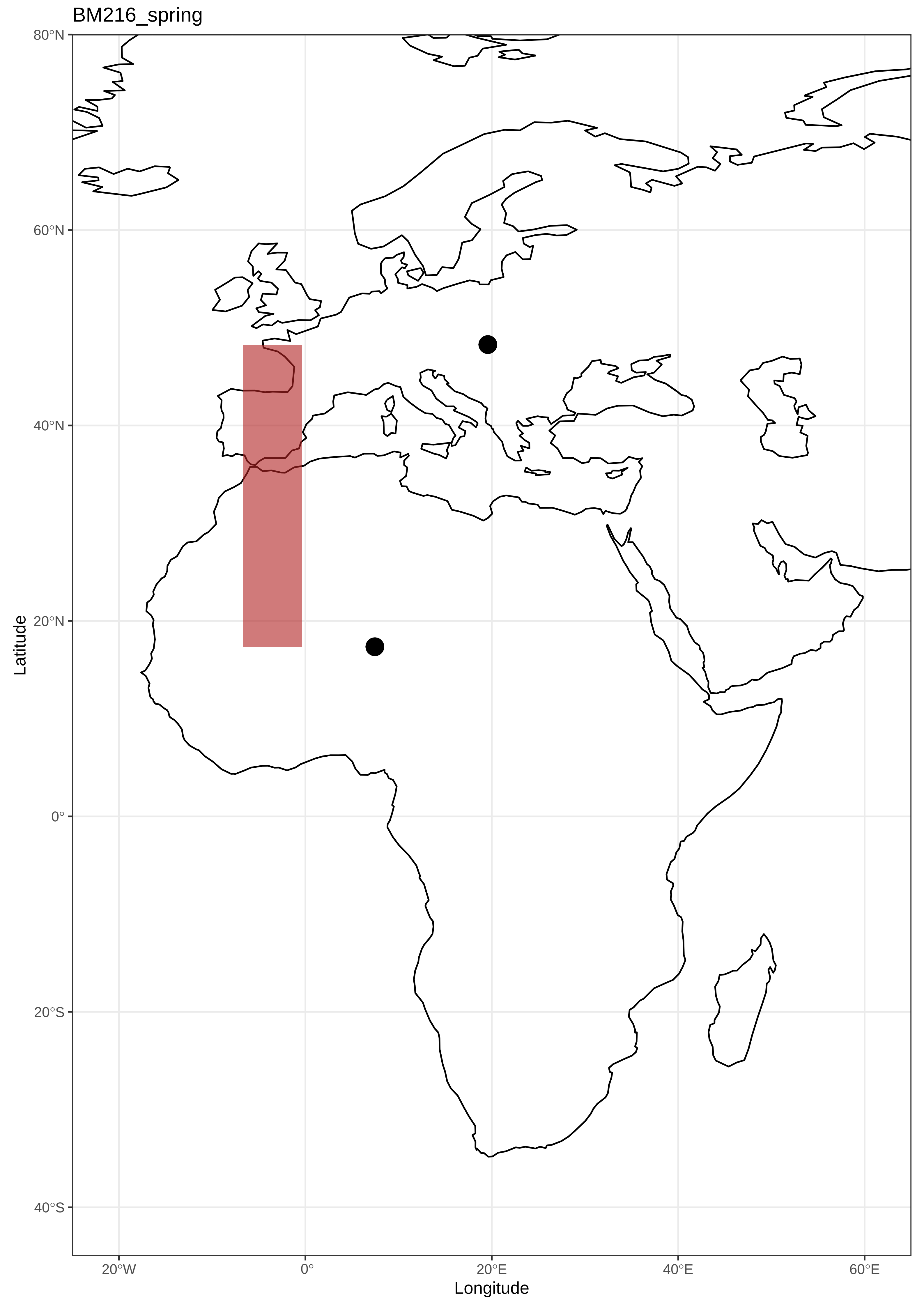
BM216_spring
80°N
60°N
40°N
20°N
0°
20°S
40°S
Latitude
20°W
0°
20°E
40°E
60°E
Longitude

BM223_spring

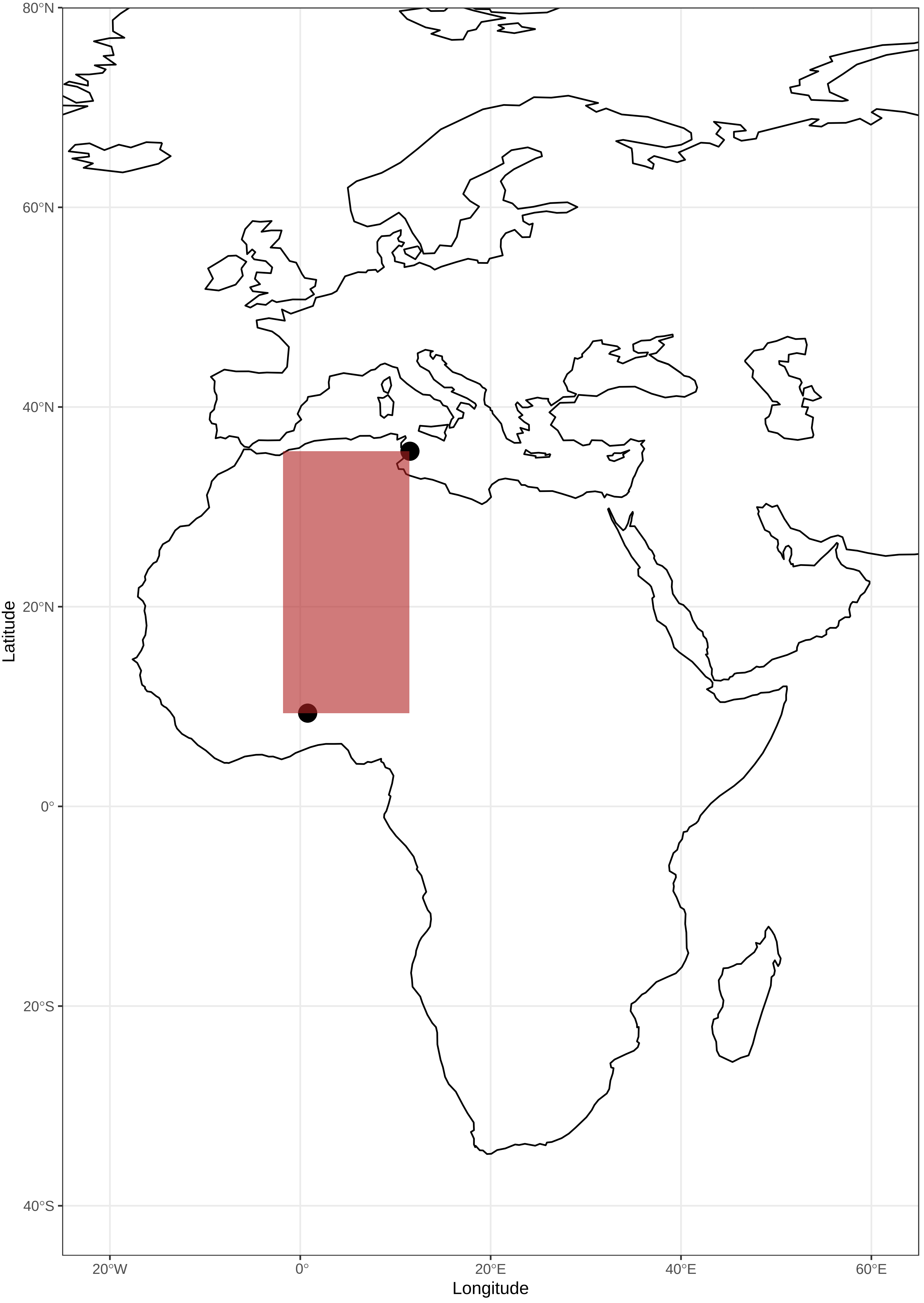

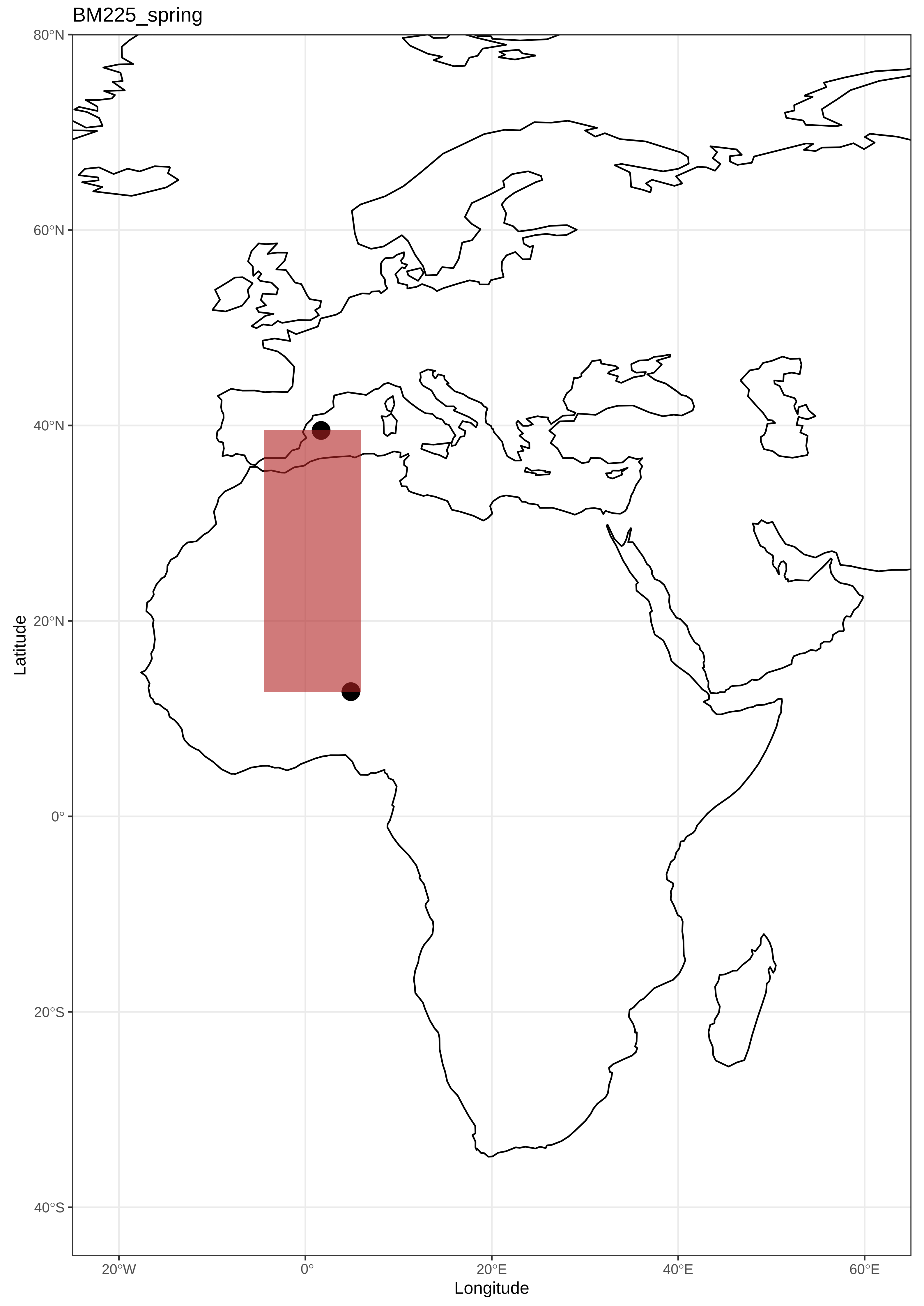
BM225_spring
80°N
60°N
40°N
20°N
0°
20°S
40°S
Latitude
20°W
0°
20°E
40°E
60°E
Longitude

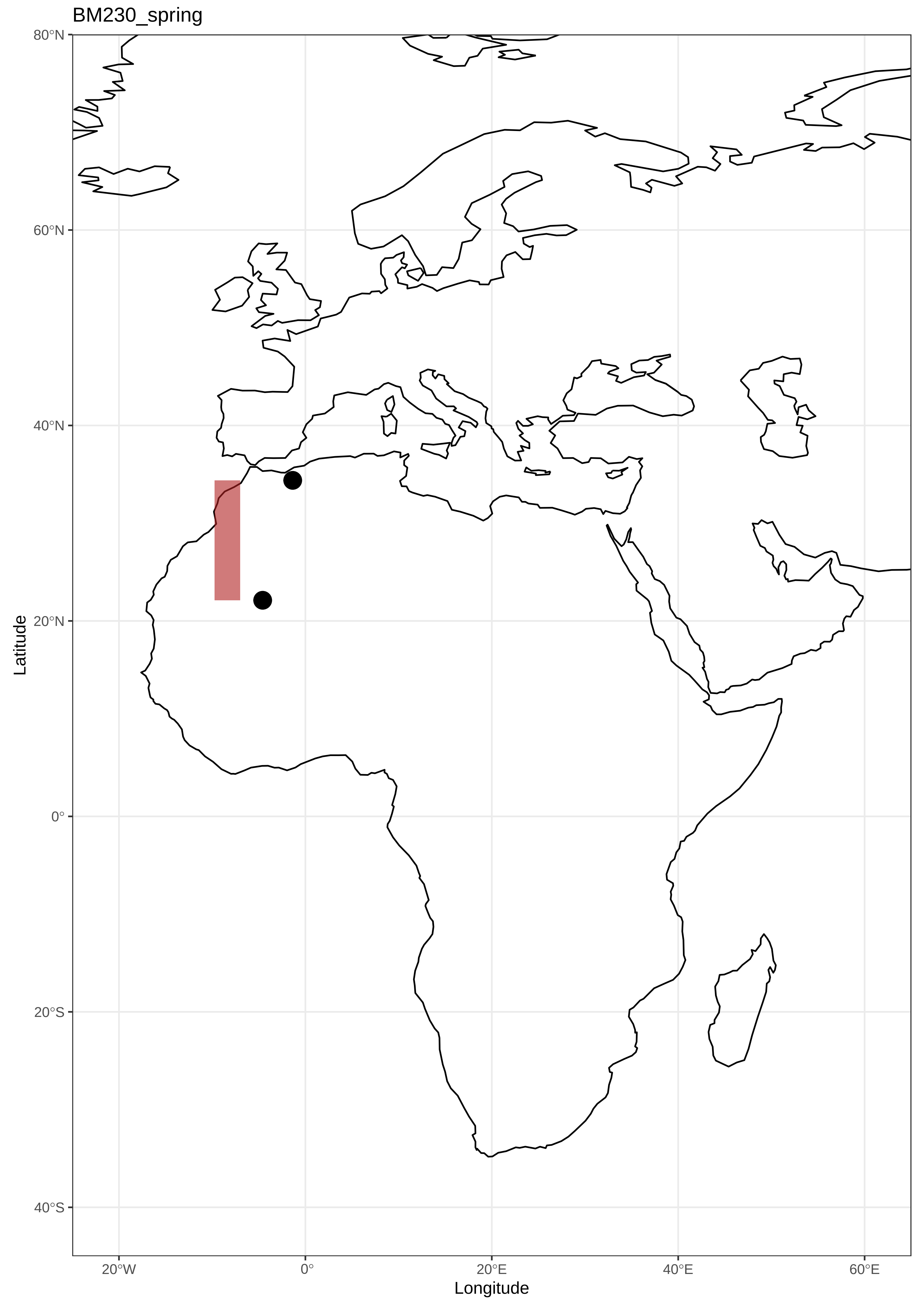

BM230_spring
80°N
60°N
40°N
20°N
0°
20°S
40°S
20°W
0°
20°E
40°E
60°E
Longitude
Latitude

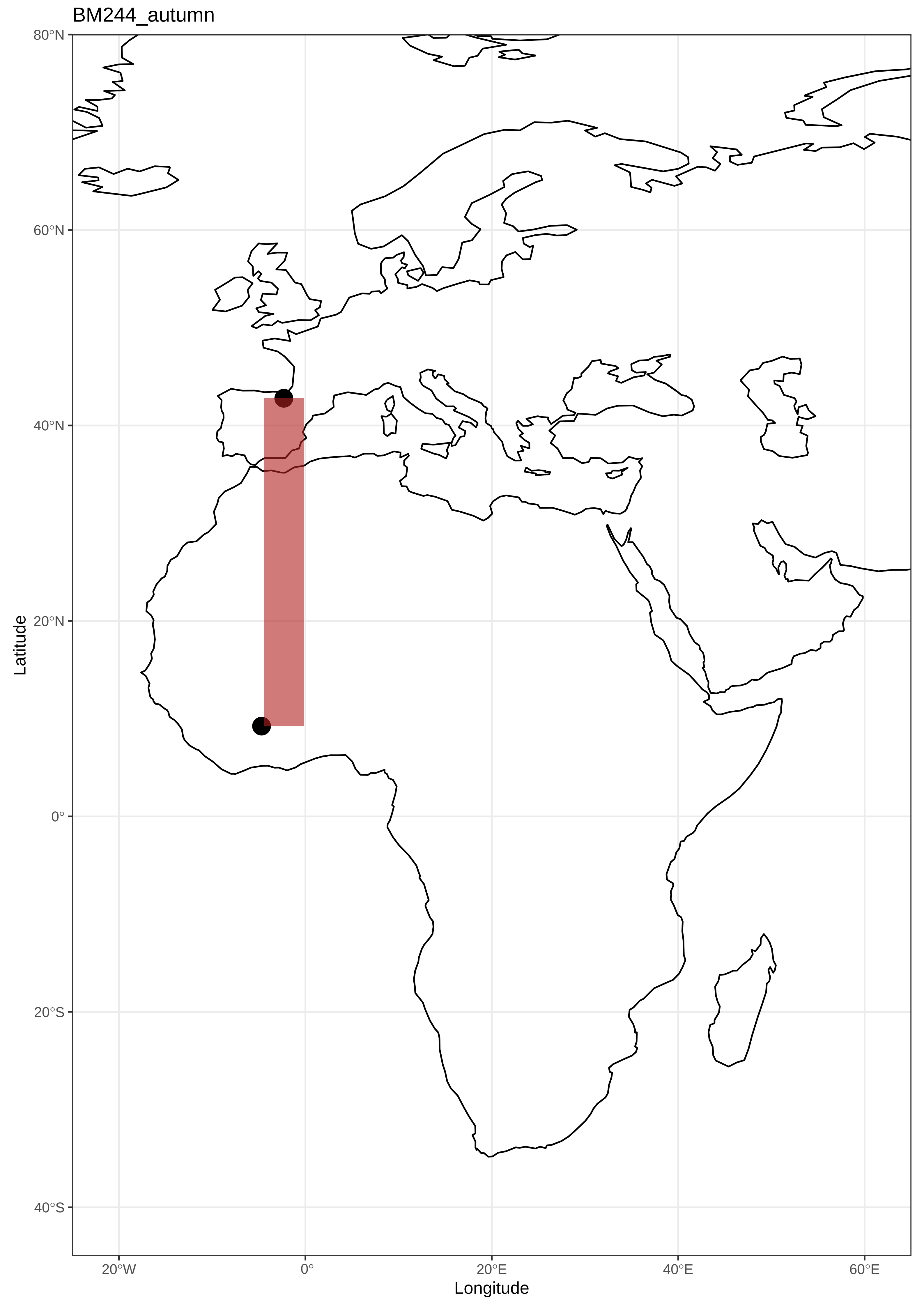
BM244_autumn
80°N
60°N
40°N
20°N
0°
20°S
40°S
Latitude
20°W
0°
20°E
40°E
60°E
Longitude

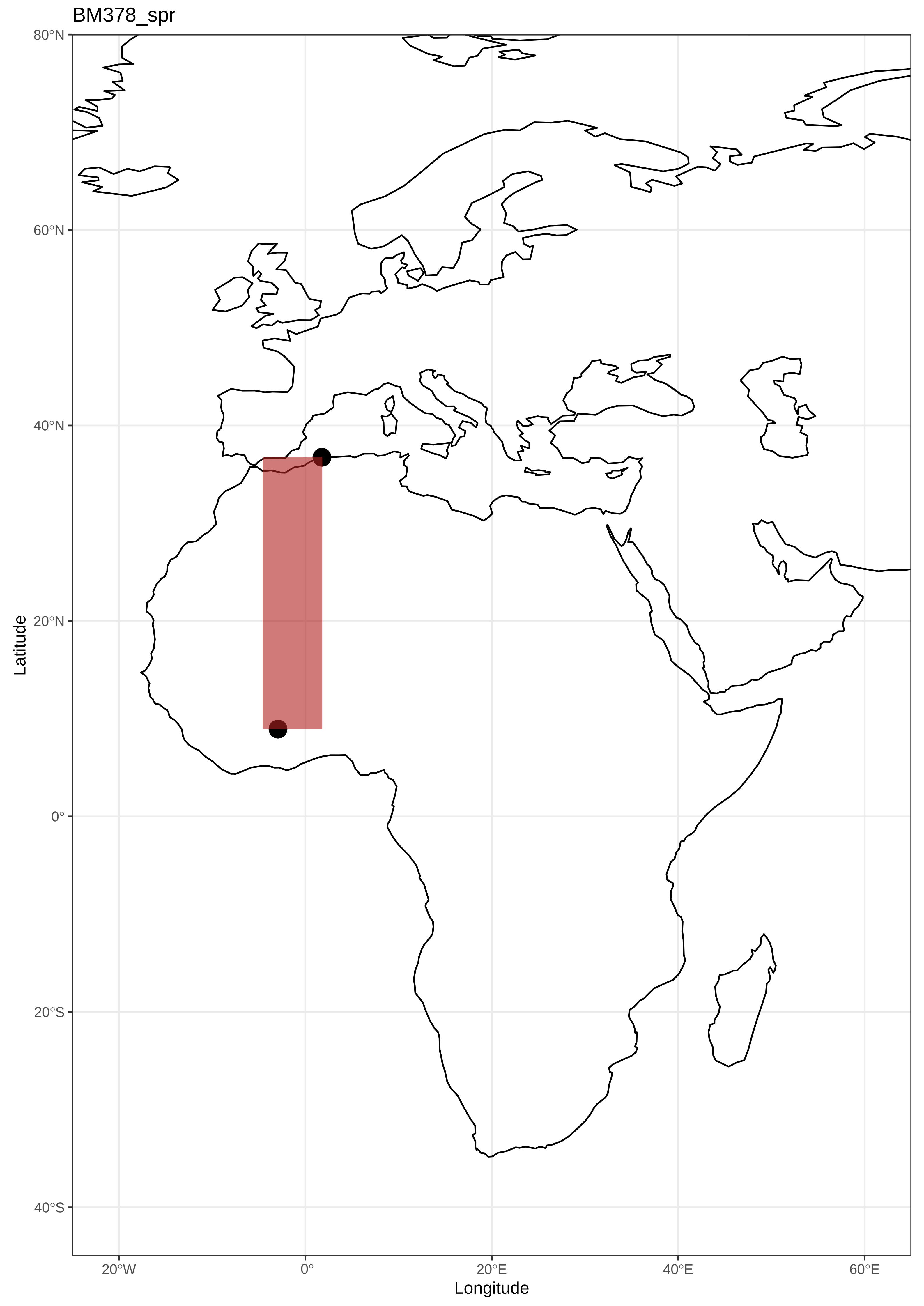
BM378_spr
80°N
60°N
40°N
20°N
0°
20°S
40°S
Latitude
20°W
0°
20°E
40°E
60°E
Longitude

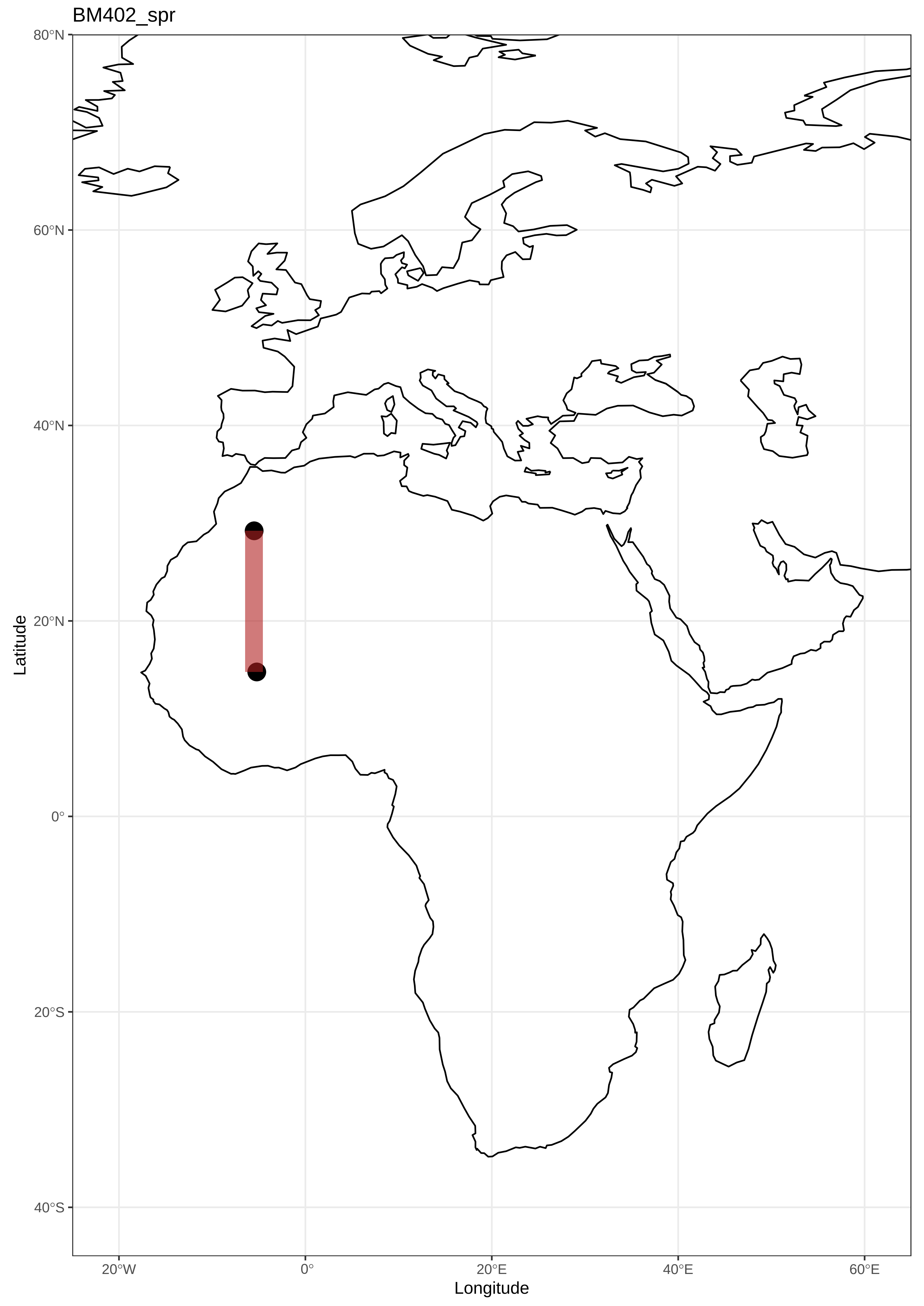

BM402_spr
80°N
60°N
40°N
20°N
0°
20°S
40°S
20°W
0°
20°E
40°E
60°E
Longitude
Latitude

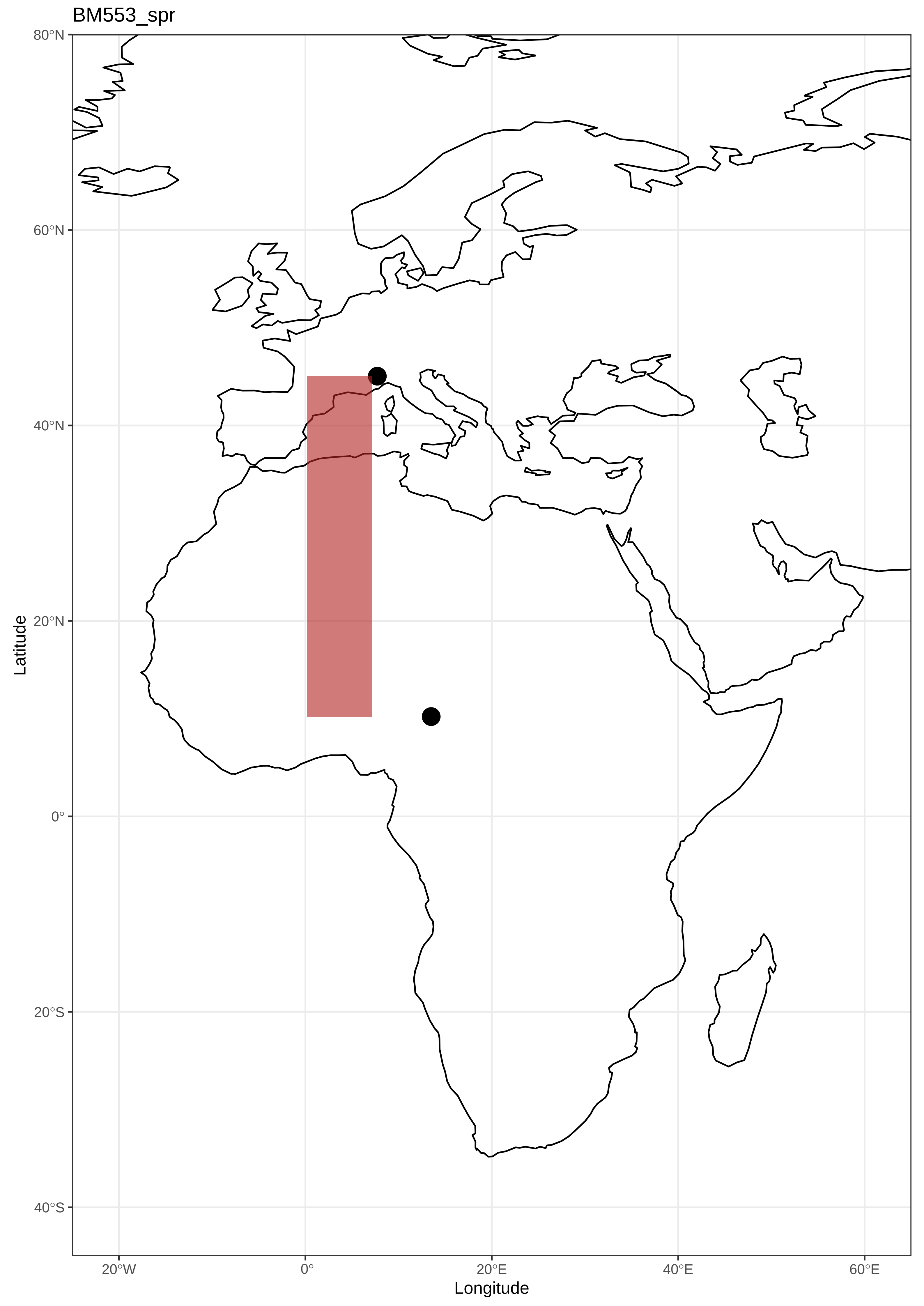
BM553_spr
Latitude
Longitude
80°N
60°N
40°N
20°N
0°
20°S
40°S
20°W
0°
20°E
40°E
60°E

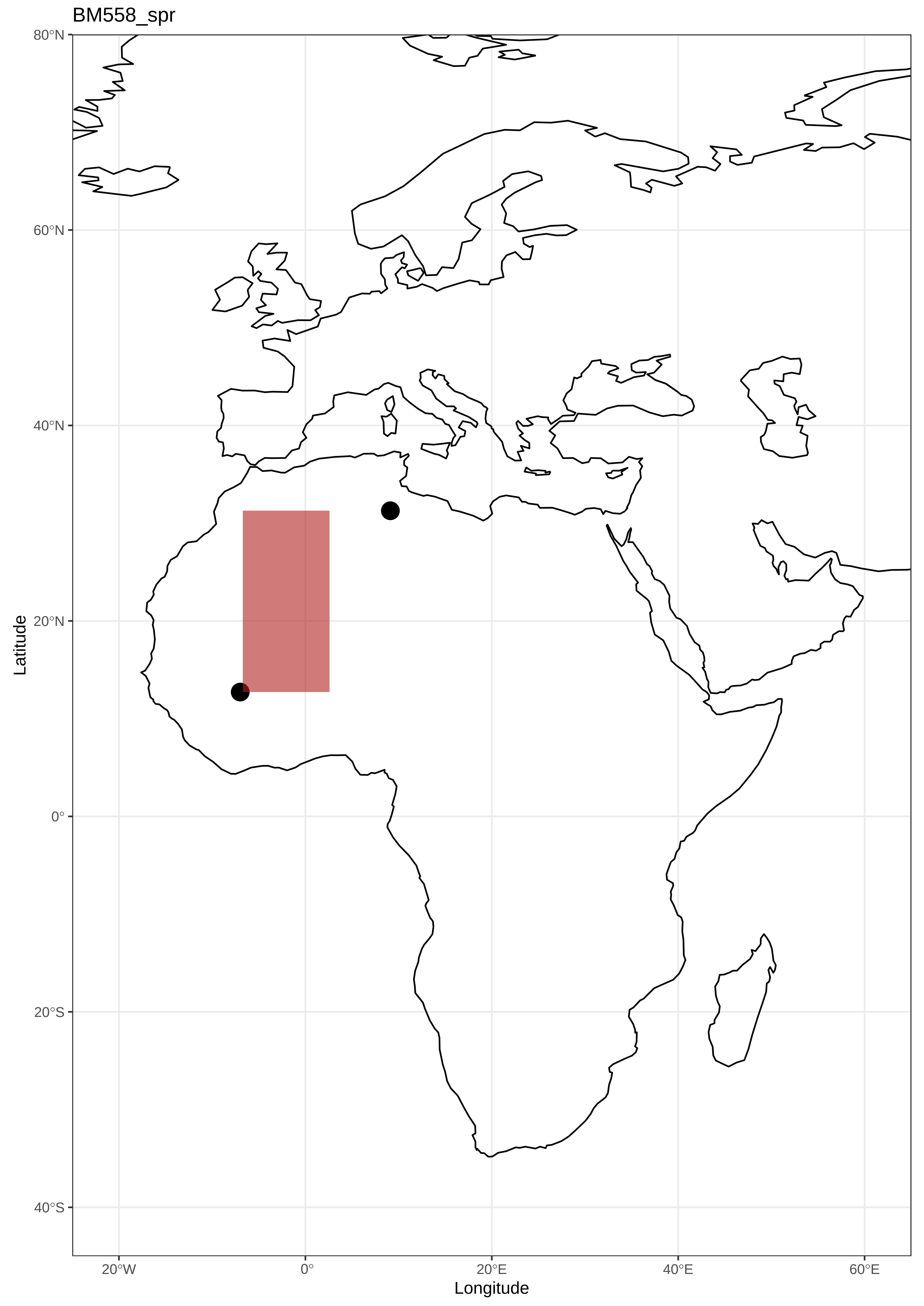
BM558_spr
80°N
60°N
40°N
20°N
0°
20°S
40°S
Latitude
20°W
0°
20°E
40°E
60°E
Longitude

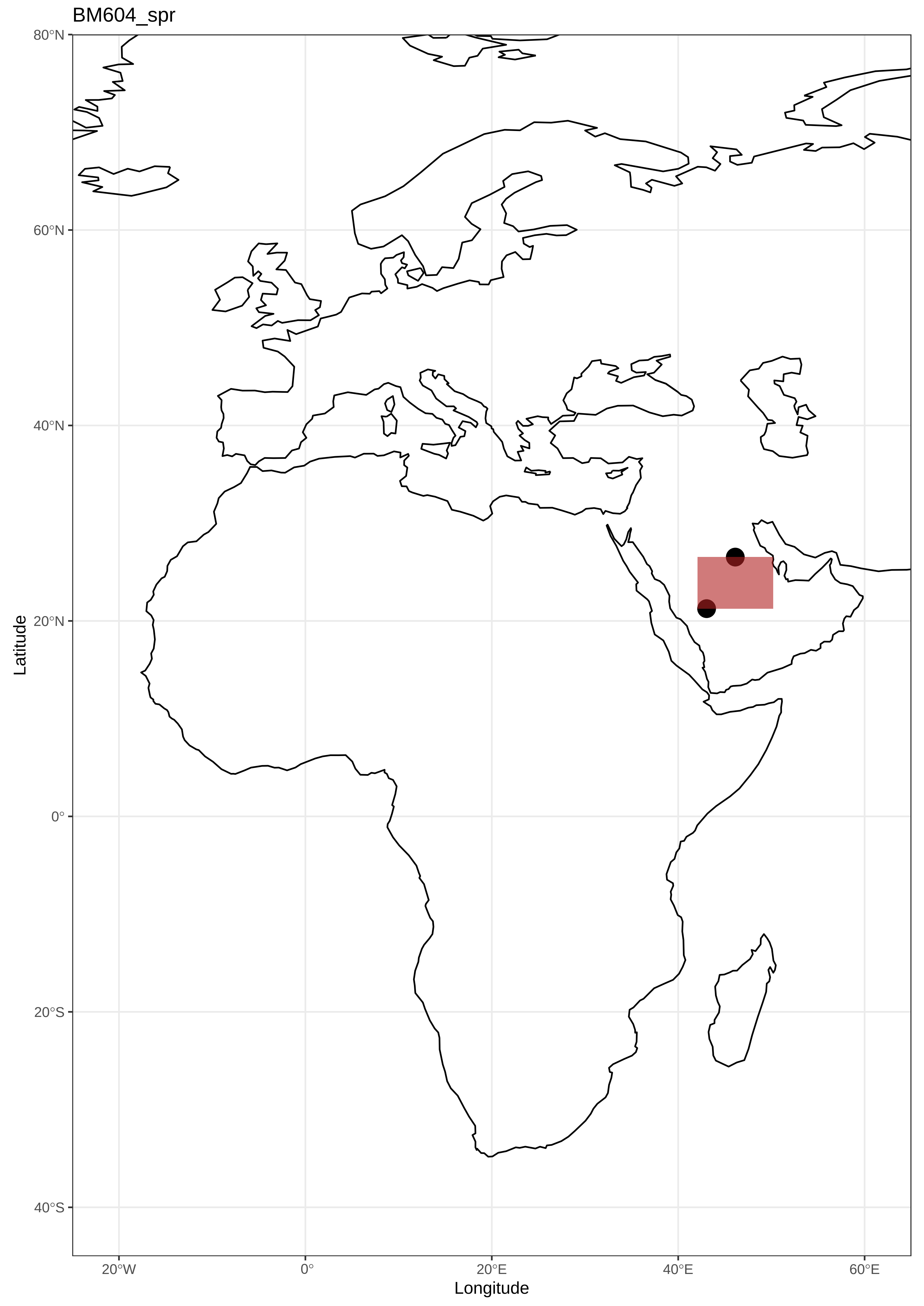

BM604_spr
80°N
60°N
40°N
20°N
0°
20°S
40°S
Latitude
20°W
0°
20°E
40°E
60°E
Longitude

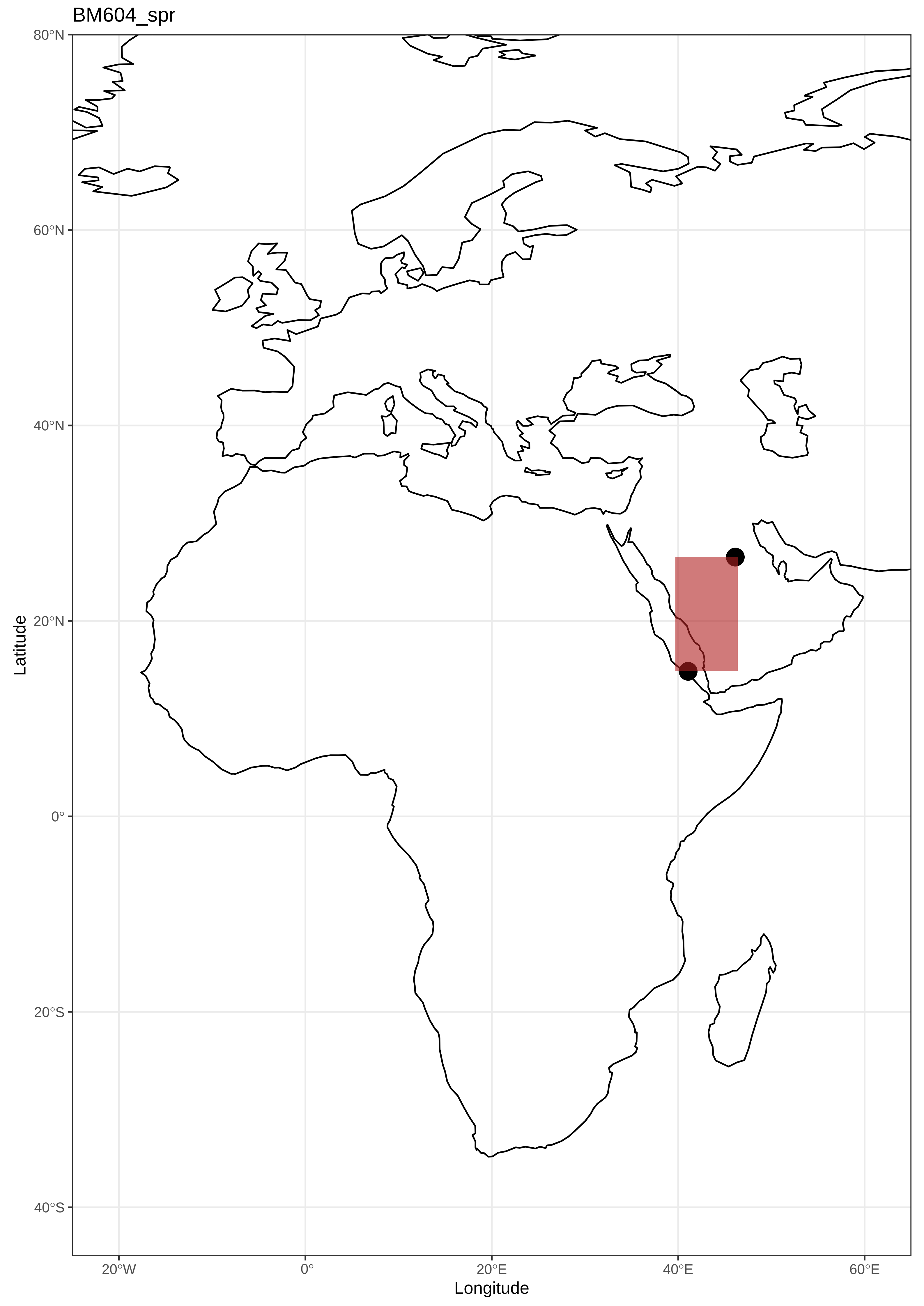
BM604_spr
80°N
60°N
40°N
20°N
0°
20°S
40°S
Latitude
20°W
0°
20°E
40°E
60°E
Longitude

BM648_spr

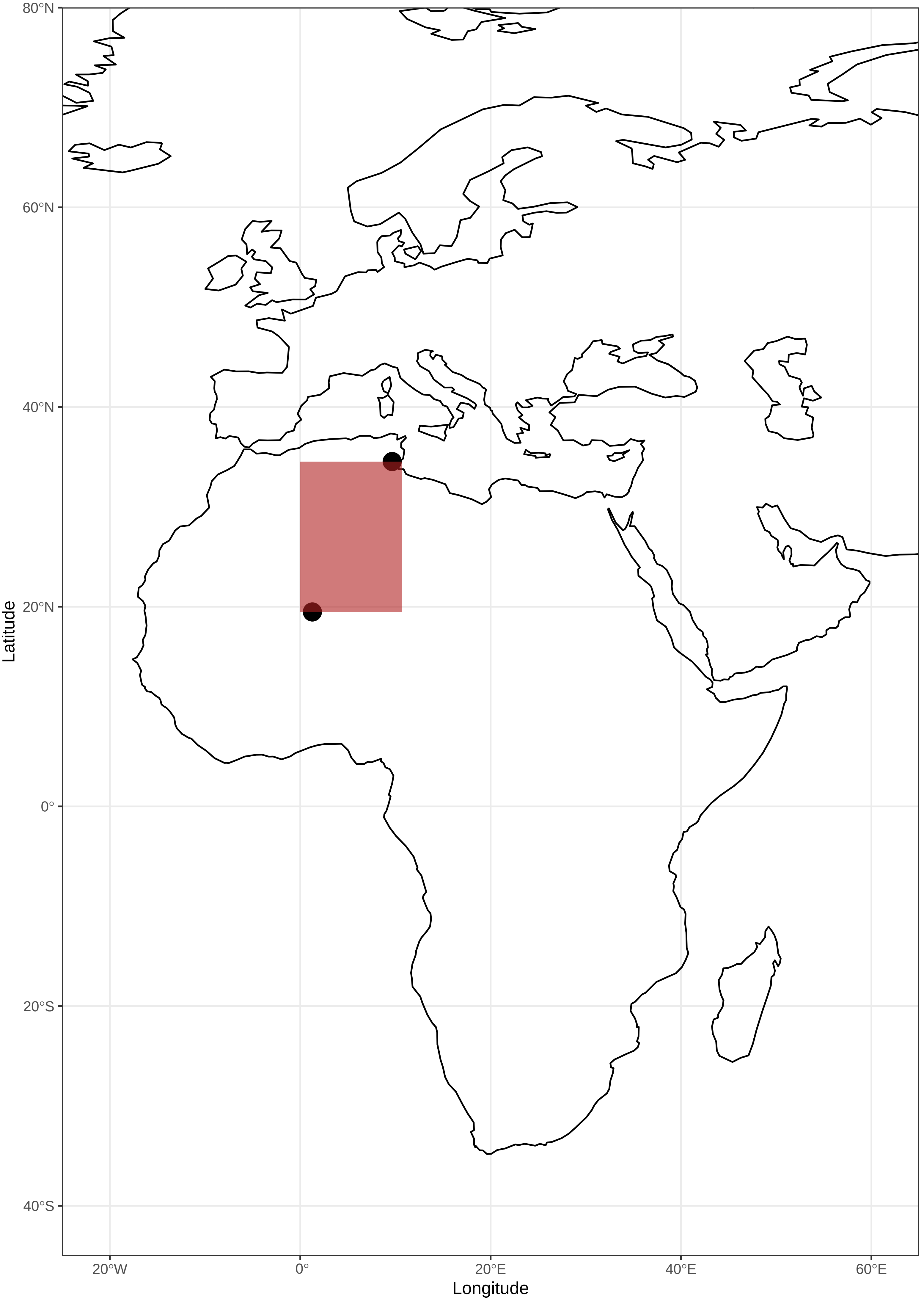

BM653_spr

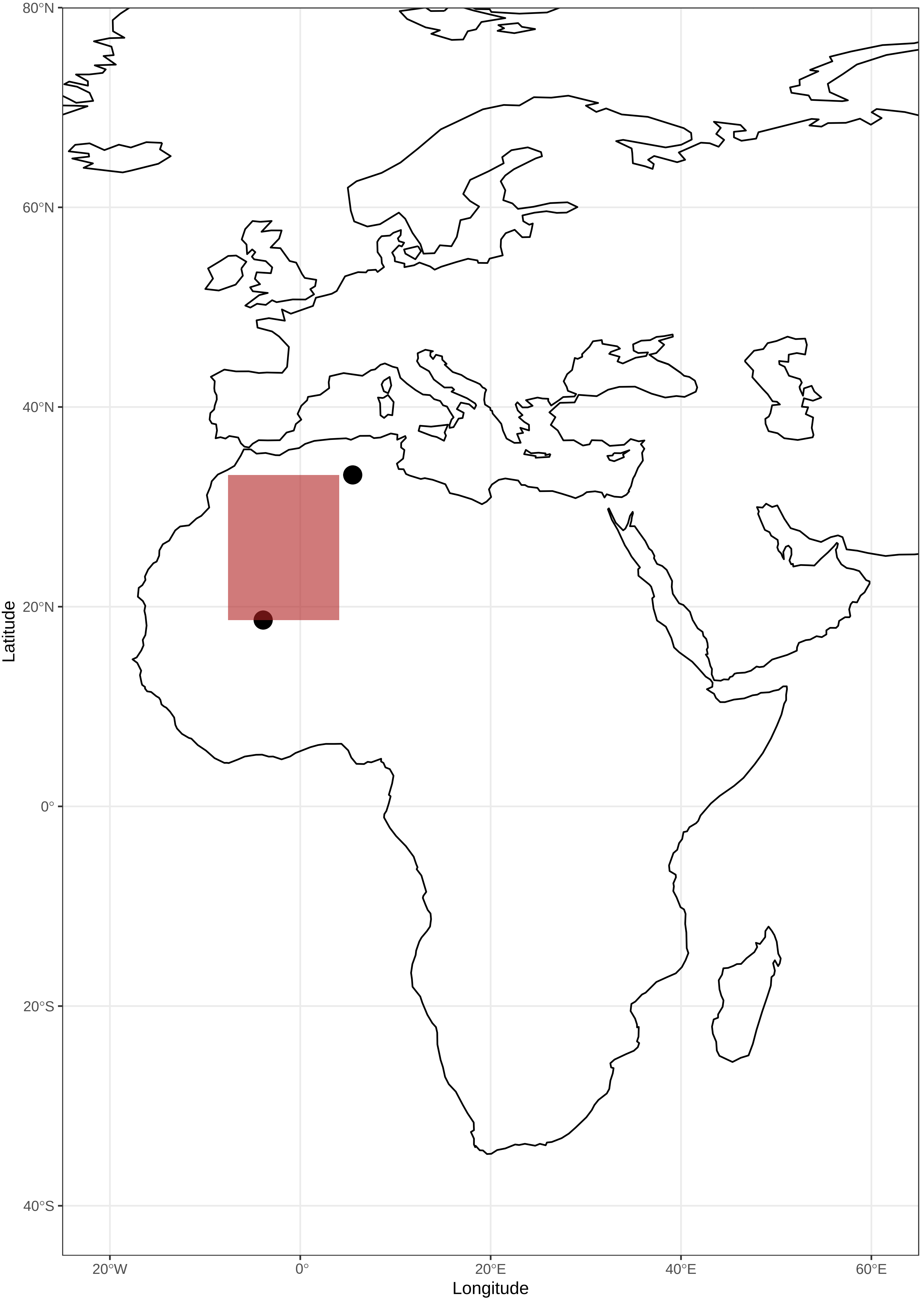

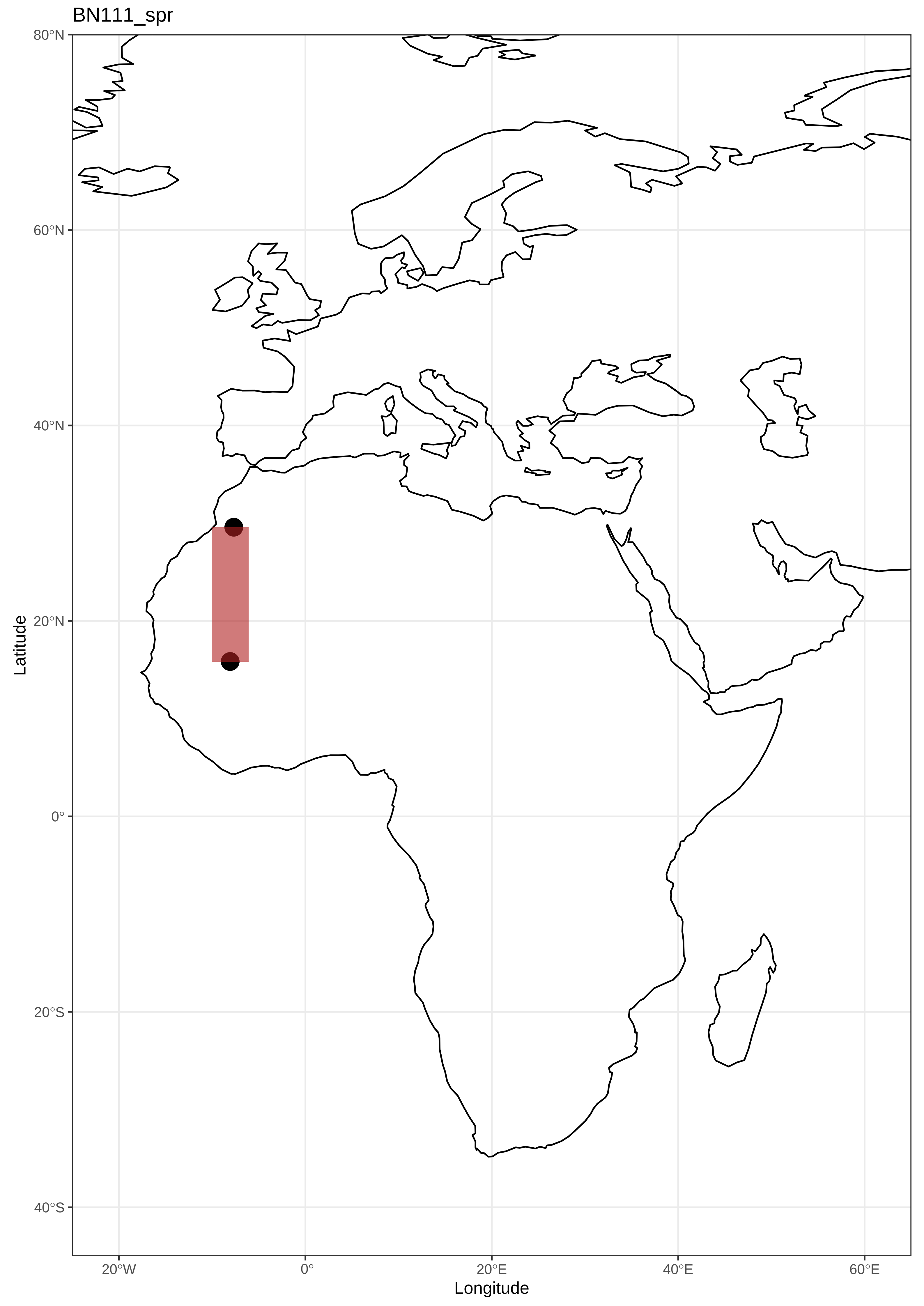
BN111_spr
80°N
60°N
40°N
20°N
0°
20°S
40°S
20°W
0°
20°E
40°E
60°E
Latitude
Longitude

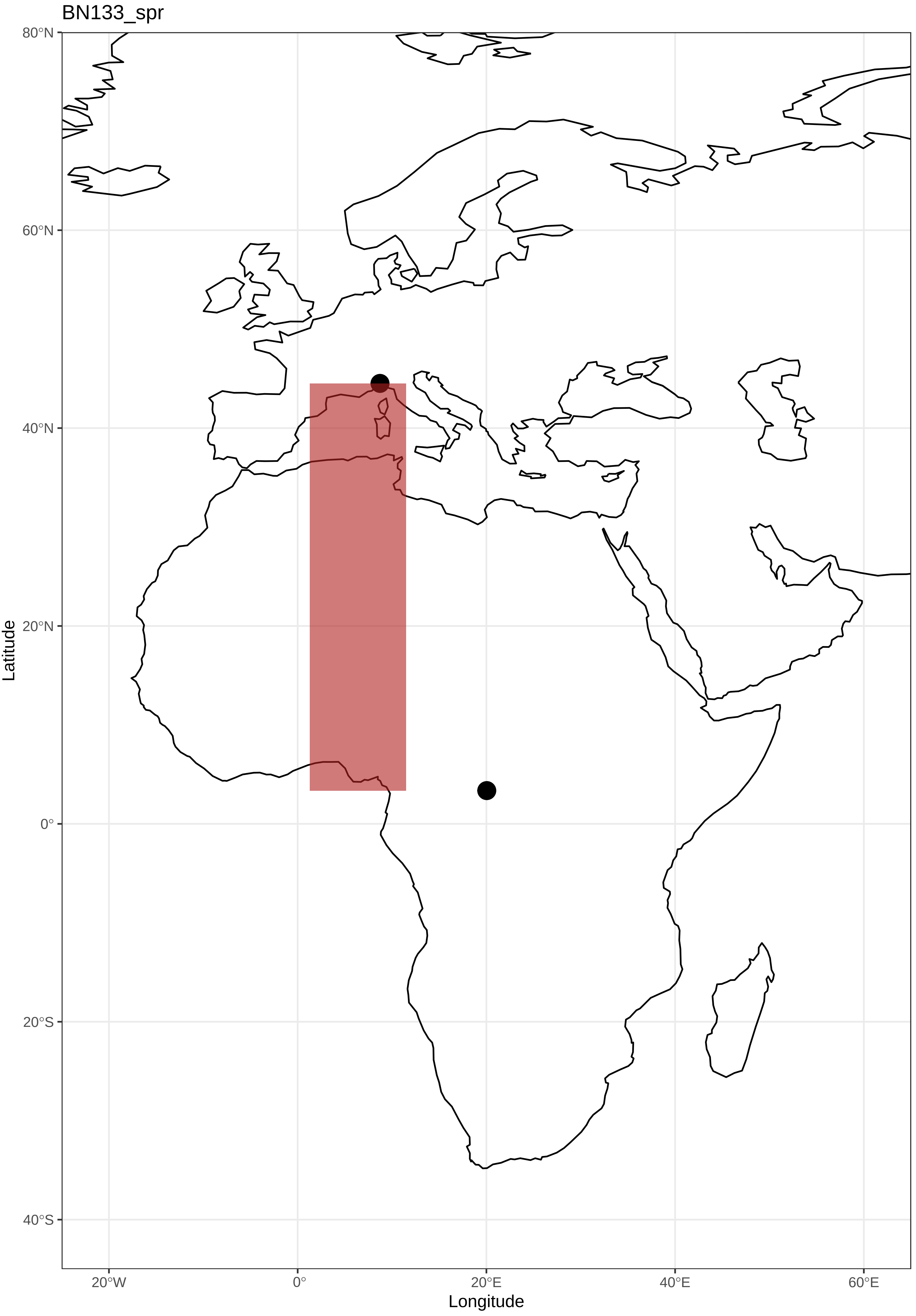

BN133_spr
80°N
60°N
40°N
20°N
0°
20°S
40°S
20°W
0°
20°E
40°E
60°E
Latitude
Longitude

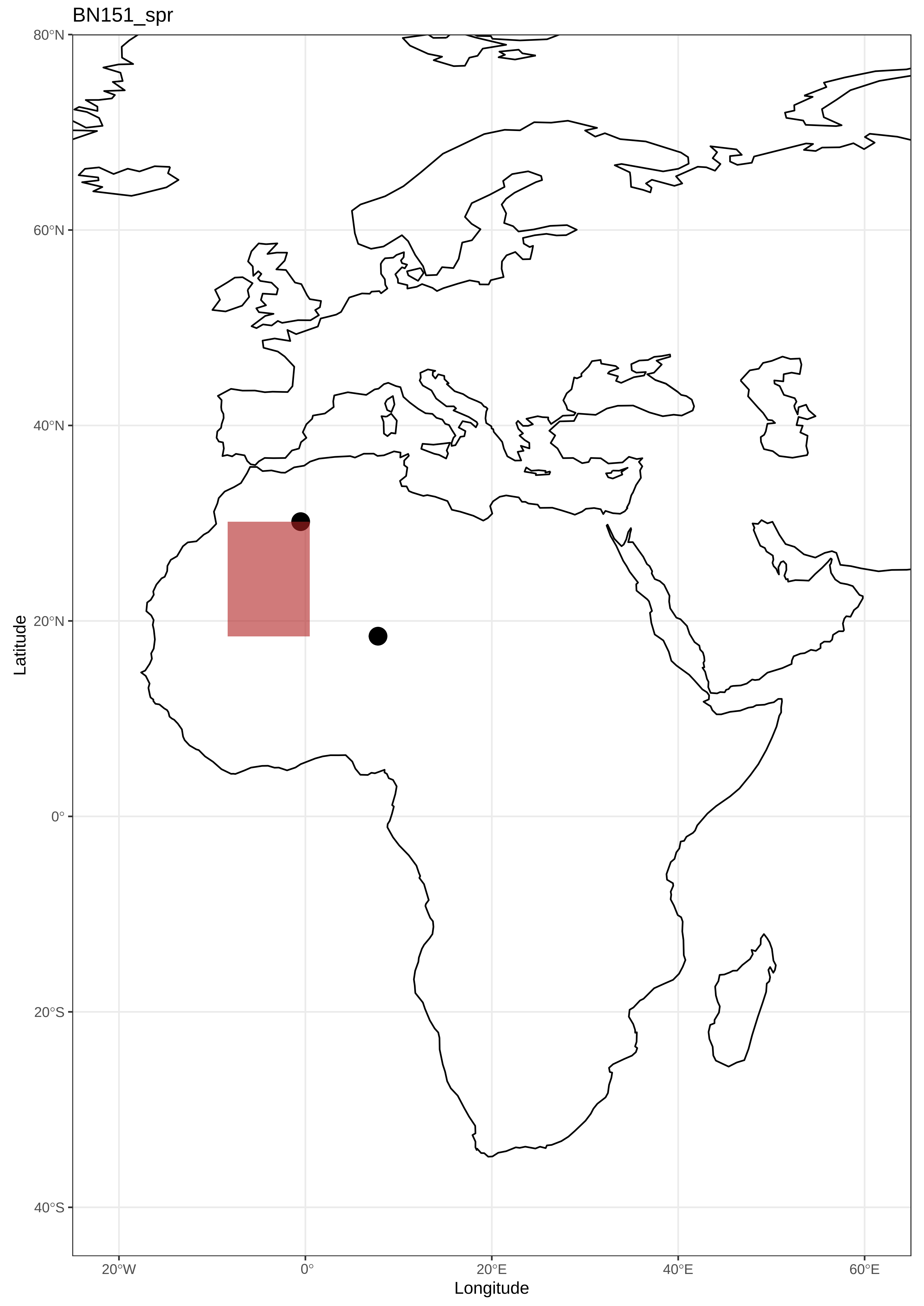
BN151_spr
80°N
60°N
40°N
20°N
0°
20°S
40°S
Latitude
20°W
0°
20°E
40°E
60°E
Longitude

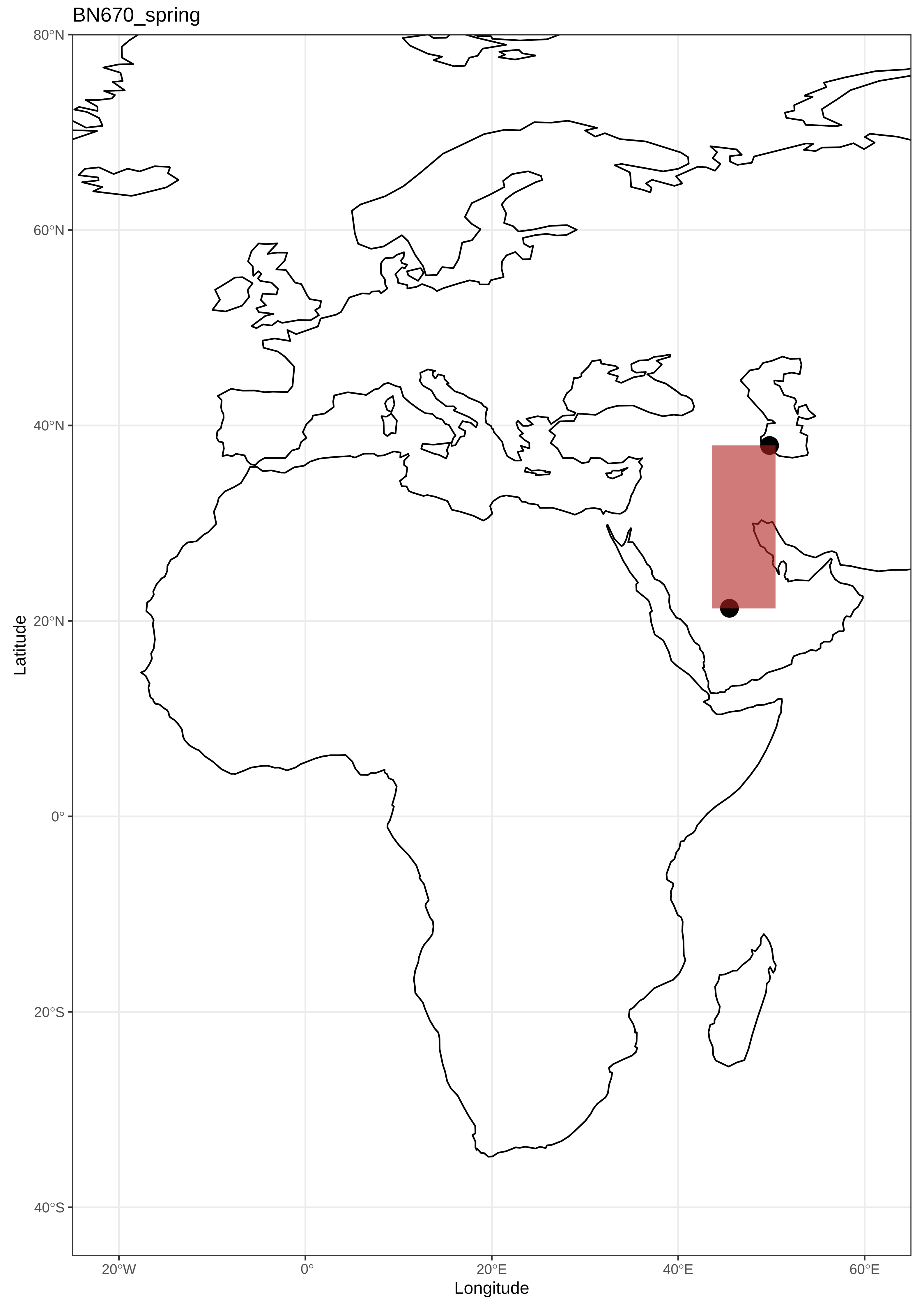
BN670_spring
80°N
60°N
40°N
20°N
0°
20°S
40°S
Latitude
20°W
0°
20°E
40°E
60°E
Longitude

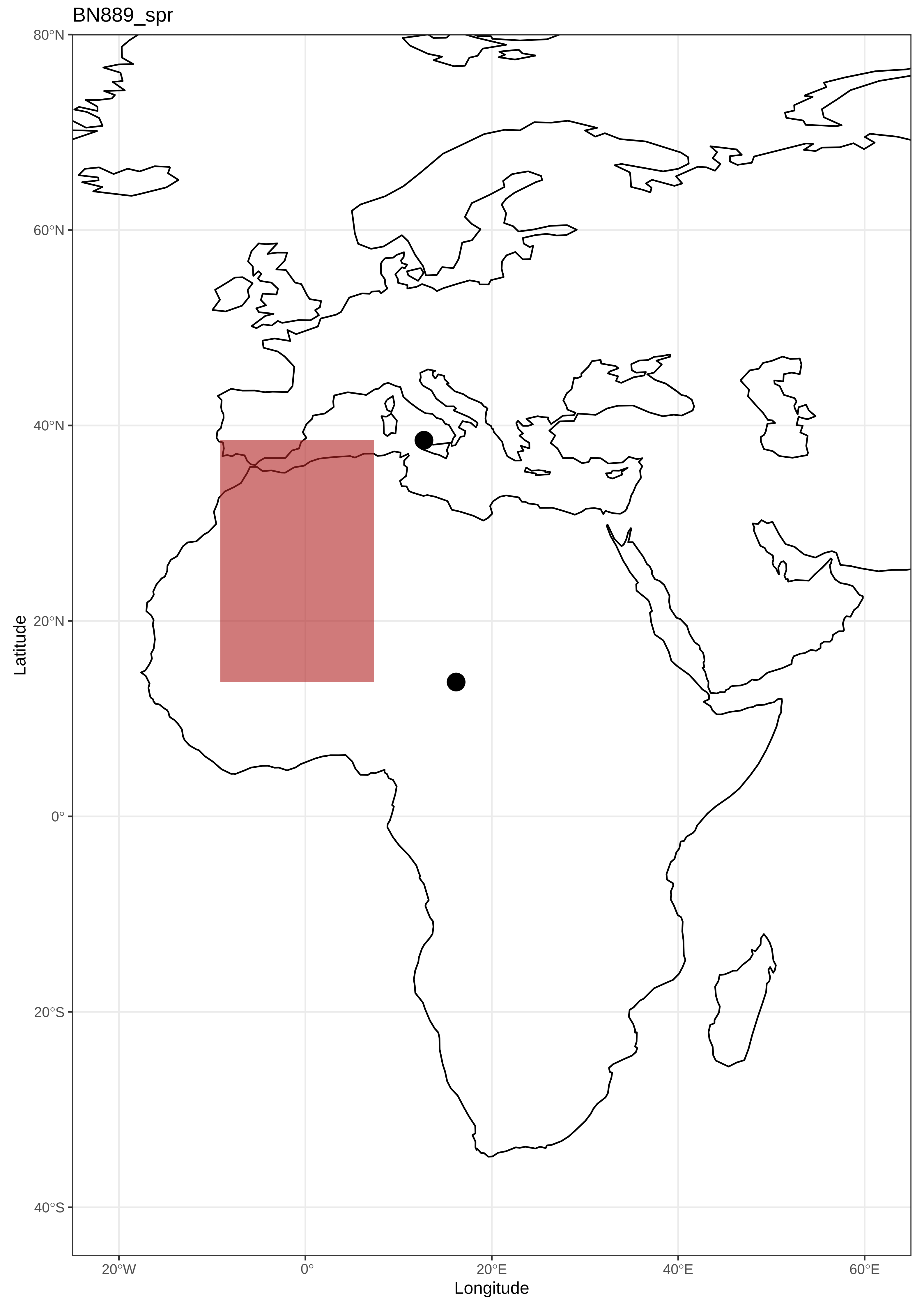
BN889_spr
80°N
60°N
40°N
20°N
0°
20°S
40°S
20°W
0°
20°E
40°E
60°E
Longitude
Latitude

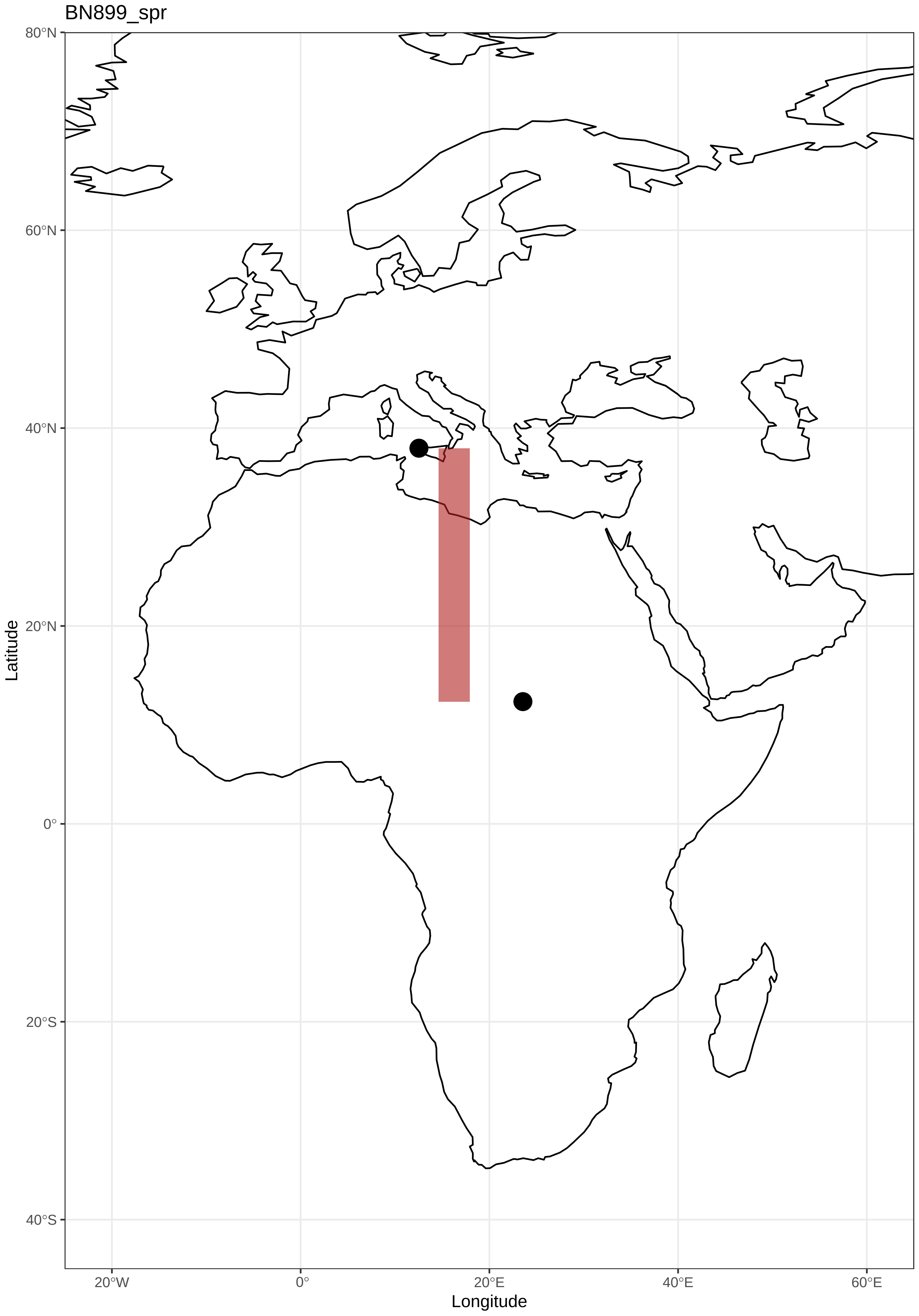
BN899_spr
80°N
60°N
40°N
20°N
0°
20°S
40°S
Latitude
20°W
0°
20°E
40°E
60°E
Longitude

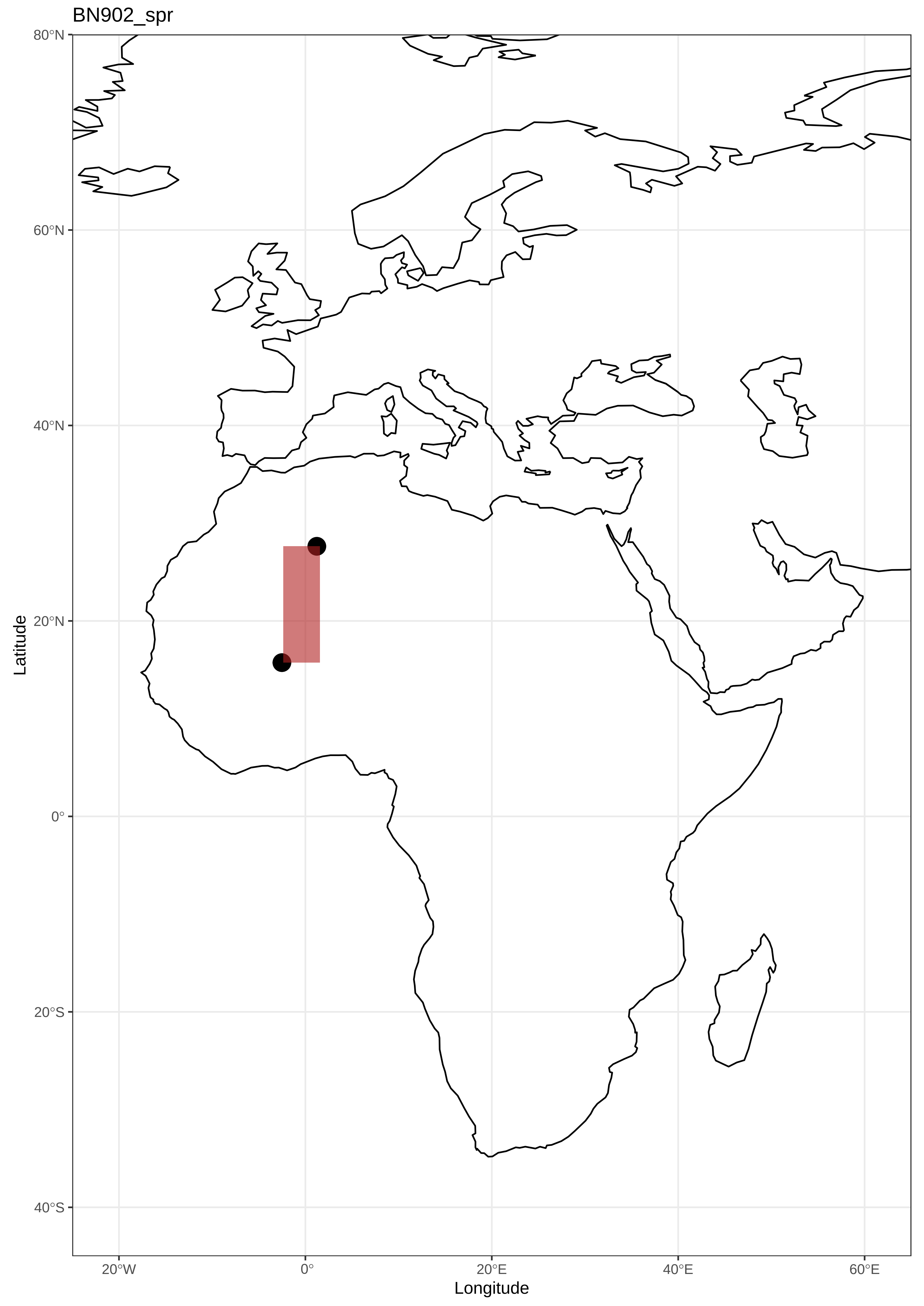
BN902_spr
80°N
60°N
40°N
20°N
0°
20°S
40°S
Latitude
20°W
0°
20°E
40°E
60°E
Longitude

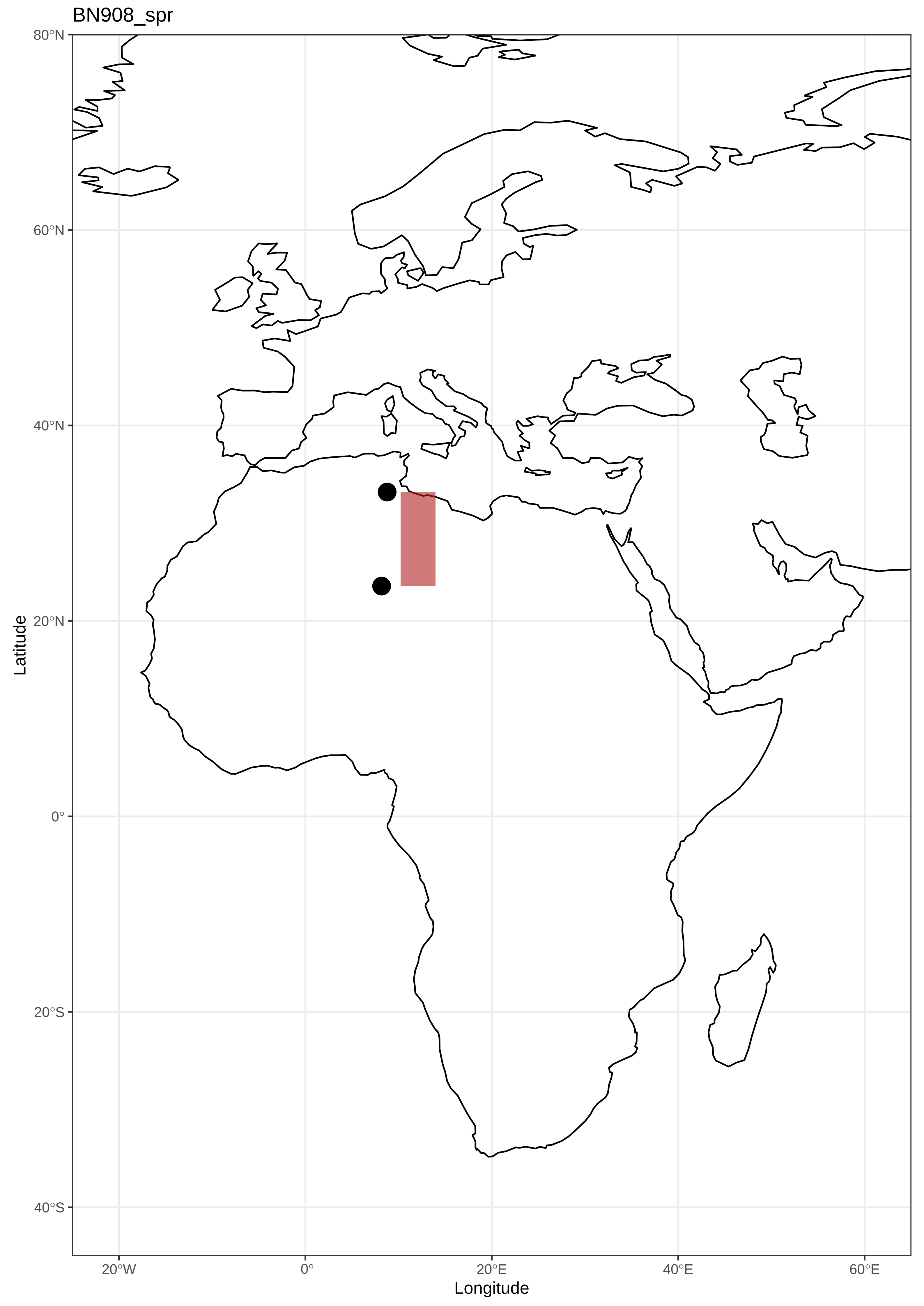
BN908_spr
80°N
60°N
40°N
20°N
0°
20°S
40°S
Latitude
20°W
0°
20°E
40°E
60°E
Longitude

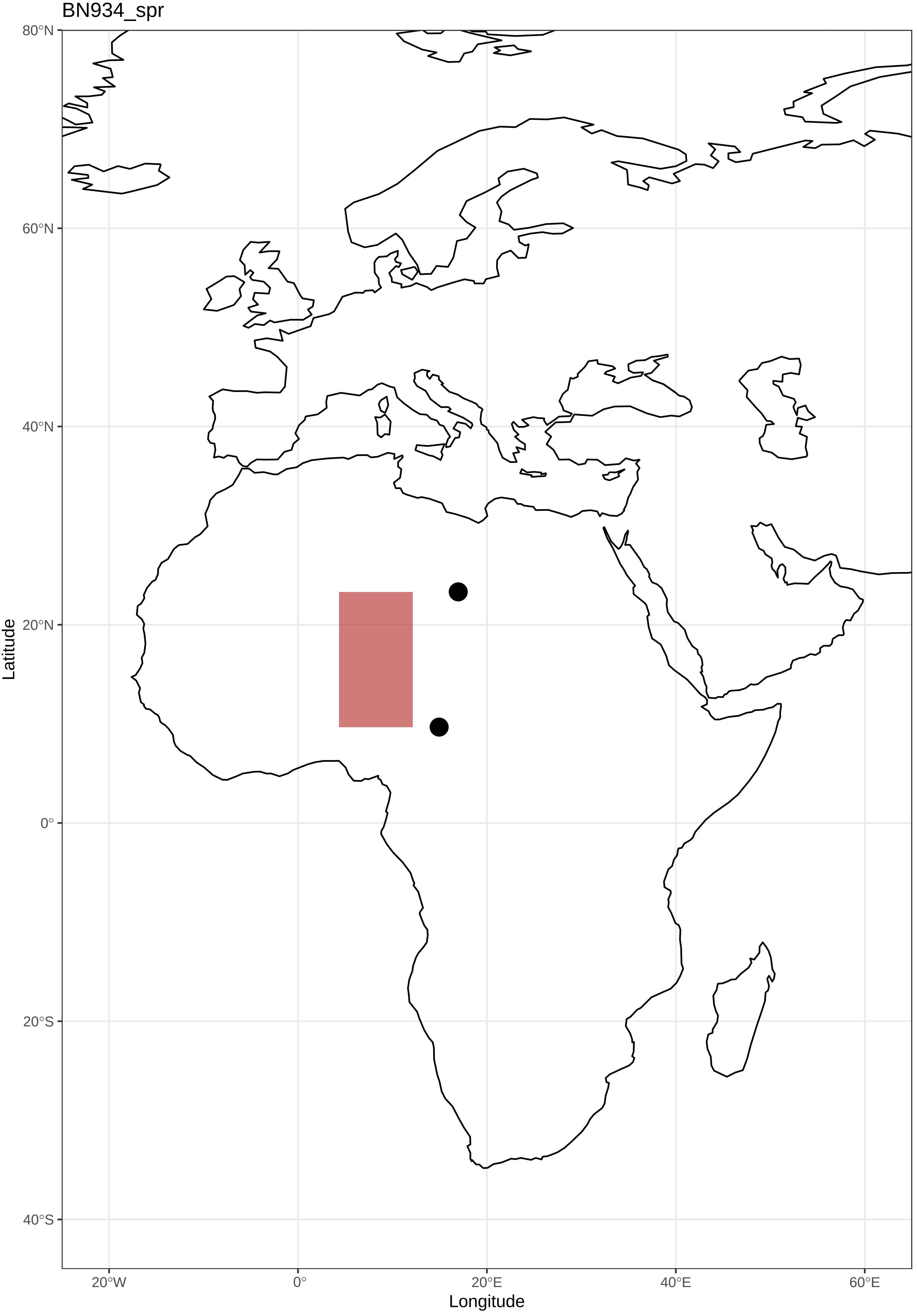

BN934_spr
80°N
60°N
40°N
20°N
0°
20°S
40°S
Latitude
20°W
0°
20°E
40°E
60°E
Longitude

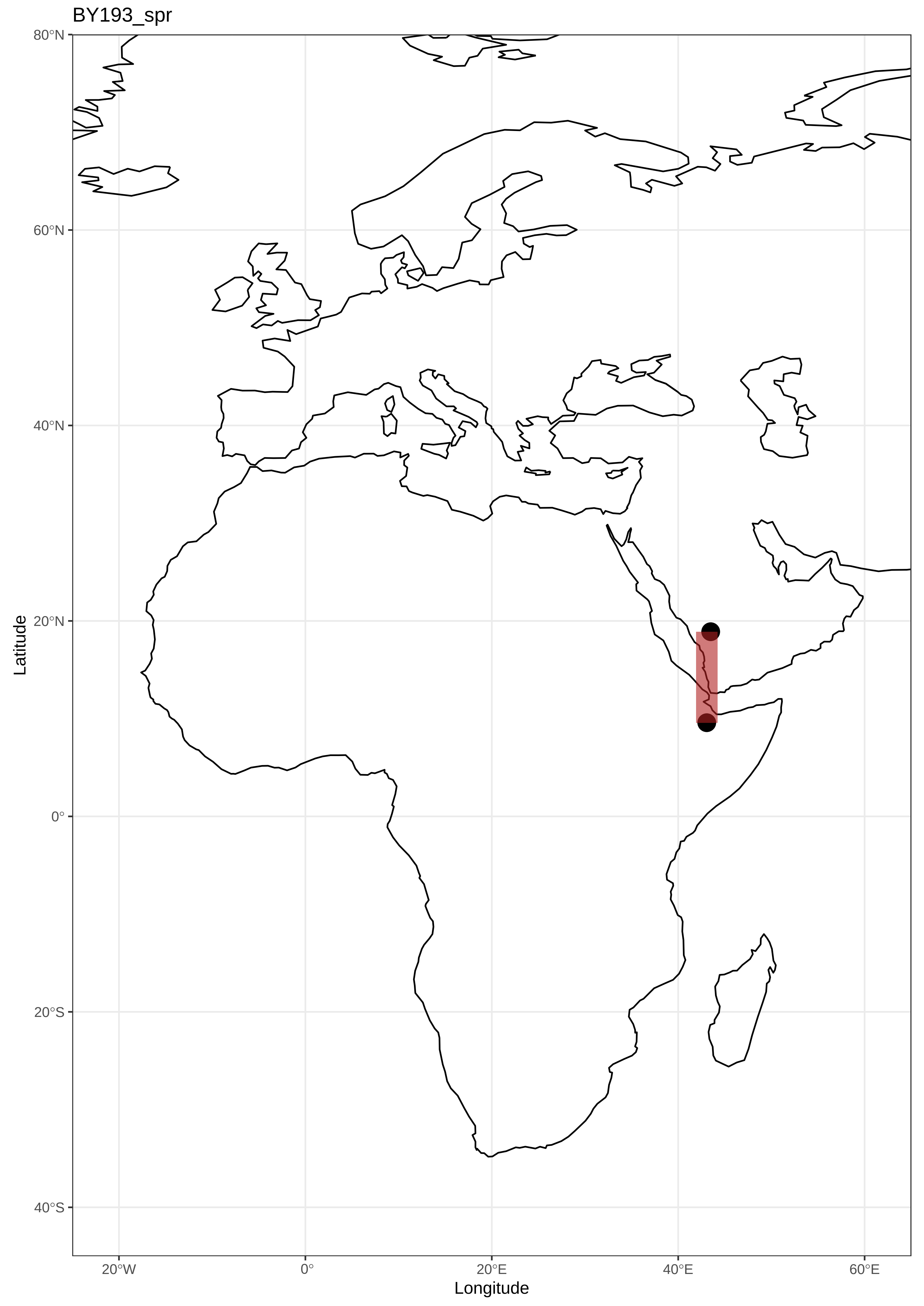
BY193_spr
80°N
60°N
40°N
20°N
0°
20°S
40°S
Latitude
20°W
0°
20°E
40°E
60°E
Longitude

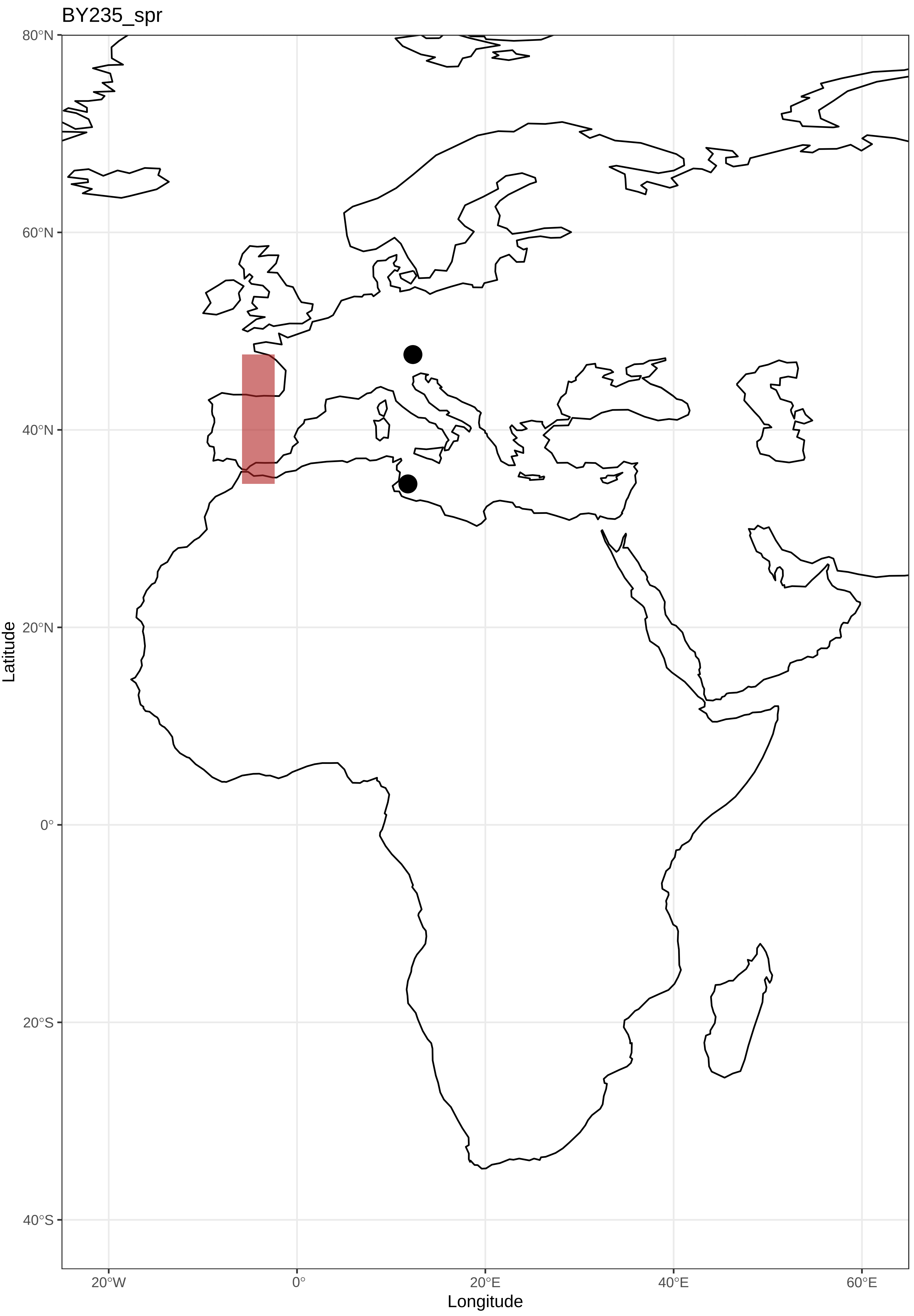

BY235_spr
80°N
60°N
40°N
20°N
0°
20°S
40°S
Latitude
20°W
0°
20°E
40°E
60°E
Longitude

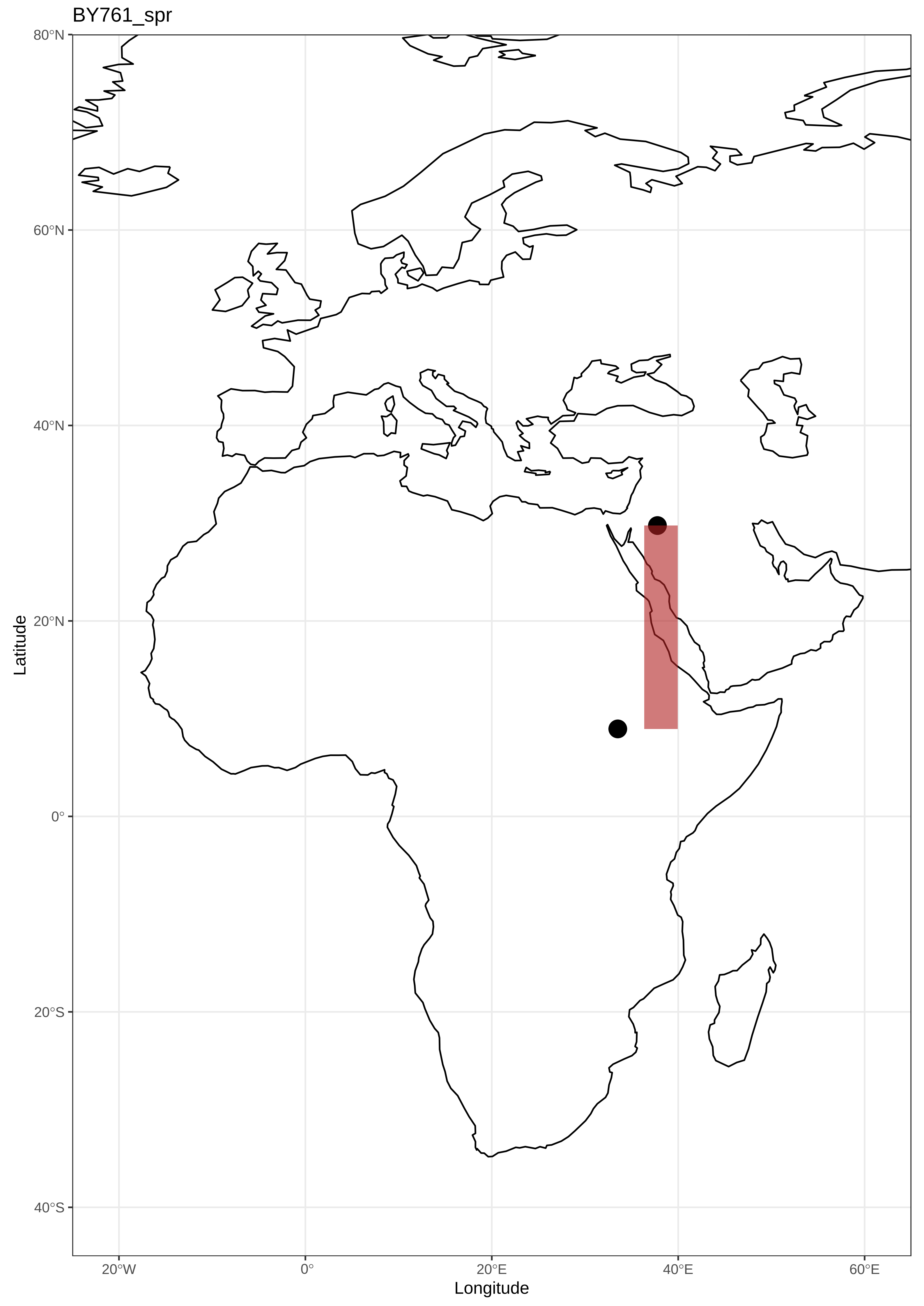

BY761_spr
80°N
60°N
40°N
20°N
0°
20°S
40°S
Latitude
20°W
0°
20°E
40°E
60°E
Longitude

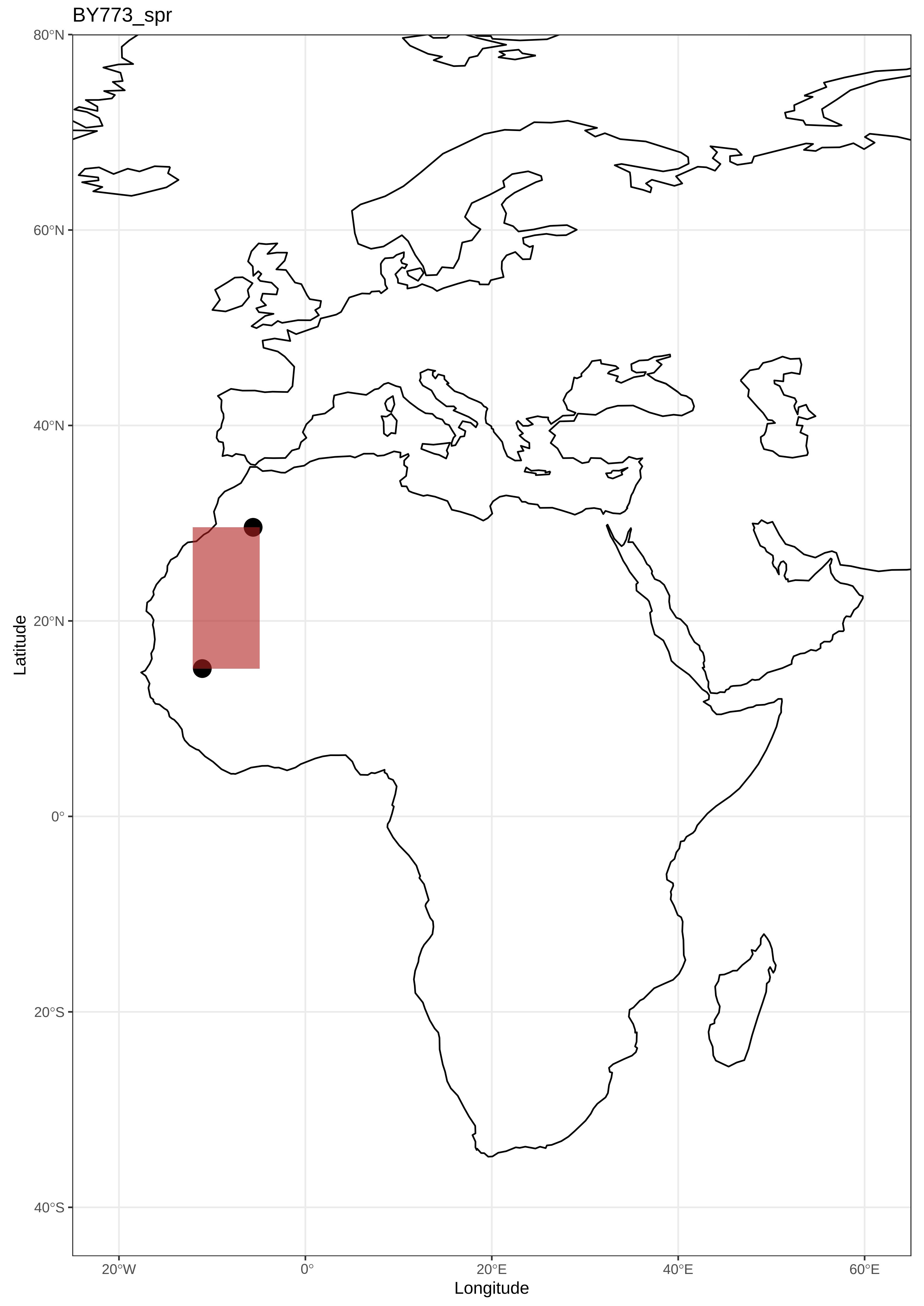
BY773_spr
80°N
60°N
40°N
20°N
0°
20°S
40°S
Latitude
20°W
0°
20°E
40°E
60°E
Longitude

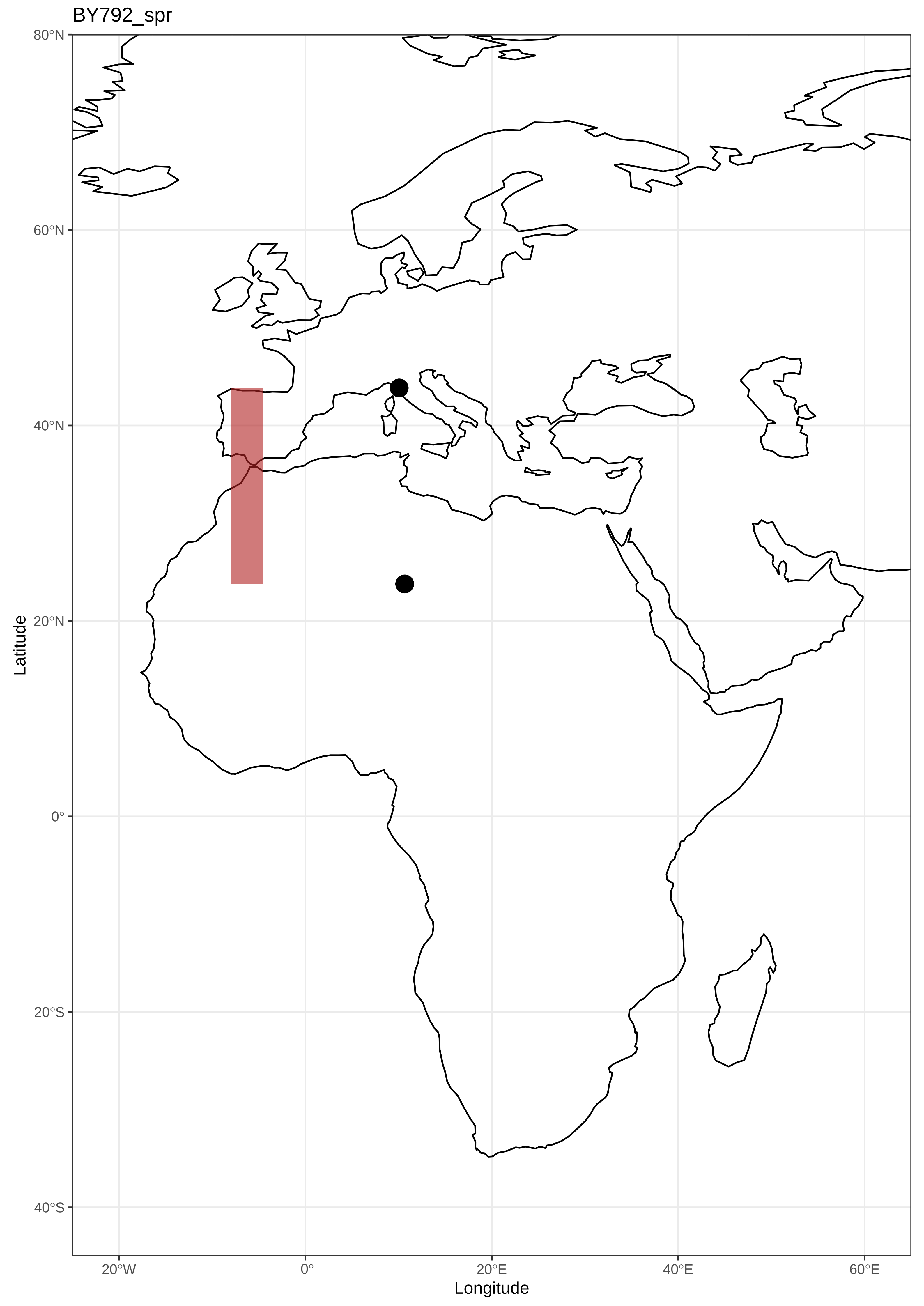

BY792_spr
80°N
60°N
40°N
20°N
0°
20°S
40°S
Latitude
20°W
0°
20°E
40°E
60°E
Longitude

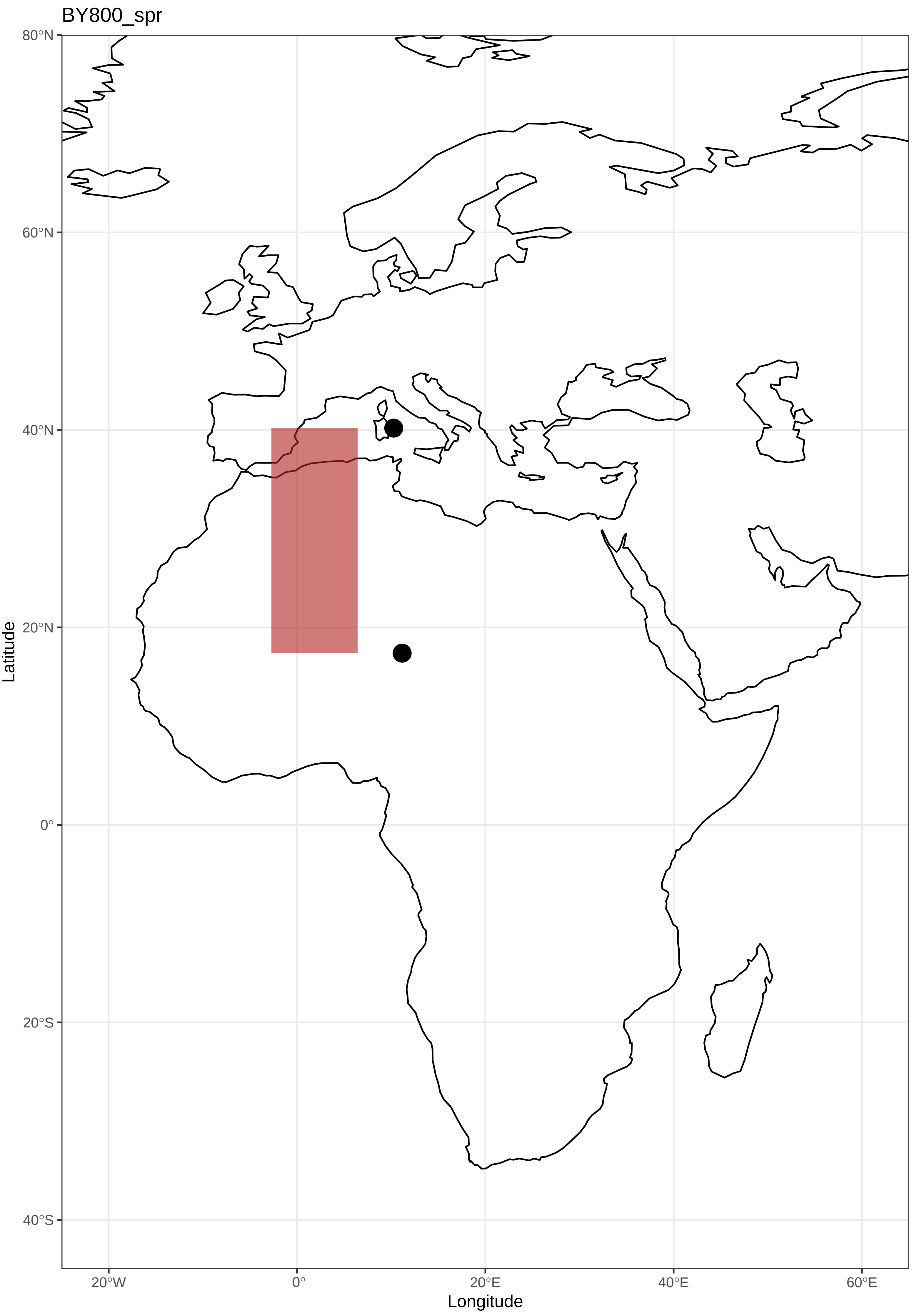
BY800_spr
80°N
60°N
40°N
20°N
0°
20°S
40°S
Latitude
20°W
0°
20°E
40°E
60°E
Longitude

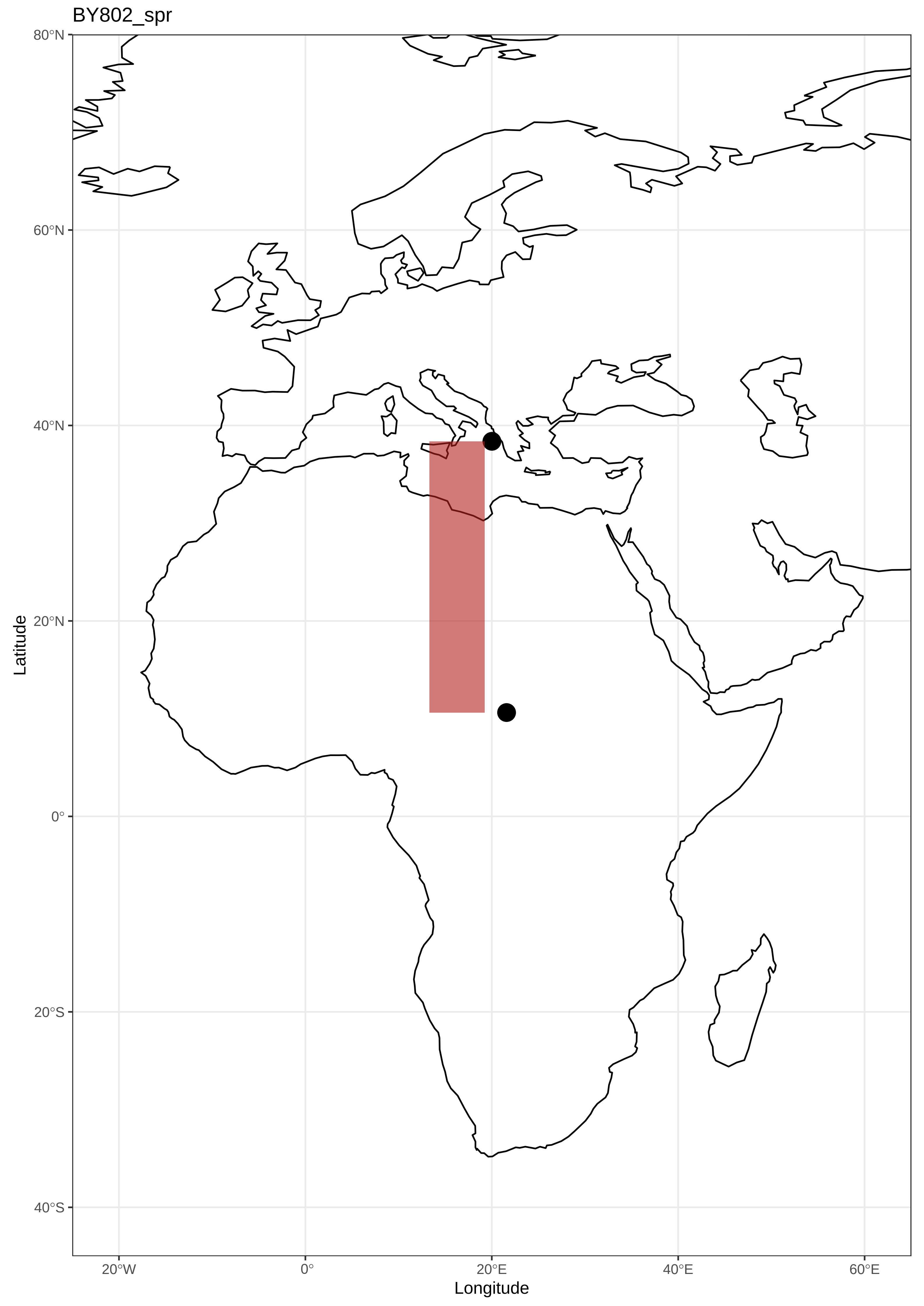

BY802_spr
80°N
60°N
40°N
20°N
0°
20°S
40°S
Latitude
20°W
0°
20°E
40°E
60°E
Longitude

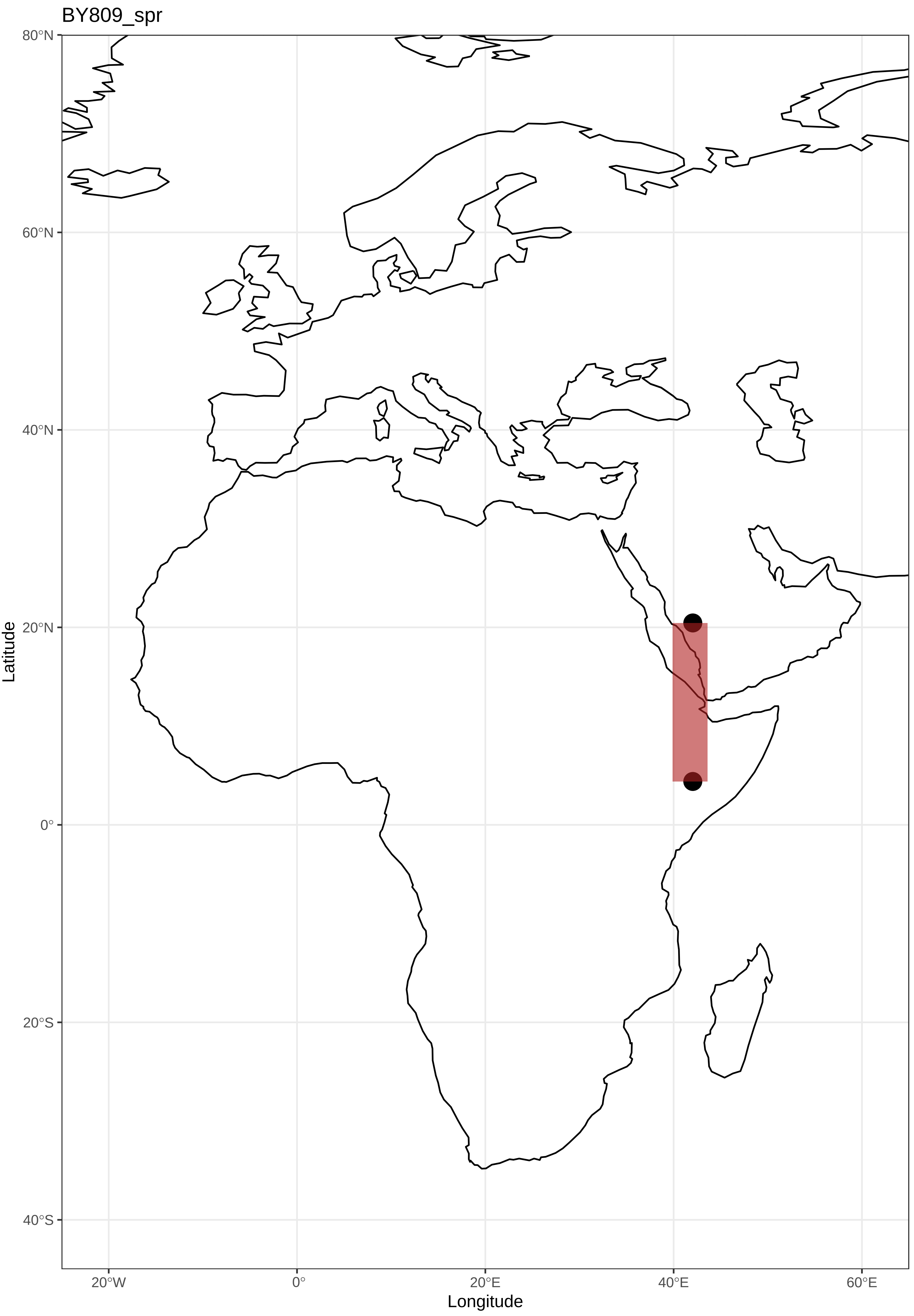
BY809_spr
80°N
60°N
40°N
20°N
0°
20°S
40°S
Latitude
20°W
0°
20°E
40°E
60°E
Longitude

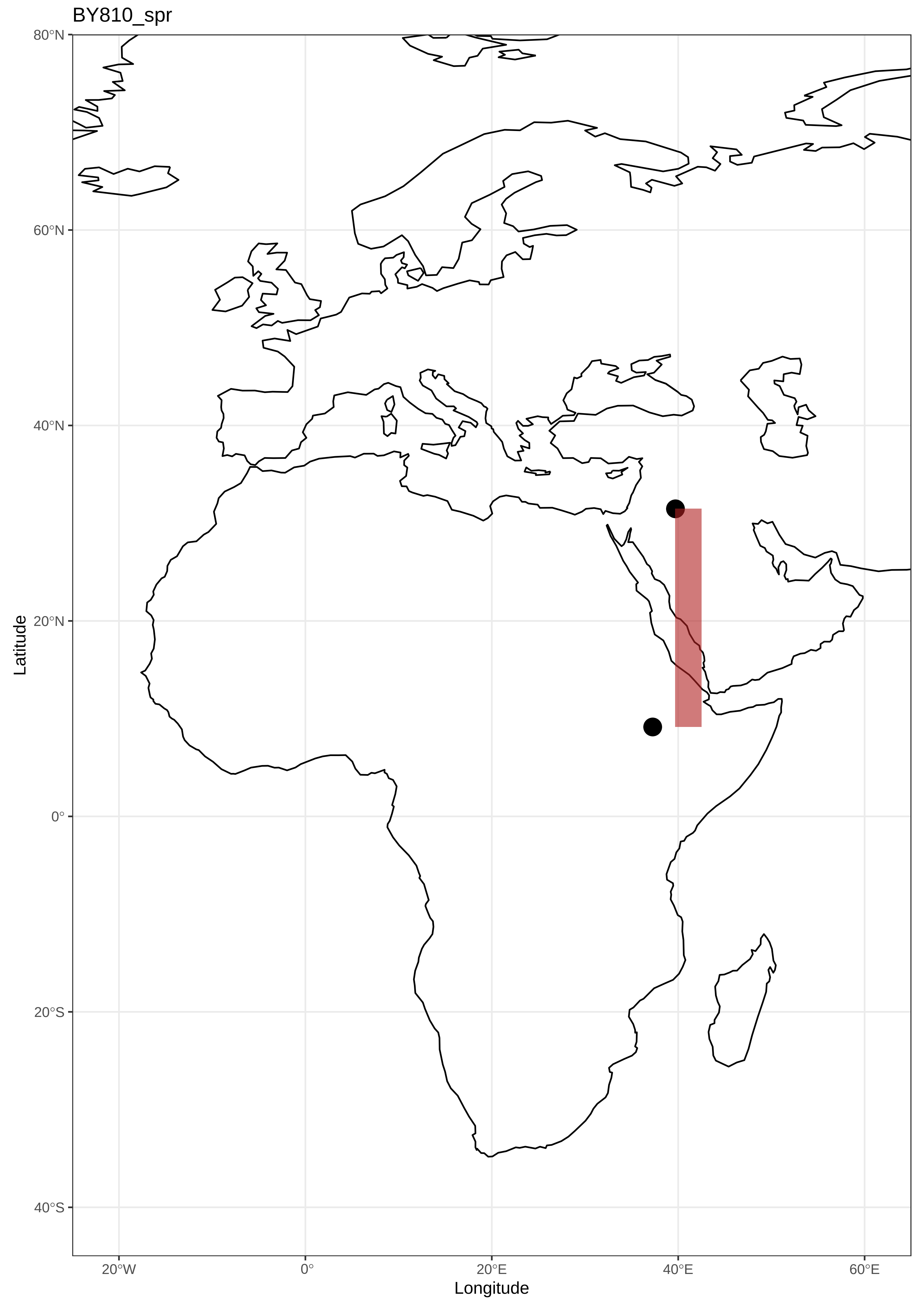
BY810_spr
80°N
60°N
40°N
20°N
0°
20°S
40°S
Latitude
20°W
0°
20°E
40°E
60°E
Longitude
